# Epigenetic and Metabolic Reprogramming of Fibroblasts in Crohn’s Disease Strictures Reveals Histone Deacetylases as Therapeutic Targets

**DOI:** 10.1093/ecco-jcc/jjad209

**Published:** 2023-12-09

**Authors:** Amy Lewis, David T Humphreys, Belen Pan-Castillo, Giulio Berti, Carla Felice, Hannah Gordon, Radha Gadhok, Anke Nijhuis, Shameer Mehta S, Liliane Eleid, Sidra Iqbal, Alessandro Armuzzi, Annamaria Minicozzi, Eleni Giannoulatou, Joanne ChinAleong, Roger Feakins, Virag Sagi-Kiss, Dora Barisic, Margarita-Ioanna Koufaki, Jacob G Bundy, James O Lindsay, Andrew Silver

**Affiliations:** Centre for Genomics and Child Health, Blizard Institute, Barts and The London School of Medicine & Dentistry, London E1 2AT, UK; Victor Chang Cardiac Research Institute, Sydney, NSW 2010, Australia; St Vincent’s Clinical School, University of New South Wales, Sydney, NSW 2052, Australia; Centre for Genomics and Child Health, Blizard Institute, Barts and The London School of Medicine & Dentistry, London E1 2AT, UK; Centre for Genomics and Child Health, Blizard Institute, Barts and The London School of Medicine & Dentistry, London E1 2AT, UK; Centre for Genomics and Child Health, Blizard Institute, Barts and The London School of Medicine & Dentistry, London E1 2AT, UK; Department of Internal Medicine University of Padua, Internal Medicine 1 Unit, Ca’ Foncello Hospital, Treviso, Italy; Centre for Immunobiology, Blizard Institute, Barts and The London School of Medicine & Dentistry, London E1 2AT, UK; Centre for Immunobiology, Blizard Institute, Barts and The London School of Medicine & Dentistry, London E1 2AT, UK; Centre for Genomics and Child Health, Blizard Institute, Barts and The London School of Medicine & Dentistry, London E1 2AT, UK; Centre for Genomics and Child Health, Blizard Institute, Barts and The London School of Medicine & Dentistry, London E1 2AT, UK; Centre for Genomics and Child Health, Blizard Institute, Barts and The London School of Medicine & Dentistry, London E1 2AT, UK; Centre for Genomics and Child Health, Blizard Institute, Barts and The London School of Medicine & Dentistry, London E1 2AT, UK; IBD Center, IRCCS Humanitas Research Hospital, Rozzano, Milan, Italy; Department of Colorectal Surgery, Division of Surgery & Perioperative Care, The Royal London Hospital, Whitechapel, London E1 1BB, UK; Victor Chang Cardiac Research Institute, Sydney, NSW 2010, Australia; St Vincent’s Clinical School, University of New South Wales, Sydney, NSW 2052, Australia; Department of Histopathology, The Royal London Hospital, London E1 1BB, UK; Department of Cellular Pathology, Royal Free London NHS Foundation Trust, London NW3 2QG, UK; Department of Metabolism, Digestion and Reproduction, Imperial College London, Burlington Danes Building, Du Cane Road, London W12 0NN, UK; Department of Metabolism, Digestion and Reproduction, Imperial College London, Burlington Danes Building, Du Cane Road, London W12 0NN, UK; Department of Metabolism, Digestion and Reproduction, Imperial College London, Burlington Danes Building, Du Cane Road, London W12 0NN, UK; Department of Metabolism, Digestion and Reproduction, Imperial College London, Burlington Danes Building, Du Cane Road, London W12 0NN, UK; Centre for Immunobiology, Blizard Institute, Barts and The London School of Medicine & Dentistry, London E1 2AT, UK; Centre for Genomics and Child Health, Blizard Institute, Barts and The London School of Medicine & Dentistry, London E1 2AT, UK

**Keywords:** Crohn’s disease, fibrosis, histone deacetylase, valproic acid, Collagen-I

## Abstract

**Background and Aims:**

No effective therapeutic intervention exists for intestinal fibrosis in Crohn’s disease [CD]. We characterized fibroblast subtypes, epigenetic and metabolic changes, and signalling pathways in CD fibrosis to inform future therapeutic strategies.

**Methods:**

We undertook immunohistochemistry, metabolic, signalling pathway and epigenetic [Transposase-Accessible Chromatin using sequencing] analyses associated with collagen production in CCD-18Co intestinal fibroblasts and primary fibroblasts isolated from stricturing [SCD] and non-stricturing [NSCD] CD small intestine. SCD/NSCD fibroblasts were cultured with TGFβ and valproic acid [VPA].

**Results:**

Stricturing CD was characterized by distinct histone deacetylase [HDAC] expression profiles, particularly *HDAC1*, *HDAC2*, and *HDAC7*. As a proxy for HDAC activity, reduced numbers of H3K27ac+ cells were found in SCD compared to NSCD sections. Primary fibroblasts had increased extracellular lactate [increased glycolytic activity] and intracellular hydroxyproline [increased collagen production] in SCD compared to NSCD cultures. The metabolic effect of TGFβ stimulation was reversed by the HDAC inhibitor VPA. SCD fibroblasts appeared ‘metabolically primed’ and responded more strongly to both TGFβ and VPA. Treatment with VPA revealed TGFβ-dependent and TGFβ-independent Collagen-I production in CCD-18Co cells and primary fibroblasts. VPA altered the epigenetic landscape with reduced chromatin accessibility at the *COL1A1* and *COL1A2* promoters.

**Conclusions:**

Increased HDAC expression profiles, H3K27ac hypoacetylation, a significant glycolytic phenotype and metabolic priming characterize SCD-derived as compared to NSCD fibroblasts. Our results reveal a novel epigenetic component to Collagen-I regulation and TGFβ-mediated CD fibrosis. HDAC inhibitor therapy may ‘reset’ the epigenetic changes associated with fibrosis.

## 1. Introduction

Our understanding of the molecular, epigenetic, and metabolic mechanisms driving fibrosis and stricture formation in Crohn’s disease [CD] is incomplete limiting the identification of novel therapeutic targets. Surgical resection remains the main therapeutic strategy for symptomatic fibrotic strictures. Fibrosis is a transmural process marked by increased extracellular matrix [ECM] deposition and thickening of the submucosa and smooth muscle layers mediated by fibroblasts, the primary source of ECM proteins.^1^ TGFβ promotes fibroblast activation, ECM synthesis [e.g. Collagen-I production], and development of stricturing CD [SCD].^2,3^ Importantly, TGFβ impacts, and is modulated by, fibroblast metabolism.^4–6^

Histone deacetylases [HDACs] are epigenetic regulators of gene expression and are potential therapeutic targets in a spectrum of diseases including inflammation and cancer. Pharmacologically diverse HDAC inhibitors exist.^7,8^ Valproic acid [VPA] targets Class I [HDAC1–3 and 8] and Class IIa [HDAC4–5, 7 and 9] HDACs,^9^ and can restore pathologically low levels of histone-3 acetylation at lysine 27 [H3K27ac], a recognized surrogate marker of HDAC activity.^10–12^ Recently, we validated these observations in inflammatory bowel disease [IBD] patients, where active inflammation was associated with reduced H3K27ac.^13^ VPA inhibits fibrosis in numerous disease models,^14–16^ but its mechanism of action is unclear.

Here we explore changes in histone acetylation, HDAC expression, and the metabolic signatures associated with SCD and non-stricturing [NSCD] regions of the CD small bowel. The impacts of VPA-induced HDAC inhibition on the epigenome, metabolome, gene expression, and pro-fibrotic signalling pathways are assessed in cell lines and primary CD fibroblast cultures derived from surgical resection specimens. Our findings highlight the significance of the epigenome and metabolome in intestinal fibrosis and provide an important pre-clinical basis to support HDAC inhibitor use in treating CD intestinal fibrosis.

## 2. Methods

### 2.1. Ethics approval

Appropriate local Ethics Committee approvals [London—City Road & Hampstead Research Ethics Committee; 15/LO/2127] and informed consent were obtained prior to patient recruitment.

### 2.2. Primary CD fibroblasts isolation, cell culture, and treatments

Primary CD fibroblasts were isolated as previously described.^17^ Adherent cells were maintained in Dulbecco’s modified Eagle medium [DMEM] under standard culture conditions and experiments were performed with cells from passages 2–4. CCD-18Co [normal human intestinal fibroblasts; ATCC CRL-1459] were cultured as described previously.^17^ Experiments using CCD-18Co were performed in quadruplicate over independent passages. For TGFβ1 assays, CCD-18Co cells and/or primary fibroblasts were serum-starved overnight before treatment with recombinant human TGFβ1 [10 ng/mL, R&D Systems] reconstituted in PBS. For siRNA-mediated gene knockdown in 96-well plates, cells were transfected with an siRNA targeted against TGFβ1|1 [25 nM, SC-37685] or a non-targeted control [NTC, 25 nM, SC-37007] using DharmaFECT 2 reagent [T-2002-02]. Transfections were performed according to the Dharmacon DharmaFECT 1–4 transfection protocol, using 0.1 µL of DharmaFECT 2 per reaction. Differences between treatments were determined using a paired *t*-test matched for passage.

### 2.3. Metabolism experiments: sample handling, QC processes, and data acquisition using ^1^H NMR spectroscopy

Following culture as above, medium was aspirated and stored, and cell samples were harvested into 2 mL of ice-cold methanol per plate, frozen and kept at –80°C. The amine sub-metabolome was analysed by LC-MS with pre-column derivatization, as described.^18^ Samples were handled in a randomized order. Thawed cell samples [>1.5 mL] were transferred to glass Corning tubes, and 0.8 mL chloroform was added and mixed by vortexing; then 0.8 mL water and 0.8 mL chloroform was added to split the phases, and centrifuged [2500 *g* for 10 min at room temperature]. An aliquot [450 µL] of the top polar layer was transferred to microcentrifuge tubes, dried under reduced pressure, and stored at −80°C prior to analysis. The dried extracts were reconstituted in 30 µL water, centrifuged and 10 µL was used for the assay. 6-Aminoquinolyl-*N*-hydroxysuccinimidyl carbamate [AccQTag Ultra, Waters,] derivatization was performed according to the manufacturer’s protocol [1:7:2 ratio of sample/borate buffer/AccQTag reagent]. Then, 10 µL of each derivatized sample was combined to give a single biological quality control [QC] sample. Targeted LC-MS data were acquired on a Waters Acquity LC system with XEVO TQ-S triple quadrupole mass spectrometer [Waters] operated in positive MRM mode. An HSS T3 UHPLC column [Waters] was used with 0.1% formic acid in water, and 0.1% formic acid in acetonitrile as mobile phases. Solvent blanks and process blanks were monitored to check that signals did not result from contaminants; a run of QC samples were injected before and after the run, and during the run, every 10th injection was a QC sample. The QC data were used to ensure data quality for all metabolites; any metabolites with a coefficient of variance >0.3 for the QC samples were excluded.

The exometabolomic data were acquired using ^1^H nuclear magnetic resonance [NMR] spectroscopy following established protocols.^19^ Briefly, 0.7 mL of supernatant was added to 0.2 mL of sodium phosphate buffer [pH 7, 0.5 M] and 0.1 mL of IS-2 [Chenomx] containing 0.5 mM 3-[trimethylsilyl]-1-propanesulfonic acid-d_6_ [DSS], mixed and centrifuged [16 000 *g*, room temperature, 5 min]. The supernatant [0.6 mL] was then transferred to 5-mm SampleJet NMR tubes and analysed on an Avance DRX600 NMR spectrometer [Bruker] equipped with a SampleJet autosampler and 5-mm inverse probe. Samples were maintained at 4°C on the autosampler and 27°C during acquisition; tuning, matching, and calibration of the 90° pulse length were carried out for each sample independently. Data were acquired using the noesygppr1 pulse sequence for water suppression, using eight dummy scans and 32 total scans with an overall recycle delay of 5 s and processed using a Bruker AU program for phase and baseline correction and referencing of the DSS singlet [δ = 0]. Processed spectra were then read into NMR Suite 8.3 [Chenomx], and absolute metabolite concentrations were generated by computer-assisted manual fitting.

### 2.4. Analysis of metabolomic data

Exometabolomic data for the CCD-18Co line were analysed using a linear mixed effects model, with passage as a random effect and VPA, TGFβ, and VPA × TGFβ as fixed effects. The endometabolomic data were represented as fold-changes for each passage [calculated against the global mean for that passage], and the log-transformed fold-changes were then analysed by two-way ANOVA, with VPA, TGFβ, and VPA × × TGFβ as factors. The exo- and endometabolomic data for the primary cells were analysed by a linear model, with SCD, VPA, TGFβ, and VPA × TGFβ as factors. Next, the endometabolomic data were also analysed as log-transformed fold-change data [as above], with the SCD and NSCD groups analysed separately; and the exometabolomic data were analysed as a linear model with cell culture, VPA, TGFβ, and VPA × TGFβ as factors for the SCD and NSCD groups separately.

### 2.5. TaqMan qPCR, histology, fibrosis and ulcer scoring, and immunohistochemistry

RNA from NSCD and SCD tissue was isolated from surgically resected tissue. RNA was converted to cDNA using a ‘High Capacity RNA-to-cDNA Kit’ [Applied Biosystems], diluted 1:10, and incubated with Taqman gene expression probes and Universal Mastermix [Applied Biosystems] on a 7500 System RealTime PCR cycler [ABI]. Cycle threshold [Ct] values were exported and normalized to control genes [e.g. RPLPO and/or ACTB] using the 2-ΔCt method. All normalized data were log_2_ transformed prior to statistical analysis.

Formalin-fixed paraffin-embedded [FFPE] surgical resection tissue from the ileum of CD patients with a documented stricture were obtained from Barts Health NHS Trust pathology archive. From each CD patient, SCD and NSCD FFPE blocks were identified from pathology reports and relative levels of inflammation and fibrosis were assessed by a pathologist [R.F.], as described previously.^20^ TGFβ1|1 immunohistochemistry [IHC] was performed by Pathognomics Ltd, using protocols optimized for the automated Ventana stains system [Roche].

### 2.6. Immunofluorescence [IF], western blots, and ELISAs

Cells were seeded in a 96-well plate [2500 cells per well]. Following treatment cells were fixed, permeabilized, and stained as described.^21^ Antibodies and conditions used for IF staining were: Collagen-I [1:500 dilution; NB600-408, Novus Biologicals] and TGFB1|1 [1:250, ab42476, Abcam; note: no longer available but archived datasheets are downloadable and alternatives indicated by Abcam] were incubated overnight at 4°C. The secondary antibody mix consisted of: CellMask Deep Red [1:100 000, Applied Biosystems] and DAPI [1 ng/mL], Alexa Fluor 488-conjugated anti-rabbit antibody [1:500 dilution; Invitrogen], and/or Alexa Fluor 555-conjugated anti-mouse antibody [1:500 dilution; Invitrogen].

Stained cells in PBS were imaged on an IN Cell 2200 Analyser [GE Healthcare]. IN Cell Developer software [V1.9] was used to process and analyse images, and calculate protein density levels on a per-cell basis. Background was corrected by subtracting the values from the secondary antibody-only control.

Total protein was extracted from CCD-18Co and human fibroblast samples with RIPA buffer [R0278] and protein quantification was determined by a DC Protein Assay [5000111]. Antibody and blotting conditions are given for each protein in Supplementary Table S1. Immunoreactive band intensity was analysed using a computer-assisted scanning densitometry [Image-Lab 3.0.1, Bio-Rad Laboratories]. Pro-collagen-1α1 levels were measured using Human Pro-Collagen I alpha 1 DuoSet ELISA following the manufacturer’s instructions [DY6220-05, R&D Systems]. Data were log_2_ transformed prior to statistical analysis.

### 2.7. 3D organotypic model

Methods used are as described previously.^17^

### 2.8. General statistics

Statistics for metabolomic experiments are given above [Analysis of metabolomic data] and in the figure legends. Experiments involving CCD-18Co fibroblast cells (qPCR, IF, western blot, and ELISAs) were performed in quadruplicate over four independent cell passages, unless stated otherwise in the figure legend. Differences post-treatment were evaluated using a paired *t*-test to account for cell passage. In general, data are presented as fold-changes and significant results relative to control are indicated by the symbol * or ^$^ [*p < 0.05, **p < 0.01, *** *p* < 0.001, **** *p* < 0.0001]. Solid bars indicate comparisons between particular sets of treatments. The symbol is given above the bar. To standardize variance, qPCR data were log transformed before statistical analysis. Treatment effects in primary fibroblast cultures were determined by a paired *t*-test.

## 3. Results

### 3.1. Increased HDAC expression and hypoacetylation of H3K27ac is associated with stricture formation in CD

HDAC mRNA expression (including Class I and Class IIa HDACs and known targets for VPA such as HDAC2, 4 and 7) was increased in the mucosa overlying SCD compared to NSCD intestinal segments [*n* = 6].^9,17^ In addition, collagen gene expression was significantly increased in SCD compared to NSCD mucosa [Table 1]. Consequently, HDAC expression was assessed in our previously published scRNA-seq dataset [Array Express accession code: https://www.ebi.ac.uk/biostudies/arrayexpress/studies/E-MTAB-11792] from primary fibroblasts isolated from full-thickness sections of SCD and NSCD ileum.^22^ This revealed distinct profiles of HDAC expression associated with the four fibroblast clusters, notably for HDAC2 and HDAC7 [Supplementary Figure S1A and B], as categorized by differentiation trajectory analysis of the two Lumican-positive fibroblast subtypes identified from our scRNA-seq data reported previously.^22^ These findings support the data given in Table 1. HDAC2 localization was documented using IHC in FFPE sections from SCD and NSCD intestine isolated from CD patient resection specimens [Supplementary Table S2]. HDAC2 exhibited an ‘on’ or ‘off’ expression pattern [Supplementary Figure S1C]; scoring of positive cells identified an increase in HDAC2-positive stromal cells in the mucosa overlying SCD tissue relative to paired NSCD controls [Supplementary Figure S1D].

**Table 1.** Fold expression change in HDACs and collagen genes in stricturing compared to non-stricturing CD intestine. RNA was isolated from the mucosa overlying SCD and patient-matched NSCD intestine [*n* = 6] as described.^17^ The mRNA levels are normalized to the housekeeping gene *RPLPO*. Differences were determined by a paired *t*-test. Significant results are indicated by asterisks [*p < 0.05, **p < 0.01].

	Gene	Fold change [SCD/NSCD]	*p*-value	Significance
Class I	HDAC1	0.761	0.567	
	HDAC2	2.090	0.097	
	HDAC3	1.159	0.912	
	HDAC8	1.635	0.268	
Class IIA	HDAC4	2.440	0.002	**
	HDAC5	1.704	0.181	
	HDAC7	3.342	0.012	*
	HDAC9	3.753	0.071	
Class IIB	HDAC6	1.449	0.803	
	HDAC10	0.919	0.486	
Collagens	COL1A1	16.486	0.008	**
	COl1A2	5.734	0.010	*
	COL3A1	6.285	0.005	**
	COL5A2	5.074	0.008	**

HDAC, histone deacetylase; SCD, structuring CD; NSCD, non-structuring CD.

**Figure 1.**
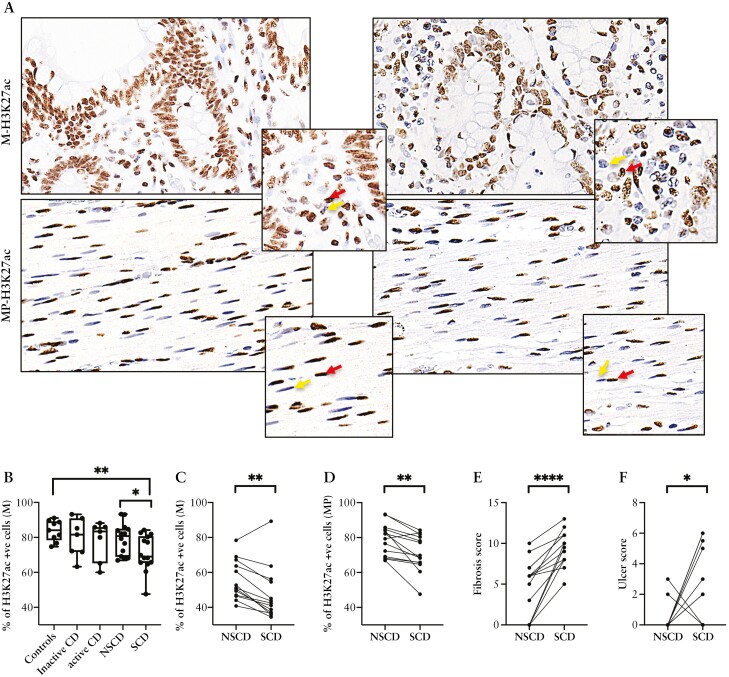
Reduction of H3K27ac levels in stricturing [SCD] and non-stricturing [NSCD] intestine. [A] Representative H3K27ac IHC images for mucosal [M-H3K27ac] and *muscularis propria* [MP-H3K27ac] [top and bottom panels respectively; 400× magnification] for NSCD (left) and SCD (right) tissue. Examples of positively and negatively stained stromal cells highlighted by red or yellow arrows, respectively. [B] Box and whisker plot showing percentage of H3K27ac-positive cells [H3K27ac+ve] in the mucosa [M] [*n* = 14] of non-inflamed (inactive) and inflamed (active) SCD and NSCD samples compared to control. [C] Comparison of H3K27ac-positive cells in the mucosa [M] and [D] *muscularis propria* (MP) of NSCD and SCD tissue. [E] Fibrosis scores and [F] ulcer scores in SCD formalin-fixed paraffin-embedded sections relative to patient-matched NSCD sections [*n* = 14 pairs]. Box plots show 25th to 75th percentiles, median [horizontal bar], and lowest and highest value [whiskers]. Differences between SCD and NSCD samples were determined by a paired *t*-test. Significant results are indicated by asterisks [*p < 0.05, **p< 0.01, ****p < 0.0001].

To assess the functional consequence of differential HDAC expression, the fraction of H3K27ac-positive cells was scored in SCD paired with adjacent NSCD samples, as well as healthy and CD controls [Figure 1A; Supplementary Table S2]. This method was used previously to assess the effects of HDAC inhibitors in IBD mouse models and human IBD tissues.^13^ Numbers of H3K27ac+ cells in the mucosa of SCD samples were reduced relative to healthy controls [Figure 1B]. This was confirmed in a paired sub-analysis of SCD intestinal samples compared to NSCD control samples from the same patient. A significant reduction in H3K27ac+ cells in the SCD mucosa and *muscularis propria* was demonstrated [Figure 1C and D]. This was consistent with increased HDAC activity throughout the stricture and linked to a marked increase in the histological degree of fibrosis, but much less in tissue ulceration scores as a marker of inflammation [Figure 1E and F]. Moreover, there was no difference in the percentage of H3K27ac+ cells in inflamed CD tissue relative to either healthy controls or inactive CD controls, consistent with our previously published work [Figure 1B].^13^ Collectively, our data highlight a reduction in H3K27ac consistent with an increase in Class I HDAC expression in SCD.

### 3.2. HDAC inhibition by VPA inhibits TGFβ-inducible changes in pro-fibrotic metabolites in cell lines and primary CD fibroblasts

To determine the role of HDAC enzymes and the impact of TGFβ on the metabolic activity of fibroblasts, the HDAC inhibitor VPA was tested in CCD-18Co intestinal fibroblasts and primary CD fibroblasts treated with or without TGFβ. To assess the drug’s effect on cellular metabolism, a dose of 5 mM VPA was selected based on prior use in IBD mouse models and cultured IBD human tissue explants.^11,13^

**Figure 2.**
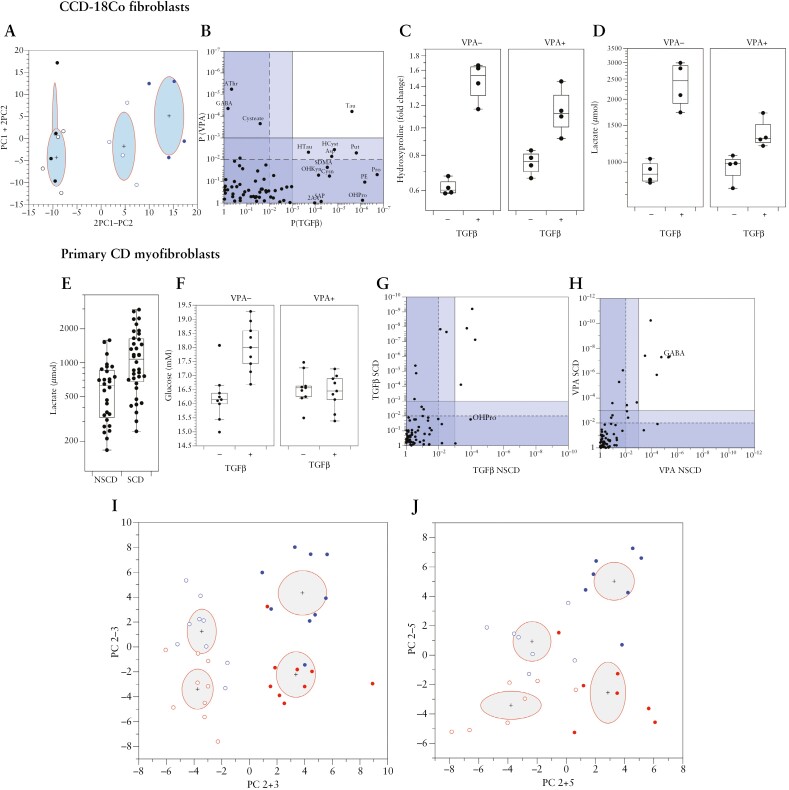
Stricturing [SCD] and non-stricturing [NSCD] Crohn’s disease primary fibroblast cultures are glycolytically and metabolically distinct. [A–D] TGFβ induces metabolic changes in CCD-18Co cells. [A] Metabolic changes are partially reversed by VPA. Plot shows principal component [PC] scores 1 and 2 [rotated to show group discrimination most clearly]. Blue: TGFβ added; black: no TGFβ; empty symbols: VPA added; filled symbols: no VPA. Ellipses represent standard deviations and the mean is indicated by a cross. [B] Metabolic effect of TGFβ is greater than VPA for CCD-18Co cells, and largely affects different individual metabolites. GABA is affected by VPA through an HDAC-independent mechanism, and is a positive control; here, it was significantly changed by VPA, *p* = 4.3e-5; *p* values from two-way ANOVA. [C] Hydroxyproline is increased by TGFβ treatment in CCD-18Co cells, partially counteracted by VPA treatment [*p* = 0.0073, interaction term, two-way ANOVA]. [D] TGFβ increases glycolytic activity in CCD-18Co cells, as evidenced by increased extracellular lactate, which is partially reversed by VPA [*p* = 0.0012, interaction term, linear mixed effects model]. [E] SCD and NSCD primary fibroblast cultures are significantly differentiated from each other in terms of glycolytic activity, as evidenced by extracellular lactate production [*p* = 0.00028, ANOVA, with SCD/NSCD, TGFβ, and VPA as factors]. [F] TGFβ treatment decreases glucose utilization in SCD primary cell cultures, reversed by VPA [*p* = 0.00094, interaction term, linear mixed effects model]. [G] TGFβ [left] has a similar metabolic effect in SCD and NSCD cultures, but effects are greater for SCD. [H] The same is true for VPA [right], although metabolic changes are different from TGFβ. Solid lines indicate a *p*-value threshold of 0.001 and dashed lines a threshold of 0.01. [I, J] PC analysis of endometabolome data indicates that, for SCD [I] and NSCD [J] cultures, there is an overall metabolic effect of both TGFβ and VPA; these effects are largely independent. Blue: TGFβ added; red: no TGFβ; empty symbols: VPA added; filled symbols: no VPA. Ellipses represent standard deviations, and the mean is indicated by a cross. NB. Separation occurs within the first three components for SCD and first five components for NSCD cells. Components are rotated to show separation more clearly.

To visualize patterns or clusters in the data we used principal components analysis [PCA] as an unsupervised approach to dimension reduction. This gave a picture of the overall metabolic effects, across all the metabolites analysed. The tight clustering of pooled QC samples indicated the high technical quality and reproducibility of the data [Supplementary Figure S2A–C]. Based on their metabolite profiles, the CCD-18Co cultures were separated by TGFβ treatment within the first two principal components [PCs]. The effect was reversed partially, but not completely, by VPA [Figure 2A]. When considered along a composite axis [2PC1–PC2, Figure 2A], TGFβ treatment induced significant differences [two-way ANOVA, *p* = 7.5e-10], and the interaction term, which assessed whether the effect of TGFβ was the same in the presence or absence of VPA, was also highly significant [*p* = 0.0008] indicating that VPA did indeed alter the response to TGFβ. Analysis of individual metabolites confirmed the PCA results, with more metabolites significantly altered by TGFβ than by VPA [13 compared to four metabolites with *p* < 0.001; Figure 2B; Supplementary Table S3]. Importantly, one of the most significantly affected by TGFβ [*p* = 8.3e-7] was hydroxyproline [OHPro], a marker for collagen production. The interaction term *p* values were generally less significant than the main effects of either TGFβ or VPA, but there were five metabolites for which VPA significantly reversed the effect of TGFβ including OHPro [Figure 2C], ophthalmic acid, asparagine, aspartate, and 5-aminopentanoate [Supplementary Figure S2D]. In the exometabolome analysis, VPA partially supressed a TGFβ-induced shift towards glycolysis marked by changes in lactate production (linear mixed effects model [LME]; interaction term, *p* = 0.0012) [Figure 2D].

These analyses were repeated in primary CD patient-derived intestinal fibroblast cultures derived either from SCD or NSCD resected tissue [*n* = 9 and *n* = 7, respectively; Supplementary Table S4]. For some patients, multiple cultures were derived from distinct sections of the intestine and each of these cultures were treated as independent as reported.^17^ Multivariate and univariate approaches determined the effects of fibroblast origin [SCD or NSCD], and the effects of treatments [TGFβ and VPA], as well as the interactions between them.

SCD fibroblasts were metabolically distinct from NSCD fibroblasts in both the exo- and endometabolomic data. An increase in extracellular lactate [*p* = 0.00028] in SCD compared to NSCD fibroblasts, independent of the treatment effects [all *p* > 0.05], indicated a clear shift towards a glycolytic phenotype [Figure 2E]. SCD and NSCD cell cultures were also separated for intracellular metabolites along PC1 [*p* = 0.0008, *t*-test]. PCA again showed excellent technical replication [Supplementary Figure S2B]. This difference along PC1 was maintained even when the effects of VPA and TGFβ were allowed for [*p* = 0.0011, ANOVA]. Differences in several intracellular metabolites were observed in SCD and NSCD cell cultures [Supplementary Table S5], with ten metabolites with *p* < 0.001, 20 metabolites with *p* < 0.01, and 30 metabolites with false discover rate [FDR] < 0.05 [after treatment effects had been allowed for; ANOVA]. Importantly, these included OHPro, which was increased in the SCD samples [*p* = 0.0016]. Given these baseline differences, the NSCD and SCD samples were analysed separately.

The exometabolomic response of SCD was much greater than for NSCD cells. Glucose levels did not change due to TGFβ or VPA treatment in the NSCD cells [*p* > 0.05 for all, LME]. However, in SCD cells, TGFβ treatment led to a significant increase in glucose (i.e. a decrease in glucose utilization, *p* = 0.00049, which was wholly abrogated by VPA [*p* = 9.9e-4, interaction term; Figure 2F]). Similarly, there was only a borderline effect of TGFβ on lactate in NSCD cultures [*p* = 0.049, LME], whereas the SCD cultures had a highly significant [*p* = 4.1e-6] increase in lactate response to TGFβ, which was decreased in response to VPA [*p* = 0.001]; the interaction term was not significant, but the SCD cells treated with both VPA and TGFβ had similar levels of lactate to the untreated cells [Supplementary Figure S2E].

The effect of TGFβ on endometabolomic profiles was also greater in SCD than NSCD cell cultures, with nine metabolites different at *p* < 0.001 in the SCD background, compared to only six different at *p* < 0.001 in the NSCD background [four in common], and more significant *p* values overall for the SCD cells [Figure 2G; Supplementary Table S6]. The same was true for VPA treatment, with 12 and eight metabolites significant at *p* < 0.001 in the SCD and NSCD backgrounds, respectively [six in common], and more significant *p* values for the SCD than NSCD samples. GABA, as a positive control unrelated to HDAC activity, was highly significant in both backgrounds [Figure 2H; Supplementary Table S6]. Again, the interaction terms were much less significant than the main effect. Whilst both VPA and TGFβ treatments had clear metabolic effects, there was little statistical evidence that VPA was tending to counteract the TGFβ effect. However, there were three metabolites with an interaction between TGFβ and VPA with *p* < 0.01 on the SCD background; there were no metabolites which met this threshold in NSCD cells. The most significant interactions were for OHPro [*p* = 0.019] and cystathionine [SCD cells *p* = 0.0011; NSCD cells *p* = 0.022; Supplementary Figure S2F and G]. For the SCD group, there was a separation of the treatment groups within the first three PCs, with a very clear effect of VPA across PCs 2 and 3 [perfect separation of the groups along PC2 + 3, *p* = 1.7e-13], and an orthogonal effect of TGFβ [PC2–3, *p* = 1.6e-6]. The group separations were less clear for the NSCD group, but there was separation within the first five PCs [Figure 2I and J].

Therefore, SCD cells are metabolically distinct from NSCD cells at baseline with increased glycolytic activity [extracellular lactate] and collagen production [intracellular OHPro]. In addition, they are ‘metabolically primed’, in that they also respond more strongly to TGFβ and VPA treatment. Overall, VPA tended to counteract the metabolic effects of TGFβ and, in particular, changes in OHPro.

### 3.3. VPA increases histone acetylation, inhibits TGFβ signalling, and suppresses Collagen-I in intestinal fibroblasts

**Figure 3.**
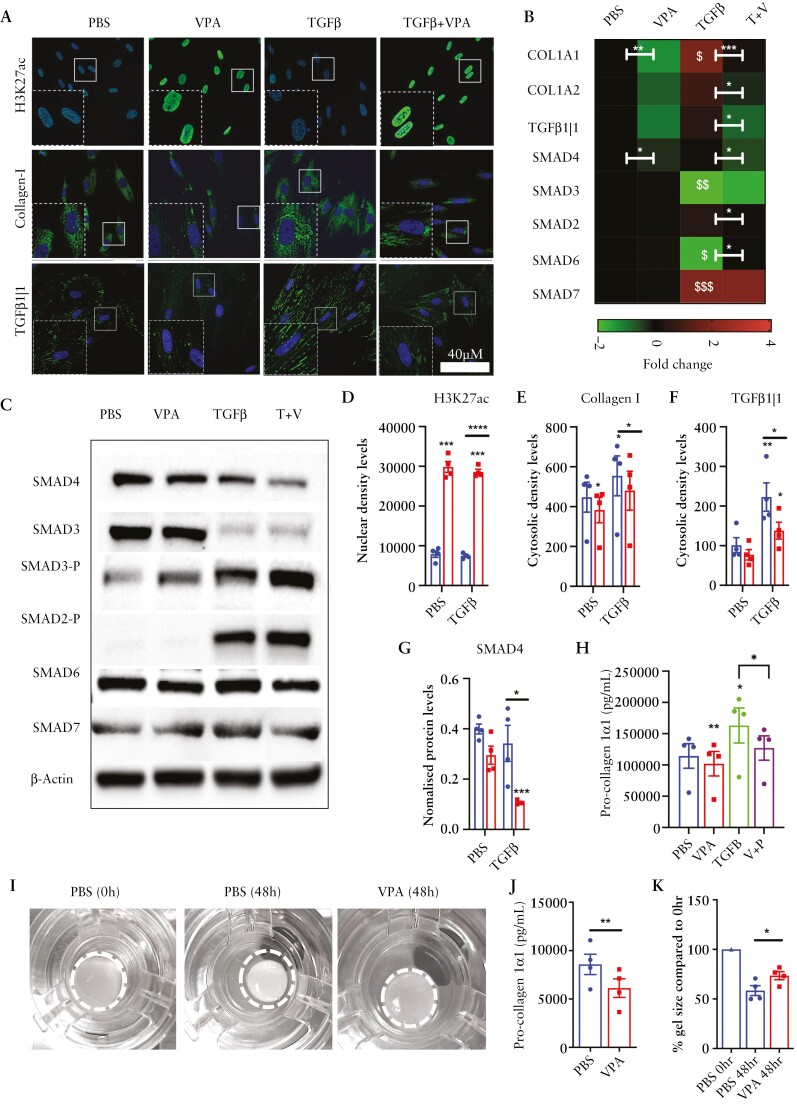
VPA impacts SMAD signalling, its downstream target TGFβ1|1, and reduces collagen in TGFβ-stimulated intestinal fibroblasts. [A] Representative immunofluorescent [IF] images of H3K27ac, Collagen-I, and TGFβ1|1 in CCD-18Co cells treated with vehicle control [PBS] or TGFβ1 in combination with VPA. [B] Heatmap showing mRNA levels of key selected genes in VPA- and TGFβ-treated cells [*n* = 4]; *significant differences associated with VPA treatment, ^$^significant differences associated with TGFβ treatment. [C] Representative western blots for SMAD expression in cells treated with VPA and TGFβ [*n* = 3; SMAD4, *n* = 4]. [D–F] Protein quantification bar graphs from IF images [*n* = 4]. [G] SMAD4 protein level normalized to β-Actin loading control. [H] Pro-collagen-1α1 in conditioned media measured by ELISA [*n* = 4]. [I] VPA inhibits gel contraction by CCD-18Co fibroblasts. 3D gel contraction assays used 15 000 CCD-18Co cells and a Caco2 epithelial cell barrier in the presence or absence of 5 mM VPA or vehicle control [PBS] for 48 h. [J, K] Gel size was quantified after 48 h and Pro-col 1α1 levels in conditioned media measured by ELISA. Differences between treatments were determined by a paired *t*-test. Significant are results indicated by * or ^$^ symbols [* or ^$^*p* < 0.05, ** or ^$$^*p* < 0.01, *** or ^$$$^*p* < 0.001 and **** or ^$$$$^*p* < 0.0001].

The functional impact of inhibiting HDAC activity on TGFβ signalling and Collagen I production was assessed in both CCD-18Co cells and primary SCD and NSCD cultures [Figure 3]. In CCD-18Co fibroblasts, VPA treatment significantly increased the positive control H3K27ac [Figure 3A and D] and inhibited basal and TGFβ-induced up-regulation of Collagen-I at both the mRNA and protein levels, consistent with the changes in OHPro in the endometabolome [Figure 3A, B, and E]. Consistent with a role for the SMAD signalling pathway in fibrosis, TGFβ increased fibroblast SMAD2 and SMAD3 phosphorylation. In contrast, VPA inhibited mRNA expression and protein levels of SMAD4 and its downstream target TGFβ1|1 [Figure 3A–C, F, and G; Supplementary Figure S3]. However, levels of phospho-SMAD2 and phospho-SMAD3 as well as the inhibitory SMAD proteins SMAD6 and SMAD7 were unaffected by VPA treatment [Figure 3C; Supplementary Figure S4]. SMAD4 suppression alone was unlikely to account for the observed reduction in Collagen-I by VPA, since selective knockdown of SMAD4 in CCD-18Co cells reduced stimulated TGFβ1|1 expression, but did not impact the effect of TGFβ or VPA on Collagen-I expression, implying VPA-mediated inhibition of Collagen-I is regulated by a different mechanism [Supplementary Figure S3]. This hypothesis was reinforced by the analysis of secreted proteins: VPA inhibited Pro-collagen-1α1 in both control and TGFβ-stimulated cells [Figure 3H], which indicated that VPA has both TGFβ-dependent and TGFβ-independent actions. The effects on Pro-collagen-1α1 in the absence of TGFβ-stimulation were validated in a 3D organotypic gut model, where VPA also inhibited gel contraction [Figure 3I–K].

**Figure 4.**
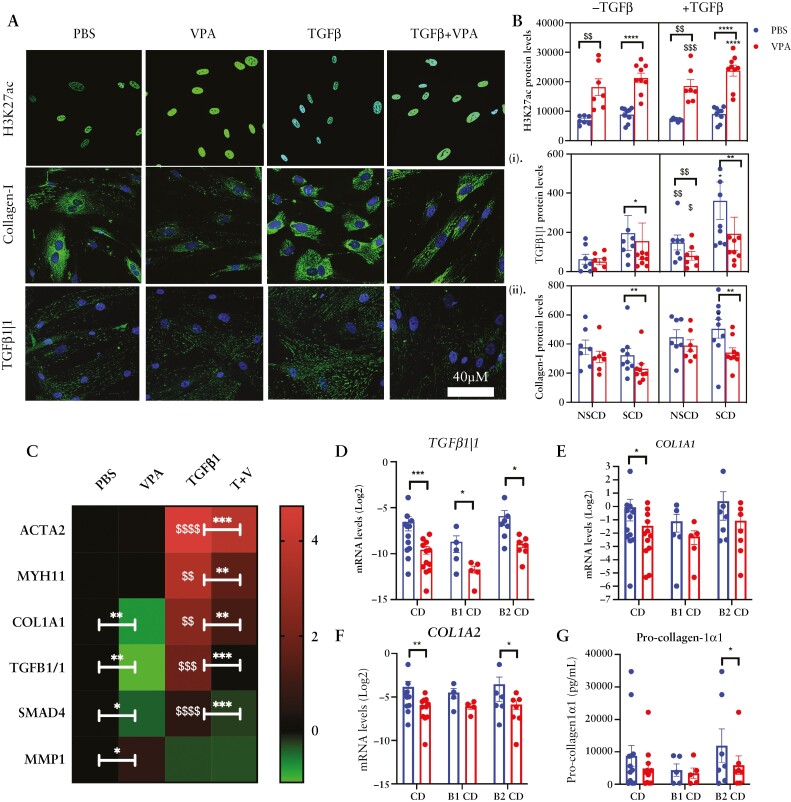
Anti-fibrotic actions of VPA in primary CD patient-derived intestinal fibroblasts. [A] Representative immunofluorescent [IF] images of H3K27ac, Collagen-I, and TGFβ1|1 in primary CD fibroblast cultures treated with vehicle control [PBS] or TGFβ1 in combination with VPA. [B] IF protein quantification bar graphs for cultures derived from NSCD [*n* = 7] or SCD [*n* = 9] intestinal segments and if stimulated with TGFβ1 or vehicle control [PBS]. [C] Heat map showing differences between treatments for fibroblast activation markers. *Significant differences associated with VPA treatment; ^$^significant difference associated with TGFβ treatment. [D–F] *TGFβ1|1*, *COL1A1*, and *COL1A2* mRNA levels in cultured CD mucosal biopsies treated with TGFβ and/or VPA for 48 h [*n* = 9]. B1, inflammatory phenotype; B2, SCD phenotype. [G] Pro-collagen-1α1 levels in conditioned media measured by ELISA [*n* = 8]. Differences between treatments were determined by a paired *t*-test. Significant results are indicated by * or ^$^ symbols [* or ^$^*p* < 0.05, ** or ^$$^*p* < 0.01, *** or ^$$$^*p* < 0.001 and **** or ^$$$$^*p* < 0.0001].

The anti-fibrotic actions of VPA were validated in primary CD patient-derived intestinal fibroblasts [Figure 4]. We confirmed VPA-mediated changes in both H3K27ac and Collagen-I by IF [Figure 4A and B]. TGFβ1|1 is upregulated in SCD compared to NSCD small bowel tissue [Supplementary Figure S5] and, as in CCD 18co cells, levels in primary CD intestinal fibroblasts are also inhibited by VPA [Figure 4A and B]. The effects of VPA were most pronounced in SCD compared to NSCD cultures despite both groups showing a similar increase in the positive control, H3K27ac [Figure 4B]. Therefore, under basal culture conditions, VPA significantly down-regulated both Collagen-I and TGFβ1|1 in SCD cultures only and not NSCD [Figure 4Bi–ii]. When primary cultures were stimulated with TGFβ both NSCD and SCD cultures showed increased expression of TGFβ1|1, which was inhibited by VPA [Figure 4Bi]. TGFβ1|1 knockdown inhibited TGFβ-induced Collagen-I up-regulation [Supplementary Figures S5 and S6]. However, TGFβ induced Collagen-I only in SCD cultures and again this increase was blocked by VPA [Figure 4Bii] suggesting differences in collagen regulation between NSCD and SCD cultures.

These results were consistent with qPCR data from SCD primary fibroblast cultures in which VPA suppressed *COL1A1*, *COL1A2*, *TGFβ1|1*, and *SMAD4* mRNAs levels under all conditions tested, but also increased MMP1 expression in control cells. In TGFβ-stimulated cultures VPA blocked the up-regulation of fibroblast activation markers, such as ACTA2 [Figure 4C]. Similar changes in mRNA expression were observed in CD mucosal biopsies cultured with or without VPA, with the most marked impact seen in biopsies cultured from CD patients with an SCD phenotype, compared to biopsies cultured from CD patients with an inflammatory phenotype [Figure 4D–G].

### 3.4. Suppression of Collagen-I by VPA is associated with reduced gene transcription at the COL1A1 and COL1A2 promoters mediated by reduced chromatin accessibility

**Figure 5.**
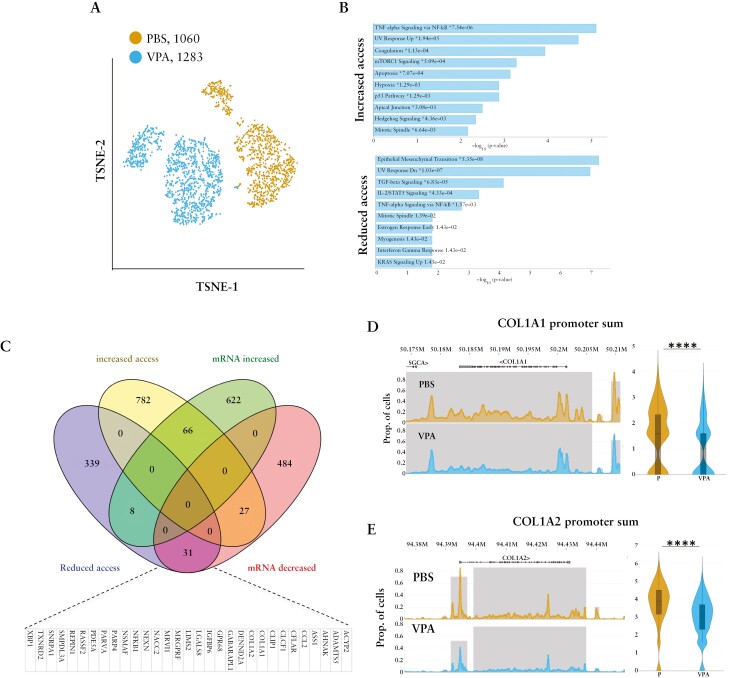
Significant epigenetic reprogramming of fibroblasts following VPA-mediated changes in chromatin accessibility at gene promoters. [A] sc-ATAC-seq of CCD-18Co cells treated with PBS [*n* = 1060] or VPA [*n* = 1283] as a 2D t-distributed stochastic neighbour embedding [TSNE] plot. [B] Pathway enrichment analysis of promoters with increased and reduced promoter accessibility in VPA-treated fibroblasts using the Molecular Signatures Database. [C] Overlap between changes in gene expression [illumina array] and promoter accessibility [sc-ATAC-seq] as a Venn diagram. Up-regulated genes and genes with increased promoter accessibility [top]; down-regulated genes and genes with reduced promoter accessibility [bottom]. Box indicates down-regulated genes with reduced promoter accessibility. [D, E] sc-ATAC-seq peak distribution charts showing the sizes of called peaks [grey rectangles] associated with the COL1A1 and COL1A2 promoters in CCD-18Co cells treated with PBS [*n* = 1060] or VPA [*n* = 1283]. Peak height is proportional to the number of cells within that cluster that had cut sites falling within the called peak. Cut site tracks are shown from cells treated with PBS [gold] and VPA [blue]; these are measures of average accessibility of a particular base for each cell in the cluster. Significant differences between VPA- and PBS-treated cells were calculated by comparing the sum of cut sites per cell [within peaks] which are close to one of the transcription start sites for each gene, respectively [promoter sums analysis]; log_2_ fold-change [FC] in VPA-treated cells and *p* values [****p < 0.0001] are shown.

Since our data indicate that suppression of Collagen-I by VPA is independent of its effects on SMAD signalling, alternative mechanisms of action were investigated using the CCD-18Co cell line and the scAssay for Transposase-Accessible Chromatin using sequencing [scATAC-seq] [Figure 5A]. VPA increased chromatin accessibility at 875 promoters [Supplementary Table S7], and genes associated with these promoters mapped to a diverse set of pathways in the Molecular Signatures Database [MsigDB] [Figure 5B; Supplementary Table S8]. VPA also decreased the promoter accessibility of 387 genes [Supplementary Table S7], which were significantly associated with five hallmark pathways in MsigDB [Supplementary Table S8], including the Epithelial Mesenchymal Transition [EMT], TGFβ Signalling and TNFα Signalling via NF-κB pathways.

Promoter accessibility data were then cross-compared with RNA gene expression array data to identify genes in which changes in promoter accessibility correlated with changes in transcription. Again, the gene expression array highlighted widespread changes in VPA-treated cells [Supplementary Table S9]. Overall, the gene expression array identified 766 up-regulated and 604 down-regulated genes [Supplementary Table S9]. Both up- and down-regulated genes were associated significantly with the EMT pathway in MsigDB [Supplementary Table S10]. Down-regulated genes were also linked to inflammatory pathways [Supplementary Table S10]. Expression of 66 of the up-regulated genes correlated with increased promoter accessibility, and 31 down-regulated genes, including *CO1A1* and *COL1A2*, were linked to reduced promoter accessibility [Figure 5C–E]. This supports the hypothesis that VPA effects on Collagen-I are mediated by its impact on chromatin remodelling. These data reveal that key profibrotic genes can be modulated through epigenetic regulation using a broad-acting HDAC inhibitor, opening the way for investigation of more specific HDAC inhibitors.

## 4. Discussion

HDACs are important in fibrosis in organs other than the CD intestine.^23–25^ H3K27ac levels have been used as a proxy for HDAC activity in a mouse model of colitis, and IBD patients with active disease.^13^ Moreover, histone acetylome studies using H3K27ac have identified chromatin-mediated gene expression changes during fibrosis in other organs.^26^ Here, we show that SCD tissue exhibited a reduced percentage of H3K27ac+ cells in the mucosa and *muscularis propria*, suggesting hypoacetylation is present in all SCD intestinal layers. Our paired study design, where stricture sections were compared with non-strictured sections from the same patient, controlled for confounding variables such as genotype, medication, gender, and age. Importantly, changes in H3K27ac in strictures are probably not driven by inflammation. This is consistent with our previous study of active IBD patients, which indicated that while inflammation drove H3K27ac hypoacetylation in the intestine of ulcerative colitis [UC] patients with active disease, this relationship was weaker in CD patients and not significant in sub-group analysis.^13^

Inhibiting Class I and Class IIa HDAC activity using VPA in *in vitro* models, CD patient cell cultures and mucosal biopsies increased H3K27ac levels, inhibited TGFβ signalling, and suppressed Collagen-I production, which was directly linked to reduced chromatin accessibility at collagen gene promoters. Importantly, our data highlight a novel epigenetic component to Collagen-I regulation in CD fibrosis and show that H3K27ac hypoacetylation is probably a feature of fibrosis in SCD. This is consistent with the analysis of RNA isolated from whole CD tissue and scRNA-seq analysis of fibroblasts isolated from SCD patients that showed higher levels of Class I and Class IIa HDAC enzymes relative to other fibroblast sub-types in the SCD small bowel. Our findings accord with reports of increased HDAC isoform expression, in fibroblasts in idiopathic pulmonary fibrosis,^14^ and in congestive heart failure.^27^

Consistent with a role for HDACs in fibroblast activation and fibrogenesis, we showed through endometabolome analysis of CCD-18Co fibroblasts and primary CD intestinal fibroblasts treated with VPA that HDAC activity correlated positively with changes in OHPro, a key component of collagen. Furthermore, VPA could suppress OHPro levels and inhibit any TGFβ1-induced increases. Interestingly, OHPro levels were elevated in primary SCD fibroblasts relative to NSCD-derived fibroblast cultures, consistent with increased HDAC expression and a pro-fibrotic phenotype. Since VPA can suppress this phenotype, it implies that SCD cultured fibroblasts retain a ‘memory’ of their stricture microenvironment linked to changes in their epigenome and the significant shift towards glycolysis in SCD- but not NSCD-derived fibroblasts. The clearest evidence of this is the changes in extracellular lactate secretion. For the primary cell cultures, extracellular glucose and lactate levels were uncorrelated, indicating that, overall, the cells are still deriving a large proportion of their energy from oxidative metabolism. Despite this, the shift towards a more glycolytic phenotype in the SCD cell cultures is very clear. The change in extracellular glucose is also interesting: for the SCD but not NSCD cultures, TGFβ treatment reduces the amount of glucose consumed, which we interpret as a switch away from growth and towards production of specialized compounds [e.g. collagen]. This effect is totally reversed by VPA and the metabolic data provide the clearest evidence that VPA can counteract the physiological effects of TGFβ treatment.

Here, we show that a metabolic shift occurs in the fibroblasts isolated from strictures in CD patients and distinguishes them from fibroblasts from non-strictured regions. This is an important addition to the literature since many studies/reviews rely on data from fibrosis in other tissues, or at best fibroblasts from inflamed tissue or animal models using chemically induced inflammation. We consider information from human tissue vital to elucidate the mechanisms and identify therapeutic interventions. An important further advance in therapeutic intervention would now be to understand the underlying reasons for the shift to glycolysis that occurs in the fibroblasts in CD strictures. Others have described multiple possible reasons for this shift in the metabolic signature in fibrosis.^5^ These may include: the consequences of hypoxia and the role of hypoxia inducible factor [HIF-1A]; increased glutaminolysis and reduced fatty acid oxidation; and the interaction between the Wnt/β-Catenin pathway and TGFβ. We have recently shown the importance of the cross-talk between Wnt and TGFβ signalling pathways in CD intestinal fibrosis.^17^ Dissecting the mechanisms driving the altered metabolic profile will be challenging and involve, for example, laser capture micro-dissection with downstream metabolomics/proteomics at the single-cell level. In addition, the mechanisms underlying ‘metabolic memory’ are considered to arise from environmental changes such as hypoxia and/or the influence of pathologies and inflammation.^28^ Interestingly, cardiac fibroblasts are thought to retain a metabolic memory of hypoxia. This has been linked to fibroblast activation, excessive collagen production, and cytokine secretion, through maintenance of a profibrotic environment. In pulmonary hypertension hypoxic conditions can cause a metabolic pyruvate to lactate shift.^29–31^ The mechanisms supporting such metabolic memory are diverse and include miRNAs, epigenetic modifications, cell–cell signalling, and cytokine signalling. Additional mechanisms may involve metabolites of the gut microbiome such as short-chain fatty acids (SCFAs). Butyrate is one of the most abundant SCFAs in the intestine and has marked effects on epigenetic mechanisms including histone acetylation and methylation.^32^ Future studies might involve the addition of butyrate to primary CD fibroblast cultures and evaluation of its effects on HDAC and H3K27ac expression and collagen production, and modification of the fibroblast response by VPA.

Our findings indicate that TGFβ suppresses SMAD3 RNA and protein while increasing SMAD3 phosphorylation. These observations align with existing literature, which suggests that SMAD3 down-regulation is cell type-specific, primarily occurring in response to TGF-β1 in mesenchymal cells rather than epithelial cells. This down-regulation involves protein degradation through the ubiquitin–proteasome pathway and the inhibition of gene transcription. Additionally, SMAD3 down-regulation has been documented in models of kidney fibrosis and may play a role in fine-tuning TGFβ signals.^33^ We confirmed that VPA-mediated inhibition of TGFβ1 signalling was marked by suppression of SMAD4 and identified changes in the TGFβ1 reporter gene, TGFβ1|1, which was linked to the extent of fibrosis in SCD patients. Interestingly, TGFβ1|1 has been implicated in TGFβ signalling, ECM regulation, stromal remodelling, and fibrosis in other organs.^1^ Our findings are consistent with studies demonstrating that VPA inhibits TGFβ signalling and fibrosis in other organs.^14,16^ However, we observed that VPA can inhibit Collagen-I expression independent of its effects on TGFβ signalling, implying additional mechanisms of action.

To explore other potential mechanisms, we performed scATAC-seq to evaluate VPA-induced changes in the chromatin landscape of intestinal fibroblasts in the absence on TGFβ1. This demonstrated widespread epigenetic remodelling of VPA-treated fibroblasts and highlighted reduced chromatin accessibility at promoters linked to activation of fibroblasts, including those for *COL1A1* and *COL1A2* whose mRNA levels were decreased. *NFKβ*, a pro-inflammatory mediator reported previously as a target of VPA in fibrosis,^34^ also showed reduced promoter accessibility and transcription. Overall, our data indicate that VPA suppression of Collagen-I production is mediated by its ability to inhibit HDACs and thereby modulate chromatin structure.

Interrogation of the MSigDB has identified diverse pathways one of which was the hallmark EMT pathway. The EMT pathway defines genes related to processes such as wound healing, fibrosis, and metastasis. While these events are typically associated with the EMT pathway, its identification can also signify fibroblast activation and increased pro-fibrotic processes depending on the context. In the case of CCD-18Co cells, which are already differentiated fibroblasts, the latter interpretation is more relevant. It is likely that sufficient genes in the EMT pathway have been upregulated that overlap with the other highlighted pathways [TGFβ signalling and TNFα signalling via NF-κB pathways].

In conclusion, using primary fibroblasts isolated from SCD and NSCD segments of the CD small bowel, we have identified distinct increases in HDAC expression with reduced H3K27ac levels in SCD. We demonstrate that VPA-induced increases in H3K27ac levels and changes in OHPro in CCD-18Co cells and primary SCD/NSCD cultures mirror changes in Collagen-I expression and secretion. Our results reveal a novel epigenetic component to Collagen-I regulation in CD fibrosis. In parallel, we report the novel discovery of a significant shift towards glycolysis in SCD-derived fibroblasts that is distinct from NSCD-derived fibroblasts. To our knowledge this is the first report of a difference in metabolism between NSCD and SCD fibroblasts *ex vivo*. Our findings suggest that HDAC inhibitor therapy may support ‘resetting’ the epigenetic changes associated with fibrosis.

## Supplementary Data

Supplementary data are available online at *ECCO-JCC* online.

jjad209_suppl_Supplementary_Tables_1

jjad209_suppl_Supplementary_Figures_1

jjad209_suppl_Supplementary_Tables_2

jjad209_suppl_Supplementary_Figures_2

jjad209_suppl_Supplementary_Tables_3

jjad209_suppl_Supplementary_Tables_4

jjad209_suppl_Supplementary_Tables_5

jjad209_suppl_Supplementary_Tables_6

jjad209_suppl_Supplementary_Figures_3

jjad209_suppl_Supplementary_Figures_4

jjad209_suppl_Supplementary_Figures_5

jjad209_suppl_Supplementary_Figures_6

jjad209_suppl_Supplementary_Tables_7

jjad209_suppl_Supplementary_Tables_8

jjad209_suppl_Supplementary_Tables_9

jjad209_suppl_Supplementary_Tables_10

## Data Availability

The data underlying the present article will be shared on request to the corresponding authors.
