# Supplementary material for: Epigenetic and Metabolic Reprogramming of Fibroblasts in Crohn’s Disease Strictures Reveals Histone Deacetylases as Therapeutic Targets
Source: J Crohns Colitis. 2023 Dec 9;18(6):895–907. doi: 10.1093/ecco-jcc/jjad209 (PMC11147807; doi:10.1093/ecco-jcc/jjad209)
Supplement: jjad209_suppl_Supplementary_Tables_1 [file jjad209_suppl_supplementary_tables_1.docx]

**Supplementary Table 1. Western blot antibody conditions**

| Gene ID | company | Species | Protein loaded | blocking buffer | Primary antibody dilution | Secondary antibody dilution | ECL |
| --- | --- | --- | --- | --- | --- | --- | --- |
| SMAD4 | Cell signalling 38454 | Rabbit | 30 μg | 5% BSA | 1/500 | 1/1000 | strong |
| SMAD7 | Santa cruz sc-365446 B-8 | Mouse | 30 μg | 5% MILK | 1/500 | 1/1000 | strong |
| SMAD7 | Invitrogen 701940 | Rabbit | 30 μg | 5% MILK | 1/300 | 1/1000 | strong |
| P-SMAD3 | Cell signalling 9520 | Rabbit | 10 μg | 5% BSA | 1/1000 | 1/2000 | normal |
| P-SMAD2 | Cell signalling 3108 | Rabbit | 30 μg | 5% BSA | 1/500 | 1/1000 | strong |
| TGFB1\|1 | BD transduction 611164 | Mouse | 10 μg | 5% BSA | 1/1000 | 1/2000 | normal |
| β-ACTIN | Abcam Ab8227 | Rabbit | 10 μg | 5% BSA | 1/1000 | 1/2000 | normal |
| β-ACTIN | Abgent AM1021B | Mouse | 10 μg | 5% BSA | 1/1000 | 1/2000 | normal |
