# Supplementary figures and images for: Epigenetic and Metabolic Reprogramming of Fibroblasts in Crohn’s Disease Strictures Reveals Histone Deacetylases as Therapeutic Targets
Source: J Crohns Colitis. 2023 Dec 9;18(6):895–907. doi: 10.1093/ecco-jcc/jjad209 (PMC11147807; doi:10.1093/ecco-jcc/jjad209)

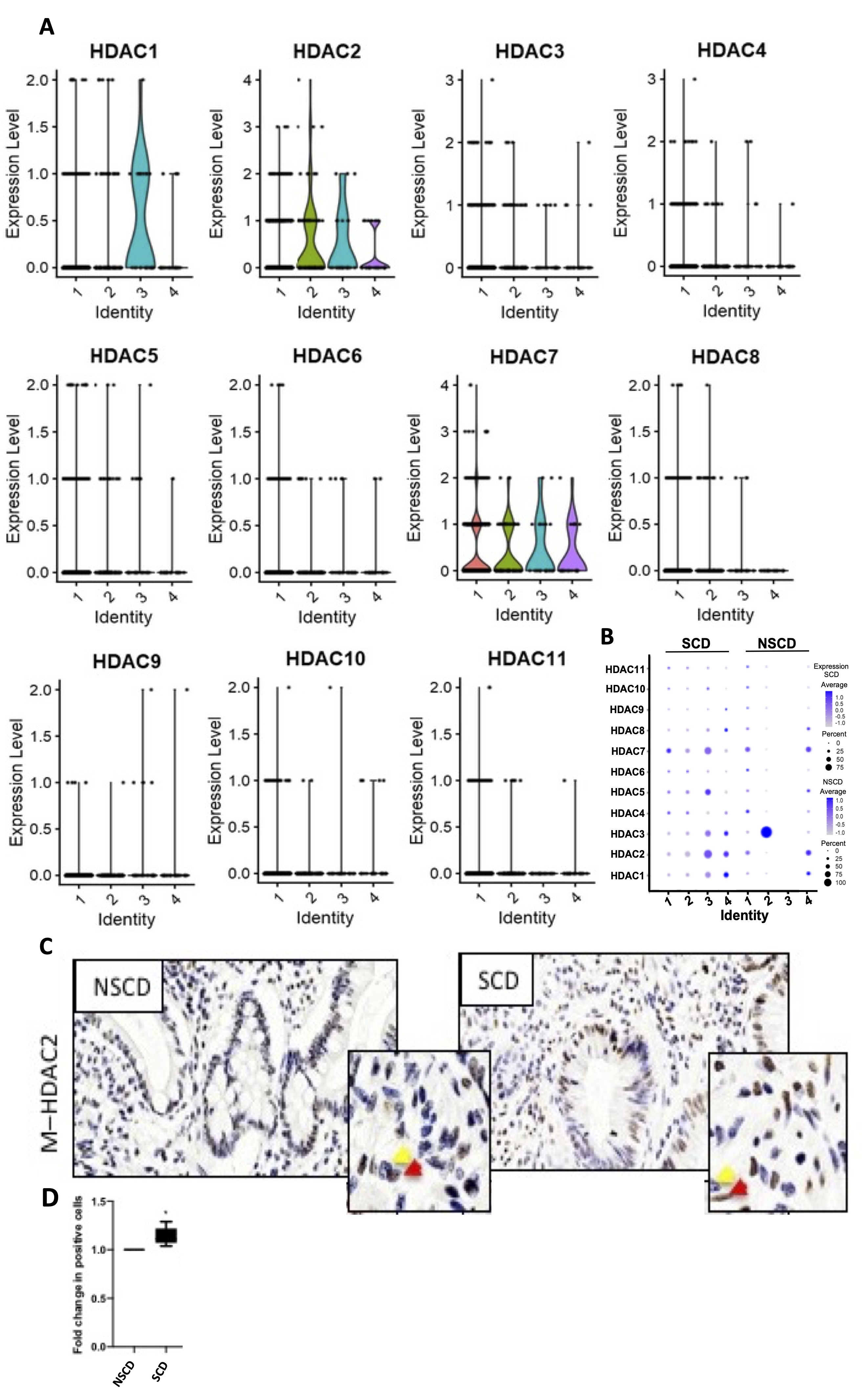

Supplement: jjad209_suppl_Supplementary_Figures_1 [file jjad209_suppl_supplementary_figures_1.jpeg]

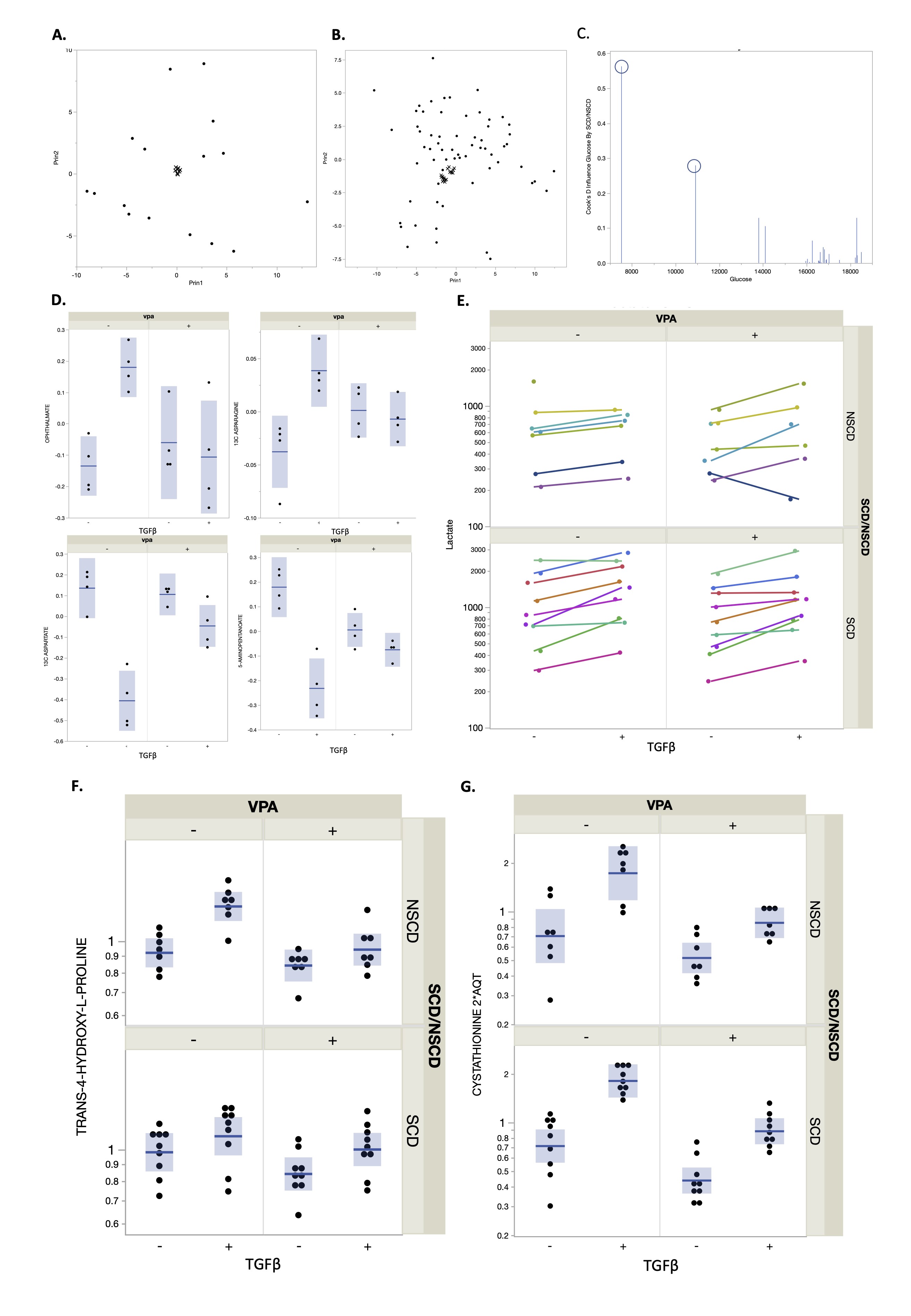

Supplement: jjad209_suppl_Supplementary_Figures_2 [file jjad209_suppl_supplementary_figures_2.jpeg]

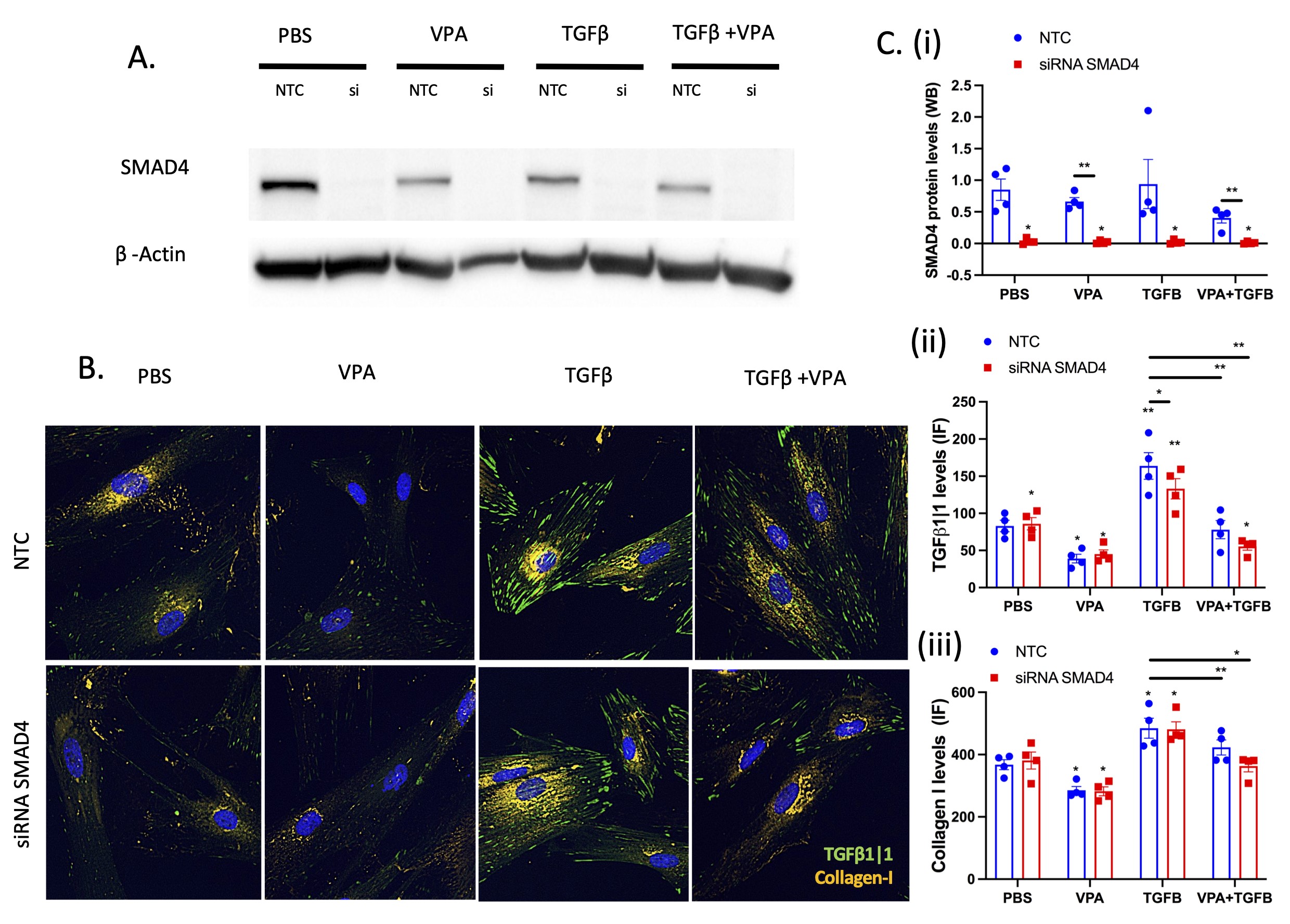

Supplement: jjad209_suppl_Supplementary_Figures_3 [file jjad209_suppl_supplementary_figures_3.jpeg]

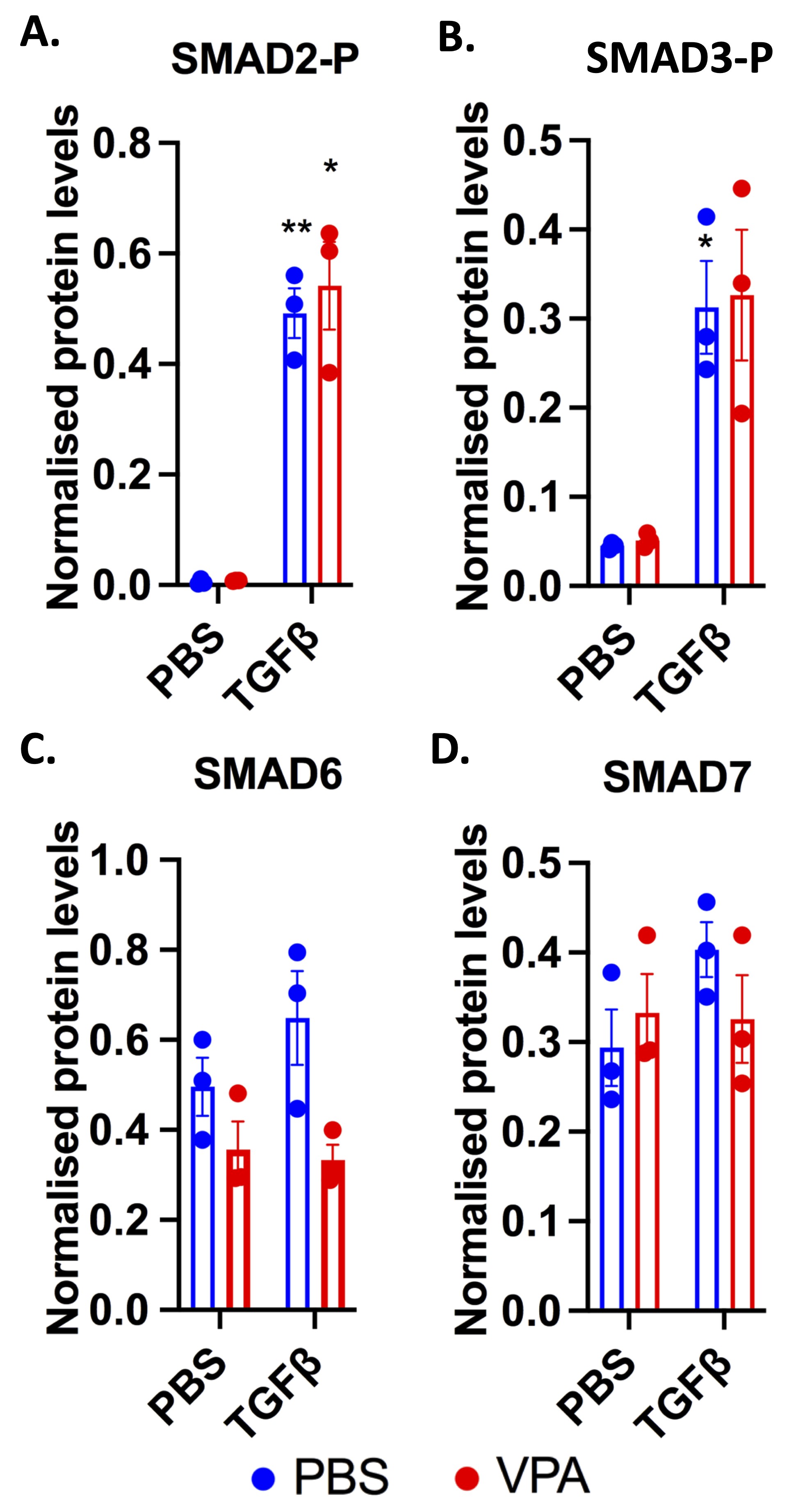

Supplement: jjad209_suppl_Supplementary_Figures_4 [file jjad209_suppl_supplementary_figures_4.jpeg]

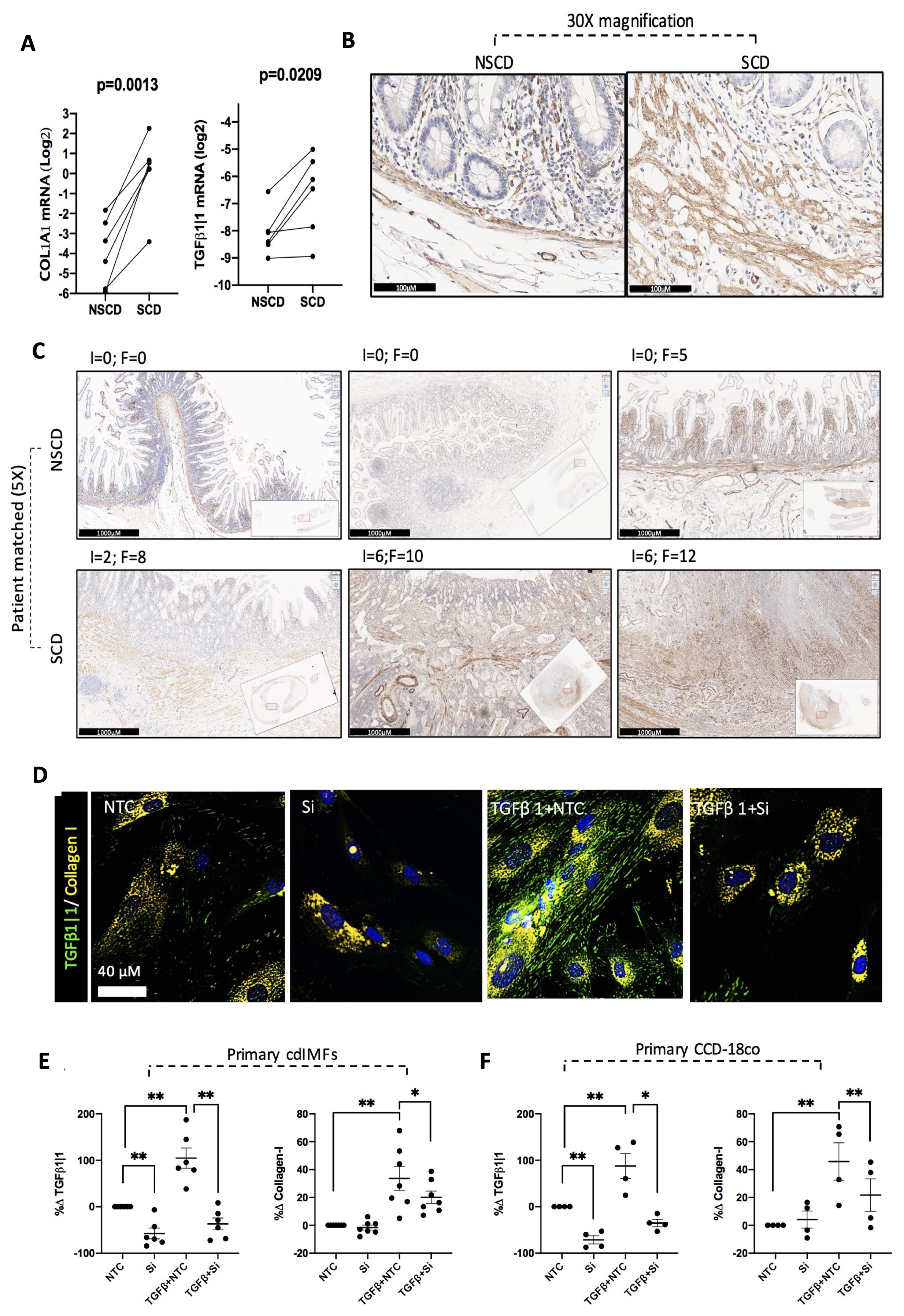

Supplement: jjad209_suppl_Supplementary_Figures_5 [file jjad209_suppl_supplementary_figures_5.jpeg]

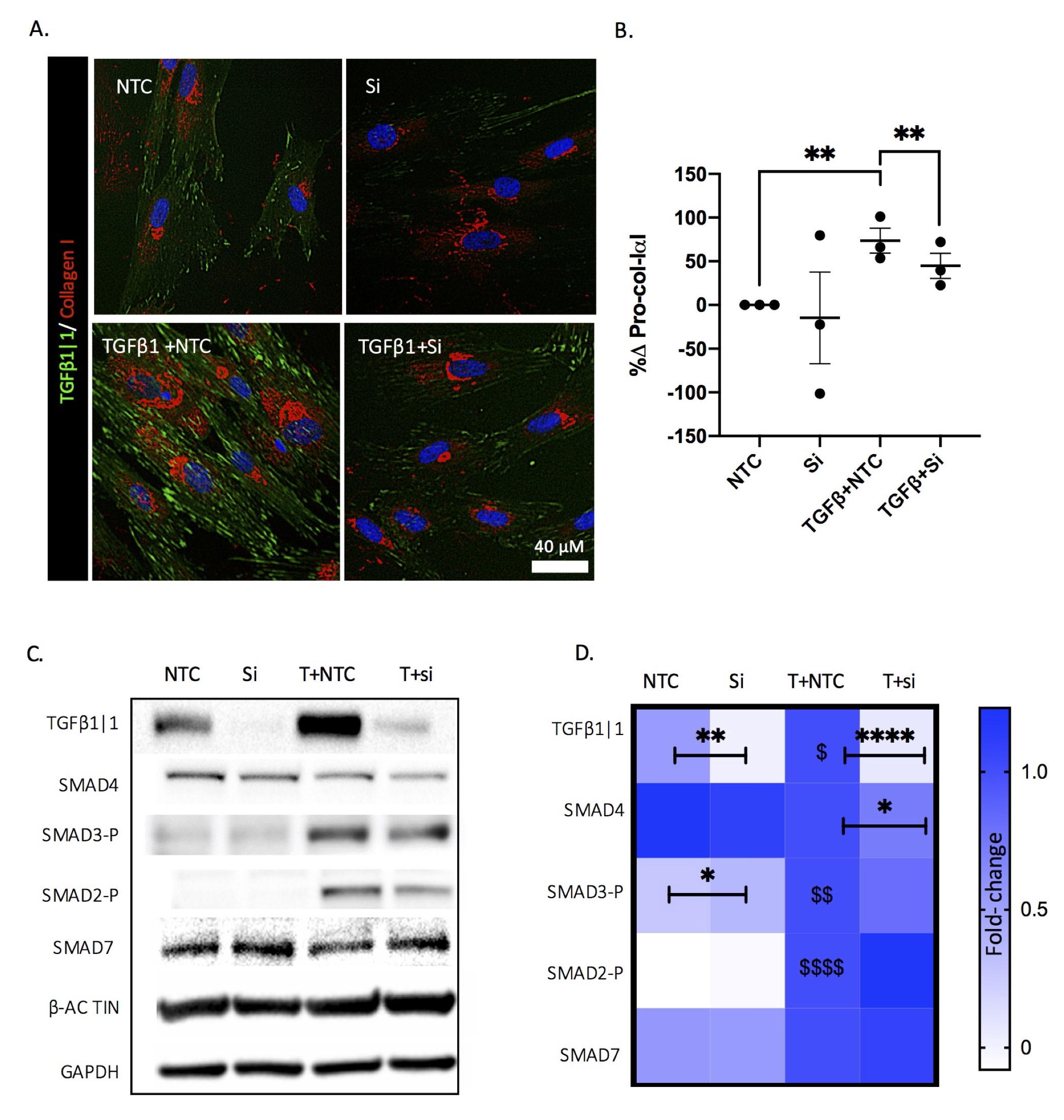

Supplement: jjad209_suppl_Supplementary_Figures_6 [file jjad209_suppl_supplementary_figures_6.jpeg]
