# Supplementary material for: Epigenetic and Metabolic Reprogramming of Fibroblasts in Crohn’s Disease Strictures Reveals Histone Deacetylases as Therapeutic Targets
Source: J Crohns Colitis. 2023 Dec 9;18(6):895–907. doi: 10.1093/ecco-jcc/jjad209 (PMC11147807; doi:10.1093/ecco-jcc/jjad209)
Supplement: jjad209_suppl_Supplementary_Tables_2 [file jjad209_suppl_supplementary_tables_2.docx]

**Supplementary Table 2: FFPE cohort characteristics**

| ID | Age | Gender | Age at diagnosis | Montreal | CRP | Active disease at sample collection? | Relevant medications | Blocks | Ulcer score | Fibrosis score |
| --- | --- | --- | --- | --- | --- | --- | --- | --- | --- | --- |
| CD1 | 36 | F | 23 | A2L1B2 | <5 | yes | adalimumab & methotrexate | NSCD | 0 | 9 |
|  |  |  |  |  |  |  |  | SCD | 5 | 11 |
| CD2 | 27 | F | 15 | A1L1B2&3 | 52 | yes | thiopurine | NSCD | 0 | 7 |
|  |  |  |  |  |  |  |  | SCD | 0 | 11 |
| CD3 | 45 | F | 28 | A2L3B2&3 | <5 | yes | none | NSCD | 2 | 6 |
|  |  |  |  |  |  |  |  | SCD | 0 | 9 |
| CD4 | 28 | F | 20 | A2L1B2 | <5 | no | none | NSCD | 0 | 6 |
|  |  |  |  |  |  |  |  | SCD | 0 | 10 |
| CD5 | 28 | M | 19 | A2L1B2 |  | yes | Exclusive enteral nutrition | NSCD | 3 | 7 |
|  |  |  |  |  |  |  |  | SCD | 0 | 11 |
| CD6 | x | M | 16 | A1L1L4B2 |  | yes | x | NSCD | 0 | 6 |
|  |  |  |  |  |  |  |  | SCD | 0 | 8 |
| CD7 | 30 | F | 18 | A2L3B2 | 40 | yes | adalimumab & 5mg pred | NSCD | 0 | 0 |
|  |  |  |  |  |  |  |  | SCD | 3 | 8 |
| CD8 | 51 | M | 28 | A2L1L4B2 | 18 | yes | adalimumab | NSCD | 0 | 0 |
|  |  |  |  |  |  |  |  | SCD | 6 | 10 |
| CD9 | 72 | F | 63 | A3L1L4B2 | 73 | yes | adalimumab | NSCD | 0 | 0 |
|  |  |  |  |  |  |  |  | SCD | 6 | 9.5 |
| CD10 | 52 | F | 35 | A2L1L4B2&3 | 12 | no | none | NSCD | 0 | 6 |
|  |  |  |  |  |  |  |  | SCD | 0 | 7 |
| CD11 | x |  |  |  |  |  |  | NSCD | 0 | 5 |
|  |  |  |  |  |  |  |  | SCD | 6 | 12 |
| CD12 | x |  |  |  |  |  |  | NSCD | 0 | 0 |
|  |  |  |  |  |  |  |  | SCD | 2 | 5 |
| CD13 | x |  |  |  |  |  |  | NSCD | 0 | 3 |
|  |  |  |  |  |  |  |  | SCD | 5.5 | 8 |
| CD14 | x |  |  |  |  |  |  | NSCD | 0 | 10 |
|  |  |  |  |  |  |  |  | SCD | 2 | 13 |

Abbreviations: CD, Crohns disease; CRP, C-reactive protein; NSCD, non-strictured and SCD, strictured; x, no data available. Differences in ulcer and fibrosis scores between NSCD and SCD tissues are shown in Figure 2.
