# Supplementary material for: Epigenetic and Metabolic Reprogramming of Fibroblasts in Crohn’s Disease Strictures Reveals Histone Deacetylases as Therapeutic Targets
Source: J Crohns Colitis. 2023 Dec 9;18(6):895–907. doi: 10.1093/ecco-jcc/jjad209 (PMC11147807; doi:10.1093/ecco-jcc/jjad209)
Supplement: jjad209_suppl_Supplementary_Tables_3 [file jjad209_suppl_supplementary_tables_3.docx]

**Supplementary Table 3. Metabolites identified following analysis of VPA and TGFβ treated CCD-18Co cultures.** CCD-18Co intestinal fibroblast cultures were treated with TGFβ or VPA alone or TGFβ and VPA. P-values (two-way ANOVA) are given for individual metabolites. More metabolites were significantly altered by TGFβ than by VPA (13 compared to 4 metabolites with P < 0.001). In the top three significantly affected by TGFβ was hydroxyproline (OHPro; P = 8.3e-7), a marker for collagen production.

| **Metabolite** | **TGFβ** | **VPA** | **TGFβ x VPA** |
| --- | --- | --- | --- |
| PROLINE | 1.89E-07 | 0.048793 | 0.218773 |
| O-PHOSPHOETHANOLAMINE | 6.62E-07 | 0.107647 | 0.143018 |
| TRANS-4-HYDROXY-L-PROLINE (OHPro) | 8.31E-07 | 0.741258 | 0.007322 |
| PUTRESCINE | 1.55E-06 | 0.004849 | 0.010341 |
| TAURINE | 2.42E-06 | 5.95E-05 | 0.025949 |
| HOMOCYSTINE | 1.43E-05 | 0.003419 | 0.001251 |
| ASPARTATE | 1.86E-05 | 0.007055 | 0.002378 |
| CYSTATHIONINE | 2.34E-05 | 0.05681 | 0.03951 |
| SDMA | 2.92E-05 | 0.022387 | 0.024132 |
| 5-AMINOPENTANOATE | 5.43E-05 | 0.836366 | 0.001494 |
| HYDROXYKYNURENINE | 7.06E-05 | 0.051477 | 0.120778 |
| 2-AMINOADIPATE | 0.000105 | 0.982219 | 0.301828 |
| HYPOTAURINE | 0.000196 | 0.004511 | 0.611943 |
| GLUTAMATE | 0.001072 | 0.174821 | 0.707798 |
| GLYCINE | 0.001265 | 0.944061 | 0.031358 |
| ALANINE | 0.001749 | 0.18157 | 0.518098 |
| N-METHYLASPARTATE | 0.002365 | 0.755343 | 0.13836 |
| GLUTAMINE | 0.00237 | 0.755699 | 0.138265 |
| TYRAMINE | 0.002947 | 0.22311 | 0.373105 |
| AGMATINE | 0.003529 | 0.878283 | 0.829415 |
| HOMOSERINE | 0.005654 | 0.266135 | 0.653347 |
| S-CARBOXYMETHYLCYSTEINE | 0.006591 | 0.053623 | 0.927943 |
| SARCOSINE | 0.00682 | 0.581659 | 0.19282 |
| CYSTINE | 0.008904 | 0.524319 | 0.087604 |
| CITRULLINE | 0.010893 | 0.274196 | 0.191691 |
| HISTIDINE | 0.013927 | 0.308276 | 0.441723 |
| CYSTEINE | 0.014438 | 0.017527 | 0.403584 |
| O-PHOSPHOSERINE | 0.01457 | 0.26851 | 0.274576 |
| ASPARAGINE | 0.016866 | 0.79228 | 0.004912 |
| CIS-4-HYDROXY-D-PROLINE | 0.017439 | 0.07951 | 0.509574 |
| 5-HYDROXYLYSINE | 0.020955 | 0.228094 | 0.430151 |
| METHIONINE | 0.023062 | 0.227859 | 0.22509 |
| CYSTEATE (possible overlap from 1- and 3-methylhistidine) | 0.025764 | 0.000218 | 0.177073 |
| TYROSINE | 0.026674 | 0.312785 | 0.438293 |
| GLUCOSAMINE 6-SULFATE (possible overlap from glucosamine phosphate) | 0.027875 | 0.538961 | 0.137842 |
| N,N,N-TRIMETHYLLYSINE | 0.033194 | 0.033092 | 0.590695 |
| N-METHYLGLUTAMATE | 0.037051 | 0.817492 | 0.725876 |
| TRYPTOPHAN | 0.038413 | 0.507021 | 0.23991 |
| OPHTHALMATE | 0.041493 | 0.097304 | 0.009788 |
| BETA-ALANINE | 0.042169 | 0.551619 | 0.800811 |
| KYNURENINE | 0.050625 | 0.491832 | 0.584701 |
| Ammonium | 0.064687 | 0.504913 | 0.791288 |
| C-Mannosyl Tryptophan | 0.082795 | 0.2565 | 0.674121 |
| DIETHANOLAMINE | 0.122092 | 0.303374 | 0.492942 |
| PHENYLALANINE | 0.140631 | 0.412525 | 0.309424 |
| ORNITHINE | 0.151269 | 0.025973 | 0.706057 |
| 3-AMINO-4-HYDROXYBENZOATE (possible overlap from 3-Amino-5-HydroxyBenzoate or Dopamine) | 0.193294 | 0.769416 | 0.194776 |
| ETHANOLAMINE | 0.193955 | 0.592465 | 0.475311 |
| GSH - GLUTATHIONE REDUCED | 0.197974 | 0.00846 | 0.498599 |
| ISOLEUCINE | 0.200041 | 0.13805 | 0.920249 |
| P-OCTOPAMINE | 0.207628 | 0.018946 | 0.54228 |
| LEUCINE | 0.27449 | 0.175064 | 0.98313 |
| METHIONINE SULFOXIMINE | 0.338971 | 0.805992 | 0.875894 |
| GSSG - GLUTATHIONE OXIDIZED | 0.344014 | 0.011778 | 0.612103 |
| SERINE | 0.347779 | 0.101211 | 0.343471 |
| 3-METHOXYTYRAMINE | 0.402958 | 0.3799 | 0.297459 |
| 2-AMINOBUTYRATE | 0.413972 | 0.506761 | 0.05437 |
| VALINE | 0.451583 | 0.178895 | 0.991577 |
| ALLOTHREONINE | 0.464643 | 5.58E-06 | 0.288204 |
| GABA | 0.664866 | 4.35E-05 | 0.014581 |
| ARGININE | 0.692605 | 0.244017 | 0.894223 |
| L-DOPA | 0.833227 | 0.082396 | 0.068554 |
| N1-ACETYLSPERMINE | 0.873959 | 0.347781 | 0.164291 |
| S-ADENOSYLMETHIONINE | 0.877479 | 0.165338 | 0.93072 |
| N-ALPHA-ACETYLLYSINE | 0.904009 | 0.178506 | 0.670332 |
| LYSINE | 0.920993 | 0.250367 | 0.823506 |
| THREONINE | 0.946603 | 0.655277 | 0.813267 |
