# Supplementary material for: Epigenetic and Metabolic Reprogramming of Fibroblasts in Crohn’s Disease Strictures Reveals Histone Deacetylases as Therapeutic Targets
Source: J Crohns Colitis. 2023 Dec 9;18(6):895–907. doi: 10.1093/ecco-jcc/jjad209 (PMC11147807; doi:10.1093/ecco-jcc/jjad209)
Supplement: jjad209_suppl_Supplementary_Tables_4 [file jjad209_suppl_supplementary_tables_4.docx]

**Supplementary Table 4. Primary CD fibroblasts and cohort characteristics**

| Sample ID | NSCD cultures (n=7) | SCD cultures  (n=9) | AGE | Gender | Age at diagnosis | Montreal | CRP | Active disease | Medications |
| --- | --- | --- | --- | --- | --- | --- | --- | --- | --- |
| 19RLH017 | 0 | 2 | 25 | M | 11 | A1L3L4B2p | 20 | yes | thiopurine |
| 19RLH016 | 0 | 3 | 28 | M | 12 | A1L3B2P | 37 | yes | none |
| 19RLH0019 | 0 | 2 | 35 | M | 26 | A2L1L4B2&3 | 18 | yes | adalimumab |
| 19RLH023 | 2 | 1 | 27 | M | 12 | A1L3B2p | 13 | yes | vedolizumab |
| 19RLH024 | 3 | 0 | 24 | F | 18 | A2L2L4B1 | 58 | yes | none |
| 19RLH026 | 0 | 1 | 26 | F | 16 | A1L3B2&3p | 61 | yes | adalimumab, thiopurine |
| 19RLH031 | 2 | 0 | 33 | M | 16 | A1L3B1p | 2 | No | adalimumab, thiopurine |

*No patients were taking steroids at the time of collection. Where indicated samples from several independent strictured / non strictured sections of the resection specimen were harvested and cell cultures derived.
