# Supplementary material for: Epigenetic and Metabolic Reprogramming of Fibroblasts in Crohn’s Disease Strictures Reveals Histone Deacetylases as Therapeutic Targets
Source: J Crohns Colitis. 2023 Dec 9;18(6):895–907. doi: 10.1093/ecco-jcc/jjad209 (PMC11147807; doi:10.1093/ecco-jcc/jjad209)
Supplement: jjad209_suppl_Supplementary_Tables_5 [file jjad209_suppl_supplementary_tables_5.docx]

**Supplementary table 5. Statistical comparison of intracellular metabolites.** Intracellular metabolites were measured in NSCD and SCD primary fibroblast cultures following treatment with VPA or TGFβ alone or VPA and TGFβ. P-values from analysis by linear model (ANOVA) identified significant differences in a number of metabolites in SCD fibroblasts compared to NSCD fibroblasts including hydroxyproline (OHPro), a marker for collagen production.

|  | SCD | TGFβ | VPA | TGFβ x VPA |
| --- | --- | --- | --- | --- |
| N-EPSILON-ACETYLLYSINE | 3.4664E-05 | 0.74413195 | 0.91370841 | 0.29633861 |
| SDMA | 5.2597E-05 | 0.12504898 | 0.77890092 | 0.67792232 |
| GLUCOSAMINATE | 0.00013795 | 0.51097813 | 0.73633117 | 0.91451073 |
| 4-HYDROXY-L-PHENYLGLYCINE | 0.00016461 | 0.0431438 | 0.91552758 | 0.75099692 |
| ASPARAGINE | 0.00018855 | 5.3908E-05 | 0.05111158 | 0.61985341 |
| ALANINE | 0.00020694 | 0.00611573 | 0.07949108 | 0.78532115 |
| HOMOCYSTINE | 0.00036723 | 0.38742889 | 0.47359316 | 0.90438112 |
| HYDROXYKYNURENINE | 0.00047889 | 0.41560992 | 0.33464105 | 0.42919896 |
| 2-AMINOADIPATE | 0.00066396 | 0.01754934 | 0.80166121 | 0.36078124 |
| METHIONINE SULFOXIMINE | 0.00079669 | 0.63525581 | 0.74238338 | 0.90375206 |
| AGMATINE | 0.00104872 | 0.08448727 | 0.46227874 | 0.54388317 |
| TRANS-4-HYDROXY-L-PROLINE (OHPro) | 0.00156644 | 0.02287697 | 0.09939878 | 0.71782717 |
| GABA | 0.00392159 | 0.71038835 | 0.00187096 | 0.53835823 |
| 2-AMINOPHENOL | 0.00557791 | 0.93416163 | 0.30590694 | 0.99335485 |
| HYPOTAURINE | 0.00710904 | 0.04308239 | 0.22743479 | 0.83307297 |
| C-MANNOSYL TRYPTOPHAN | 0.00807648 | 0.2650793 | 0.01595531 | 0.202471 |
| CYSTINE | 0.00850115 | 0.64157171 | 0.42038705 | 0.8108376 |
| 5-AMINOPENTANOATE | 0.00870701 | 0.99131746 | 0.06666303 | 0.36768183 |
| GSH GLUTATHIONE REDUCED | 0.00940073 | 0.28212397 | 0.97082672 | 0.7947972 |
| GSSG GLUTATHIONE OXIDIZED | 0.01431312 | 0.03413522 | 0.50698689 | 0.75025598 |
| METHYLTRYPTAMINE | 0.01802261 | 0.04349115 | 0.04441118 | 0.58377279 |
| ANSERINE | 0.0185162 | 0.22436456 | 0.66633709 | 0.82348681 |
| 3-METHOXYTYRAMINE | 0.01886984 | 0.55270801 | 0.66535768 | 0.87796019 |
| PROLINE | 0.02001528 | 0.1327426 | 0.19532399 | 0.95447902 |
| HOMOSERINE | 0.02004358 | 0.87490226 | 0.01456745 | 0.87898927 |
| 3-NITRO-L-TYROSINE | 0.0213881 | 0.44480491 | 0.66748807 | 0.56588519 |
| S-ADENOSYLHOMOCYSTEINE | 0.02185115 | 0.5332219 | 0.14912752 | 0.43051586 |
| EPINEPHRINE | 0.03137709 | 0.00389836 | 0.2302031 | 0.5615218 |
| CYSTEATE | 0.03311745 | 0.00051972 | 0.0097653 | 0.98113994 |
| L-DOPA | 0.03435386 | 0.29305261 | 0.71094401 | 0.3851654 |
| TAURINE | 0.05868115 | 0.07497956 | 0.43754914 | 0.83565249 |
| ASPARTATE | 0.06262274 | 0.15783838 | 2.0498E-05 | 0.89248063 |
| GLUTAMINE | 0.0705209 | 0.66864781 | 0.04628662 | 0.72715607 |
| OPHTHALMATE | 0.07454439 | 0.02109087 | 0.08255717 | 0.96241237 |
| AMINOISOBUTANOATE | 0.08186149 | 0.01700857 | 0.00144334 | 0.66253153 |
| 3-SULFINOALANINE | 0.08244677 | 0.01911527 | 3.5907E-07 | 0.11289255 |
| 2-AMINOISOBUTYRATE | 0.09247953 | 0.60468513 | 0.84828271 | 0.17889751 |
| KYNURENINE | 0.10353046 | 0.51161603 | 0.29708429 | 0.74465378 |
| ARGININE | 0.11843516 | 0.68996747 | 0.59701441 | 0.9351206 |
| N1-ACETYLSPERMINE | 0.12454047 | 0.25834446 | 0.71386369 | 0.77639336 |
| CARNOSINE | 0.12981293 | 0.73723731 | 0.04976468 | 0.42387487 |
| TYROSINE | 0.14780485 | 0.29986872 | 0.34381755 | 0.79890461 |
| 1-METHYL-L-HISTIDINE (possible overlap with 3-methylhistidine) | 0.17784183 | 0.03875657 | 2.4875E-07 | 0.13220928 |
| TRYPTOPHAN | 0.20054434 | 0.12207646 | 0.42255476 | 0.84119237 |
| NORADRENALINE | 0.20298251 | 0.01568454 | 0.69674669 | 0.83093649 |
| 5-AMINOLEVULINATE | 0.24300843 | 0.72768474 | 0.66087901 | 0.79375968 |
| LYSINE | 0.24625751 | 0.42341824 | 0.76670043 | 0.71251822 |
| CITRULLINE | 0.27754553 | 0.36258873 | 0.20631257 | 0.39517068 |
| ALLOTHREONINE | 0.28799303 | 0.8442667 | 0.52696551 | 0.70376289 |
| O-PHOSPHOSERINE | 0.29201114 | 0.17421488 | 0.0781499 | 0.42914983 |
| 3-AMINO-4-HYDROXYBENZOATE | 0.29360223 | 0.61309058 | 0.92440549 | 0.92324359 |
| CYSTATHIONINE | 0.31314997 | 0.00021246 | 0.0036057 | 0.09457832 |
| GLUTAMATE | 0.33622858 | 0.70389085 | 0.62562696 | 0.75106221 |
| SARCOSINE | 0.33764853 | 0.89966944 | 0.17698162 | 0.98831071 |
| cis-HYDROXY-L-PROLINE | 0.50763324 | 0.03887147 | 0.29817369 | 0.43280248 |
| AMMONIUM | 0.55217291 | 0.05974259 | 0.86987536 | 0.86899422 |
| DIETHANOLAMINE | 0.55982248 | 0.17323199 | 0.72997512 | 0.57511579 |
| N-ALPHA-ACETYLLYSINE | 0.64539881 | 0.82045716 | 0.51257643 | 0.48381641 |
| CDP-ETHANOLAMINE | 0.66425188 | 0.78088485 | 0.09987311 | 0.91339123 |
| TYRAMINE | 0.67783737 | 0.33778978 | 0.35862357 | 0.86036094 |
| S-CARBOXYMETHYLCYSTEINE | 0.68730559 | 0.51479125 | 0.18291989 | 0.6446567 |
| L-TRYPTOPHANAMIDE | 0.72554336 | 0.94495004 | 0.46989013 | 0.8637886 |
| ETHANOLAMINE | 0.73778562 | 0.10158406 | 0.67076518 | 0.57412798 |
| O-PHOSPHOETHANOLAMINE | 0.74104162 | 0.36581311 | 0.9570007 | 0.81521462 |
| METHIONINE | 0.85367708 | 0.57669397 | 0.59616563 | 0.90506565 |
| HISTIDINE | 0.89566941 | 0.47961744 | 0.17986035 | 0.8934333 |
| 3-AMINO-5-HYDROXYBENZOATE | 0.99073276 | 0.63218137 | 0.62555883 | 0.82465985 |
