# Supplementary material for: Epigenetic and Metabolic Reprogramming of Fibroblasts in Crohn’s Disease Strictures Reveals Histone Deacetylases as Therapeutic Targets
Source: J Crohns Colitis. 2023 Dec 9;18(6):895–907. doi: 10.1093/ecco-jcc/jjad209 (PMC11147807; doi:10.1093/ecco-jcc/jjad209)
Supplement: jjad209_suppl_Supplementary_Tables_6 [file jjad209_suppl_supplementary_tables_6.docx]

**Supplementary table 6. The effect of TGFβ and valproic acid (VPA) on the endometabolomic profiles of treated primary fibroblasts isolated from structuring (SCD) and non-stricturing (NSCD) Crohn’s disease intestine.** Primary fibroblast cultures were treated with TGFβ or VPA alone or TGFβ and VPA. P-values from ANOVA with NSCD and SCD data analysed separately. GABA was considered as a positive control unrelated to HDAC activity, which was highly significant in SCD and NSCD backgrounds.

| **metabolite** | **TGFβ (NSCD)** | **VPA (NSCD)** | **TGFβ x VPA (NSCD)** | **TGFβ (SCD)** | **VPA (SCD)** | **TGFβ x VPA (SCD)** |
| --- | --- | --- | --- | --- | --- | --- |
| AMINOISOBUTANOATE | 0.007988228 | 0.000118626 | 0.928859939 | 1.45307E-08 | 5.82943E-11 | 0.07567887 |
| CYSTATHIONINE | 8.60653E-05 | 0.00032679 | 0.022176198 | 6.30838E-10 | 4.00792E-08 | 0.001148134 |
| 1-METHYL-L-HISTIDINE (possible overlap from 3-methylhistidine) | 0.045926602 | 4.08891E-06 | 0.189250063 | 0.020362839 | 4.5671E-08 | 0.045926735 |
| GABA | 0.084766784 | 1.76368E-05 | 0.358730675 | 0.448257538 | 5.1398E-08 | 0.550435517 |
| 3-SULFINOALANINE | 0.01071418 | 5.00336E-06 | 0.161768994 | 0.015865985 | 5.30677E-08 | 0.054914355 |
| CYSTEATE | 0.003336577 | 0.017041748 | 0.469095668 | 2.23215E-08 | 5.94104E-07 | 0.11841708 |
| ASPARTATE | 0.719433417 | 3.9996E-05 | 0.950563988 | 0.012565417 | 1.362E-06 | 0.802720492 |
| O-PHOSPHOSERINE | 0.006439162 | 0.033397171 | 0.404253176 | 0.035154918 | 5.09978E-06 | 0.673418568 |
| 5-AMINOPENTANOATE | 0.359704124 | 0.001414159 | 0.056632608 | 0.773219033 | 0.000223209 | 0.003884645 |
| ASPARAGINE | 5.64885E-05 | 0.111056262 | 0.364092466 | 7.4087E-08 | 0.000254249 | 0.4952203 |
| HOMOSERINE | 0.084440287 | 0.008373507 | 0.278251303 | 0.793799434 | 0.000370098 | 0.449494792 |
| C-MANNOSYLTRYPTOPHAN | 0.191376604 | 0.008229513 | 0.601073249 | 0.094442464 | 0.001142424 | 0.037967935 |
| S-ADENOSYLHOMOCYSTEINE | 0.142905171 | 0.108447432 | 0.300661521 | 0.556207922 | 0.002419345 | 0.311071597 |
| CDP-ETHANOLAMINE | 0.839874506 | 0.006718417 | 0.470715129 | 0.306550653 | 0.003764901 | 0.967470744 |
| GLUTAMINE | 0.553459322 | 0.136747557 | 0.943845272 | 0.814297441 | 0.003776315 | 0.417904665 |
| HYPOTAURINE | 0.275657395 | 0.139543882 | 0.635087082 | 4.08371E-06 | 0.011911117 | 0.497848065 |
| ALANINE | 0.000428644 | 3.49372E-05 | 0.240951553 | 7.8629E-05 | 0.012218898 | 0.670842074 |
| SARCOSINE | 0.248896939 | 0.432768422 | 0.790371225 | 0.398491856 | 0.013624316 | 0.821861785 |
| OPHTHALMATE | 0.311931855 | 0.063203268 | 0.118107343 | 0.0007278 | 0.014862281 | 0.389456321 |
| KYNURENINE | 0.995607854 | 0.742609024 | 0.328336653 | 0.621013569 | 0.034330984 | 0.789210007 |
| TRANS-4-HYDROXY-L-PROLINE (OHPro) | 0.000108151 | 0.000403006 | 0.018929835 | 0.01683716 | 0.038934321 | 0.734061128 |
| METHYLTRYPTAMINE | 0.097199069 | 0.018512552 | 0.302282289 | 0.010090619 | 0.042771821 | 0.423667313 |
| ALLOTHREONINE | 0.851857674 | 0.395500346 | 0.138411977 | 0.475522527 | 0.050697211 | 0.512470186 |
| O-PHOSPHOETHANOLAMINE | 0.609653206 | 0.715204013 | 0.992676924 | 0.019892646 | 0.051001703 | 0.740504209 |
| TAURINE | 0.247241073 | 0.297187557 | 0.529605512 | 1.32515E-05 | 0.073715115 | 0.42901405 |
| PROLINE | 0.318000359 | 0.085318045 | 0.540987911 | 0.016955138 | 0.079518062 | 0.948837688 |
| CITRULLINE | 0.508870574 | 0.84707398 | 0.987945366 | 0.548061239 | 0.089006758 | 0.273236873 |
| TYROSINE | 0.452851774 | 0.759808767 | 0.544157016 | 0.133536285 | 0.093441975 | 0.647346356 |
| N1-ACETYLSPERMINE | 0.748993274 | 0.671587126 | 0.182870734 | 0.115071717 | 0.096642799 | 0.247152341 |
| HISTIDINE | 0.811080248 | 0.536998587 | 0.378570083 | 0.219670688 | 0.109098149 | 0.977423706 |
| 2-AMINOADIPATE | 0.000184375 | 0.695300834 | 0.081401432 | 1.25622E-08 | 0.119563147 | 0.005200656 |
| CARNOSINE | 0.977215193 | 0.051862276 | 0.844246967 | 0.473562691 | 0.161202515 | 0.100268942 |
| 5-AMINOLEVULINATE | 0.559413395 | 0.360665285 | 0.418885555 | 0.221467223 | 0.167548552 | 0.948889289 |
| GSH GLUTATHIONE REDUCED | 0.271689789 | 0.689127748 | 0.896670649 | 0.123453441 | 0.184744493 | 0.625123237 |
| cis-HYDROXY-L-PROLINE | 0.000935404 | 0.238983673 | 0.96377841 | 0.699957349 | 0.221077481 | 0.052939017 |
| LYSINE | 0.769417796 | 0.787123732 | 0.787602875 | 0.232415348 | 0.221392319 | 0.787655539 |
| S-CARBOXYMETHYLCYSTEINE | 0.747817982 | 0.049880522 | 0.337193199 | 0.093546775 | 0.235356008 | 0.558700865 |
| L-TRYPTOPHANAMIDE | 0.644734496 | 0.213136746 | 0.863723116 | 0.551628989 | 0.248838663 | 0.94899793 |
| HYDROXYKYNURENINE | 0.4420236 | 0.170406136 | 0.098073337 | 0.181992624 | 0.252082805 | 0.348674275 |
| GSSG GLUTATHIONE OXIDIZED | 0.084890774 | 0.174142635 | 0.644038652 | 0.003527894 | 0.28742334 | 0.996183146 |
| AGMATINE | 0.044512966 | 0.568045132 | 0.856881584 | 0.012840554 | 0.321164728 | 0.480248749 |
| TRYPTOPHAN | 0.074383167 | 0.480801127 | 0.821324148 | 0.159747742 | 0.324929137 | 0.401960468 |
| 3-AMINO-5-HYDROXYBENZOATE | 0.315613313 | 0.863215012 | 0.931756854 | 0.674931459 | 0.328361742 | 0.412319023 |
| ARGININE | 0.740371435 | 0.955306802 | 0.481899832 | 0.25930022 | 0.377733491 | 0.837726095 |
| N-ALPHA-ACETYLLYSINE | 0.484321023 | 0.583092429 | 0.137794552 | 0.940863096 | 0.390661725 | 0.041210885 |
| 2-AMINOPHENOL | 0.605303897 | 0.378746612 | 0.5243016 | 0.390651116 | 0.451311449 | 0.958859879 |
| 13C-METHIONINE | 0.039020966 | 0.435151707 | 0.556953637 | 0.822618228 | 0.456686296 | 0.288866547 |
| TYRAMINE | 0.038048299 | 0.290768041 | 0.211822665 | 0.810131999 | 0.514628644 | 0.497002236 |
| GLUTAMATE | 0.563670242 | 0.587648178 | 0.853335389 | 0.253911485 | 0.571012123 | 0.89681542 |
| NORADRENALINE | 0.263679575 | 0.075955446 | 0.91966249 | 0.016943063 | 0.609253086 | 0.479749064 |
| HOMOCYSTINE | 0.035461663 | 0.052724095 | 0.661623086 | 0.064648192 | 0.626553019 | 0.725739888 |
| 2-AMINOISOBUTYRATE | 0.739688734 | 0.725176361 | 0.244657107 | 0.416052265 | 0.638141235 | 0.605185426 |
| 3-METHOXYTYRAMINE | 0.934706198 | 0.951572229 | 0.36385565 | 0.0946879 | 0.714789742 | 0.575094819 |
| DIETHANOLAMINE | 0.44809685 | 0.714518286 | 0.157320716 | 0.088633536 | 0.725927391 | 0.297080725 |
| CYSTINE | 0.988089342 | 0.671447672 | 0.20883067 | 0.356770152 | 0.734509969 | 0.726366074 |
| EPINEPHRINE | 0.113057283 | 0.18087811 | 0.987408354 | 0.002408562 | 0.749613246 | 0.440697791 |
| 3-NITRO-L-TYROSINE | 0.961876206 | 0.465467163 | 0.371234536 | 0.158530764 | 0.780990933 | 0.403201592 |
| SDMA | 0.027763643 | 0.231899247 | 0.908354116 | 0.091131117 | 0.838834481 | 0.37623487 |
| ANSERINE | 0.278893615 | 0.966963391 | 0.357958559 | 0.24605656 | 0.84848189 | 0.783156625 |
| N-EPSILON-ACETYLLYSINE | 0.381959769 | 0.547548626 | 0.30439342 | 0.594287837 | 0.866535597 | 0.061396358 |
| GLUCOSAMINATE | 0.724513829 | 0.481015546 | 0.302552566 | 0.515063764 | 0.866798465 | 0.561529163 |
| 3-AMINO-4-HYDROXYBENZOATE | 0.5751085 | 0.767449916 | 0.474442152 | 0.208904186 | 0.872748958 | 0.350870469 |
| L-DOPA | 0.037155103 | 0.429115951 | 0.465327251 | 0.171234247 | 0.911526921 | 0.230632692 |
| ETHANOLAMINE | 0.006313984 | 0.221081548 | 0.687820614 | 0.672214543 | 0.953642013 | 0.399344451 |
| METHIONINE SULFOXIMINE | 0.941037852 | 0.871298101 | 0.459776288 | 0.565998032 | 0.978006568 | 0.699507362 |
| AMMONIUM | 0.168545183 | 0.353452532 | 0.686815111 | 0.054738123 | 0.992886415 | 0.852394553 |
