# Supplementary material for: Epigenetic and Metabolic Reprogramming of Fibroblasts in Crohn’s Disease Strictures Reveals Histone Deacetylases as Therapeutic Targets
Source: J Crohns Colitis. 2023 Dec 9;18(6):895–907. doi: 10.1093/ecco-jcc/jjad209 (PMC11147807; doi:10.1093/ecco-jcc/jjad209)
Supplement: jjad209_suppl_Supplementary_Tables_7 [file jjad209_suppl_supplementary_tables_7.docx]

| **Supplementary Table 7. Differential promoter sum analysis**  **(scAssay for Transposase-Accessible Chromatin using sequencing).**  CCD-18Co fibroblast were treated with PBS (untreated controls) or VPA. | | | | | | |  |
| --- | --- | --- | --- | --- | --- | --- | --- |
| Name | PBS Average | VPA Average | VPA Log2 Fold Change | VPA P-Value | |  |  |
| IRF8 | 1.30595748 | 0.29772426 | -2.1300435 | 2.5078E-37 |  |  |  |
| SERPINF1 | 1.27253566 | 0.6300489 | -1.0132315 | 2.3127E-11 |  |  |  |
| ELANE | 1.08871567 | 0.54797357 | -0.9893943 | 3.213E-11 |  |  |  |
| ATOH8 | 1.09623558 | 0.57935531 | -0.9190899 | 3.4326E-11 |  |  |  |
| BFSP1 | 1.50732392 | 0.7998322 | -0.9135245 | 6.6739E-09 |  |  |  |
| SYNC | 2.79907712 | 1.5087378 | -0.891226 | 1.4992E-11 |  |  |  |
| RASSF2 | 1.90170135 | 1.04444889 | -0.864027 | 2.4117E-10 |  |  |  |
| ZNF827 | 1.51651492 | 0.83765122 | -0.855703 | 2.8713E-07 |  |  |  |
| REPIN1 | 1.04443176 | 0.58257395 | -0.841323 | 5.7972E-07 |  |  |  |
| RP11-511P7.7 | 1.04443176 | 0.58257395 | -0.841323 | 5.7972E-07 |  |  |  |
| PKNOX2 | 2.73557567 | 1.53851022 | -0.8299521 | 5.4346E-11 |  |  |  |
| ITPRIP | 3.45080254 | 1.94647292 | -0.8257781 | 5.3613E-11 |  |  |  |
| IFIT3 | 1.1204664 | 0.63809551 | -0.8114679 | 3.3654E-07 |  |  |  |
| LRRC4 | 1.07367585 | 0.617979 | -0.7961293 | 0.00028419 |  |  |  |
| MAPKAPK2 | 1.45217792 | 0.8360419 | -0.7959685 | 6.4564E-08 |  |  |  |
| TLN2 | 1.34355702 | 0.77649705 | -0.7903633 | 3.6088E-08 |  |  |  |
| GPR137B | 1.69365055 | 0.98409937 | -0.7827482 | 6.6738E-09 |  |  |  |
| MRVI1 | 1.21822521 | 0.71131958 | -0.7755246 | 2.2658E-08 |  |  |  |
| SMAD9 | 1.29843757 | 0.76201317 | -0.7682497 | 3.4979E-08 |  |  |  |
| PAPPA | 1.48392865 | 0.88914947 | -0.7383864 | 1.3333E-06 |  |  |  |
| VEGFC | 1.57082537 | 0.94386637 | -0.7343616 | 2.1983E-06 |  |  |  |
| CLIP4 | 1.5841941 | 0.95191297 | -0.7343447 | 6.3203E-06 |  |  |  |
| VEZF1 | 2.05544171 | 1.235958 | -0.7334216 | 5.5119E-06 |  |  |  |
| AP003419.11 | 1.85825299 | 1.12571956 | -0.7226726 | 1.5335E-07 |  |  |  |
| CLCF1 | 1.85825299 | 1.12571956 | -0.7226726 | 1.5335E-07 |  |  |  |
| TMTC1 | 1.38032102 | 0.83765122 | -0.7200256 | 3.8508E-05 |  |  |  |
| RNF217 | 1.79892927 | 1.09916578 | -0.7102991 | 1.0877E-06 |  |  |  |
| KIAA1614 | 1.66774864 | 1.02030908 | -0.7084364 | 3.4027E-07 |  |  |  |
| SIRPA | 1.31849066 | 0.81270676 | -0.6975223 | 8.4562E-07 |  |  |  |
| EYA1 | 1.09456449 | 0.67510987 | -0.6965006 | 1.2015E-06 |  |  |  |
| LIMCH1 | 1.14803939 | 0.71775686 | -0.6769937 | 2.0463E-06 |  |  |  |
| C8orf86 | 1.0469384 | 0.66062599 | -0.6636212 | 5.6422E-06 |  |  |  |
| NACC2 | 2.25847924 | 1.4298811 | -0.6591339 | 8.2345E-07 |  |  |  |
| PTK7 | 1.69699273 | 1.07502597 | -0.6581951 | 1.0763E-06 |  |  |  |
| BOC | 1.72790791 | 1.09514248 | -0.6575011 | 8.931E-07 |  |  |  |
| LHFPL2 | 1.55160783 | 0.98409937 | -0.6564411 | 7.2897E-06 |  |  |  |
| SH2D4A | 1.31264184 | 0.83765122 | -0.6475401 | 4.7291E-06 |  |  |  |
| PLEKHA6 | 1.1279863 | 0.72178016 | -0.6435344 | 6.4639E-06 |  |  |  |
| NCOR2 | 1.34940584 | 0.86581433 | -0.6397031 | 0.00010289 |  |  |  |
| PHACTR2 | 1.14803939 | 0.73787336 | -0.6371597 | 0.00039607 |  |  |  |
| ASS1 | 2.15821379 | 1.38803877 | -0.6364677 | 2.2834E-06 |  |  |  |
| FAM65C | 1.19733657 | 0.77086443 | -0.6347364 | 1.2202E-05 |  |  |  |
| PLD1 | 2.00698008 | 1.29630751 | -0.630279 | 3.8009E-06 |  |  |  |
| GPR39 | 1.22240294 | 0.79017628 | -0.6289463 | 8.9888E-06 |  |  |  |
| FGF2 | 1.12130194 | 0.72580346 | -0.6269555 | 1.544E-05 |  |  |  |
| BNC2 | 2.04207298 | 1.32205664 | -0.6269183 | 7.4759E-05 |  |  |  |
| BNC1 | 1.08286685 | 0.70246832 | -0.6237674 | 0.01823083 |  |  |  |
| LIMS2 | 1.78305391 | 1.15710131 | -0.6234636 | 3.1922E-05 |  |  |  |
| C9orf91 | 1.07200476 | 0.69844502 | -0.6175112 | 7.7817E-05 |  |  |  |
| RHOBTB1 | 1.97690044 | 1.28906557 | -0.6165794 | 5.8677E-05 |  |  |  |
| SPRY1 | 1.51818601 | 0.99214597 | -0.6133039 | 0.00017837 |  |  |  |
| TGIF1 | 5.04836537 | 3.33209771 | -0.5992315 | 1.8503E-06 |  |  |  |
| ARHGAP22 | 1.17895457 | 0.77971569 | -0.5959776 | 8.0958E-05 |  |  |  |
| COL1A1 | 14.5819385 | 9.65753122 | -0.5943741 | 1.3354E-06 |  |  |  |
| PEA15 | 1.24663375 | 0.82558132 | -0.5940721 | 0.00256156 |  |  |  |
| IL1R1 | 1.92760326 | 1.27780033 | -0.5928182 | 9.4924E-06 |  |  |  |
| KAZN | 2.82665012 | 1.87405351 | -0.5926937 | 9.6925E-06 |  |  |  |
| COL1A2 | 2.59771068 | 1.7227774 | -0.5922502 | 2.2515E-05 |  |  |  |
| PXDN | 1.48894192 | 0.98812267 | -0.5911159 | 0.00021099 |  |  |  |
| HOXB3 | 2.11142325 | 1.40654596 | -0.5857603 | 4.332E-05 |  |  |  |
| TRHDE | 1.82483118 | 1.2158415 | -0.5854693 | 0.00014981 |  |  |  |
| CCDC7 | 1.00516113 | 0.67189123 | -0.5805547 | 7.2925E-05 |  |  |  |
| SH3RF1 | 1.82650227 | 1.22227878 | -0.579176 | 2.656E-05 |  |  |  |
| KLF4 | 2.8851383 | 1.93359836 | -0.5771253 | 7.117E-05 |  |  |  |
| MDFIC | 1.03356967 | 0.6928124 | -0.5765457 | 0.00125396 |  |  |  |
| GPR68 | 1.1739413 | 0.78695763 | -0.5765099 | 0.00017404 |  |  |  |
| PDGFA | 1.24412712 | 0.83523724 | -0.5744108 | 7.0117E-05 |  |  |  |
| RASA3 | 1.37781438 | 0.92777316 | -0.5701169 | 0.00010469 |  |  |  |
| AC037459.4 | 1.50648837 | 1.01467646 | -0.5697815 | 0.00011424 |  |  |  |
| ZNF106 | 2.54674241 | 1.72197274 | -0.5643455 | 2.1282E-05 |  |  |  |
| ACY1 | 1.40371629 | 0.95030365 | -0.5623838 | 0.00043827 |  |  |  |
| FAT1 | 2.32866506 | 1.57713391 | -0.5619351 | 0.00012866 |  |  |  |
| TJP2 | 1.96353171 | 1.33654052 | -0.5546483 | 0.00017791 |  |  |  |
| GYPC | 2.33451388 | 1.59322711 | -0.5509135 | 4.1048E-05 |  |  |  |
| PDLIM2 | 1.75965863 | 1.20135762 | -0.5503043 | 0.00011815 |  |  |  |
| DPP4 | 1.72540127 | 1.17802247 | -0.5502337 | 0.00018273 |  |  |  |
| TRAJ31 | 1.20736312 | 0.8279953 | -0.5437173 | 0.0002503 |  |  |  |
| STON1 | 1.05780049 | 0.72580346 | -0.5429127 | 0.00026892 |  |  |  |
| DENND5A | 1.16224367 | 0.79822288 | -0.5415877 | 0.00653121 |  |  |  |
| VGLL4 | 5.57058125 | 3.83581497 | -0.538164 | 2.3196E-05 |  |  |  |
| LGALS8 | 1.03022749 | 0.70971026 | -0.5371512 | 0.00023861 |  |  |  |
| KCTD15 | 1.23911384 | 0.85454909 | -0.5356436 | 0.0011861 |  |  |  |
| NEK6 | 1.30010866 | 0.89719608 | -0.534726 | 0.00027317 |  |  |  |
| ACTA2 | 1.59004292 | 1.09836112 | -0.5333701 | 0.00025653 |  |  |  |
| MDH2 | 2.08886353 | 1.44758362 | -0.5288023 | 9.0795E-05 |  |  |  |
| STYXL1 | 2.08886353 | 1.44758362 | -0.5288023 | 9.0795E-05 |  |  |  |
| MTA3 | 1.68278846 | 1.16917121 | -0.5250467 | 0.00043827 |  |  |  |
| STON1-GTF2A1L | 1.09122231 | 0.75959919 | -0.522167 | 0.00046293 |  |  |  |
| LATS1 | 1.20903421 | 0.84167453 | -0.5220943 | 0.0004508 |  |  |  |
| SECTM1 | 1.17477685 | 0.8199487 | -0.5183473 | 0.00058568 |  |  |  |
| CDH13 | 1.14469721 | 0.7998322 | -0.5167543 | 0.00157149 |  |  |  |
| SLC7A6 | 1.52487037 | 1.06617471 | -0.5159001 | 0.00049915 |  |  |  |
| ST5 | 5.36587262 | 3.7545443 | -0.5150459 | 6.7408E-05 |  |  |  |
| ECHDC1 | 1.00014786 | 0.70005434 | -0.5141774 | 0.00067198 |  |  |  |
| PDE4B | 1.22992284 | 0.86098637 | -0.5140931 | 0.00071636 |  |  |  |
| COA1 | 2.41806842 | 1.69783293 | -0.5099309 | 0.00018326 |  |  |  |
| ELAC1 | 1.06949813 | 0.75155259 | -0.5085263 | 0.00082158 |  |  |  |
| RP11-729L2.2 | 1.06949813 | 0.75155259 | -0.5085263 | 0.00082158 |  |  |  |
| FOXP1 | 3.79254062 | 2.66744842 | -0.5075424 | 9.9462E-05 |  |  |  |
| LARP6 | 1.0285564 | 0.72338948 | -0.5072995 | 0.0065186 |  |  |  |
| TXNRD2 | 3.03637202 | 2.13637272 | -0.5069943 | 0.00093573 |  |  |  |
| DENND2A | 5.21631 | 3.6716643 | -0.5064659 | 0.00038099 |  |  |  |
| GRSF1 | 1.02772085 | 0.72419414 | -0.5045259 | 0.00488289 |  |  |  |
| HHAT | 1.59672728 | 1.12571956 | -0.5039498 | 0.00343863 |  |  |  |
| SNTB1 | 1.10459103 | 0.77891103 | -0.5035386 | 0.01932337 |  |  |  |
| MAN2A2 | 1.47975092 | 1.04444889 | -0.5022713 | 0.00069487 |  |  |  |
| ADH5 | 1.22825175 | 0.86983763 | -0.4973909 | 0.0011447 |  |  |  |
| GLI3 | 2.40720633 | 1.70829352 | -0.4945807 | 0.00067134 |  |  |  |
| COBLL1 | 1.02354313 | 0.72741278 | -0.4922634 | 0.00118443 |  |  |  |
| NFASC | 1.01769431 | 0.72338948 | -0.4919953 | 0.00179006 |  |  |  |
| SMAD1 | 1.49061301 | 1.06054209 | -0.4907736 | 0.00181239 |  |  |  |
| OSBPL5 | 1.23075839 | 0.87627491 | -0.4897025 | 0.00142509 |  |  |  |
| PIEZO1 | 1.55160783 | 1.10560306 | -0.488613 | 0.00060321 |  |  |  |
| NFKB1 | 1.35525466 | 0.96720151 | -0.4863205 | 0.00079204 |  |  |  |
| MARVELD1 | 1.44716465 | 1.03318364 | -0.4857974 | 0.01708553 |  |  |  |
| SIX4 | 1.31180629 | 0.93823374 | -0.4831727 | 0.00080182 |  |  |  |
| GPD2 | 1.26167357 | 0.90443802 | -0.4798728 | 0.0009418 |  |  |  |
| SEMA4C | 1.75631645 | 1.25929315 | -0.4796588 | 0.00063069 |  |  |  |
| PRDM1 | 1.1923233 | 0.85615841 | -0.4774367 | 0.00132642 |  |  |  |
| MLXIP | 2.65035004 | 1.90463059 | -0.4764726 | 0.00032718 |  |  |  |
| ADAM33 | 1.99611799 | 1.43631838 | -0.4745727 | 0.000674 |  |  |  |
| ARL8A | 1.10709767 | 0.79741822 | -0.4729629 | 0.00304688 |  |  |  |
| ABLIM3 | 1.10542658 | 0.79661356 | -0.4722403 | 0.00243236 |  |  |  |
| TSPAN9 | 2.04374407 | 1.47413741 | -0.4711014 | 0.00083835 |  |  |  |
| SERTAD2 | 2.00865117 | 1.4499976 | -0.4699316 | 0.00954941 |  |  |  |
| SH2D7 | 1.15806594 | 0.8360419 | -0.4696784 | 0.00790903 |  |  |  |
| TBC1D2B | 1.15806594 | 0.8360419 | -0.4696784 | 0.00790903 |  |  |  |
| RXRA | 2.03037535 | 1.47011411 | -0.4655779 | 0.00102588 |  |  |  |
| ERBIN | 1.9743938 | 1.4298811 | -0.4652688 | 0.00069877 |  |  |  |
| MISP | 1.74963209 | 1.26733975 | -0.4649768 | 0.00169432 |  |  |  |
| INMT | 1.73960554 | 1.26090247 | -0.4640314 | 0.00130621 |  |  |  |
| INMT-FAM188B | 1.73960554 | 1.26090247 | -0.4640314 | 0.00130621 |  |  |  |
| CFLAR | 1.82232454 | 1.32125198 | -0.4636127 | 0.01622421 |  |  |  |
| CEP78 | 1.0928934 | 0.79259026 | -0.4630997 | 0.00945629 |  |  |  |
| CTD-2349B8.1 | 1.23577166 | 0.89719608 | -0.4615543 | 0.00424943 |  |  |  |
| ITPRIPL2 | 1.23577166 | 0.89719608 | -0.4615543 | 0.00424943 |  |  |  |
| DOCK10 | 1.48727083 | 1.07985393 | -0.4615223 | 0.00164963 |  |  |  |
| ZBTB1 | 2.84920985 | 2.07039059 | -0.4604768 | 0.00077786 |  |  |  |
| ZBTB25 | 2.84920985 | 2.07039059 | -0.4604768 | 0.00077786 |  |  |  |
| TBC1D20 | 1.15221712 | 0.83765122 | -0.4596071 | 0.04960512 |  |  |  |
| THRA | 1.09707112 | 0.79902754 | -0.4569416 | 0.00430108 |  |  |  |
| CRYAB | 1.09874221 | 0.80063686 | -0.456236 | 0.0030654 |  |  |  |
| UHRF1BP1L | 1.10041331 | 0.80224618 | -0.4555328 | 0.00799193 |  |  |  |
| HRCT1 | 1.47306656 | 1.07422131 | -0.4552247 | 0.00332092 |  |  |  |
| KLF3 | 2.06964598 | 1.50954246 | -0.4550417 | 0.00130621 |  |  |  |
| LAMC1 | 1.75548091 | 1.28182363 | -0.4534052 | 0.00401039 |  |  |  |
| PSME4 | 1.80394254 | 1.318838 | -0.4516296 | 0.0138131 |  |  |  |
| ANXA6 | 1.16224367 | 0.84972113 | -0.451478 | 0.00247971 |  |  |  |
| WIPF1 | 1.36945893 | 1.00260656 | -0.4495285 | 0.0043592 |  |  |  |
| FIGN | 1.46721774 | 1.07422131 | -0.4494883 | 0.00238186 |  |  |  |
| BIRC2 | 1.38616984 | 1.01548112 | -0.4486227 | 0.04523294 |  |  |  |
| PRICKLE2 | 2.4214106 | 1.77588497 | -0.4471093 | 0.00273818 |  |  |  |
| RASL11A | 2.61107941 | 1.91509118 | -0.4470443 | 0.00119656 |  |  |  |
| CLMP | 1.04860949 | 0.76925511 | -0.4465398 | 0.00956998 |  |  |  |
| CACNA2D1 | 1.33603711 | 0.98088073 | -0.4454849 | 0.00519013 |  |  |  |
| KIF13A | 1.14971048 | 0.84408851 | -0.4454333 | 0.00300001 |  |  |  |
| NEXN | 1.01435213 | 0.74511531 | -0.4446092 | 0.00343863 |  |  |  |
| GABPB1 | 1.93595872 | 1.42263916 | -0.4442406 | 0.0017081 |  |  |  |
| TRAF3IP2 | 1.82316009 | 1.33975916 | -0.4442178 | 0.00587114 |  |  |  |
| HOXA1 | 1.80561363 | 1.3268846 | -0.4441946 | 0.00376223 |  |  |  |
| CSGALNACT2 | 1.62513582 | 1.19492033 | -0.4433717 | 0.00263208 |  |  |  |
| ANXA2 | 1.69030836 | 1.24480926 | -0.4410979 | 0.01005801 |  |  |  |
| SPON2 | 2.83667667 | 2.08970243 | -0.4407291 | 0.00161337 |  |  |  |
| GREM1 | 1.20819866 | 0.88995413 | -0.4407038 | 0.00710515 |  |  |  |
| PTPN18 | 1.32601057 | 0.97766209 | -0.4393618 | 0.00301015 |  |  |  |
| ADAMTSL5 | 1.11628867 | 0.82316734 | -0.4390779 | 0.00578622 |  |  |  |
| MMP14 | 1.33436602 | 0.98490403 | -0.4377798 | 0.00311394 |  |  |  |
| CTIF | 2.26934133 | 1.67691177 | -0.4362613 | 0.00182904 |  |  |  |
| DCLK2 | 1.18814557 | 0.87788423 | -0.4362569 | 0.00684838 |  |  |  |
| SLC9A3R2 | 1.00265449 | 0.74109201 | -0.4356916 | 0.01228826 |  |  |  |
| ALDH1L2 | 1.18396785 | 0.87547025 | -0.4351477 | 0.00496091 |  |  |  |
| RASSF8 | 1.85323972 | 1.37114091 | -0.434432 | 0.00619328 |  |  |  |
| NRP1 | 1.57082537 | 1.16273393 | -0.4337264 | 0.00366363 |  |  |  |
| RAPGEF2 | 1.88248381 | 1.39447605 | -0.4326777 | 0.00254571 |  |  |  |
| ADGRA2 | 1.20736312 | 0.8947821 | -0.4319081 | 0.00427036 |  |  |  |
| MRPS35 | 1.40789402 | 1.04364422 | -0.4316083 | 0.041271 |  |  |  |
| COL6A1 | 1.33269493 | 0.98812267 | -0.4312699 | 0.00408941 |  |  |  |
| C5orf42 | 1.23911384 | 0.9189219 | -0.4309598 | 0.00442293 |  |  |  |
| SSFA2 | 1.42376938 | 1.05651879 | -0.4301006 | 0.00306393 |  |  |  |
| CHST3 | 1.02688531 | 0.76201317 | -0.4299935 | 0.02997604 |  |  |  |
| ROCK2 | 2.21586643 | 1.64472536 | -0.4298179 | 0.00971717 |  |  |  |
| CBFB | 1.04610285 | 0.77649705 | -0.4295854 | 0.00484081 |  |  |  |
| RIOK3 | 1.4020452 | 1.04123024 | -0.4289442 | 0.00431173 |  |  |  |
| ANXA11 | 1.19065221 | 0.88432151 | -0.4287646 | 0.0043592 |  |  |  |
| PDE10A | 1.89585254 | 1.40895994 | -0.4279836 | 0.00483917 |  |  |  |
| PRKACB | 1.1831323 | 0.87949355 | -0.4275211 | 0.00457956 |  |  |  |
| WNT5A | 2.68711404 | 1.99797117 | -0.4273449 | 0.00222587 |  |  |  |
| RAP1B | 1.37113002 | 1.01950442 | -0.4271935 | 0.00355742 |  |  |  |
| RNF2 | 1.00599667 | 0.74833395 | -0.4264742 | 0.00637779 |  |  |  |
| RB1 | 1.30595748 | 0.97202947 | -0.4257204 | 0.01410467 |  |  |  |
| TRIM66 | 2.07131707 | 1.54253352 | -0.4250315 | 0.00230694 |  |  |  |
| CRYBG3 | 1.26919348 | 0.94628035 | -0.4232511 | 0.00465177 |  |  |  |
| SASH1 | 1.39536084 | 1.04042558 | -0.4231682 | 0.00447476 |  |  |  |
| XPOT | 2.22756406 | 1.66162323 | -0.4226717 | 0.00283614 |  |  |  |
| PRKCA | 1.0377474 | 0.77488773 | -0.4210154 | 0.01214331 |  |  |  |
| PLCB1 | 1.51150165 | 1.1289382 | -0.4207409 | 0.00423186 |  |  |  |
| WWC2 | 1.99695353 | 1.49264459 | -0.4197115 | 0.0029463 |  |  |  |
| PCNX1 | 1.41624947 | 1.05973743 | -0.4180799 | 0.00649102 |  |  |  |
| EFS | 1.22323848 | 0.91570326 | -0.417427 | 0.0065936 |  |  |  |
| NAV2 | 2.43645042 | 1.82416458 | -0.4173589 | 0.00814015 |  |  |  |
| FBLN5 | 1.90337244 | 1.42505314 | -0.4173164 | 0.01083758 |  |  |  |
| SEC62 | 1.03440522 | 0.77488773 | -0.4163653 | 0.01159461 |  |  |  |
| TRAM2 | 1.67276191 | 1.25366053 | -0.4158356 | 0.00651392 |  |  |  |
| UNC5B | 1.4480002 | 1.08548655 | -0.4154386 | 0.00542859 |  |  |  |
| NSMAF | 1.28673993 | 0.96559219 | -0.4139248 | 0.01258353 |  |  |  |
| JDP2 | 1.67276191 | 1.25687917 | -0.4121388 | 0.00454153 |  |  |  |
| IGFBP6 | 1.5198571 | 1.14261742 | -0.411326 | 0.00830705 |  |  |  |
| SPTBN1 | 2.58851968 | 1.94647292 | -0.4110898 | 0.00349711 |  |  |  |
| PARVA | 1.51400828 | 1.13859412 | -0.4108518 | 0.00467927 |  |  |  |
| HDAC7 | 1.80728472 | 1.35987567 | -0.410118 | 0.00486219 |  |  |  |
| MAST4 | 2.95114639 | 2.22086204 | -0.409997 | 0.00475369 |  |  |  |
| DDR1 | 1.64101119 | 1.235958 | -0.4087063 | 0.00622027 |  |  |  |
| TACC2 | 2.81578803 | 2.12108418 | -0.4085732 | 0.00525371 |  |  |  |
| CAPZA2 | 1.27170011 | 0.95835025 | -0.4078258 | 0.00606135 |  |  |  |
| PRKG1 | 2.49660969 | 1.88210011 | -0.4074485 | 0.00357897 |  |  |  |
| ANKRD33B | 1.77469845 | 1.3389545 | -0.4062348 | 0.01764743 |  |  |  |
| ACTG2 | 1.16558585 | 0.87949355 | -0.4059803 | 0.02069512 |  |  |  |
| PIM1 | 4.4233774 | 3.338535 | -0.4058135 | 0.00350017 |  |  |  |
| RP11-463D19.2 | 1.54241683 | 1.16434325 | -0.4054164 | 0.00747926 |  |  |  |
| UBE2W | 1.54241683 | 1.16434325 | -0.4054164 | 0.00747926 |  |  |  |
| C1orf21 | 1.44215138 | 1.08870519 | -0.4053345 | 0.007538 |  |  |  |
| RHBDD1 | 1.07868912 | 0.81431608 | -0.405266 | 0.00897151 |  |  |  |
| TPBG | 1.39452529 | 1.05410481 | -0.4034745 | 0.00633156 |  |  |  |
| GGT5 | 1.73125009 | 1.30918208 | -0.4029135 | 0.0073974 |  |  |  |
| BMPR1A | 2.08886353 | 1.58035255 | -0.4022699 | 0.01942394 |  |  |  |
| PPP3CC | 1.47139547 | 1.11606364 | -0.3985005 | 0.00751275 |  |  |  |
| CUL1 | 1.50565283 | 1.14261742 | -0.397787 | 0.00723444 |  |  |  |
| ACTR10 | 1.14302612 | 0.86742365 | -0.3977217 | 0.01014628 |  |  |  |
| XBP1 | 1.90922126 | 1.44919294 | -0.3975205 | 0.01180432 |  |  |  |
| ETV6 | 1.46972438 | 1.11606364 | -0.396862 | 0.00726734 |  |  |  |
| P4HA2 | 1.58920737 | 1.20699024 | -0.3966457 | 0.00762039 |  |  |  |
| NFATC2 | 1.39953856 | 1.06295607 | -0.3965941 | 0.00830705 |  |  |  |
| TNXB | 3.47085563 | 2.63687134 | -0.3963267 | 0.00829518 |  |  |  |
| FAF2 | 1.68780173 | 1.28262829 | -0.395807 | 0.00705309 |  |  |  |
| METRNL | 2.14150289 | 1.62863216 | -0.3947685 | 0.00831331 |  |  |  |
| PHF10 | 1.3928542 | 1.05973743 | -0.394063 | 0.01072741 |  |  |  |
| STK24 | 1.29509539 | 0.98651335 | -0.3923576 | 0.01507357 |  |  |  |
| PARP4 | 1.24914039 | 0.95271763 | -0.3905172 | 0.04523115 |  |  |  |
| C1orf198 | 2.15320052 | 1.6439207 | -0.3891512 | 0.0083497 |  |  |  |
| LRRN4CL | 1.1831323 | 0.90363336 | -0.3884918 | 0.0296702 |  |  |  |
| CCL2 | 1.58837182 | 1.21342752 | -0.3882186 | 0.00800145 |  |  |  |
| VDAC1 | 2.0203488 | 1.54414284 | -0.3875986 | 0.0084349 |  |  |  |
| RAP1GDS1 | 1.84321317 | 1.4097646 | -0.3865547 | 0.00947989 |  |  |  |
| DENND5B | 1.19148775 | 0.91167996 | -0.3858588 | 0.03283719 |  |  |  |
| BMP2K | 1.22992284 | 0.94225705 | -0.384079 | 0.01145331 |  |  |  |
| LIFR | 1.36945893 | 1.04927685 | -0.3839401 | 0.01042774 |  |  |  |
| MSL1 | 1.40371629 | 1.07583063 | -0.3835357 | 0.01180217 |  |  |  |
| CDH11 | 2.42976605 | 1.86520225 | -0.3813146 | 0.00731074 |  |  |  |
| AHNAK | 4.11506115 | 3.15990044 | -0.3809158 | 0.00629073 |  |  |  |
| MSRB3 | 1.00933886 | 0.77569239 | -0.3795075 | 0.01745184 |  |  |  |
| P4HTM | 1.49061301 | 1.14664073 | -0.3782442 | 0.02712976 |  |  |  |
| MAP4K4 | 1.37113002 | 1.05490947 | -0.3779805 | 0.01165205 |  |  |  |
| KLHL42 | 1.1555593 | 0.88914947 | -0.3777867 | 0.01506903 |  |  |  |
| ADAMTS5 | 2.81411694 | 2.16614515 | -0.3774003 | 0.00885473 |  |  |  |
| FOSL2 | 8.50752337 | 6.54912903 | -0.3773561 | 0.00442341 |  |  |  |
| HIPK1 | 1.77386291 | 1.36631295 | -0.376392 | 0.01002524 |  |  |  |
| MDGA1 | 1.84404872 | 1.4218345 | -0.374916 | 0.02352616 |  |  |  |
| CLIP1 | 1.13132849 | 0.87305627 | -0.3735629 | 0.01894209 |  |  |  |
| DPH6 | 1.11545312 | 0.86098637 | -0.3732556 | 0.02348758 |  |  |  |
| QKI | 2.33284279 | 1.80082943 | -0.3732552 | 0.00906274 |  |  |  |
| FAM69A | 1.4112362 | 1.09111917 | -0.3708966 | 0.01668292 |  |  |  |
| SMAD7 | 2.59186186 | 2.00521311 | -0.3700749 | 0.01028815 |  |  |  |
| PCBP1 | 1.49144856 | 1.15388267 | -0.3699755 | 0.01433233 |  |  |  |
| PAMR1 | 1.45886229 | 1.1289382 | -0.3696308 | 0.01450893 |  |  |  |
| CDC42BPA | 1.70033491 | 1.31642402 | -0.3689774 | 0.01258329 |  |  |  |
| ABHD14B | 2.72805576 | 2.11303757 | -0.3684027 | 0.01279467 |  |  |  |
| MARCH6 | 1.40789402 | 1.09192383 | -0.3664152 | 0.01765005 |  |  |  |
| PRDM5 | 1.11211094 | 0.86259569 | -0.3662381 | 0.03321286 |  |  |  |
| CTNNA1 | 1.42711156 | 1.10721238 | -0.365918 | 0.01584335 |  |  |  |
| PIEZO2 | 1.05780049 | 0.82075336 | -0.3657278 | 0.02150015 |  |  |  |
| SPATS2L | 1.4020452 | 1.08870519 | -0.3646686 | 0.02909982 |  |  |  |
| VIM | 5.27981145 | 4.10054817 | -0.3645703 | 0.01257265 |  |  |  |
| TMEM254 | 1.13132849 | 0.87868889 | -0.3642936 | 0.03864556 |  |  |  |
| ZC3H12C | 1.58586519 | 1.2319347 | -0.3641177 | 0.0407112 |  |  |  |
| ILK | 1.33937929 | 1.04123024 | -0.3630162 | 0.02904709 |  |  |  |
| RRP8 | 1.33937929 | 1.04123024 | -0.3630162 | 0.02904709 |  |  |  |
| IGF2BP2 | 2.12479198 | 1.65277196 | -0.3622544 | 0.01370866 |  |  |  |
| EMP1 | 1.99946017 | 1.55540809 | -0.3621295 | 0.0147309 |  |  |  |
| FBLIM1 | 1.54575901 | 1.20296694 | -0.3614888 | 0.02989238 |  |  |  |
| SVEP1 | 1.36862338 | 1.06537005 | -0.3611176 | 0.02647535 |  |  |  |
| ACYP2 | 2.69045622 | 2.09855369 | -0.3583058 | 0.02648073 |  |  |  |
| OMA1 | 1.5933851 | 1.24319994 | -0.357815 | 0.01889303 |  |  |  |
| SNX33 | 1.70869036 | 1.33332188 | -0.3576563 | 0.01663938 |  |  |  |
| TLK1 | 1.10124885 | 0.85937705 | -0.3574768 | 0.02669601 |  |  |  |
| TRERF1 | 2.47070778 | 1.9287704 | -0.3570845 | 0.01456973 |  |  |  |
| OSBPL10 | 1.17895457 | 0.92053122 | -0.3566866 | 0.04991932 |  |  |  |
| ZNF860 | 1.17895457 | 0.92053122 | -0.3566866 | 0.04991932 |  |  |  |
| RUNX2 | 1.15305267 | 0.90041472 | -0.3565087 | 0.02645019 |  |  |  |
| NPLOC4 | 2.08635689 | 1.62943682 | -0.3564334 | 0.01414994 |  |  |  |
| CACNB3 | 4.86538092 | 3.80362857 | -0.3550744 | 0.01053833 |  |  |  |
| GPR20 | 1.38199211 | 1.08065859 | -0.3545922 | 0.03295079 |  |  |  |
| GSAP | 1.15890148 | 0.906852 | -0.3535346 | 0.03868941 |  |  |  |
| DEGS1 | 1.17979012 | 0.92374986 | -0.3526768 | 0.02515034 |  |  |  |
| FAM175A | 1.57834528 | 1.235958 | -0.3525632 | 0.01747149 |  |  |  |
| CAMK2D | 2.14568061 | 1.68093507 | -0.3519981 | 0.01573442 |  |  |  |
| RGS20 | 1.76717854 | 1.38642945 | -0.349874 | 0.02166293 |  |  |  |
| DR1 | 1.17811903 | 0.92455452 | -0.3493782 | 0.03219181 |  |  |  |
| SMAD3 | 3.77666525 | 2.96436802 | -0.3492714 | 0.01418016 |  |  |  |
| YAP1 | 1.94932744 | 1.53046362 | -0.3488231 | 0.01737582 |  |  |  |
| DPYSL3 | 2.37963333 | 1.86922555 | -0.3481397 | 0.02240207 |  |  |  |
| ACAA2 | 1.44883574 | 1.13859412 | -0.3474085 | 0.03494829 |  |  |  |
| MAGI2 | 2.57682205 | 2.02532962 | -0.3472863 | 0.01503922 |  |  |  |
| UPP2 | 2.19414225 | 1.72519138 | -0.3467327 | 0.02899668 |  |  |  |
| SMPDL3A | 1.49061301 | 1.17238985 | -0.3462275 | 0.03200515 |  |  |  |
| TSHZ3 | 2.16489816 | 1.70346555 | -0.3456572 | 0.01930562 |  |  |  |
| PPHLN1 | 1.9117279 | 1.5047145 | -0.345202 | 0.02987956 |  |  |  |
| TRIO | 2.57765759 | 2.03015758 | -0.3443203 | 0.01628316 |  |  |  |
| COG4 | 1.05445831 | 0.83040928 | -0.3443085 | 0.0346766 |  |  |  |
| SF3B3 | 1.05445831 | 0.83040928 | -0.3443085 | 0.0346766 |  |  |  |
| SPIDR | 2.48324096 | 1.95612884 | -0.3440704 | 0.01979116 |  |  |  |
| TARBP1 | 1.13884839 | 0.89719608 | -0.3438007 | 0.04951019 |  |  |  |
| DNMBP | 1.45301347 | 1.1450314 | -0.3434323 | 0.02915385 |  |  |  |
| PDE5A | 1.49562628 | 1.17882713 | -0.3431743 | 0.03851027 |  |  |  |
| DIP2C | 1.59839837 | 1.26009781 | -0.3428798 | 0.02194834 |  |  |  |
| STC2 | 1.66941973 | 1.31642402 | -0.3425183 | 0.02408152 |  |  |  |
| MRGPRF | 4.67320548 | 3.68695284 | -0.3418821 | 0.01872587 |  |  |  |
| SNAP23 | 1.84237763 | 1.45402091 | -0.3413322 | 0.04133547 |  |  |  |
| ZNF703 | 2.85589421 | 2.25465776 | -0.3408968 | 0.02148791 |  |  |  |
| UCK2 | 1.55327892 | 1.22630208 | -0.3407875 | 0.02340635 |  |  |  |
| NCEH1 | 1.69114391 | 1.33573586 | -0.340164 | 0.03647104 |  |  |  |
| IL6R | 1.50398174 | 1.18848305 | -0.339446 | 0.02708311 |  |  |  |
| DMTF1 | 2.41138405 | 1.90704457 | -0.3383696 | 0.03917961 |  |  |  |
| MAMLD1 | 1.36193902 | 1.07743995 | -0.3378179 | 0.02941882 |  |  |  |
| FGFR1 | 1.74712545 | 1.38240615 | -0.3376074 | 0.02364413 |  |  |  |
| DAP | 1.0377474 | 0.82236268 | -0.3353143 | 0.03998284 |  |  |  |
| MTSS1L | 1.53406137 | 1.2158415 | -0.3351876 | 0.02954114 |  |  |  |
| YTHDC1 | 1.17811903 | 0.93421044 | -0.334402 | 0.03301221 |  |  |  |
| BBX | 1.92593217 | 1.5288543 | -0.3329281 | 0.0345288 |  |  |  |
| JAK1 | 1.34188593 | 1.06537005 | -0.3326717 | 0.03057842 |  |  |  |
| LAMB1 | 1.07200476 | 0.85133045 | -0.3322367 | 0.04435808 |  |  |  |
| FBXO4 | 1.07785358 | 0.85615841 | -0.3319297 | 0.04337927 |  |  |  |
| RNF41 | 1.793916 | 1.42505314 | -0.3319095 | 0.02522429 |  |  |  |
| ATF1 | 1.34188593 | 1.06617471 | -0.3315833 | 0.0379863 |  |  |  |
| MESDC1 | 1.55244337 | 1.23354402 | -0.3315224 | 0.0316885 |  |  |  |
| OTULIN | 1.71286809 | 1.36148499 | -0.3310396 | 0.03991104 |  |  |  |
| FNIP2 | 1.00933886 | 0.80224618 | -0.3309966 | 0.0403865 |  |  |  |
| CDC42EP2 | 6.11452131 | 4.86417066 | -0.3299597 | 0.0215147 |  |  |  |
| DPF2 | 6.11452131 | 4.86417066 | -0.3299597 | 0.0215147 |  |  |  |
| NID1 | 1.88833263 | 1.50230052 | -0.3297607 | 0.03320503 |  |  |  |
| PELI2 | 1.21571857 | 0.96720151 | -0.3296678 | 0.03784787 |  |  |  |
| TMEM219 | 1.00432558 | 0.79902754 | -0.329613 | 0.04017288 |  |  |  |
| MYLK | 2.67374531 | 2.12752146 | -0.3295491 | 0.02377698 |  |  |  |
| RBMS3 | 1.0928934 | 0.86983763 | -0.3290587 | 0.04576366 |  |  |  |
| BAZ2B | 1.15138158 | 0.91650792 | -0.3288827 | 0.04519113 |  |  |  |
| FARP1 | 1.34773475 | 1.07341665 | -0.328095 | 0.03641301 |  |  |  |
| C1R | 1.18981666 | 0.94788967 | -0.3276923 | 0.04842941 |  |  |  |
| SEMA5A | 1.91256344 | 1.524831 | -0.3266827 | 0.03414865 |  |  |  |
| FAS | 1.45301347 | 1.15871063 | -0.3263111 | 0.03978736 |  |  |  |
| BAZ1A | 1.4296182 | 1.1410081 | -0.3251022 | 0.04392034 |  |  |  |
| N4BP2 | 2.47321441 | 1.97544069 | -0.324068 | 0.02633099 |  |  |  |
| HIC1 | 5.12189336 | 4.09491554 | -0.3227509 | 0.02243073 |  |  |  |
| KIAA0930 | 1.85156863 | 1.48137935 | -0.3216298 | 0.03556904 |  |  |  |
| ZFP64 | 1.92175444 | 1.53770556 | -0.3214726 | 0.04622642 |  |  |  |
| GAS1 | 2.39467315 | 1.9167005 | -0.3210571 | 0.02805281 |  |  |  |
| BCL2L13 | 2.37211342 | 1.89899797 | -0.320787 | 0.04791252 |  |  |  |
| ZNF207 | 1.4480002 | 1.15951529 | -0.3203268 | 0.03981177 |  |  |  |
| MFAP4 | 1.83151554 | 1.46689547 | -0.3200942 | 0.03830544 |  |  |  |
| GLTSCR1L | 4.14848296 | 3.32324645 | -0.3198873 | 0.02385824 |  |  |  |
| USP13 | 1.40120965 | 1.12250092 | -0.3197379 | 0.04102346 |  |  |  |
| EIF4ENIF1 | 1.43379592 | 1.14905471 | -0.3191782 | 0.04018218 |  |  |  |
| GPRC5C | 1.34105038 | 1.07502597 | -0.3187665 | 0.04501353 |  |  |  |
| KCTD20 | 1.9668739 | 1.57793857 | -0.3176961 | 0.03395534 |  |  |  |
| PXT1 | 1.9668739 | 1.57793857 | -0.3176961 | 0.03395534 |  |  |  |
| ANP32A | 1.52487037 | 1.2238881 | -0.3170125 | 0.03975881 |  |  |  |
| MRC2 | 2.39300205 | 1.92152846 | -0.3164224 | 0.04405462 |  |  |  |
| FAM107A | 1.83485772 | 1.47494207 | -0.3148353 | 0.0488724 |  |  |  |
| HRH1 | 2.33284279 | 1.87646749 | -0.3139234 | 0.0323868 |  |  |  |
| DACT1 | 1.85741745 | 1.49425391 | -0.3137004 | 0.03687539 |  |  |  |
| CNKSR3 | 2.30610533 | 1.85554632 | -0.313467 | 0.04585774 |  |  |  |
| SNRPA1 | 1.72289464 | 1.38723411 | -0.3124416 | 0.04435808 |  |  |  |
| RAI14 | 1.8933459 | 1.524831 | -0.3121195 | 0.03971546 |  |  |  |
| SETD7 | 1.81898236 | 1.46609081 | -0.3109835 | 0.04632783 |  |  |  |
| SEPT9 | 7.93517476 | 6.40107156 | -0.3098746 | 0.02469844 |  |  |  |
| ST3GAL1 | 3.73906571 | 3.02391287 | -0.3061552 | 0.03298912 |  |  |  |
| RFTN1 | 1.757152 | 1.4218345 | -0.3053105 | 0.045464 |  |  |  |
| TRAF3 | 2.33952715 | 1.89577933 | -0.3032843 | 0.04944423 |  |  |  |
| FBXO11 | 2.80743258 | 2.27718825 | -0.3018729 | 0.03997709 |  |  |  |
| GABARAPL1 | 2.79824158 | 2.27960223 | -0.2956155 | 0.04779628 |  |  |  |
| MICALL2 | 3.34468828 | 4.09089224 | 0.29051168 | 0.04739013 |  |  |  |
| HYAL3 | 3.78836289 | 4.63403785 | 0.29067215 | 0.0438257 |  |  |  |
| IFRD2 | 3.78836289 | 4.63403785 | 0.29067215 | 0.0438257 |  |  |  |
| NAT6 | 3.78836289 | 4.63403785 | 0.29067215 | 0.0438257 |  |  |  |
| C12orf57 | 3.28118683 | 4.01444953 | 0.29095077 | 0.04492959 |  |  |  |
| CHD2 | 1.82316009 | 2.23212728 | 0.29188153 | 0.04924047 |  |  |  |
| EGR1 | 13.092161 | 16.0384863 | 0.29285956 | 0.03402399 |  |  |  |
| BORCS6 | 3.8769307 | 4.75151823 | 0.29345154 | 0.04136828 |  |  |  |
| NR4A1 | 9.80596093 | 12.0232321 | 0.294112 | 0.03751173 |  |  |  |
| IRX5 | 2.78654394 | 3.42061033 | 0.29572819 | 0.04192453 |  |  |  |
| HIST1H2AM | 2.63698131 | 3.23714782 | 0.29578193 | 0.04562445 |  |  |  |
| HIST1H2BO | 2.63698131 | 3.23714782 | 0.29578193 | 0.04562445 |  |  |  |
| HIST1H3J | 2.63698131 | 3.23714782 | 0.29578193 | 0.04562445 |  |  |  |
| PTPN6 | 3.2953911 | 4.04824526 | 0.29681251 | 0.03978736 |  |  |  |
| CBX3 | 4.34650722 | 5.34213879 | 0.29754542 | 0.03771376 |  |  |  |
| HNRNPA2B1 | 4.34650722 | 5.34213879 | 0.29754542 | 0.03771376 |  |  |  |
| FAM166A | 4.99405492 | 6.14116633 | 0.29829318 | 0.04519113 |  |  |  |
| TUBB4B | 4.99405492 | 6.14116633 | 0.29829318 | 0.04519113 |  |  |  |
| ARRB2 | 2.0203488 | 2.48479057 | 0.29843499 | 0.04435808 |  |  |  |
| SH2B1 | 2.81244585 | 3.46003868 | 0.29891429 | 0.04005435 |  |  |  |
| ZBTB3 | 1.711197 | 2.10579563 | 0.299251 | 0.04612657 |  |  |  |
| MAP3K11 | 4.4141864 | 5.43387005 | 0.2998176 | 0.03464722 |  |  |  |
| KIAA1328 | 1.79809372 | 2.21362009 | 0.29983803 | 0.04876382 |  |  |  |
| TNK2 | 2.50580069 | 3.08667637 | 0.30072215 | 0.04344961 |  |  |  |
| CDC37 | 1.72707236 | 2.12752146 | 0.30073742 | 0.04701571 |  |  |  |
| RPA3 | 2.03121089 | 2.50249309 | 0.30094105 | 0.04555702 |  |  |  |
| CATSPERG | 2.34119824 | 2.88470667 | 0.30110963 | 0.04237412 |  |  |  |
| ARF3 | 1.81396909 | 2.23615058 | 0.30176666 | 0.0428571 |  |  |  |
| RP11-302B13.5 | 1.81396909 | 2.23615058 | 0.30176666 | 0.0428571 |  |  |  |
| LSS | 1.98776253 | 2.45099484 | 0.30213381 | 0.04868337 |  |  |  |
| FOXF1 | 6.69438983 | 8.25661788 | 0.30260343 | 0.03250651 |  |  |  |
| ADRM1 | 1.94180753 | 2.39547329 | 0.30281892 | 0.04448598 |  |  |  |
| FAM83E | 2.52919596 | 3.12127675 | 0.30339733 | 0.04669455 |  |  |  |
| RPL18 | 2.52919596 | 3.12127675 | 0.30339733 | 0.04669455 |  |  |  |
| TUFM | 1.54993674 | 1.91348186 | 0.30386445 | 0.04618002 |  |  |  |
| FAAP100 | 1.30679302 | 1.61334362 | 0.30386483 | 0.04990694 |  |  |  |
| MBD3L1 | 2.58016423 | 3.18645423 | 0.30443071 | 0.03549539 |  |  |  |
| ZNF558 | 2.58016423 | 3.18645423 | 0.30443071 | 0.03549539 |  |  |  |
| RAB11B | 2.37462006 | 2.93379094 | 0.30500198 | 0.03560027 |  |  |  |
| HFE2 | 3.36390582 | 4.15606972 | 0.30504806 | 0.03301221 |  |  |  |
| SLBP | 1.72205909 | 2.12832612 | 0.30547461 | 0.04212007 |  |  |  |
| RAB3D | 2.49076087 | 3.07862976 | 0.30563953 | 0.04924047 |  |  |  |
| CAPN10 | 1.81814681 | 2.24741582 | 0.3056965 | 0.03830544 |  |  |  |
| PEX10 | 1.50063956 | 1.85554632 | 0.30613348 | 0.0427336 |  |  |  |
| SLC27A1 | 2.71635813 | 3.3586515 | 0.30615482 | 0.04841898 |  |  |  |
| NDUFS8 | 1.60341164 | 1.98268263 | 0.30618722 | 0.04555702 |  |  |  |
| KIAA2026 | 1.56246992 | 1.9327937 | 0.30673297 | 0.04191139 |  |  |  |
| CCDC94 | 1.82399563 | 2.25626708 | 0.3067338 | 0.04327027 |  |  |  |
| CPEB4 | 1.54408792 | 1.91026322 | 0.30688817 | 0.03972542 |  |  |  |
| TMEM18 | 3.07062938 | 3.79880061 | 0.30696716 | 0.02997604 |  |  |  |
| CXXC1 | 1.58001637 | 1.95532418 | 0.30734369 | 0.04077075 |  |  |  |
| DUSP13 | 1.97522935 | 2.44455756 | 0.30746238 | 0.03905086 |  |  |  |
| C6orf1 | 1.52069265 | 1.88210011 | 0.30748349 | 0.04152993 |  |  |  |
| ZNF574 | 2.93025775 | 3.62660333 | 0.30754465 | 0.03803146 |  |  |  |
| EMILIN1 | 1.87830608 | 2.32707718 | 0.30898855 | 0.04005435 |  |  |  |
| SMG7 | 2.65369222 | 3.28784141 | 0.30908304 | 0.03859691 |  |  |  |
| MCM10 | 1.23242948 | 1.52724498 | 0.30925307 | 0.04514999 |  |  |  |
| CUEDC2 | 1.03106304 | 1.27780033 | 0.30931397 | 0.04852642 |  |  |  |
| DUSP8 | 1.65020219 | 2.04544612 | 0.3096544 | 0.04337927 |  |  |  |
| IGSF8 | 1.03189858 | 1.27940965 | 0.30996098 | 0.04989201 |  |  |  |
| SPHK2 | 4.0315066 | 5.00096288 | 0.31086447 | 0.02971881 |  |  |  |
| GFI1B | 1.83235108 | 2.27396961 | 0.31141424 | 0.04439764 |  |  |  |
| ZNF644 | 1.5933851 | 1.97785467 | 0.31171645 | 0.03751261 |  |  |  |
| KIF2C | 0.96839713 | 1.20296694 | 0.31269125 | 0.04760407 |  |  |  |
| KLC4 | 1.09205785 | 1.35665703 | 0.31280332 | 0.04559376 |  |  |  |
| MRPL2 | 1.09205785 | 1.35665703 | 0.31280332 | 0.04559376 |  |  |  |
| SPIRE1 | 1.25248257 | 1.55621275 | 0.31307695 | 0.04703608 |  |  |  |
| EPHA7 | 1.04108958 | 1.29389353 | 0.31340875 | 0.0481596 |  |  |  |
| USP19 | 1.09540003 | 1.36148499 | 0.31352014 | 0.04819794 |  |  |  |
| PPRC1 | 2.31362524 | 2.8758554 | 0.31376315 | 0.04252911 |  |  |  |
| FAH | 1.07116922 | 1.33171256 | 0.31388739 | 0.0444337 |  |  |  |
| IGFLR1 | 1.63265573 | 2.03015758 | 0.31424919 | 0.03697685 |  |  |  |
| NCKIPSD | 1.4020452 | 1.74369856 | 0.31446841 | 0.04958419 |  |  |  |
| ADNP | 1.61343819 | 2.00682243 | 0.31465061 | 0.04834806 |  |  |  |
| KBTBD7 | 1.47223101 | 1.83140652 | 0.3148077 | 0.03457549 |  |  |  |
| HAS2 | 1.00181895 | 1.24641858 | 0.31493969 | 0.04775756 |  |  |  |
| HSPA2 | 2.8391833 | 3.53245809 | 0.31514506 | 0.03476749 |  |  |  |
| NUDT16L1 | 2.01700662 | 2.50973503 | 0.31522872 | 0.03384463 |  |  |  |
| TIPARP | 3.9780317 | 4.95187861 | 0.31589712 | 0.0260286 |  |  |  |
| ULK1 | 2.08217916 | 2.59341969 | 0.31667475 | 0.03144917 |  |  |  |
| MADCAM1 | 1.4204272 | 1.76944769 | 0.31682627 | 0.03717616 |  |  |  |
| CDCA3 | 4.06325733 | 5.06211705 | 0.31708133 | 0.02277708 |  |  |  |
| USP5 | 4.06325733 | 5.06211705 | 0.31708133 | 0.02277708 |  |  |  |
| CCNG1 | 1.37196556 | 1.70990284 | 0.317515 | 0.03492807 |  |  |  |
| FAM86C1 | 2.18244461 | 2.72136065 | 0.31830194 | 0.03109107 |  |  |  |
| CSRP1 | 5.77696097 | 7.20331773 | 0.31834782 | 0.02332408 |  |  |  |
| CABLES2 | 1.53656801 | 1.9167005 | 0.31877907 | 0.0468718 |  |  |  |
| DNAJC25 | 1.11461758 | 1.39045275 | 0.31880401 | 0.04080745 |  |  |  |
| DNAJC25-GNG10 | 1.11461758 | 1.39045275 | 0.31880401 | 0.04080745 |  |  |  |
| NDUFS7 | 1.19566548 | 1.49183993 | 0.31909358 | 0.04240326 |  |  |  |
| RP11-727F15.14 | 6.67350119 | 8.32662332 | 0.31929102 | 0.0195775 |  |  |  |
| STX5 | 6.67350119 | 8.32662332 | 0.31929102 | 0.0195775 |  |  |  |
| WDR74 | 6.67350119 | 8.32662332 | 0.31929102 | 0.0195775 |  |  |  |
| DCTN3 | 0.90907341 | 1.13457082 | 0.31941992 | 0.04876382 |  |  |  |
| PTGER1 | 1.31514848 | 1.64150672 | 0.31963024 | 0.04535104 |  |  |  |
| DACT3 | 2.50580069 | 3.12771403 | 0.3197716 | 0.02571508 |  |  |  |
| NUSAP1 | 2.17826688 | 2.71894667 | 0.31978526 | 0.02653783 |  |  |  |
| OIP5 | 2.17826688 | 2.71894667 | 0.31978526 | 0.02653783 |  |  |  |
| MSTO1 | 0.9383175 | 1.17158519 | 0.32006473 | 0.04223914 |  |  |  |
| EIF2D | 1.09874221 | 1.37194557 | 0.32016404 | 0.03784787 |  |  |  |
| RAB4B | 2.79573494 | 3.49142043 | 0.32053238 | 0.02577457 |  |  |  |
| RAB4B-EGLN2 | 2.79573494 | 3.49142043 | 0.32053238 | 0.02577457 |  |  |  |
| S100A1 | 1.17227021 | 1.46528615 | 0.32168591 | 0.04841898 |  |  |  |
| S100A13 | 1.17227021 | 1.46528615 | 0.32168591 | 0.04841898 |  |  |  |
| C10orf76 | 1.06197822 | 1.32768926 | 0.32194733 | 0.03860962 |  |  |  |
| ZNF581 | 2.5943685 | 3.2435851 | 0.32214369 | 0.02720504 |  |  |  |
| C8orf82 | 3.61540499 | 4.52138543 | 0.32257552 | 0.03560027 |  |  |  |
| LRRC14 | 3.61540499 | 4.52138543 | 0.32257552 | 0.03560027 |  |  |  |
| LRRC24 | 3.61540499 | 4.52138543 | 0.32257552 | 0.03560027 |  |  |  |
| RECQL4 | 3.61540499 | 4.52138543 | 0.32257552 | 0.03560027 |  |  |  |
| ORAI3 | 1.73542782 | 2.17097311 | 0.32293521 | 0.02982752 |  |  |  |
| C5orf45 | 2.30526979 | 2.88390201 | 0.32301074 | 0.02997604 |  |  |  |
| FTH1 | 2.68377186 | 3.35784684 | 0.3232155 | 0.02843568 |  |  |  |
| SMURF1 | 1.9576829 | 2.45019018 | 0.32364926 | 0.0248754 |  |  |  |
| EVI5L | 2.66873204 | 3.34014432 | 0.3236964 | 0.02949234 |  |  |  |
| DDX54 | 1.49144856 | 1.86761623 | 0.32434178 | 0.03383988 |  |  |  |
| RITA1 | 1.49144856 | 1.86761623 | 0.32434178 | 0.03383988 |  |  |  |
| KPNA2 | 1.56079883 | 1.95451952 | 0.32439563 | 0.02698153 |  |  |  |
| RTN4RL2 | 0.80630132 | 1.0098485 | 0.32444725 | 0.04519113 |  |  |  |
| TTF2 | 1.38199211 | 1.730824 | 0.32455275 | 0.03518467 |  |  |  |
| NUP133 | 1.16391476 | 1.45804421 | 0.32485425 | 0.03862404 |  |  |  |
| L3MBTL1 | 1.65855764 | 2.07843719 | 0.32544633 | 0.03884057 |  |  |  |
| AP2S1 | 1.91089235 | 2.39547329 | 0.32596259 | 0.03165652 |  |  |  |
| GTF2H4 | 1.37280111 | 1.72116808 | 0.32610638 | 0.04103972 |  |  |  |
| NKX2-3 | 1.06699149 | 1.33814984 | 0.32647348 | 0.03991104 |  |  |  |
| KCNAB2 | 2.2668347 | 2.843669 | 0.32699538 | 0.0268922 |  |  |  |
| DUSP12 | 1.36611675 | 1.71392614 | 0.32706373 | 0.0296702 |  |  |  |
| CEP131 | 1.45134238 | 1.82094594 | 0.32715176 | 0.03789224 |  |  |  |
| CHEK2 | 1.93679426 | 2.43087834 | 0.32770672 | 0.0227686 |  |  |  |
| HSCB | 1.93679426 | 2.43087834 | 0.32770672 | 0.0227686 |  |  |  |
| DNMT3B | 1.15388821 | 1.44838828 | 0.32774645 | 0.04859412 |  |  |  |
| CDK19 | 1.95183408 | 2.45019018 | 0.32796409 | 0.02917278 |  |  |  |
| ZBTB9 | 1.25164702 | 1.57150129 | 0.32813597 | 0.03625912 |  |  |  |
| HMMR | 1.72707236 | 2.16855913 | 0.3282902 | 0.04841898 |  |  |  |
| NUDCD2 | 1.72707236 | 2.16855913 | 0.3282902 | 0.04841898 |  |  |  |
| ESCO1 | 1.25331812 | 1.57552459 | 0.3298993 | 0.02944986 |  |  |  |
| PLCD1 | 3.96215634 | 4.98165104 | 0.33031149 | 0.01765 |  |  |  |
| CNNM4 | 0.95586395 | 1.20216228 | 0.33050377 | 0.03499665 |  |  |  |
| GRN | 1.52152819 | 1.91348186 | 0.3305382 | 0.04771098 |  |  |  |
| SLC25A42 | 2.35122478 | 2.95712608 | 0.33070773 | 0.0195775 |  |  |  |
| CABP1 | 0.97925922 | 1.2319347 | 0.33091917 | 0.0353933 |  |  |  |
| CYB561D2 | 1.14803939 | 1.44436498 | 0.33106162 | 0.0320927 |  |  |  |
| NPRL2 | 1.14803939 | 1.44436498 | 0.33106162 | 0.0320927 |  |  |  |
| XXcos-LUCA11.5 | 1.14803939 | 1.44436498 | 0.33106162 | 0.0320927 |  |  |  |
| CCDC159 | 1.96102508 | 2.46708804 | 0.33110164 | 0.02460823 |  |  |  |
| TMEM205 | 1.96102508 | 2.46708804 | 0.33110164 | 0.02460823 |  |  |  |
| RAB2B | 1.2917532 | 1.62541352 | 0.33130201 | 0.02856677 |  |  |  |
| TOX4 | 1.2917532 | 1.62541352 | 0.33130201 | 0.02856677 |  |  |  |
| RIN1 | 1.683624 | 2.1186702 | 0.33146559 | 0.0283867 |  |  |  |
| HLA-DPA1 | 0.86311841 | 1.08629121 | 0.33149737 | 0.04149735 |  |  |  |
| PTOV1 | 1.24412712 | 1.56586867 | 0.33164644 | 0.03080825 |  |  |  |
| POP5 | 1.20736312 | 1.5208077 | 0.3327877 | 0.03354888 |  |  |  |
| WIZ | 1.31264184 | 1.65357662 | 0.33294493 | 0.03917961 |  |  |  |
| SYNGR3 | 0.97006822 | 1.22227878 | 0.33316714 | 0.0423338 |  |  |  |
| SRXN1 | 0.99095686 | 1.24883257 | 0.33344378 | 0.0443139 |  |  |  |
| SEC14L5 | 1.5933851 | 2.00843175 | 0.33384053 | 0.02940005 |  |  |  |
| CNIH2 | 1.35358356 | 1.7066842 | 0.33424645 | 0.04349806 |  |  |  |
| INAFM2 | 1.628478 | 2.05349272 | 0.3344253 | 0.02280843 |  |  |  |
| JARID2 | 2.91187575 | 3.67246896 | 0.33474818 | 0.01841318 |  |  |  |
| MYL12A | 1.98024262 | 2.49846979 | 0.33526805 | 0.02602146 |  |  |  |
| ACAD11 | 1.18731003 | 1.49827721 | 0.33541209 | 0.04769207 |  |  |  |
| UBA5 | 1.18731003 | 1.49827721 | 0.33541209 | 0.04769207 |  |  |  |
| MYO15B | 1.13550621 | 1.43309974 | 0.33559657 | 0.03636207 |  |  |  |
| ATF4 | 6.03848668 | 7.6201317 | 0.33562614 | 0.01389668 |  |  |  |
| RPS19BP1 | 6.03848668 | 7.6201317 | 0.33562614 | 0.01389668 |  |  |  |
| CARS2 | 1.26835793 | 1.60207837 | 0.33680166 | 0.02901937 |  |  |  |
| CTU1 | 1.40120965 | 1.77025235 | 0.33712231 | 0.02233167 |  |  |  |
| KRBA1 | 1.01769431 | 1.28584693 | 0.3371777 | 0.02956768 |  |  |  |
| RPL28 | 2.43143714 | 3.07219248 | 0.33738596 | 0.01923654 |  |  |  |
| TMEM238 | 2.43143714 | 3.07219248 | 0.33738596 | 0.01923654 |  |  |  |
| YBX3 | 1.75297427 | 2.21603408 | 0.33805604 | 0.04077075 |  |  |  |
| HIST3H2A | 1.51400828 | 1.91428652 | 0.33828853 | 0.03272502 |  |  |  |
| HIST3H2BB | 1.51400828 | 1.91428652 | 0.33828853 | 0.03272502 |  |  |  |
| WDR1 | 5.65914906 | 7.15584279 | 0.3385303 | 0.015645 |  |  |  |
| THAP10 | 1.44549356 | 1.82818788 | 0.33869784 | 0.02388892 |  |  |  |
| KCTD3 | 0.87815823 | 1.11123568 | 0.3393288 | 0.03549539 |  |  |  |
| SPATA2 | 1.11628867 | 1.41298324 | 0.33982061 | 0.02909982 |  |  |  |
| TRMT61A | 0.79293259 | 1.00421588 | 0.34047994 | 0.03728618 |  |  |  |
| ALG3 | 1.34439257 | 1.70266089 | 0.34066647 | 0.02416842 |  |  |  |
| EEF1AKMT4 | 1.34439257 | 1.70266089 | 0.34066647 | 0.02416842 |  |  |  |
| EEF1AKMT4-ECE2 | 1.34439257 | 1.70266089 | 0.34066647 | 0.02416842 |  |  |  |
| CHD4 | 1.32517502 | 1.67852109 | 0.34083436 | 0.0438257 |  |  |  |
| SERGEF | 1.09874221 | 1.39206207 | 0.34115212 | 0.02565628 |  |  |  |
| FAM64A | 1.2917532 | 1.63667876 | 0.34126148 | 0.0241511 |  |  |  |
| SPATA2L | 2.05878389 | 2.60870823 | 0.34144726 | 0.0320927 |  |  |  |
| TYRO3 | 1.20067875 | 1.52161236 | 0.3415543 | 0.02554533 |  |  |  |
| CDK1 | 1.21906075 | 1.5449475 | 0.34159503 | 0.02295973 |  |  |  |
| TBL3 | 0.88150041 | 1.11767296 | 0.34218093 | 0.03283719 |  |  |  |
| COPS7B | 2.84503212 | 3.60729149 | 0.34241373 | 0.01667746 |  |  |  |
| HYAL2 | 1.92258999 | 2.43812028 | 0.3426119 | 0.01853232 |  |  |  |
| CENPM | 2.40386415 | 3.049662 | 0.34321775 | 0.01740899 |  |  |  |
| PRPF31 | 1.04443176 | 1.32527528 | 0.34334034 | 0.02602679 |  |  |  |
| TFPT | 1.04443176 | 1.32527528 | 0.34334034 | 0.02602679 |  |  |  |
| TOMM34 | 0.94416631 | 1.19813898 | 0.34341929 | 0.02966368 |  |  |  |
| RCN3 | 0.96254831 | 1.22147412 | 0.34343524 | 0.02673278 |  |  |  |
| NR1D1 | 2.49076087 | 3.1607051 | 0.34358779 | 0.01831024 |  |  |  |
| GUK1 | 1.89167481 | 2.40110591 | 0.34392577 | 0.02900585 |  |  |  |
| DBP | 3.20181001 | 4.06433846 | 0.34408653 | 0.0157901 |  |  |  |
| USP8 | 1.22323848 | 1.5529941 | 0.34415338 | 0.02882566 |  |  |  |
| GON4L | 2.25012379 | 2.85734822 | 0.34458762 | 0.01639035 |  |  |  |
| ALS2CL | 1.22073184 | 1.55058012 | 0.34486762 | 0.04519422 |  |  |  |
| TMIE | 1.22073184 | 1.55058012 | 0.34486762 | 0.04519422 |  |  |  |
| MPRIP | 1.61343819 | 2.04946942 | 0.34497607 | 0.02016247 |  |  |  |
| FOXH1 | 1.674433 | 2.12832612 | 0.34591671 | 0.04457133 |  |  |  |
| ZSCAN20 | 1.78639609 | 2.27075097 | 0.34599853 | 0.02016613 |  |  |  |
| C6orf48 | 1.30177975 | 1.65518595 | 0.34632794 | 0.03066774 |  |  |  |
| APEH | 2.3846466 | 3.03276414 | 0.34677966 | 0.01850893 |  |  |  |
| TESK2 | 1.69197946 | 2.15246592 | 0.34714983 | 0.01597588 |  |  |  |
| REXO4 | 0.97758813 | 1.24480926 | 0.34837028 | 0.03784787 |  |  |  |
| MAPT | 1.02939195 | 1.3107914 | 0.34840505 | 0.03558948 |  |  |  |
| ARL4A | 2.07967253 | 2.64894124 | 0.34896265 | 0.01352247 |  |  |  |
| SHISA4 | 1.90587908 | 2.4276597 | 0.3489999 | 0.02279582 |  |  |  |
| MED11 | 1.32266838 | 1.68495837 | 0.34908379 | 0.02119497 |  |  |  |
| NEURL4 | 1.60006946 | 2.03900884 | 0.34959407 | 0.04385733 |  |  |  |
| CTSC | 0.88734923 | 1.13135218 | 0.35018662 | 0.0407343 |  |  |  |
| CCNB1 | 1.13968394 | 1.45321625 | 0.35040144 | 0.03438349 |  |  |  |
| C19orf70 | 2.38213996 | 3.0375921 | 0.35059067 | 0.01398716 |  |  |  |
| HSD11B1L | 2.38213996 | 3.0375921 | 0.35059067 | 0.01398716 |  |  |  |
| TEAD2 | 2.25931479 | 2.88148803 | 0.35084263 | 0.01255312 |  |  |  |
| DET1 | 0.94333077 | 1.2037716 | 0.35145733 | 0.02492697 |  |  |  |
| C19orf73 | 1.58586519 | 2.0237203 | 0.3515979 | 0.02633898 |  |  |  |
| LRSAM1 | 2.47237887 | 3.15507248 | 0.35169824 | 0.01554335 |  |  |  |
| RPL12 | 2.47237887 | 3.15507248 | 0.35169824 | 0.01554335 |  |  |  |
| XKR8 | 0.82886105 | 1.05812811 | 0.35199975 | 0.02856407 |  |  |  |
| STMN1 | 1.45969783 | 1.86359293 | 0.35225874 | 0.02078359 |  |  |  |
| ELL3 | 1.23326503 | 1.57471993 | 0.35241682 | 0.02788319 |  |  |  |
| NET1 | 1.18647448 | 1.51517508 | 0.35259829 | 0.01909951 |  |  |  |
| SRSF10 | 0.98594358 | 1.25929315 | 0.35278113 | 0.02333354 |  |  |  |
| LZTFL1 | 1.02020095 | 1.30354946 | 0.35334569 | 0.02119497 |  |  |  |
| NUF2 | 1.27086457 | 1.6238042 | 0.35337844 | 0.01938236 |  |  |  |
| FZD5 | 1.22992284 | 1.57150129 | 0.35337892 | 0.02032363 |  |  |  |
| SERBP1 | 1.33687266 | 1.70829352 | 0.35351634 | 0.01588973 |  |  |  |
| SRI | 1.29509539 | 1.65518595 | 0.35375018 | 0.02482586 |  |  |  |
| ESRRA | 2.17325361 | 2.77849153 | 0.35435301 | 0.01415717 |  |  |  |
| DKKL1 | 2.23341288 | 2.85654356 | 0.35493178 | 0.01137994 |  |  |  |
| MCF2L | 1.71621027 | 2.19511291 | 0.35493988 | 0.019002 |  |  |  |
| MRPL39 | 0.89570468 | 1.14583607 | 0.35501773 | 0.02261181 |  |  |  |
| CSK | 0.91325113 | 1.16836655 | 0.35512785 | 0.02633099 |  |  |  |
| ATP5D | 1.79892927 | 2.30132805 | 0.35520713 | 0.01548382 |  |  |  |
| CBARP | 1.79892927 | 2.30132805 | 0.35520713 | 0.01548382 |  |  |  |
| CLDN12 | 0.9917924 | 1.26894907 | 0.35526825 | 0.02104878 |  |  |  |
| CERCAM | 1.60090501 | 2.04866476 | 0.35565451 | 0.01584335 |  |  |  |
| NFYC | 2.35790915 | 3.01747559 | 0.35575202 | 0.01082141 |  |  |  |
| HDAC3 | 1.83903545 | 2.35363096 | 0.35582118 | 0.01708553 |  |  |  |
| RELL2 | 1.83903545 | 2.35363096 | 0.35582118 | 0.01708553 |  |  |  |
| ARPC1B | 1.18814557 | 1.5208077 | 0.35591959 | 0.01935281 |  |  |  |
| FANCE | 1.33436602 | 1.70829352 | 0.35622224 | 0.01646528 |  |  |  |
| VPRBP | 2.41556178 | 3.09472297 | 0.35737216 | 0.01148148 |  |  |  |
| ZNF571 | 0.82134114 | 1.05249549 | 0.35744076 | 0.02997604 |  |  |  |
| PC | 2.4397926 | 3.12610471 | 0.35752943 | 0.01006515 |  |  |  |
| PCDH7 | 1.37196556 | 1.75818245 | 0.3576668 | 0.02708295 |  |  |  |
| ZNF628 | 1.22156739 | 1.56586867 | 0.358029 | 0.01548382 |  |  |  |
| CACFD1 | 1.23911384 | 1.58839915 | 0.35806745 | 0.02814279 |  |  |  |
| PAX9 | 1.05947158 | 1.35907101 | 0.35903706 | 0.01838372 |  |  |  |
| TWF2 | 1.55745664 | 1.99797117 | 0.35919543 | 0.01567673 |  |  |  |
| TSPAN31 | 1.41792056 | 1.81933662 | 0.35946835 | 0.01789238 |  |  |  |
| CENPU | 1.46304001 | 1.87727215 | 0.35950767 | 0.01399829 |  |  |  |
| LIPT2 | 1.00683222 | 1.29228421 | 0.35984591 | 0.01936025 |  |  |  |
| PRELID2 | 0.77956387 | 1.00099724 | 0.36035717 | 0.0265507 |  |  |  |
| CELSR3 | 3.52433054 | 4.52460407 | 0.36040139 | 0.01042189 |  |  |  |
| LTA4H | 1.86995063 | 2.40110591 | 0.36058229 | 0.01163426 |  |  |  |
| SLC35G2 | 0.95920613 | 1.2319347 | 0.36074338 | 0.01987071 |  |  |  |
| RABAC1 | 1.4112362 | 1.81289934 | 0.36117015 | 0.01481224 |  |  |  |
| NXPH4 | 2.44229923 | 3.13736996 | 0.3612367 | 0.01738246 |  |  |  |
| PPWD1 | 0.93246868 | 1.19813898 | 0.36138903 | 0.02133722 |  |  |  |
| TTLL1 | 0.86061177 | 1.10640772 | 0.36214202 | 0.02269354 |  |  |  |
| PTGER4 | 1.02270758 | 1.3148147 | 0.36221477 | 0.04346185 |  |  |  |
| MRPL57 | 1.27671339 | 1.64150672 | 0.36239366 | 0.03462673 |  |  |  |
| SKA3 | 1.27671339 | 1.64150672 | 0.36239366 | 0.03462673 |  |  |  |
| POLL | 0.80713687 | 1.0380116 | 0.36260709 | 0.02792272 |  |  |  |
| VPS51 | 0.80713687 | 1.0380116 | 0.36260709 | 0.03591878 |  |  |  |
| DNLZ | 2.2208797 | 2.8557389 | 0.36264113 | 0.01215056 |  |  |  |
| RAB43 | 1.47975092 | 1.90302127 | 0.36277735 | 0.03815687 |  |  |  |
| SLC16A13 | 1.10542658 | 1.4218345 | 0.36292104 | 0.02214687 |  |  |  |
| RAVER1 | 2.36041578 | 3.03598278 | 0.36303884 | 0.01088251 |  |  |  |
| HDAC1 | 0.92745541 | 1.19331101 | 0.36333816 | 0.03932108 |  |  |  |
| SNAP47 | 1.44465801 | 1.85876497 | 0.36345111 | 0.01567673 |  |  |  |
| EIF4EBP3 | 1.44465801 | 1.85876497 | 0.36345111 | 0.01965627 |  |  |  |
| RUNDC3B | 0.90823786 | 1.16917121 | 0.3640546 | 0.01977223 |  |  |  |
| MAD2L2 | 1.32517502 | 1.70587954 | 0.36414838 | 0.01421852 |  |  |  |
| CDCA5 | 0.7912615 | 1.01869976 | 0.36416344 | 0.02305091 |  |  |  |
| ZFPL1 | 0.7912615 | 1.01869976 | 0.36416344 | 0.02305091 |  |  |  |
| DHCR7 | 1.31264184 | 1.69059099 | 0.36486734 | 0.01739152 |  |  |  |
| MIEF1 | 0.90823786 | 1.16997587 | 0.36504648 | 0.01877702 |  |  |  |
| MRPS36 | 1.1112754 | 1.43149042 | 0.36507255 | 0.01615623 |  |  |  |
| P2RY6 | 0.80630132 | 1.03881626 | 0.36521686 | 0.03286358 |  |  |  |
| SOD2 | 1.82483118 | 2.35121698 | 0.36552243 | 0.0143586 |  |  |  |
| DPP9 | 2.50914287 | 3.23312451 | 0.36565765 | 0.0141226 |  |  |  |
| JMJD8 | 1.01184549 | 1.30435412 | 0.36609001 | 0.02669601 |  |  |  |
| CTC-512J12.6 | 0.85726959 | 1.10560306 | 0.36670132 | 0.01866727 |  |  |  |
| ZNF285 | 0.85726959 | 1.10560306 | 0.36670132 | 0.01866727 |  |  |  |
| SMARCD1 | 1.10876876 | 1.4298811 | 0.36670606 | 0.01423214 |  |  |  |
| ARHGAP33 | 1.01184549 | 1.30515878 | 0.36697919 | 0.01692893 |  |  |  |
| ENOX1 | 1.19566548 | 1.54253352 | 0.36727711 | 0.01414612 |  |  |  |
| TMEM170B | 0.99429904 | 1.28343295 | 0.36799312 | 0.01537641 |  |  |  |
| FAM184A | 1.1096043 | 1.43229508 | 0.3680523 | 0.01523165 |  |  |  |
| SPOCD1 | 0.88818477 | 1.14664073 | 0.36818172 | 0.02392036 |  |  |  |
| EPB41 | 1.21655412 | 1.57069663 | 0.36839696 | 0.01331304 |  |  |  |
| ZNF730 | 0.95335731 | 1.23113004 | 0.36861752 | 0.0272372 |  |  |  |
| TCAIM | 1.35441911 | 1.75013584 | 0.36961062 | 0.01152845 |  |  |  |
| KCNN1 | 0.90990895 | 1.17641315 | 0.37030752 | 0.02388892 |  |  |  |
| CA5B | 1.61928701 | 2.09372573 | 0.37056839 | 0.01667746 |  |  |  |
| TNFRSF10D | 1.48894192 | 1.92635642 | 0.37142484 | 0.01367806 |  |  |  |
| GCHFR | 1.00432558 | 1.29952616 | 0.37149644 | 0.01621752 |  |  |  |
| RNASEH1 | 1.49980401 | 1.9408403 | 0.37174645 | 0.00925316 |  |  |  |
| HIST1H2BH | 1.0469384 | 1.35504771 | 0.37191713 | 0.01452904 |  |  |  |
| HIST1H3F | 1.0469384 | 1.35504771 | 0.37191713 | 0.01452904 |  |  |  |
| NFIA | 1.80644918 | 2.33834242 | 0.37220329 | 0.01024653 |  |  |  |
| ATP5I | 2.71050931 | 3.50912295 | 0.37247718 | 0.01024653 |  |  |  |
| MYL5 | 2.71050931 | 3.50912295 | 0.37247718 | 0.01024653 |  |  |  |
| NUP155 | 1.06782703 | 1.38321081 | 0.37309818 | 0.01298227 |  |  |  |
| BRCA1 | 1.13634176 | 1.47252809 | 0.37367061 | 0.01239302 |  |  |  |
| FLNA | 5.14194645 | 6.66339077 | 0.37392623 | 0.00700637 |  |  |  |
| LRRC74B | 0.9199355 | 1.19250635 | 0.37410026 | 0.01479078 |  |  |  |
| NT5DC3 | 0.85643405 | 1.11043102 | 0.37438847 | 0.0152524 |  |  |  |
| KIF23 | 1.637669 | 2.12349816 | 0.37465428 | 0.01475179 |  |  |  |
| PYGM | 0.78791932 | 1.0219184 | 0.37481115 | 0.02839197 |  |  |  |
| SHF | 2.7581354 | 3.5767144 | 0.37487371 | 0.0104688 |  |  |  |
| RP11-307N16.6 | 0.92494877 | 1.1997483 | 0.37499554 | 0.01400918 |  |  |  |
| KCNIP3 | 1.09122231 | 1.41539722 | 0.37502217 | 0.02078359 |  |  |  |
| ETV2 | 2.07215262 | 2.68756493 | 0.37506413 | 0.00763614 |  |  |  |
| CLU | 1.33687266 | 1.73404264 | 0.37508975 | 0.01108945 |  |  |  |
| MTMR4 | 3.10321565 | 4.02812875 | 0.37629013 | 0.00708881 |  |  |  |
| CRABP2 | 1.02688531 | 1.33332188 | 0.37649158 | 0.02119497 |  |  |  |
| PPP1R3C | 1.36110347 | 1.76783837 | 0.37702531 | 0.0122463 |  |  |  |
| CENPF | 1.2842333 | 1.66886517 | 0.37776172 | 0.01070689 |  |  |  |
| PIGU | 1.14051948 | 1.48218401 | 0.37780447 | 0.01083402 |  |  |  |
| ACADVL | 3.61206281 | 4.6935827 | 0.37782475 | 0.00610151 |  |  |  |
| DLG4 | 3.61206281 | 4.6935827 | 0.37782475 | 0.00610151 |  |  |  |
| TGFB1I1 | 1.5106661 | 1.96337079 | 0.37798577 | 0.00957215 |  |  |  |
| PAQR4 | 1.35525466 | 1.76220575 | 0.37863237 | 0.03268685 |  |  |  |
| TNIK | 1.04777394 | 1.36309431 | 0.37930379 | 0.01148595 |  |  |  |
| TUBGCP6 | 1.30512193 | 1.69783293 | 0.37931468 | 0.01244772 |  |  |  |
| COPS7A | 0.95920613 | 1.24802791 | 0.37945564 | 0.01756218 |  |  |  |
| ZNF414 | 1.58920737 | 2.06797661 | 0.37975993 | 0.0073929 |  |  |  |
| PKMYT1 | 0.76953732 | 1.0018019 | 0.3801713 | 0.02067139 |  |  |  |
| GTPBP6 | 2.73474013 | 3.5606212 | 0.38065501 | 0.00519621 |  |  |  |
| EIF4A2 | 2.03622416 | 2.65215988 | 0.38116165 | 0.00935956 |  |  |  |
| HSPA5 | 4.0590796 | 5.28822656 | 0.38159844 | 0.00454153 |  |  |  |
| CDK2AP2 | 3.60203626 | 4.6935827 | 0.38183409 | 0.00481068 |  |  |  |
| PITPNM1 | 3.60203626 | 4.6935827 | 0.38183409 | 0.00481068 |  |  |  |
| CD55 | 1.44215138 | 1.87968613 | 0.38209554 | 0.01567295 |  |  |  |
| ZNF559 | 1.03941849 | 1.35504771 | 0.38230873 | 0.02055914 |  |  |  |
| ZNF559-ZNF177 | 1.03941849 | 1.35504771 | 0.38230873 | 0.02055914 |  |  |  |
| CTB-43P18.3 | 0.9642194 | 1.25768383 | 0.38305355 | 0.02078359 |  |  |  |
| SYT11 | 1.62012255 | 2.11384224 | 0.38361451 | 0.00722057 |  |  |  |
| MAML3 | 1.63182019 | 2.12913078 | 0.38363361 | 0.01024747 |  |  |  |
| CCNO | 0.84807859 | 1.10721238 | 0.38433404 | 0.0159761 |  |  |  |
| RPL36 | 6.02094023 | 7.85992042 | 0.38451793 | 0.00407221 |  |  |  |
| HECTD3 | 1.69532164 | 2.21362009 | 0.38470663 | 0.0084676 |  |  |  |
| UROD | 1.69532164 | 2.21362009 | 0.38470663 | 0.0084676 |  |  |  |
| EXT1 | 3.42573618 | 4.47471514 | 0.38533389 | 0.0045725 |  |  |  |
| MFSD7 | 2.75395767 | 3.59763557 | 0.38547219 | 0.00732461 |  |  |  |
| OSBPL9 | 1.28506884 | 1.67932575 | 0.38583436 | 0.01454995 |  |  |  |
| FAM200B | 1.22156739 | 1.59644575 | 0.38591512 | 0.0082182 |  |  |  |
| GPR162 | 1.15806594 | 1.51356576 | 0.38600472 | 0.00978989 |  |  |  |
| BMF | 1.44465801 | 1.88853739 | 0.38636625 | 0.0075943 |  |  |  |
| L3MBTL2 | 1.01184549 | 1.32447062 | 0.38815674 | 0.0100117 |  |  |  |
| CFAP99 | 1.01100995 | 1.32366596 | 0.38847135 | 0.02416842 |  |  |  |
| NAA40 | 0.76870178 | 1.00662986 | 0.38866739 | 0.01733438 |  |  |  |
| ADGRE3 | 1.01351658 | 1.32768926 | 0.38927758 | 0.02088469 |  |  |  |
| ZNF333 | 1.01351658 | 1.32768926 | 0.38927758 | 0.02088469 |  |  |  |
| SETD6 | 1.42627602 | 1.86842089 | 0.38938706 | 0.00956814 |  |  |  |
| TMEM135 | 0.9366464 | 1.22710674 | 0.38938817 | 0.01040601 |  |  |  |
| DZIP3 | 1.10291994 | 1.44516964 | 0.38966583 | 0.00890421 |  |  |  |
| KIAA1524 | 1.13216403 | 1.48379333 | 0.38996952 | 0.00858125 |  |  |  |
| DPH2 | 1.09540003 | 1.43631838 | 0.39067024 | 0.01065065 |  |  |  |
| PDE4C | 1.74461882 | 2.28764883 | 0.39081486 | 0.0055259 |  |  |  |
| CENPK | 0.93998859 | 1.23273936 | 0.39085675 | 0.01104333 |  |  |  |
| RNPEPL1 | 1.48810638 | 1.9529102 | 0.39197697 | 0.00935956 |  |  |  |
| NFIB | 0.82886105 | 1.08790053 | 0.3920021 | 0.01224309 |  |  |  |
| ARF4 | 4.2044645 | 5.51835936 | 0.39228554 | 0.00405576 |  |  |  |
| GLT25D1 | 1.15973703 | 1.52241702 | 0.3923337 | 0.04747 |  |  |  |
| MDH1 | 1.83652881 | 2.41076183 | 0.39237736 | 0.00501666 |  |  |  |
| WDPCP | 1.83652881 | 2.41076183 | 0.39237736 | 0.00501666 |  |  |  |
| RFX7 | 1.05362276 | 1.38321081 | 0.39240254 | 0.01159461 |  |  |  |
| ACP2 | 0.84139423 | 1.1047984 | 0.39259225 | 0.02997604 |  |  |  |
| CHN1 | 0.96087722 | 1.26170713 | 0.3926634 | 0.00945851 |  |  |  |
| NPM1 | 1.86911508 | 2.45501814 | 0.39325081 | 0.00660265 |  |  |  |
| FAM43A | 2.86341412 | 3.76098158 | 0.39330514 | 0.00520962 |  |  |  |
| AAMP | 3.0121412 | 3.95731866 | 0.39367154 | 0.00407537 |  |  |  |
| RPL39L | 0.84724305 | 1.11364966 | 0.39411213 | 0.01070184 |  |  |  |
| HIST1H1E | 2.43728596 | 3.20335209 | 0.39421922 | 0.00679381 |  |  |  |
| HIST1H2BD | 2.43728596 | 3.20335209 | 0.39421922 | 0.00679381 |  |  |  |
| EEFSEC | 1.07952467 | 1.41942052 | 0.39465179 | 0.01850524 |  |  |  |
| CLPB | 1.49562628 | 1.96658943 | 0.39477501 | 0.01501981 |  |  |  |
| SNTA1 | 1.0745114 | 1.41298324 | 0.39480802 | 0.00890487 |  |  |  |
| C12orf4 | 0.86562505 | 1.13859412 | 0.39511126 | 0.02080044 |  |  |  |
| RAD51AP1 | 0.86562505 | 1.13859412 | 0.39511126 | 0.02080044 |  |  |  |
| ACTB | 11.9666814 | 15.7415667 | 0.39557355 | 0.00253967 |  |  |  |
| ZNF580 | 1.85240417 | 2.43731562 | 0.39576431 | 0.0069669 |  |  |  |
| CCDC184 | 0.92327768 | 1.21503684 | 0.39585816 | 0.01042774 |  |  |  |
| FBXL18 | 11.9742013 | 15.7576599 | 0.3961414 | 0.00249195 |  |  |  |
| TNFAIP8L1 | 0.96338386 | 1.26814441 | 0.39624532 | 0.00856186 |  |  |  |
| PNP | 0.94416631 | 1.24319994 | 0.39664727 | 0.01006556 |  |  |  |
| CEBPA | 0.8004525 | 1.05410481 | 0.39677085 | 0.011161 |  |  |  |
| GPC2 | 1.23577166 | 1.62863216 | 0.39803062 | 0.01054989 |  |  |  |
| STAG3 | 1.23577166 | 1.62863216 | 0.39803062 | 0.01054989 |  |  |  |
| CD3EAP | 2.26850579 | 2.99011715 | 0.39836109 | 0.00942574 |  |  |  |
| PPP1R13L | 2.26850579 | 2.99011715 | 0.39836109 | 0.00942574 |  |  |  |
| BAIAP2 | 4.07161278 | 5.36869257 | 0.39893514 | 0.00388714 |  |  |  |
| PXMP4 | 1.04025404 | 1.37275023 | 0.39986487 | 0.01018397 |  |  |  |
| PLAT | 0.86980277 | 1.14825005 | 0.40034659 | 0.01548382 |  |  |  |
| LAMA5 | 1.26334466 | 1.66806051 | 0.40070979 | 0.00924 |  |  |  |
| PTK6 | 1.41624947 | 1.87003021 | 0.40080043 | 0.00637806 |  |  |  |
| RAB3C | 0.90907341 | 1.20055296 | 0.4009161 | 0.01106603 |  |  |  |
| TPM2 | 5.16617727 | 6.82190882 | 0.40105969 | 0.00326164 |  |  |  |
| LGALS1 | 3.67305762 | 4.85049143 | 0.40110508 | 0.00271145 |  |  |  |
| YPEL4 | 1.19900766 | 1.58357119 | 0.40111325 | 0.01108945 |  |  |  |
| APBA3 | 1.76550745 | 2.33190514 | 0.40128584 | 0.00676836 |  |  |  |
| MRPL54 | 1.76550745 | 2.33190514 | 0.40128584 | 0.00676836 |  |  |  |
| UBE2C | 1.4296182 | 1.88853739 | 0.40145559 | 0.00831331 |  |  |  |
| PPAN | 0.96589049 | 1.27619101 | 0.40161915 | 0.00741605 |  |  |  |
| PPAN-P2RY11 | 0.96589049 | 1.27619101 | 0.40161915 | 0.00741605 |  |  |  |
| TMEM192 | 1.25833139 | 1.66323255 | 0.40226265 | 0.00559232 |  |  |  |
| ENPP3 | 1.10542658 | 1.46126285 | 0.40236118 | 0.00729263 |  |  |  |
| RBAK | 0.95753504 | 1.26653509 | 0.40319232 | 0.00715987 |  |  |  |
| RBAK-RBAKDN | 0.95753504 | 1.26653509 | 0.40319232 | 0.00715987 |  |  |  |
| SLC44A1 | 1.14302612 | 1.51195644 | 0.4033161 | 0.00600802 |  |  |  |
| RNF26 | 1.02187204 | 1.35182907 | 0.40342205 | 0.00798361 |  |  |  |
| NRN1L | 0.79878141 | 1.05732345 | 0.40417785 | 0.0092299 |  |  |  |
| FIZ1 | 2.80993921 | 3.71913925 | 0.40435754 | 0.00420382 |  |  |  |
| ZNF524 | 2.80993921 | 3.71913925 | 0.40435754 | 0.00420382 |  |  |  |
| NAGS | 1.45468456 | 1.92555176 | 0.40438441 | 0.00467927 |  |  |  |
| PYY | 1.45468456 | 1.92555176 | 0.40438441 | 0.00467927 |  |  |  |
| UBIAD1 | 1.04025404 | 1.37757819 | 0.40492697 | 0.00576022 |  |  |  |
| WIPI2 | 0.97925922 | 1.29711217 | 0.40524931 | 0.04523294 |  |  |  |
| YIF1B | 1.33436602 | 1.76783837 | 0.40562986 | 0.00453562 |  |  |  |
| ZSCAN25 | 0.83805205 | 1.11043102 | 0.40565993 | 0.01039368 |  |  |  |
| ADCK5 | 1.2750423 | 1.68978633 | 0.40608198 | 0.0055396 |  |  |  |
| UXT | 0.88651368 | 1.17560849 | 0.40686543 | 0.0108041 |  |  |  |
| INHBA | 1.72122355 | 2.28362553 | 0.40774423 | 0.00350819 |  |  |  |
| GCA | 1.21738966 | 1.61656226 | 0.40891067 | 0.00514887 |  |  |  |
| BHLHE40 | 1.9209189 | 2.5507727 | 0.4090099 | 0.0106091 |  |  |  |
| MAGED2 | 0.81215014 | 1.07904927 | 0.40957885 | 0.00734926 |  |  |  |
| CTD-2515O10.6 | 0.84724305 | 1.12571956 | 0.40965298 | 0.0083637 |  |  |  |
| RP1-56K13.1 | 0.9199355 | 1.22308344 | 0.41060181 | 0.0080576 |  |  |  |
| FAM117A | 3.11491329 | 4.14239049 | 0.41120936 | 0.00209357 |  |  |  |
| AFMID | 1.3652812 | 1.81611798 | 0.41146073 | 0.00443147 |  |  |  |
| TK1 | 1.3652812 | 1.81611798 | 0.41146073 | 0.00443147 |  |  |  |
| HIST1H1B | 1.5474301 | 2.05912534 | 0.41198681 | 0.00465065 |  |  |  |
| HIST1H2AL | 1.5474301 | 2.05912534 | 0.41198681 | 0.00465065 |  |  |  |
| IMPDH2 | 0.88150041 | 1.17319451 | 0.41207593 | 0.00751776 |  |  |  |
| IARS2 | 0.80880796 | 1.07663529 | 0.41229327 | 0.01440422 |  |  |  |
| PNKD | 4.07495497 | 5.42662811 | 0.41323457 | 0.00181239 |  |  |  |
| MYH9 | 7.18067726 | 9.56338598 | 0.41339963 | 0.00154446 |  |  |  |
| C8orf41 | 1.35191247 | 1.80082943 | 0.41345743 | 0.00537425 |  |  |  |
| PISD | 1.79140936 | 2.38662203 | 0.41373316 | 0.00334267 |  |  |  |
| BCS1L | 0.91241559 | 1.21664616 | 0.41482447 | 0.0070426 |  |  |  |
| ZNF142 | 0.91241559 | 1.21664616 | 0.41482447 | 0.0070426 |  |  |  |
| PFKFB4 | 2.38882433 | 3.18645423 | 0.41555544 | 0.00409065 |  |  |  |
| UCN2 | 2.38882433 | 3.18645423 | 0.41555544 | 0.00409065 |  |  |  |
| EPOP | 0.99513458 | 1.32768926 | 0.41566177 | 0.00723601 |  |  |  |
| EFCAB12 | 0.89069141 | 1.18848305 | 0.41579198 | 0.03268685 |  |  |  |
| KLHL12 | 0.92077104 | 1.22871606 | 0.41591764 | 0.0066864 |  |  |  |
| WDR7 | 0.95001513 | 1.26814441 | 0.41638799 | 0.00642194 |  |  |  |
| C9orf172 | 4.1601806 | 5.55537373 | 0.41720156 | 0.0021222 |  |  |  |
| MAMDC4 | 4.1601806 | 5.55537373 | 0.41720156 | 0.0021222 |  |  |  |
| PHPT1 | 4.1601806 | 5.55537373 | 0.41720156 | 0.0021222 |  |  |  |
| ATP8B3 | 1.77804063 | 2.37535679 | 0.41771129 | 0.00478898 |  |  |  |
| ART5 | 0.93998859 | 1.25607451 | 0.41789351 | 0.00773898 |  |  |  |
| AC006538.4 | 0.82969659 | 1.1088217 | 0.41801058 | 0.00677543 |  |  |  |
| SLC39A3 | 0.82969659 | 1.1088217 | 0.41801058 | 0.00677543 |  |  |  |
| E2F1 | 1.19148775 | 1.59242245 | 0.41822098 | 0.00615137 |  |  |  |
| TRIM11 | 0.97508149 | 1.30354946 | 0.4185497 | 0.00441351 |  |  |  |
| RND3 | 7.19655262 | 9.62614947 | 0.41965047 | 0.00135987 |  |  |  |
| C10orf55 | 3.17925029 | 4.25504292 | 0.4204253 | 0.00195689 |  |  |  |
| PLAU | 3.17925029 | 4.25504292 | 0.4204253 | 0.00195689 |  |  |  |
| RBM28 | 0.92327768 | 1.235958 | 0.42047166 | 0.00763466 |  |  |  |
| TMEM179B | 2.45316132 | 3.2838181 | 0.4206388 | 0.00260737 |  |  |  |
| TMEM223 | 2.45316132 | 3.2838181 | 0.4206388 | 0.00260737 |  |  |  |
| AP3D1 | 1.61009601 | 2.15568456 | 0.42083383 | 0.00400914 |  |  |  |
| PCGF1 | 0.88567813 | 1.18606907 | 0.42099618 | 0.00581591 |  |  |  |
| NRXN2 | 0.7653596 | 1.02513704 | 0.42120949 | 0.01707239 |  |  |  |
| INO80B | 1.1831323 | 1.58598517 | 0.42252568 | 0.00334468 |  |  |  |
| INO80B-WBP1 | 1.1831323 | 1.58598517 | 0.42252568 | 0.00334468 |  |  |  |
| TTYH2 | 1.38700538 | 1.86037429 | 0.42341908 | 0.00333115 |  |  |  |
| GABBR1 | 2.60105286 | 3.48981111 | 0.42396699 | 0.00238982 |  |  |  |
| MAFA | 1.70869036 | 2.29408611 | 0.42487372 | 0.00812641 |  |  |  |
| SLC25A46 | 1.02604976 | 1.37838285 | 0.42558808 | 0.00393841 |  |  |  |
| IP6K2 | 1.9484919 | 2.61755949 | 0.42573383 | 0.00230212 |  |  |  |
| KCNH2 | 0.94416631 | 1.26894907 | 0.42620416 | 0.00396453 |  |  |  |
| NUP54 | 1.26418021 | 1.69944225 | 0.42663345 | 0.0029162 |  |  |  |
| GLI1 | 2.53504477 | 3.40773577 | 0.42671196 | 0.00180291 |  |  |  |
| INHBE | 2.53504477 | 3.40773577 | 0.42671196 | 0.00180291 |  |  |  |
| PFDN6 | 1.03106304 | 1.38723411 | 0.4277912 | 0.00378306 |  |  |  |
| WDR46 | 1.03106304 | 1.38723411 | 0.4277912 | 0.00378306 |  |  |  |
| ETV4 | 2.02786871 | 2.7286026 | 0.42807363 | 0.00179006 |  |  |  |
| EEF1A1 | 5.27479817 | 7.09790725 | 0.42825738 | 0.0011447 |  |  |  |
| CLK2 | 0.82718996 | 1.11364966 | 0.4286349 | 0.00823573 |  |  |  |
| GBA2 | 1.55411446 | 2.09211641 | 0.42869423 | 0.00335472 |  |  |  |
| NOL12 | 0.79711032 | 1.07341665 | 0.42897299 | 0.01901287 |  |  |  |
| RP1-37E16.12 | 0.79711032 | 1.07341665 | 0.42897299 | 0.01901287 |  |  |  |
| CFD | 0.9734104 | 1.3107914 | 0.42901006 | 0.00449275 |  |  |  |
| MAT2A | 4.16686496 | 5.61089528 | 0.42923078 | 0.00132726 |  |  |  |
| MED23 | 1.02939195 | 1.38723411 | 0.43012945 | 0.004014 |  |  |  |
| HNRNPAB | 2.09889007 | 2.82838046 | 0.43023028 | 0.00273969 |  |  |  |
| JUP | 1.41040065 | 1.90141195 | 0.43076659 | 0.00219423 |  |  |  |
| EFNA4 | 0.93998859 | 1.26733975 | 0.43076659 | 0.00537718 |  |  |  |
| RP11-540D14.8 | 0.93998859 | 1.26733975 | 0.43076659 | 0.00537718 |  |  |  |
| RSPH14 | 1.77302736 | 2.39064533 | 0.43103563 | 0.00472941 |  |  |  |
| RPS19 | 3.54271254 | 4.77807202 | 0.43152143 | 0.00146042 |  |  |  |
| FAM49B | 1.00599667 | 1.35746169 | 0.43198764 | 0.00322402 |  |  |  |
| LAG3 | 3.31544419 | 4.47391048 | 0.43227466 | 0.00156389 |  |  |  |
| PTMS | 3.31544419 | 4.47391048 | 0.43227466 | 0.00156389 |  |  |  |
| FAM214B | 4.40248877 | 5.94080595 | 0.43230548 | 0.00159876 |  |  |  |
| FAM46B | 5.49036889 | 7.42138064 | 0.43476592 | 0.0010872 |  |  |  |
| TRNP1 | 5.49036889 | 7.42138064 | 0.43476592 | 0.0010872 |  |  |  |
| KAT8 | 1.37113002 | 1.853937 | 0.43501851 | 0.00290655 |  |  |  |
| SLC16A3 | 3.17005929 | 4.28722932 | 0.43547083 | 0.00142921 |  |  |  |
| ACHE | 2.48073432 | 3.35623752 | 0.43598211 | 0.00263099 |  |  |  |
| UFSP1 | 2.48073432 | 3.35623752 | 0.43598211 | 0.00263099 |  |  |  |
| EFNB2 | 1.12213749 | 1.51839372 | 0.43603151 | 0.00334468 |  |  |  |
| ICT1 | 1.03273413 | 1.39769469 | 0.43628847 | 0.0066123 |  |  |  |
| GAPDH | 4.46264804 | 6.04058381 | 0.43675456 | 0.00097672 |  |  |  |
| ACTG1 | 5.95827432 | 8.06510876 | 0.4367856 | 0.00092997 |  |  |  |
| AL021546.6 | 0.89737577 | 1.2158415 | 0.43782736 | 0.00449275 |  |  |  |
| COX6A1 | 0.89737577 | 1.2158415 | 0.43782736 | 0.00449275 |  |  |  |
| RHEBL1 | 0.98677913 | 1.33734518 | 0.43826402 | 0.00364374 |  |  |  |
| FAM131A | 2.00865117 | 2.72216531 | 0.43839856 | 0.00157149 |  |  |  |
| PDE4A | 2.57013768 | 3.48417848 | 0.43888161 | 0.00148642 |  |  |  |
| ZNF540 | 1.06114267 | 1.43953702 | 0.43970144 | 0.00306393 |  |  |  |
| TUFT1 | 0.96756159 | 1.31320538 | 0.44035011 | 0.00533436 |  |  |  |
| NHLRC4 | 0.9475085 | 1.28665159 | 0.44108549 | 0.00625015 |  |  |  |
| PIGQ | 0.9475085 | 1.28665159 | 0.44108549 | 0.00625015 |  |  |  |
| TMEM102 | 1.08955121 | 1.47977003 | 0.44136184 | 0.00623355 |  |  |  |
| ZBTB26 | 1.20652757 | 1.63909274 | 0.44179044 | 0.01024653 |  |  |  |
| SLC29A1 | 1.28841102 | 1.7509405 | 0.44230923 | 0.00190943 |  |  |  |
| TMTC2 | 1.27587784 | 1.73404264 | 0.44241852 | 0.00167537 |  |  |  |
| SCML1 | 0.91492222 | 1.2440046 | 0.44293128 | 0.00256484 |  |  |  |
| ALOXE3 | 3.06143838 | 4.16411632 | 0.4437303 | 0.00108873 |  |  |  |
| HES7 | 3.06143838 | 4.16411632 | 0.4437303 | 0.00108873 |  |  |  |
| ILVBL | 1.43630256 | 1.95612884 | 0.44544055 | 0.00168995 |  |  |  |
| PYCRL | 1.87579945 | 2.554796 | 0.44555887 | 0.00433832 |  |  |  |
| PDCD10 | 2.02536207 | 2.76159366 | 0.44719099 | 0.00091744 |  |  |  |
| SERPINI1 | 2.02536207 | 2.76159366 | 0.44719099 | 0.00091744 |  |  |  |
| HMGN3 | 1.30762857 | 1.78312691 | 0.44723044 | 0.00157149 |  |  |  |
| KIF3A | 0.78875487 | 1.07663529 | 0.44847561 | 0.00404794 |  |  |  |
| FBXL15 | 1.73375673 | 2.36650552 | 0.44869669 | 0.00110995 |  |  |  |
| PSD | 1.73375673 | 2.36650552 | 0.44869669 | 0.00110995 |  |  |  |
| SLC9A1 | 1.62513582 | 2.21844806 | 0.44881663 | 0.00130621 |  |  |  |
| ATF3 | 4.08247487 | 5.57307625 | 0.44898745 | 0.0013588 |  |  |  |
| B3GALT4 | 0.96171277 | 1.31401004 | 0.44997315 | 0.00382643 |  |  |  |
| UNG | 1.36945893 | 1.87324885 | 0.45172071 | 0.00139542 |  |  |  |
| TBX6 | 1.50565283 | 2.06073466 | 0.45257696 | 0.00137972 |  |  |  |
| H1FX | 2.50663623 | 3.43268023 | 0.45348468 | 0.00102994 |  |  |  |
| VCAN | 1.88248381 | 2.57893581 | 0.45399279 | 0.00083581 |  |  |  |
| CBR1 | 0.90071795 | 1.23515334 | 0.45518924 | 0.00243961 |  |  |  |
| ATP6V1G2 | 1.39452529 | 1.9126772 | 0.4556065 | 0.00101251 |  |  |  |
| ATP6V1G2-DDX39B | 1.39452529 | 1.9126772 | 0.4556065 | 0.00101251 |  |  |  |
| NFKBIL1 | 1.39452529 | 1.9126772 | 0.4556065 | 0.00101251 |  |  |  |
| CHMP2A | 1.09874221 | 1.50712848 | 0.45566735 | 0.00185553 |  |  |  |
| PCOLCE | 0.98928577 | 1.35746169 | 0.45613373 | 0.00189842 |  |  |  |
| ECSIT | 1.46972438 | 2.01808768 | 0.45724293 | 0.0017586 |  |  |  |
| QTRT1 | 0.78374159 | 1.07663529 | 0.45766478 | 0.0156397 |  |  |  |
| FGF11 | 1.29091766 | 1.77427565 | 0.45859836 | 0.00273969 |  |  |  |
| TDRKH | 1.52654146 | 2.10096767 | 0.46059471 | 0.00115835 |  |  |  |
| ADAMTS10 | 3.00712793 | 4.14078117 | 0.46144076 | 0.00071006 |  |  |  |
| BAX | 0.8464075 | 1.16595257 | 0.46170113 | 0.00195689 |  |  |  |
| CD68 | 5.82291596 | 8.02246178 | 0.46228543 | 0.00028179 |  |  |  |
| EIF4A1 | 5.82291596 | 8.02246178 | 0.46228543 | 0.00028179 |  |  |  |
| MPDU1 | 5.82291596 | 8.02246178 | 0.46228543 | 0.00028179 |  |  |  |
| C11orf1 | 1.14553276 | 1.57874323 | 0.46248558 | 0.00228812 |  |  |  |
| FDXACB1 | 1.14553276 | 1.57874323 | 0.46248558 | 0.00228812 |  |  |  |
| RP11-108O10.8 | 1.14553276 | 1.57874323 | 0.46248558 | 0.00228812 |  |  |  |
| SYF2 | 1.00933886 | 1.39125741 | 0.46266368 | 0.00163857 |  |  |  |
| ZSCAN18 | 0.90406013 | 1.24802791 | 0.4648012 | 0.00156389 |  |  |  |
| ADNP2 | 1.28089111 | 1.76944769 | 0.46591093 | 0.00117805 |  |  |  |
| NUP107 | 0.85894068 | 1.18687373 | 0.46615562 | 0.00709139 |  |  |  |
| HMG20B | 2.07298816 | 2.86459016 | 0.4664852 | 0.00070702 |  |  |  |
| AXIN1 | 1.62346473 | 2.25063446 | 0.47107363 | 0.00062094 |  |  |  |
| RRAD | 0.88734923 | 1.23032538 | 0.47109594 | 0.03319655 |  |  |  |
| EMC3 | 0.78457714 | 1.08870519 | 0.47220096 | 0.0017081 |  |  |  |
| COL20A1 | 0.90489568 | 1.25607451 | 0.47273558 | 0.00853339 |  |  |  |
| CCNL1 | 4.18274032 | 5.80803702 | 0.47355843 | 0.00031235 |  |  |  |
| LRRC23 | 3.90116152 | 5.42099548 | 0.47460376 | 0.00027046 |  |  |  |
| DERL3 | 1.50063956 | 2.08728845 | 0.47585001 | 0.00107636 |  |  |  |
| ATF7IP | 1.21404748 | 1.68898167 | 0.47606794 | 0.00412653 |  |  |  |
| WDR38 | 0.93915304 | 1.30757276 | 0.47710814 | 0.0057078 |  |  |  |
| VASN | 4.98987719 | 6.94904512 | 0.47778045 | 0.00023746 |  |  |  |
| C17orf53 | 0.78541269 | 1.09433781 | 0.47810632 | 0.01583522 |  |  |  |
| SMARCD3 | 1.79809372 | 2.50651639 | 0.4790528 | 0.00058568 |  |  |  |
| NFKBIZ | 6.83392591 | 9.53683219 | 0.48078539 | 0.00020647 |  |  |  |
| POC5 | 0.96254831 | 1.34378246 | 0.4810251 | 0.00080739 |  |  |  |
| IRX4 | 0.92327768 | 1.28906557 | 0.48112892 | 0.00897787 |  |  |  |
| CYB5R1 | 0.86646059 | 1.21101354 | 0.48262121 | 0.00122975 |  |  |  |
| RPL10 | 2.19999107 | 3.07460646 | 0.48277832 | 0.00062197 |  |  |  |
| RAB36 | 1.34021484 | 1.87324885 | 0.48284323 | 0.00141723 |  |  |  |
| MXD3 | 1.42794711 | 1.99636185 | 0.48321267 | 0.00067139 |  |  |  |
| NLRP1 | 1.00850331 | 1.41137392 | 0.48455646 | 0.00088141 |  |  |  |
| PCBP3 | 1.67777518 | 2.34799834 | 0.48470253 | 0.00034704 |  |  |  |
| GCAT | 3.67222208 | 5.14580171 | 0.48668444 | 0.00025294 |  |  |  |
| H1F0 | 3.67222208 | 5.14580171 | 0.48668444 | 0.00025294 |  |  |  |
| SLC2A1 | 3.49759309 | 4.90279434 | 0.48717847 | 0.00063963 |  |  |  |
| FLOT1 | 2.88430276 | 4.04502661 | 0.4878402 | 0.0001943 |  |  |  |
| IER3 | 2.88430276 | 4.04502661 | 0.4878402 | 0.0001943 |  |  |  |
| EHD3 | 0.9475085 | 1.32929858 | 0.48810031 | 0.00633156 |  |  |  |
| IPO11 | 0.89403359 | 1.25446519 | 0.48829343 | 0.00130621 |  |  |  |
| COMMD4 | 0.72358233 | 1.01628578 | 0.48959845 | 0.00262675 |  |  |  |
| C11orf91 | 1.52069265 | 2.13556806 | 0.48968682 | 0.00030718 |  |  |  |
| CA11 | 1.02772085 | 1.44356032 | 0.48985897 | 0.00230204 |  |  |  |
| C1orf35 | 0.99095686 | 1.39206207 | 0.48999167 | 0.00083642 |  |  |  |
| DNTTIP2 | 1.55578555 | 2.18545699 | 0.49009278 | 0.00079762 |  |  |  |
| RPL10A | 2.48491205 | 3.49061577 | 0.4901788 | 0.00044098 |  |  |  |
| LIMD2 | 1.22741621 | 1.72599604 | 0.49153981 | 0.00117362 |  |  |  |
| HSP90AB1 | 1.67276191 | 2.3528263 | 0.49198009 | 0.00069796 |  |  |  |
| SLC4A1AP | 0.73110223 | 1.02916034 | 0.49284692 | 0.00224278 |  |  |  |
| SUPT7L | 0.73110223 | 1.02916034 | 0.49284692 | 0.00224278 |  |  |  |
| SPSB2 | 3.16253938 | 4.45137999 | 0.4930934 | 0.00018076 |  |  |  |
| TPI1 | 3.16253938 | 4.45137999 | 0.4930934 | 0.00018076 |  |  |  |
| CCDC146 | 1.44048029 | 2.0277436 | 0.49310564 | 0.00050399 |  |  |  |
| SMPD3 | 1.63182019 | 2.29730475 | 0.4932714 | 0.00025951 |  |  |  |
| RSRC1 | 0.84724305 | 1.19331101 | 0.49371725 | 0.00076181 |  |  |  |
| ITGA7 | 0.79209705 | 1.11606364 | 0.49423346 | 0.00126975 |  |  |  |
| NR1H2 | 0.99346349 | 1.40091334 | 0.49548893 | 0.00046127 |  |  |  |
| USP14 | 1.0285564 | 1.45080226 | 0.49590297 | 0.00071637 |  |  |  |
| INS | 0.97424595 | 1.37435955 | 0.49605372 | 0.00457756 |  |  |  |
| CKAP2L | 1.42544047 | 2.01084573 | 0.49617092 | 0.00025147 |  |  |  |
| FRMD5 | 0.80295914 | 1.1369848 | 0.50137921 | 0.00072762 |  |  |  |
| CPNE8 | 1.0009834 | 1.41861586 | 0.50272479 | 0.00048822 |  |  |  |
| SQSTM1 | 3.3229641 | 4.7096759 | 0.50308535 | 0.00014993 |  |  |  |
| ZNF90 | 0.81967005 | 1.16353859 | 0.50497588 | 0.00067253 |  |  |  |
| E2F2 | 0.76786623 | 1.09031451 | 0.5053573 | 0.00097185 |  |  |  |
| POLQ | 0.84306532 | 1.19733432 | 0.50569435 | 0.00049181 |  |  |  |
| STMN3 | 0.7102136 | 1.00904384 | 0.50616221 | 0.00071364 |  |  |  |
| BTBD19 | 6.71193628 | 9.53683219 | 0.50676777 | 6.3134E-05 |  |  |  |
| PLK3 | 6.71193628 | 9.53683219 | 0.50676777 | 6.3134E-05 |  |  |  |
| RP11-269F19.9 | 6.71193628 | 9.53683219 | 0.50676777 | 6.3134E-05 |  |  |  |
| MIS18A | 0.71773351 | 1.02030908 | 0.50698939 | 0.00819195 |  |  |  |
| CEP112 | 0.86144732 | 1.22469276 | 0.50717886 | 0.00153646 |  |  |  |
| AC040160.13 | 1.44131583 | 2.04946942 | 0.5076387 | 0.00020647 |  |  |  |
| TMEM208 | 1.44131583 | 2.04946942 | 0.5076387 | 0.00020647 |  |  |  |
| TPM1 | 9.20687488 | 13.0990627 | 0.50868236 | 5.5467E-05 |  |  |  |
| EGR3 | 3.86523307 | 5.5014615 | 0.50920321 | 0.00010819 |  |  |  |
| LINGO1 | 0.95586395 | 1.36148499 | 0.50994026 | 0.00082933 |  |  |  |
| SERPINE1 | 9.03976579 | 12.8809998 | 0.51088858 | 8.3141E-05 |  |  |  |
| CHADL | 0.93581086 | 1.33412654 | 0.51123349 | 0.00108382 |  |  |  |
| EFNA5 | 1.51818601 | 2.17097311 | 0.51577866 | 0.00017416 |  |  |  |
| DKK1 | 0.74029323 | 1.05893277 | 0.51595522 | 0.00091744 |  |  |  |
| CDC23 | 1.16725694 | 1.67127915 | 0.51753718 | 0.00019423 |  |  |  |
| PTMA | 5.51459971 | 7.90417673 | 0.51933185 | 3.7427E-05 |  |  |  |
| VPS13A | 1.70367709 | 2.44294824 | 0.51978364 | 0.00012585 |  |  |  |
| FAM173A | 0.76285296 | 1.09433781 | 0.52010688 | 0.0039966 |  |  |  |
| PYGO2 | 0.99680567 | 1.4298811 | 0.52015837 | 0.00029404 |  |  |  |
| DISC1 | 0.91158004 | 1.30837742 | 0.52094728 | 0.0005956 |  |  |  |
| PARD6B | 0.8096435 | 1.16273393 | 0.52171678 | 0.00031931 |  |  |  |
| CWC25 | 0.72776005 | 1.04525355 | 0.52181747 | 0.00046256 |  |  |  |
| PRDM7 | 1.10291994 | 1.58437585 | 0.52227105 | 0.00026699 |  |  |  |
| MPP2 | 1.97105162 | 2.83320842 | 0.52331393 | 9.7326E-05 |  |  |  |
| GEMIN6 | 4.19026023 | 6.03495119 | 0.52625148 | 6.3197E-05 |  |  |  |
| TIMM10 | 0.77705723 | 1.12008694 | 0.52704807 | 0.000287 |  |  |  |
| ZSCAN22 | 1.51233719 | 2.17982437 | 0.52721219 | 0.0005956 |  |  |  |
| COG7 | 0.80880796 | 1.16675723 | 0.52818483 | 0.00188247 |  |  |  |
| WIPF2 | 0.82050559 | 1.18365509 | 0.52822023 | 0.00037762 |  |  |  |
| FMO4 | 0.71689796 | 1.03479296 | 0.5289879 | 0.0007619 |  |  |  |
| ADAMTS1 | 8.01622267 | 11.5669898 | 0.52901349 | 2.7452E-05 |  |  |  |
| SETDB1 | 0.84306532 | 1.21745082 | 0.5297158 | 0.00027656 |  |  |  |
| SPAG4 | 3.05475402 | 4.41275631 | 0.53053688 | 3.6819E-05 |  |  |  |
| P4HB | 4.22702423 | 6.12185448 | 0.53427536 | 3.2209E-05 |  |  |  |
| DPF1 | 1.52988365 | 2.21603408 | 0.5343387 | 9.0795E-05 |  |  |  |
| SLC1A1 | 1.04526731 | 1.51437042 | 0.53450432 | 0.00015667 |  |  |  |
| DUSP6 | 3.52934381 | 5.11281064 | 0.53464667 | 5.0398E-05 |  |  |  |
| RND2 | 1.06364931 | 1.5449475 | 0.53819813 | 0.00117362 |  |  |  |
| DLG1 | 1.01602322 | 1.47735605 | 0.53972834 | 0.00207294 |  |  |  |
| NUDT22 | 0.86144732 | 1.25285587 | 0.53995815 | 0.00028179 |  |  |  |
| TRPT1 | 0.86144732 | 1.25285587 | 0.53995815 | 0.00028179 |  |  |  |
| PPFIA3 | 2.73641122 | 3.9806538 | 0.54061603 | 3.569E-05 |  |  |  |
| CALR | 5.14111091 | 7.49299539 | 0.54342742 | 2.7452E-05 |  |  |  |
| GADD45GIP1 | 5.14111091 | 7.49299539 | 0.54342742 | 2.7452E-05 |  |  |  |
| RAD23A | 5.14111091 | 7.49299539 | 0.54342742 | 2.7452E-05 |  |  |  |
| LDLRAD2 | 0.97090377 | 1.41539722 | 0.54343022 | 0.00036189 |  |  |  |
| AIP | 1.26835793 | 1.85152302 | 0.54547185 | 8.2587E-05 |  |  |  |
| KCNN3 | 0.87230941 | 1.27377703 | 0.54577528 | 0.0002503 |  |  |  |
| HAPLN3 | 1.09707112 | 1.60368769 | 0.54740602 | 0.00048322 |  |  |  |
| PELI1 | 0.98677913 | 1.44275566 | 0.54765574 | 7.7401E-05 |  |  |  |
| CKB | 1.58502964 | 2.32224922 | 0.550797 | 9.6946E-05 |  |  |  |
| DNAJC4 | 1.19817212 | 1.75657312 | 0.551628 | 8.0296E-05 |  |  |  |
| VEGFB | 1.19817212 | 1.75657312 | 0.551628 | 8.0296E-05 |  |  |  |
| LBH | 4.08163933 | 5.99310886 | 0.55409878 | 1.8911E-05 |  |  |  |
| PIM2 | 0.81131459 | 1.19170169 | 0.55422311 | 0.00012692 |  |  |  |
| HIST1H3E | 1.36611675 | 2.01004107 | 0.55688404 | 4.8461E-05 |  |  |  |
| FGFR4 | 0.68013396 | 1.00099724 | 0.55697966 | 0.00033971 |  |  |  |
| SAP25 | 0.71272023 | 1.05008151 | 0.55855281 | 0.00102588 |  |  |  |
| MAATS1 | 0.68765387 | 1.0138718 | 0.55955792 | 0.00098718 |  |  |  |
| KCTD21 | 1.86159517 | 2.7527424 | 0.56414903 | 1.9459E-05 |  |  |  |
| USP35 | 1.86159517 | 2.7527424 | 0.56414903 | 1.9459E-05 |  |  |  |
| ISYNA1 | 1.61678037 | 2.39708261 | 0.56793926 | 0.00037871 |  |  |  |
| ARL5C | 0.67762733 | 1.00502054 | 0.56808235 | 0.01840719 |  |  |  |
| CACNA1G | 1.36862338 | 2.03418088 | 0.57145707 | 4.036E-05 |  |  |  |
| VPS37C | 1.08620903 | 1.6157576 | 0.57256249 | 2.8631E-05 |  |  |  |
| PCDH10 | 0.77204396 | 1.14985937 | 0.57419576 | 0.00224663 |  |  |  |
| SLC2A4 | 1.10542658 | 1.647944 | 0.57572275 | 0.00121665 |  |  |  |
| SLC4A8 | 1.32935275 | 1.98590127 | 0.57879238 | 0.00189676 |  |  |  |
| RAB37 | 0.7469776 | 1.1168683 | 0.5797929 | 0.00010535 |  |  |  |
| LA16c-306E5.2 | 0.97675258 | 1.46287217 | 0.5823429 | 2.7452E-05 |  |  |  |
| C17orf100 | 1.23159393 | 1.84669506 | 0.58411357 | 2.405E-05 |  |  |  |
| MED31 | 1.23159393 | 1.84669506 | 0.58411357 | 2.405E-05 |  |  |  |
| PROCR | 0.68514724 | 1.02755102 | 0.58413959 | 0.00105994 |  |  |  |
| KCNIP2 | 1.13383512 | 1.70024691 | 0.58419778 | 0.00021352 |  |  |  |
| KIAA1671 | 1.04192513 | 1.56425935 | 0.58585811 | 7.0783E-05 |  |  |  |
| RSPH3 | 1.05780049 | 1.58920381 | 0.58687231 | 2.2956E-05 |  |  |  |
| CHRM4 | 1.82316009 | 2.7406725 | 0.5878957 | 1.0224E-05 |  |  |  |
| MARCKSL1 | 1.19900766 | 1.80646205 | 0.59100806 | 2.9908E-05 |  |  |  |
| GGN | 2.52836041 | 3.81408915 | 0.5930086 | 3.9024E-06 |  |  |  |
| SPRED3 | 2.52836041 | 3.81408915 | 0.5930086 | 3.9024E-06 |  |  |  |
| ADGRL1 | 0.84139423 | 1.27216771 | 0.59595985 | 5.9754E-05 |  |  |  |
| DNAAF3 | 1.21237639 | 1.83542982 | 0.59796322 | 1.148E-05 |  |  |  |
| KIAA1161 | 0.93079759 | 1.41459256 | 0.60341766 | 2.0025E-05 |  |  |  |
| PTPN1 | 2.37127787 | 3.60648683 | 0.60478753 | 2.5049E-06 |  |  |  |
| CRIP2 | 1.18396785 | 1.80324341 | 0.60663463 | 2.4638E-05 |  |  |  |
| KAT5 | 1.47891538 | 2.2538531 | 0.60759863 | 5.4793E-06 |  |  |  |
| EML2 | 2.34370488 | 3.57590974 | 0.60937429 | 2.8895E-06 |  |  |  |
| SLC27A5 | 1.16976357 | 1.78554089 | 0.60980861 | 9.0596E-06 |  |  |  |
| HSD17B14 | 0.85894068 | 1.31642402 | 0.61551722 | 1.5961E-05 |  |  |  |
| SOX9 | 0.73026669 | 1.12008694 | 0.61654622 | 3.3319E-05 |  |  |  |
| ASNA1 | 1.60926046 | 2.4678927 | 0.6166478 | 1.7717E-06 |  |  |  |
| RTN2 | 2.13231189 | 3.27255286 | 0.61783203 | 4.5304E-06 |  |  |  |
| SHKBP1 | 0.79878141 | 1.22630208 | 0.6179243 | 1.1005E-05 |  |  |  |
| ADAMTS15 | 1.02354313 | 1.57552459 | 0.6218643 | 5.2592E-06 |  |  |  |
| EPHB1 | 0.78875487 | 1.21503684 | 0.62282331 | 6.9635E-05 |  |  |  |
| TRIM7 | 1.24162048 | 1.9126772 | 0.62304995 | 3.571E-06 |  |  |  |
| PBXIP1 | 0.8923625 | 1.37677353 | 0.62512673 | 8.4639E-06 |  |  |  |
| FAM83H | 0.78708378 | 1.21503684 | 0.62587987 | 0.00010657 |  |  |  |
| RP11-429J17.8 | 0.78708378 | 1.21503684 | 0.62587987 | 0.00010657 |  |  |  |
| TLE6 | 0.65088987 | 1.00502054 | 0.62608795 | 0.0105318 |  |  |  |
| SRSF7 | 3.48840209 | 5.38559044 | 0.62645272 | 1.7083E-06 |  |  |  |
| FCHO1 | 0.69517378 | 1.07422131 | 0.62723749 | 1.178E-05 |  |  |  |
| PLTP | 1.56414101 | 2.41719911 | 0.62771998 | 6.0115E-06 |  |  |  |
| NACAD | 0.66091642 | 1.0219184 | 0.62809758 | 2.0111E-05 |  |  |  |
| CGB7 | 0.76034632 | 1.17721781 | 0.63009848 | 2.4147E-05 |  |  |  |
| PHLDA1 | 3.87442407 | 5.99713216 | 0.63021788 | 5.7959E-07 |  |  |  |
| GPR63 | 0.68347615 | 1.06134675 | 0.63430846 | 1.4303E-05 |  |  |  |
| ATP6V1F | 7.81903395 | 12.151173 | 0.63601921 | 3.226E-07 |  |  |  |
| FLNC | 7.81903395 | 12.151173 | 0.63601921 | 3.226E-07 |  |  |  |
| RP11-309L24.4 | 7.81903395 | 12.151173 | 0.63601921 | 3.226E-07 |  |  |  |
| CUL9 | 0.9107445 | 1.41700654 | 0.63726871 | 9.6384E-06 |  |  |  |
| CCDC163P | 1.16558585 | 1.81450866 | 0.63817401 | 5.5812E-06 |  |  |  |
| MMACHC | 1.16558585 | 1.81450866 | 0.63817401 | 5.5812E-06 |  |  |  |
| NQO1 | 1.56246992 | 2.43329232 | 0.63883147 | 2.2168E-06 |  |  |  |
| CRB2 | 1.47640874 | 2.30615601 | 0.64312951 | 5.0362E-06 |  |  |  |
| CDKN1A | 6.62921728 | 10.3551716 | 0.64341581 | 1.3175E-07 |  |  |  |
| BCL6B | 0.8004525 | 1.25607451 | 0.64949762 | 5.364E-06 |  |  |  |
| SH2D3C | 1.38449874 | 2.17741039 | 0.65295676 | 9.02E-07 |  |  |  |
| PRSS27 | 0.79042596 | 1.2440046 | 0.65374275 | 2.9015E-06 |  |  |  |
| C19orf60 | 1.14135503 | 1.79761079 | 0.65496747 | 2.4894E-06 |  |  |  |
| CDH15 | 0.81465677 | 1.28343295 | 0.65521348 | 4.1551E-06 |  |  |  |
| POU3F2 | 1.04860949 | 1.65518595 | 0.6581124 | 1.2253E-06 |  |  |  |
| TNFSF9 | 0.73277332 | 1.15951529 | 0.6614842 | 7.2071E-05 |  |  |  |
| PPM1N | 1.58085192 | 2.50490707 | 0.66380042 | 1.3656E-06 |  |  |  |
| ALK | 0.82886105 | 1.31401004 | 0.66424824 | 1.772E-06 |  |  |  |
| ASPHD1 | 1.45886229 | 2.31822592 | 0.66789661 | 2.311E-07 |  |  |  |
| SEZ6L2 | 1.45886229 | 2.31822592 | 0.66789661 | 2.311E-07 |  |  |  |
| ADM | 5.21798109 | 8.29202293 | 0.66818591 | 5.312E-08 |  |  |  |
| GRAMD2 | 0.92829095 | 1.47735605 | 0.66990072 | 7.4017E-06 |  |  |  |
| BCL11A | 0.81131459 | 1.29308887 | 0.67194507 | 1.3704E-06 |  |  |  |
| DHPS | 1.2474693 | 1.98992457 | 0.67337124 | 4.7756E-06 |  |  |  |
| PPM1J | 1.47055992 | 2.3568496 | 0.68021164 | 5.7143E-07 |  |  |  |
| RADIL | 0.6458766 | 1.03559762 | 0.68043301 | 3.2209E-05 |  |  |  |
| SARDH | 0.67094296 | 1.07583063 | 0.68051659 | 0.00078605 |  |  |  |
| VCL | 4.82778138 | 7.747268 | 0.68227199 | 2.4622E-08 |  |  |  |
| NKX6-1 | 0.98093031 | 1.57713391 | 0.68463466 | 2.2515E-05 |  |  |  |
| RNF123 | 1.19482994 | 1.92233312 | 0.68569283 | 8.2849E-07 |  |  |  |
| VGF | 1.83652881 | 2.96034472 | 0.68856405 | 1.6296E-07 |  |  |  |
| ZBTB37 | 2.27519015 | 3.667641 | 0.68869659 | 6.7282E-08 |  |  |  |
| DNAH2 | 0.84222977 | 1.35907101 | 0.68980296 | 5.564E-07 |  |  |  |
| GADD45G | 0.95920613 | 1.55138478 | 0.69318023 | 1.2316E-06 |  |  |  |
| MST1 | 1.16224367 | 1.87968613 | 0.69320441 | 6.8747E-07 |  |  |  |
| HCN2 | 1.27922002 | 2.07119525 | 0.69486202 | 2.3191E-07 |  |  |  |
| AGO1 | 0.96004168 | 1.55943139 | 0.69938483 | 8.0345E-07 |  |  |  |
| AGAP2 | 0.86980277 | 1.41620188 | 0.70274552 | 1.1269E-06 |  |  |  |
| ZNF274 | 1.16140812 | 1.91026322 | 0.71751098 | 6.1171E-08 |  |  |  |
| KLHL26 | 1.29425984 | 2.1387867 | 0.72432145 | 6.6325E-07 |  |  |  |
| PABPC4 | 2.46987223 | 4.08284564 | 0.72497946 | 3.8534E-08 |  |  |  |
| SLC35B2 | 1.47807983 | 2.44616688 | 0.72650234 | 1.4397E-07 |  |  |  |
| FAM69B | 1.02604976 | 1.70990284 | 0.73636257 | 4.0113E-06 |  |  |  |
| HSPA8 | 4.12007442 | 6.87662571 | 0.73895121 | 2.8297E-09 |  |  |  |
| SLC6A8 | 0.75449751 | 1.26090247 | 0.74023677 | 6.6325E-07 |  |  |  |
| SMIM1 | 0.94917959 | 1.58678983 | 0.74086459 | 8.1088E-08 |  |  |  |
| NEK2 | 0.81632787 | 1.36792227 | 0.74418265 | 8.5818E-08 |  |  |  |
| FSCN1 | 4.35235604 | 7.30792356 | 0.74759139 | 4.8391E-09 |  |  |  |
| PRUNE2 | 0.62582351 | 1.05571413 | 0.7536103 | 5.3656E-05 |  |  |  |
| RP11-106M3.5 | 0.6918316 | 1.17238985 | 0.7602526 | 7.7886E-08 |  |  |  |
| OLFM1 | 0.66008087 | 1.1208916 | 0.76318685 | 3.8356E-05 |  |  |  |
| SNX22 | 1.96353171 | 3.34014432 | 0.76623779 | 1.4122E-08 |  |  |  |
| CD44 | 6.44957502 | 10.9771739 | 0.76719405 | 1.9416E-10 |  |  |  |
| SURF1 | 0.9550284 | 1.63024148 | 0.77096492 | 1.669E-08 |  |  |  |
| SURF2 | 0.9550284 | 1.63024148 | 0.77096492 | 1.669E-08 |  |  |  |
| SESN3 | 1.2917532 | 2.20798747 | 0.77303884 | 1.4619E-09 |  |  |  |
| ZNFX1 | 3.47252672 | 5.95528983 | 0.77807827 | 3.771E-10 |  |  |  |
| OCA2 | 0.80212359 | 1.38321081 | 0.78550611 | 1.2605E-08 |  |  |  |
| APC2 | 0.86061177 | 1.50149585 | 0.80238348 | 6.2245E-09 |  |  |  |
| PEG10 | 1.28506884 | 2.24339252 | 0.80346059 | 9.0281E-10 |  |  |  |
| SGCE | 1.28506884 | 2.24339252 | 0.80346059 | 9.0281E-10 |  |  |  |
| CILP2 | 0.72024014 | 1.26573043 | 0.81270896 | 3.0153E-08 |  |  |  |
| AMOT | 0.57736188 | 1.01548112 | 0.81371658 | 2.1563E-08 |  |  |  |
| CELF6 | 0.81465677 | 1.43470906 | 0.81586845 | 1.9006E-09 |  |  |  |
| DNAJA1 | 3.96466297 | 6.98445017 | 0.81685503 | 4.4043E-11 |  |  |  |
| CERS1 | 0.70018705 | 1.23515334 | 0.81814138 | 1.8335E-08 |  |  |  |
| GDF1 | 0.70018705 | 1.23515334 | 0.81814138 | 1.8335E-08 |  |  |  |
| RUNDC3A | 0.93413977 | 1.66242789 | 0.83103424 | 2.6703E-10 |  |  |  |
| SLC17A7 | 1.83151554 | 3.26209228 | 0.83250199 | 5.7252E-11 |  |  |  |
| SEC11C | 1.05111613 | 1.87324885 | 0.83313824 | 6.9029E-10 |  |  |  |
| ZNF385A | 0.62665906 | 1.11767296 | 0.83390595 | 3.6088E-08 |  |  |  |
| EPOR | 1.55327892 | 2.7728589 | 0.83574456 | 2.8762E-11 |  |  |  |
| PACRG | 0.69350269 | 1.24239528 | 0.84039234 | 5.3361E-09 |  |  |  |
| PARK2 | 0.69350269 | 1.24239528 | 0.84039234 | 5.3361E-09 |  |  |  |
| GRASP | 1.35274802 | 2.42283174 | 0.84043384 | 7.4689E-11 |  |  |  |
| HIST1H1D | 0.83053214 | 1.49425391 | 0.84668796 | 5.3166E-10 |  |  |  |
| BTG2 | 3.23188965 | 5.84183275 | 0.85391341 | 4.8382E-12 |  |  |  |
| KANK3 | 0.65088987 | 1.18124111 | 0.85899425 | 2.2207E-09 |  |  |  |
| GDNF | 4.35987595 | 7.92026994 | 0.86117714 | 2.2328E-12 |  |  |  |
| NME9 | 0.54978888 | 1.00502054 | 0.86928361 | 2.066E-09 |  |  |  |
| SLC2A3 | 2.11309434 | 3.86236876 | 0.86990344 | 2.4684E-12 |  |  |  |
| RCC1 | 4.24874841 | 7.78508703 | 0.87358519 | 5.0388E-13 |  |  |  |
| ICAM4 | 2.3203096 | 4.26308952 | 0.87737941 | 8.9202E-13 |  |  |  |
| ICAM5 | 2.3203096 | 4.26308952 | 0.87737941 | 8.9202E-13 |  |  |  |
| F3 | 1.89251035 | 3.49463907 | 0.88458213 | 1.5576E-11 |  |  |  |
| IGFBP5 | 8.21174029 | 15.4414284 | 0.91101922 | 9.3832E-15 |  |  |  |
| ELAVL3 | 1.02437867 | 1.93440302 | 0.91660753 | 2.6566E-12 |  |  |  |
| HSPBP1 | 0.79878141 | 1.52965896 | 0.9366323 | 2.1739E-12 |  |  |  |
| FTL | 3.40902527 | 6.55878495 | 0.94393723 | 5.774E-15 |  |  |  |
| ARC | 0.90238904 | 1.7509405 | 0.95568077 | 1.0355E-11 |  |  |  |
| TXNRD1 | 3.98972933 | 7.78025907 | 0.96341894 | 8.4988E-16 |  |  |  |
| COMP | 0.72692451 | 1.43068576 | 0.97602777 | 1.089E-12 |  |  |  |
| SLC35F2 | 0.91742886 | 1.81933662 | 0.98711343 | 4.5457E-14 |  |  |  |
| SAT1 | 1.35859684 | 2.72699328 | 1.00477691 | 2.8614E-15 |  |  |  |
| XKR6 | 0.78959041 | 1.58839915 | 1.00764645 | 8.2225E-15 |  |  |  |
| PPP1R36 | 0.79711032 | 1.60610168 | 1.00996758 | 3.2663E-12 |  |  |  |
| CAMK2N2 | 0.7728795 | 1.55862673 | 1.01119036 | 1.5178E-10 |  |  |  |
| EID3 | 3.67472871 | 7.40850607 | 1.01141812 | 3.3675E-17 |  |  |  |
| SYT5 | 0.67846287 | 1.37275023 | 1.01584169 | 7.4338E-14 |  |  |  |
| CSF1 | 2.44062814 | 4.97762774 | 1.02798974 | 4.4311E-16 |  |  |  |
| NDRG4 | 0.80546578 | 1.64553002 | 1.02991118 | 3.5186E-15 |  |  |  |
| NOVA1 | 0.51386043 | 1.06215141 | 1.04633409 | 1.7593E-13 |  |  |  |
| GPR148 | 0.53558461 | 1.10801704 | 1.04763657 | 1.8929E-13 |  |  |  |
| GIPR | 0.96171277 | 2.0156737 | 1.06695158 | 3.1677E-16 |  |  |  |
| UCHL1 | 1.63599791 | 3.4857878 | 1.09095472 | 3.3675E-17 |  |  |  |
| PITX2 | 0.56733533 | 1.22710674 | 1.11185419 | 4.8332E-16 |  |  |  |
| GDF15 | 0.68180505 | 1.47735605 | 1.11464953 | 2.4121E-13 |  |  |  |
| RGL3 | 0.49714952 | 1.09433781 | 1.13698872 | 9.2067E-16 |  |  |  |
| CADM1 | 0.53057134 | 1.17802247 | 1.14950788 | 1.6125E-16 |  |  |  |
| CHAT | 0.46623434 | 1.04847219 | 1.16772957 | 4.8036E-16 |  |  |  |
| SLC18A3 | 0.46623434 | 1.04847219 | 1.16772957 | 4.8036E-16 |  |  |  |
| RPL3 | 4.42254186 | 10.055838 | 1.18497303 | 4.201E-23 |  |  |  |
| PAIP2B | 0.96087722 | 2.19994087 | 1.19435888 | 1.4532E-19 |  |  |  |
| ZDHHC12 | 1.0745114 | 2.47593931 | 1.20368706 | 9.402E-20 |  |  |  |
| PEG3 | 0.53976234 | 1.24480926 | 1.20427374 | 2.3077E-14 |  |  |  |
| ZIM2 | 0.53976234 | 1.24480926 | 1.20427374 | 2.3077E-14 |  |  |  |
| FBXL16 | 0.59908606 | 1.39206207 | 1.21525586 | 2.281E-16 |  |  |  |
| TRIM9 | 0.54143343 | 1.26090247 | 1.21834106 | 4.861E-19 |  |  |  |
| CPLX1 | 0.55229552 | 1.37757819 | 1.31732791 | 2.7973E-18 |  |  |  |
| RAB39A | 0.50466943 | 1.26009781 | 1.31870402 | 1.6907E-21 |  |  |  |
| SNAP25 | 0.5924017 | 1.49183993 | 1.33123414 | 3.4096E-22 |  |  |  |
| STAC2 | 0.39688407 | 1.04364422 | 1.39296279 | 2.1931E-17 |  |  |  |
| BCL11B | 0.61746806 | 1.63346012 | 1.40229895 | 2.9825E-25 |  |  |  |
| TIMP3 | 3.83849561 | 10.3616089 | 1.43247767 | 3.1132E-31 |  |  |  |
| EPS8L1 | 0.8923625 | 2.41639445 | 1.43632884 | 2.6237E-21 |  |  |  |
| CCND2 | 0.46289216 | 1.28423761 | 1.47051052 | 2.0476E-23 |  |  |  |
| SDC4 | 3.69645289 | 10.2819475 | 1.47573147 | 2.5843E-34 |  |  |  |
| SCRT1 | 0.87230941 | 2.48639989 | 1.5102766 | 5.3413E-26 |  |  |  |
| CDK5R2 | 0.80630132 | 2.40995717 | 1.57864841 | 5.6169E-33 |  |  |  |
| CACNG7 | 0.40941725 | 1.23113004 | 1.58638524 | 2.8865E-30 |  |  |  |
| PRDX1 | 2.84586767 | 8.65894796 | 1.60507824 | 6.0473E-41 |  |  |  |
| LMTK3 | 0.46706988 | 1.47252809 | 1.65483894 | 4.8471E-34 |  |  |  |
| CTD-2587H24.4 | 0.43197698 | 1.37194557 | 1.66529953 | 7.5626E-33 |  |  |  |
| CRMP1 | 0.39939071 | 1.29389353 | 1.69377265 | 1.0231E-32 |  |  |  |
| APLP1 | 0.59323724 | 1.93601234 | 1.70502056 | 6.4993E-38 |  |  |  |
| MUC12 | 0.29745417 | 1.02916034 | 1.78785372 | 4.8008E-30 |  |  |  |
| PTPRN | 0.31416507 | 1.10640772 | 1.81354993 | 7.839E-36 |  |  |  |
| HIF3A | 0.28575653 | 1.0420349 | 1.86349112 | 4.2899E-35 |  |  |  |
| KCNC3 | 0.95001513 | 3.53165343 | 1.89342627 | 6.1928E-51 |  |  |  |
| MAST1 | 0.61329033 | 2.28603951 | 1.89679628 | 1.4561E-46 |  |  |  |
| CTD-2207O23.12 | 0.51469597 | 1.97383137 | 1.93749874 | 6.4186E-46 |  |  |  |
| RTBDN | 0.51469597 | 2.05107874 | 1.99286074 | 2.3376E-49 |  |  |  |
| SLC12A5 | 0.12198963 | 1.22147412 | 3.31493969 | 1.7807E-86 |  |  |  |
