# Supplementary material for: Epigenetic and Metabolic Reprogramming of Fibroblasts in Crohn’s Disease Strictures Reveals Histone Deacetylases as Therapeutic Targets
Source: J Crohns Colitis. 2023 Dec 9;18(6):895–907. doi: 10.1093/ecco-jcc/jjad209 (PMC11147807; doi:10.1093/ecco-jcc/jjad209)
Supplement: jjad209_suppl_Supplementary_Tables_8 [file jjad209_suppl_supplementary_tables_8.docx]

**Supplementary Table 8. Enriched pathways associated with genes with increased and decreased promoter accessibility (scAssay for Transposase-Accessible Chromatin using sequencing)**

| **Enriched pathways associated with genes with increased promoter accessibility** | | | | |
| --- | --- | --- | --- | --- |
| Term | P-value | Adjusted P-value | Odds Ratio | Combined Score |
| TNF-alpha Signaling via NF-kB | 7.54E-06 | 3.77E-04 | 3.03637432 | 35.81397375 |
| UV Response Up | 1.94E-05 | 4.86E-04 | 3.21840834 | 34.91376828 |
| Coagulation | 1.13E-04 | 0.001876857 | 3.11186885 | 28.29176624 |
| mTORC1 Signaling | 5.09E-04 | 0.006357781 | 2.4619883 | 18.67123788 |
| Apoptosis | 7.07E-04 | 0.007071849 | 2.61166958 | 18.94562159 |
| Hypoxia | 0.00128514 | 0.009179575 | 2.32312697 | 15.46479418 |
| p53 Pathway | 0.00128514 | 0.009179575 | 2.32312697 | 15.46479418 |
| Apical Junction | 0.00307709 | 0.019231843 | 2.18609512 | 12.64386988 |
| Hedgehog Signaling | 0.0043613 | 0.024229439 | 4.39470656 | 23.8851656 |
| Mitotic Spindle | 0.00663777 | 0.02488767 | 2.06223904 | 10.34208479 |
| Myogenesis | 0.00696855 | 0.02488767 | 2.05086171 | 10.18529386 |
| E2F Targets | 0.00696855 | 0.02488767 | 2.05086171 | 10.18529386 |
| Epithelial Mesenchymal Transition | 0.00696855 | 0.02488767 | 2.05086171 | 10.18529386 |
| Inflammatory Response | 0.00696855 | 0.02488767 | 2.05086171 | 10.18529386 |
| G2-M Checkpoint | 0.01489679 | 0.046552476 | 1.91739637 | 8.065737508 |
| Complement | 0.01489679 | 0.046552476 | 1.91739637 | 8.065737508 |
|  |  |  |  |  |
| **Enriched pathways associated with genes with decreased promoter accessibility** | | | | |
| Term | P-value | Adjusted P-value | Odds Ratio | Combined Score |
| UV Response Dn | 9.9421E-08 | 2.23697E-06 | 6.24415401 | 100.6801295 |
| Epithelial Mesenchymal Transition | 5.1393E-08 | 2.23697E-06 | 5.34065934 | 89.63641636 |
| TGF-beta Signaling | 6.7183E-05 | 0.00100774 | 7.85828984 | 75.50319598 |
| IL-2/STAT5 Signaling | 0.00042302 | 0.004759013 | 3.40755676 | 26.47018407 |
| TNF-alpha Signaling via NF-kB | 0.00153593 | 0.013823396 | 3.08180154 | 19.96581287 |
