# Supplementary material for: Epigenetic and Metabolic Reprogramming of Fibroblasts in Crohn’s Disease Strictures Reveals Histone Deacetylases as Therapeutic Targets
Source: J Crohns Colitis. 2023 Dec 9;18(6):895–907. doi: 10.1093/ecco-jcc/jjad209 (PMC11147807; doi:10.1093/ecco-jcc/jjad209)
Supplement: jjad209_suppl_Supplementary_Tables_9 [file jjad209_suppl_supplementary_tables_9.docx]

| **Supplementary Table 9. Genes up- and down-regulated by valproic acid (Illumina HT-12 array).**  **Genes up-regulated by VPA** | | | | | | | | | | |
| --- | --- | --- | --- | --- | --- | --- | --- | --- | --- | --- |
| Gene ID | Name ILMN_ | Score(d) | Numerator(r) | Denominator(s+s0) | Fold Change | q-value(%) | | |  |  |
| TUBB2B | 1680874 | 14.8164045016203 | 3.13198896648295 | 0.211386572642536 | 8.76642708699494 | 0 | |  |  |  |
| HLA-DMA | 1695311 | 8.4974048936405 | 2.93111363554916 | 0.344942211444205 | 7.62698908729323 | 0 | |  |  |  |
| LMTK3 | 1668194 | 13.7656203597202 | 2.59039592808692 | 0.18817865525819 | 6.02263959461898 | 0 | |  |  |  |
| CCK | 1712943 | 12.2038241016622 | 2.34203363503671 | 0.191909815769773 | 5.07016829281985 | 0 | |  |  |  |
| SLC12A8 | 1762529 | 16.579642562227 | 2.22444236336984 | 0.134167088043124 | 4.67330230039804 | 0 | |  |  |  |
| STMN3 | 3244117 | 9.63259024099897 | 2.15367782061053 | 0.223582418303633 | 4.44960669528751 | 0 | |  |  |  |
| TSPAN13 | 1669881 | 6.76525561477142 | 2.09512136359992 | 0.309688426114364 | 4.27262104207786 | 0 | |  |  |  |
| NFE2L3 | 2049766 | 8.84672564009074 | 2.07720952731229 | 0.234799813153354 | 4.21990209393215 | 0 | |  |  |  |
| CA12 | 1720998 | 11.1199103937652 | 2.0761523236961 | 0.186705850153269 | 4.21681089210995 | 0 | |  |  |  |
| CD24 | 2060413 | 4.82638126813729 | 2.01171356251147 | 0.416816130087435 | 4.03260909242312 | 0 | |  |  |  |
| C20orf100 | 2082209 | 6.82371234162272 | 2.00045056530575 | 0.29316162012041 | 4.00124942737834 | 0 | |  |  |  |
| ALDOC | 1755974 | 7.02180159422658 | 1.92170841222657 | 0.273677401225153 | 3.78871445564223 | 0 | |  |  |  |
| DNAJC12 | 2269256 | 12.2282581926922 | 1.91966708741226 | 0.156986142847351 | 3.78335744835502 | 0 | |  |  |  |
| HCFC1R1 | 1757877 | 7.09606223380641 | 1.9135456044154 | 0.269663024557347 | 3.76733833648293 | 0 | |  |  |  |
| AQP11 | 1762606 | 5.937023591854 | 1.90083756848841 | 0.32016675343795 | 3.73429931517431 | 0 | |  |  |  |
| ADORA2B | 1703946 | 9.13552507326262 | 1.88806475496357 | 0.206672822834175 | 3.70138384400202 | 0 | |  |  |  |
| HCFC1R1 | 2318568 | 7.5908874327938 | 1.88105465620134 | 0.247804314430338 | 3.68344233262293 | 0 | |  |  |  |
| ALPL | 1701603 | 7.13033446847678 | 1.87869193484385 | 0.263478795160247 | 3.67741484584129 | 0 | |  |  |  |
| DNAJC12 | 2311020 | 11.6370429946519 | 1.86033579067352 | 0.159863273816939 | 3.63092162849908 | 0 | |  |  |  |
| CPA4 | 1784294 | 7.21314044927006 | 1.78096164077428 | 0.246905166106188 | 3.43655164551418 | 0 | |  |  |  |
| DNAJC12 | 1803073 | 9.42404521673096 | 1.75815162151074 | 0.18656018525775 | 3.38264463332688 | 0 | |  |  |  |
| SRXN1 | 1804822 | 6.34506519884322 | 1.73003316115157 | 0.272658058969508 | 3.31735443362654 | 0 | |  |  |  |
| CYFIP2 | 1677200 | 6.00979703419544 | 1.66628868293877 | 0.27726205618221 | 3.17397041996148 | 0 | |  |  |  |
| HSPA2 | 1766499 | 7.43204070481396 | 1.62937790939445 | 0.219236946366434 | 3.09379565365646 | 0 | |  |  |  |
| INA | 1673704 | 5.21406247153859 | 1.61836073486776 | 0.310383840566108 | 3.07025979102491 | 0 | |  |  |  |
| AKR1C2 | 2412336 | 7.30867331613921 | 1.60821141405586 | 0.220041496519561 | 3.0487363929992 | 0 | |  |  |  |
| GPX3 | 1726666 | 5.27463919626406 | 1.57955230989924 | 0.299461679012663 | 2.98877089252107 | 0 | |  |  |  |
| SERPINB7 | 1767685 | 5.85385935412892 | 1.57623038180435 | 0.269263452783946 | 2.98189691021926 | 0 | |  |  |  |
| GALNTL1 | 2077952 | 6.56279526929365 | 1.55854858903729 | 0.237482433183541 | 2.94557357443459 | 0 | |  |  |  |
| WNT2B | 1740269 | 4.98861620025174 | 1.54162917876801 | 0.309029421563882 | 2.91123071744482 | 0 | |  |  |  |
| CKB | 1671478 | 7.5011846241403 | 1.53920001884862 | 0.205194258770164 | 2.90633301248917 | 0 | |  |  |  |
| LOC255783 | 1703316 | 5.68570145490056 | 1.53734970489977 | 0.270388749232465 | 2.90260791375177 | 0 | |  |  |  |
| PROCR | 1717262 | 4.91704180187444 | 1.53452855746518 | 0.312083691637562 | 2.89693749589601 | 0 | |  |  |  |
| VGF | 1757497 | 5.03412555623073 | 1.53371717652801 | 0.304664069141011 | 2.89530869774814 | 0 | |  |  |  |
| TIMP3 | 1701461 | 4.1975820824875 | 1.52257459921573 | 0.362726581468884 | 2.87303307026313 | 0 | |  |  |  |
| IL8 | 2184373 | 4.68610498147006 | 1.51850025440735 | 0.324043157464857 | 2.86493072472302 | 0 | |  |  |  |
| KYNU | 1746517 | 9.445656704255 | 1.51804265881228 | 0.160713299915764 | 2.86402216695047 | 0 | |  |  |  |
| MGC87042 | 1663575 | 8.26192123829637 | 1.51570734336158 | 0.183457007110627 | 2.85938987486009 | 0 | |  |  |  |
| TMEM119 | 1738116 | 7.36872722226976 | 1.51458951455765 | 0.205542893483729 | 2.85717522081933 | 0 | |  |  |  |
| LOC643031 | 1743078 | 5.99934005974278 | 1.51149430805523 | 0.251943429277793 | 2.85105191185225 | 0 | |  |  |  |
| TMOD1 | 1736911 | 4.71095414939553 | 1.5076491762854 | 0.320030534892566 | 2.84346328511015 | 0 | |  |  |  |
| FBLN2 | 1774602 | 8.75440357271721 | 1.5055588530317 | 0.171977318674652 | 2.83934636963151 | 0 | |  |  |  |
| HBG1 | 1796678 | 7.14105570842768 | 1.50047048967235 | 0.210118860703122 | 2.82934967783435 | 0 | |  |  |  |
| NRIP3 | 1759563 | 5.63430905169856 | 1.48580417713342 | 0.263706545647421 | 2.80073246116333 | 0 | |  |  |  |
| COL11A1 | 1789507 | 5.28260025226234 | 1.47976574233894 | 0.280120711709203 | 2.78903442636249 | 0 | |  |  |  |
| IGFBP2 | 1725193 | 3.6918479697349 | 1.47481025610339 | 0.399477515919837 | 2.77947085839488 | 0 | |  |  |  |
| RGS10 | 1668559 | 5.6552587418582 | 1.47110381483152 | 0.260130240185642 | 2.77233925876694 | 0 | |  |  |  |
| SLC29A4 | 1801377 | 7.82013276514421 | 1.47043610728445 | 0.188032115495335 | 2.77105646268438 | 0 | |  |  |  |
| ACCN2 | 1769694 | 5.02520924572241 | 1.45040321990823 | 0.28862543806367 | 2.73284421143846 | 0 | |  |  |  |
| C7orf69 | 1695301 | 9.85445679974878 | 1.43760466144066 | 0.14588370426235 | 2.70870759205798 | 0 | |  |  |  |
| ELMOD1 | 1810604 | 7.7825335032277 | 1.43153799025737 | 0.183942412797023 | 2.69734113180449 | 0 | |  |  |  |
| DNAJC12 | 1725773 | 7.81514608290354 | 1.42340175787096 | 0.182133736563774 | 2.6821720032825 | 0 | |  |  |  |
| ACN9 | 2134039 | 7.23860506452544 | 1.42257127584724 | 0.196525609998935 | 2.68062846527318 | 0 | |  |  |  |
| LPPR4 | 2172497 | 5.48685169598958 | 1.42241431474476 | 0.259240525087351 | 2.68033683641211 | 0 | |  |  |  |
| UBL3 | 1810729 | 5.5088393040321 | 1.42036717655629 | 0.257834200303623 | 2.6765362211552 | 0 | |  |  |  |
| TPMT | 1740185 | 6.58770772624479 | 1.41894539133167 | 0.215392887829363 | 2.67389977685619 | 0 | |  |  |  |
|  | 1846306 | 4.51235952155865 | 1.41269933603126 | 0.313073311043108 | 2.662348323595 | 0 | |  |  |  |
| LMCD1 | 1754969 | 5.78670070455889 | 1.40608205526625 | 0.242985101019395 | 2.65016476157259 | 0 | |  |  |  |
| CSRP2 | 1660806 | 4.40945154275669 | 1.40408474599919 | 0.318426165336969 | 2.64649833440104 | 0 | |  |  |  |
| GDF15 | 2188862 | 5.26729511132969 | 1.40212231697714 | 0.266193992806904 | 2.64290087664791 | 0 | |  |  |  |
| LUM | 1790529 | 5.86373950303214 | 1.40083673080442 | 0.238898186060285 | 2.64054683553696 | 0 | |  |  |  |
|  | 1879326 | 8.35249461924031 | 1.39941162365171 | 0.167544151471599 | 2.63793976744963 | 0 | |  |  |  |
|  | 1821280 | 5.12158112505434 | 1.39931611821898 | 0.27321955545596 | 2.63776514340713 | 0 | |  |  |  |
| SLC46A3 | 1658639 | 7.10236526063079 | 1.39912842928162 | 0.19699471625842 | 2.63742200288179 | 0 | |  |  |  |
| FBLN2 | 2390919 | 10.2815617036668 | 1.39450596633759 | 0.135631726631592 | 2.62898510174417 | 0 | |  |  |  |
| LIPA | 1718063 | 6.76859706724492 | 1.38905717377995 | 0.205220839707237 | 2.61907463763079 | 0 | |  |  |  |
| TOX2 | 3298423 | 5.71302326672845 | 1.38124565986482 | 0.241771404627202 | 2.60493190512361 | 0 | |  |  |  |
| TNFAIP6 | 1785732 | 7.5243057189412 | 1.37158383200084 | 0.182287095080162 | 2.58754478344418 | 0 | |  |  |  |
| SLC9A3R1 | 1680925 | 5.96235590759653 | 1.37136956763872 | 0.230004647305855 | 2.58716051826783 | 0 | |  |  |  |
| ENDOD1 | 1699022 | 4.27520463088981 | 1.37028578859776 | 0.320519345131922 | 2.58521772553963 | 0 | |  |  |  |
| AP1S2 | 2120273 | 7.63106547068189 | 1.36688994760277 | 0.179121769149312 | 2.57913975055668 | 0 | |  |  |  |
| SOBP | 1666502 | 4.86623626022852 | 1.36363445806977 | 0.28022364413637 | 2.57332639706905 | 0 | |  |  |  |
| CXCL12 | 1791447 | 3.3246480059049 | 1.36034325780976 | 0.409169107644979 | 2.56746259416984 | 0 | |  |  |  |
| HBG2 | 2084825 | 6.89447967733358 | 1.35852699362389 | 0.197045615797549 | 2.56423235068594 | 0 | |  |  |  |
| MOXD1 | 1687501 | 8.33216203460723 | 1.35762675111017 | 0.162938112037588 | 2.56263276738496 | 0 | |  |  |  |
| FLJ20489 | 1778144 | 5.84657878890967 | 1.35720569672414 | 0.232136732562061 | 2.56188496532314 | 0 | |  |  |  |
| NDRG4 | 2305225 | 5.90621621808675 | 1.34298618351398 | 0.22738520465968 | 2.53675850435859 | 0 | |  |  |  |
| AP1S2 | 1766411 | 10.8555302485017 | 1.33401448621171 | 0.122888007833227 | 2.5210320969158 | 0 | |  |  |  |
| SLC48A1 | 3247587 | 6.47814035538242 | 1.33070003245504 | 0.205413893409922 | 2.51524691499045 | 0 | |  |  |  |
| TM4SF1 | 1770338 | 6.09645951272116 | 1.32162950779195 | 0.216786399554392 | 2.499482641531 | 0 | |  |  |  |
| POLR3G | 3251691 | 5.52722767150956 | 1.32045686717445 | 0.238900393768983 | 2.4974518561376 | 0 | |  |  |  |
| RGS10 | 1733538 | 7.89624525171222 | 1.31774949577712 | 0.166883050585515 | 2.49276951570505 | 0 | |  |  |  |
| SYTL2 | 2336609 | 5.18142155062141 | 1.3154415862332 | 0.253876580660656 | 2.4887849679113 | 0 | |  |  |  |
| C14orf132 | 1713744 | 6.12692346235689 | 1.30890735367055 | 0.213632071905636 | 2.47753828903991 | 0 | |  |  |  |
| LOC100129668 | 3241692 | 7.94880877825561 | 1.30408557504082 | 0.164060504085621 | 2.46927167700468 | 0 | |  |  |  |
| EXTL2 | 1797950 | 8.51807298781508 | 1.29986755684234 | 0.152601129234484 | 2.46206279254577 | 0 | |  |  |  |
| LDB2 | 1800697 | 10.9910860906028 | 1.29367880480157 | 0.117702544965746 | 2.4515238629907 | 0 | |  |  |  |
| EXTL2 | 2388425 | 5.18509161600358 | 1.29326605523578 | 0.24942009727353 | 2.45082259165499 | 0 | |  |  |  |
| CFD | 1777190 | 4.4653123673131 | 1.28511404369092 | 0.287799360487789 | 2.43701316334309 | 0 | |  |  |  |
| C20orf27 | 1697363 | 5.58108875166616 | 1.28465893954192 | 0.230180704286131 | 2.4362445186507 | 0 | |  |  |  |
| C19orf4 | 1730945 | 6.46550758993267 | 1.27761811294481 | 0.197605229778736 | 2.4243838092442 | 0 | |  |  |  |
| ASMTL | 1732615 | 8.87593992869198 | 1.27595631841534 | 0.14375450134478 | 2.42159284636653 | 0 | |  |  |  |
| FAM69B | 1757440 | 4.85851869276831 | 1.27456941343573 | 0.262337040162646 | 2.41926601687184 | 0 | |  |  |  |
| CORO2A | 1813746 | 7.68125106390492 | 1.27250732657083 | 0.16566407164459 | 2.41581055819339 | 0 | |  |  |  |
| MGC39900 | 1740512 | 7.62881488860177 | 1.26987220710956 | 0.166457336513286 | 2.41140204565985 | 0 | |  |  |  |
| BOLA2 | 1782633 | 4.98195390264334 | 1.2695657592428 | 0.254832899712137 | 2.41088988576888 | 0 | |  |  |  |
| CCND2 | 2067656 | 4.02636125221278 | 1.2674895541835 | 0.314797772675494 | 2.40742283166833 | 0 | |  |  |  |
| TSPAN13 | 2130525 | 2.37014856236937 | 1.2669713334007 | 0.534553552260935 | 2.40655823279674 | 0.0359206925696922 | |  |  |  |
| ITM2C | 2366041 | 4.82313573776069 | 1.25829445721685 | 0.260887216456623 | 2.39212778224865 | 0 | |  |  |  |
| FAM105A | 1695711 | 4.99780494150789 | 1.25759436087085 | 0.251629340398271 | 2.3909672364699 | 0 | |  |  |  |
| TMEM14A | 1785191 | 7.1711759826351 | 1.25723393167256 | 0.175317679376009 | 2.39036997458158 | 0 | |  |  |  |
| SLC44A1 | 1700695 | 5.47067054975361 | 1.25178264766885 | 0.228817041034435 | 2.38135490354701 | 0 | |  |  |  |
| CD82 | 2392274 | 5.96656802249109 | 1.25083008478764 | 0.209639792938354 | 2.37978309417787 | 0 | |  |  |  |
| STEAP1 | 1733094 | 9.16557445203 | 1.24411812907192 | 0.135738151010968 | 2.36873716993989 | 0 | |  |  |  |
| PSG4 | 2384745 | 5.5645012132643 | 1.23827402707099 | 0.222531001362579 | 2.35916124363066 | 0 | |  |  |  |
| ACN9 | 1771348 | 4.16229334455944 | 1.2303728210973 | 0.295599737751671 | 2.34627614455628 | 0 | |  |  |  |
| CYB5A | 1714167 | 7.95442678110397 | 1.22762558656283 | 0.154332376215857 | 2.3418125279066 | 0 | |  |  |  |
| IL12A | 1671353 | 7.83392831376614 | 1.22741796605689 | 0.156679754638553 | 2.34147553824196 | 0 | |  |  |  |
| ITM2C | 1680453 | 6.44264561104261 | 1.22727263170274 | 0.19049202855411 | 2.34123967433059 | 0 | |  |  |  |
| ALDH4A1 | 1656368 | 5.47161505529349 | 1.22401465072368 | 0.223702624975329 | 2.33595851101632 | 0 | |  |  |  |
| TMEM35 | 2227266 | 4.6519824774775 | 1.21196269559543 | 0.260526066351954 | 2.31652571218365 | 0 | |  |  |  |
| MMD | 1733937 | 4.64029132533056 | 1.20892147042456 | 0.260527062993926 | 2.31164758080362 | 0 | |  |  |  |
| VAT1L | 1749466 | 4.9968974894237 | 1.20327336218123 | 0.240804091884624 | 2.30261524137749 | 0 | |  |  |  |
| LOC729970 | 3305942 | 6.32449821223963 | 1.20326920977111 | 0.190255284987267 | 2.30260861392761 | 0 | |  |  |  |
| LOC388588 | 1749834 | 8.73484482894601 | 1.20199192841863 | 0.137608847318661 | 2.30057091544515 | 0 | |  |  |  |
| SERPINB7 | 2395139 | 5.10293142140037 | 1.20179676514305 | 0.235511055489209 | 2.30025972245188 | 0 | |  |  |  |
|  | 1906423 | 6.5854601754128 | 1.20081430490899 | 0.182343264240257 | 2.29869380288666 | 0 | |  |  |  |
| SLC25A23 | 1679949 | 9.47429930288845 | 1.19972554297589 | 0.126629474605064 | 2.29695969685284 | 0 | |  |  |  |
| GMDS | 1711227 | 4.14543863990545 | 1.19658133803408 | 0.288650114493401 | 2.2919591613164 | 0 | |  |  |  |
| SEC11C | 1701681 | 8.73630031724375 | 1.19445862598145 | 0.13672362242674 | 2.28858936259606 | 0 | |  |  |  |
| PGBD3 | 1737416 | 5.50440011999432 | 1.19357250166213 | 0.216839705625064 | 2.28718410926438 | 0 | |  |  |  |
| LOC100134073 | 3242105 | 4.21838520771323 | 1.18847251155446 | 0.28173636428019 | 2.2791130874544 | 0 | |  |  |  |
| BMP6 | 1747650 | 4.84714997217578 | 1.17806158890508 | 0.243042116639167 | 2.26272551978045 | 0 | |  |  |  |
| C18orf56 | 1796074 | 4.28547876240245 | 1.17467820514044 | 0.274106644850553 | 2.25742523204649 | 0 | |  |  |  |
| THEM2 | 2098743 | 4.6664684407003 | 1.17235119450997 | 0.25122878455255 | 2.25378702843693 | 0 | |  |  |  |
| CHN1 | 1678493 | 5.93485968164553 | 1.16916546546697 | 0.196999681236407 | 2.2488157537679 | 0 | |  |  |  |
| FNBP1L | 1754600 | 10.6096308281292 | 1.16776444229296 | 0.110066453886112 | 2.24663295451219 | 0 | |  |  |  |
| SLC40A1 | 2053103 | 4.55233550170846 | 1.16158233410837 | 0.255161846852552 | 2.23702648034775 | 0 | |  |  |  |
| ANKRD1 | 1716264 | 2.25195508823101 | 1.1584605521812 | 0.514424358742967 | 2.23219111428762 | 0.0521258231948829 | |  |  |  |
| TNC | 1719759 | 3.29134515009023 | 1.15629963795548 | 0.351315217707805 | 2.22885017060286 | 0 | |  |  |  |
| PRCP | 1769091 | 5.89904134852308 | 1.15165807392489 | 0.195228005006819 | 2.2216908428584 | 0 | |  |  |  |
| RGS4 | 1758067 | 4.62999961055274 | 1.15144048763468 | 0.248691270947476 | 2.2213557942148 | 0 | |  |  |  |
| ASMTL | 1689786 | 5.26261542438616 | 1.15135910039635 | 0.218780778671596 | 2.22123048366134 | 0 | |  |  |  |
| SCG5 | 2065773 | 4.70615252275377 | 1.14841670258631 | 0.244024539585963 | 2.21670486793413 | 0 | |  |  |  |
| NME7 | 1716583 | 10.4277973882992 | 1.14770830142007 | 0.11006238984926 | 2.21561667479631 | 0 | |  |  |  |
| NUDT6 | 2366972 | 4.45105475879215 | 1.14601660953775 | 0.257470795494939 | 2.21302018421674 | 0 | |  |  |  |
| GPR162 | 1730816 | 6.80890271546807 | 1.14260997227644 | 0.167811176047607 | 2.20780074217634 | 0 | |  |  |  |
| COL12A1 | 1733756 | 5.86141859356645 | 1.14235916670528 | 0.194894657064613 | 2.20741696003131 | 0 | |  |  |  |
| HRASLS3 | 1667711 | 4.35615666369899 | 1.14208593811948 | 0.262177425260386 | 2.20699894216325 | 0 | |  |  |  |
| LOC653344 | 1693836 | 5.58077348429321 | 1.14144148406907 | 0.20453105421347 | 2.20601329256951 | 0 | |  |  |  |
| TAC3 | 1793006 | 3.57572230652659 | 1.13928223080739 | 0.318615969905693 | 2.20271406546993 | 0 | |  |  |  |
| PKIA | 2337974 | 5.11298112335535 | 1.13687798534872 | 0.222351297202257 | 2.1990463083765 | 0 | |  |  |  |
| CYB5A | 2312194 | 4.03376424544106 | 1.13509327947974 | 0.281398021900416 | 2.19632762963955 | 0 | |  |  |  |
| LYPD1 | 1668863 | 4.68991929871636 | 1.13101893467136 | 0.241159572826961 | 2.19013368583889 | 0 | |  |  |  |
| RABL4 | 1746492 | 9.05324754804453 | 1.12782074839801 | 0.124576373551346 | 2.18528394473662 | 0 | |  |  |  |
| CYP2U1 | 2097259 | 4.51924087793325 | 1.12705587445416 | 0.249390529271718 | 2.18412567933802 | 0 | |  |  |  |
| TMEM170B | 3236428 | 3.82303638799995 | 1.12642304715208 | 0.294640943174851 | 2.18316783916666 | 0 | |  |  |  |
| TFB1M | 1733562 | 5.4310800320713 | 1.12331206149122 | 0.206830327459346 | 2.17846519163053 | 0 | |  |  |  |
| PTGS1 | 2339835 | 2.50875271462276 | 1.11019127599844 | 0.442527184735056 | 2.15874266536103 | 0.0359206925696922 | |  |  |  |
| ASTN2 | 1744118 | 6.95640525361428 | 1.10772557487001 | 0.15923821779855 | 2.1550563227376 | 0 | |  |  |  |
| ADRB2 | 1695590 | 4.78898147033277 | 1.10748141315503 | 0.231256149144816 | 2.15469163185721 | 0 | |  |  |  |
| DACT3 | 1733851 | 3.81546646199922 | 1.10727960602327 | 0.290208187400258 | 2.15439025026613 | 0 | |  |  |  |
| SDPR | 1715991 | 4.07499237797453 | 1.10726050493672 | 0.271720877546055 | 2.15436172662042 | 0 | |  |  |  |
| ZNF277 | 1681628 | 6.32638167138938 | 1.10141806389984 | 0.17409921201576 | 2.14565491103664 | 0 | |  |  |  |
| COL8A1 | 2402392 | 4.98740831694829 | 1.10105182132738 | 0.220766328192093 | 2.14511028419938 | 0 | |  |  |  |
| SLIT3 | 1811313 | 5.38050851155055 | 1.10085748853383 | 0.204601012370964 | 2.14482135467005 | 0 | |  |  |  |
| C11orf74 | 1659017 | 5.07118782252519 | 1.09862847478216 | 0.216641251168469 | 2.14151009013968 | 0 | |  |  |  |
| TRNP1 | 1695946 | 6.27260458779059 | 1.09831372209517 | 0.175096916555684 | 2.14104292797925 | 0 | |  |  |  |
| COL8A1 | 1685433 | 6.55186468506784 | 1.0936447521578 | 0.166921144548406 | 2.13412510645678 | 0 | |  |  |  |
| C9orf95 | 1674650 | 7.27357534352892 | 1.09293302906347 | 0.150260769627666 | 2.13307254061097 | 0 | |  |  |  |
| LOC338758 | 1795835 | 6.03263338266982 | 1.09074138700287 | 0.180806841359909 | 2.12983458508483 | 0 | |  |  |  |
| PTGER2 | 1703926 | 6.12838184655241 | 1.08820343393838 | 0.177567824784051 | 2.12609112696948 | 0 | |  |  |  |
| TGM2 | 1705750 | 6.64232940821905 | 1.08595282765409 | 0.163489758022293 | 2.1227770075214 | 0 | |  |  |  |
| AKR1C3 | 1713124 | 5.28717520665536 | 1.08218991360461 | 0.204682060137212 | 2.11724748179913 | 0 | |  |  |  |
| HIBCH | 1656977 | 4.93148669652119 | 1.0799035965933 | 0.218981346407179 | 2.11389482225285 | 0 | |  |  |  |
| GALNT10 | 1681984 | 4.47877547393894 | 1.07837550966965 | 0.240774630464174 | 2.1116569931595 | 0 | |  |  |  |
| CCDC34 | 1657547 | 4.04779105413831 | 1.07836222195727 | 0.266407580711162 | 2.11163754416941 | 0 | |  |  |  |
| ARRDC4 | 1660544 | 4.35476740200267 | 1.07589735131087 | 0.247061955781171 | 2.10803285333888 | 0 | |  |  |  |
| BEX1 | 2234697 | 4.01465180210062 | 1.07467072936183 | 0.26768715752622 | 2.10624130324624 | 0 | |  |  |  |
| PSAT1 | 1692938 | 5.46192239302855 | 1.07176487276083 | 0.196224844594789 | 2.10200321043455 | 0 | |  |  |  |
| MLPH | 1795342 | 7.58809911553709 | 1.06411530284033 | 0.140234765866657 | 2.09088730129034 | 0 | |  |  |  |
| DLEU1 | 2043918 | 4.71473342540077 | 1.06001521308921 | 0.224830359947468 | 2.08495350721319 | 0 | |  |  |  |
| SCG2 | 1703178 | 4.61119486474303 | 1.05775381053862 | 0.229388226168048 | 2.08168793431422 | 0 | |  |  |  |
| BOLA2 | 1810680 | 4.61248859382121 | 1.056611897756 | 0.229076316670227 | 2.08004090186002 | 0 | |  |  |  |
| CYGB | 1758128 | 5.50573016350886 | 1.0502437615981 | 0.190754673841257 | 2.07087971947945 | 0 | |  |  |  |
| TMEM54 | 2059689 | 5.15360025841435 | 1.04969896539054 | 0.203682651497209 | 2.07009785332392 | 0 | |  |  |  |
| CA12 | 2382942 | 5.13344896362559 | 1.04893492192872 | 0.20433336911718 | 2.06900183098791 | 0 | |  |  |  |
| CDKN2B | 2376723 | 5.4022647777325 | 1.04864093129232 | 0.194111354114795 | 2.06858025527302 | 0 | |  |  |  |
| SLC29A1 | 1723971 | 6.68853672165687 | 1.0476916903392 | 0.156639895083012 | 2.06721965223731 | 0 | |  |  |  |
| MGC39900 | 1737283 | 5.61914766759092 | 1.04672002637145 | 0.186277365944399 | 2.06582783586047 | 0 | |  |  |  |
| RND2 | 1736533 | 5.07337834976074 | 1.04578739505881 | 0.206132348695841 | 2.06449281143618 | 0 | |  |  |  |
| F2RL2 | 1728923 | 4.89949165472582 | 1.04010772849858 | 0.212288907053314 | 2.05638120106975 | 0 | |  |  |  |
| SLC22A17 | 1653200 | 6.83089924280458 | 1.03712463706937 | 0.151828419686008 | 2.05213357059759 | 0 | |  |  |  |
| FLJ10781 | 1794490 | 4.37391688231437 | 1.03353778520891 | 0.236295707718624 | 2.04703785990287 | 0 | |  |  |  |
| MORN2 | 1756826 | 4.89344839580979 | 1.02811390711198 | 0.210100081568725 | 2.0393563757859 | 0 | |  |  |  |
| NUDT6 | 1665192 | 4.43524952573707 | 1.01793302798318 | 0.229509754090784 | 2.0250156083286 | 0 | |  |  |  |
| C11orf67 | 1779163 | 5.23619719994774 | 1.01762990204452 | 0.194345221004029 | 2.02459017519303 | 0 | |  |  |  |
|  | 1827736 | 4.67403506350604 | 1.01581612930471 | 0.217331730614519 | 2.02204643656514 | 0 | |  |  |  |
| CHSY3 | 1740407 | 3.31880079773009 | 1.01523562237013 | 0.305904356496631 | 2.02123297577158 | 0 | |  |  |  |
| ADARB1 | 1679797 | 5.97874368549083 | 1.01320841575023 | 0.169468448398126 | 2.01839482957664 | 0 | |  |  |  |
| GLDN | 1704376 | 3.39043022607394 | 1.01261379062124 | 0.29866822883827 | 2.01756309386714 | 0 | |  |  |  |
| NBL1 | 2405009 | 5.64412244164886 | 1.00979596511475 | 0.178911066433164 | 2.01362630044799 | 0 | |  |  |  |
| DHCR24 | 1725510 | 3.46473605962402 | 1.00957256545037 | 0.291385129509671 | 2.01331451687635 | 0 | |  |  |  |
| HS3ST3A1 | 1692056 | 4.65686556460805 | 1.0094720771255 | 0.216770714790962 | 2.01317428796328 | 0 | |  |  |  |
| SYTL2 | 1682929 | 4.39060202638434 | 1.0084497537543 | 0.229683707995907 | 2.01174821659862 | 0 | |  |  |  |
| C16orf57 | 1765880 | 4.08908433248971 | 1.00691253152839 | 0.246244012021956 | 2.00960579771971 | 0 | |  |  |  |
| GPD1L | 1694106 | 3.16911530765346 | 1.00526485519333 | 0.317206777792401 | 2.00731197281445 | 0 | |  |  |  |
| MICA | 1797929 | 3.07038546008547 | 1.00450247269555 | 0.327158425466094 | 2.00625150251775 | 0 | |  |  |  |
| TUBA4A | 1784300 | 5.47097247986063 | 1.00348347125244 | 0.183419579415981 | 2.00483495134126 | 0 | |  |  |  |
| RPAIN | 1770339 | 3.76904992260253 | 1.0027991540649 | 0.266061520716728 | 2.00388421840785 | 0 | |  |  |  |
| CYP2U1 | 1790008 | 5.34844811466187 | 1.00183330347131 | 0.187312895627603 | 2.00254311375207 | 0 | |  |  |  |
| ATP6V0E2 | 1785095 | 3.97203615644754 | 1.00076779503487 | 0.251953344737407 | 2.00106467320907 | 0 | |  |  |  |
| BOLA2 | 1659343 | 5.46065368697292 | 0.999194862081944 | 0.182980815001261 | 1.9988841532386 | 0 | |  |  |  |
| CHCHD7 | 1744138 | 3.33749631506557 | 0.997743179504477 | 0.29894959733757 | 1.99687382825942 | 0 | |  |  |  |
| CCRK | 2357730 | 5.673757047346 | 0.997125300878828 | 0.175743390589002 | 1.99601878859586 | 0 | |  |  |  |
| MGC39900 | 1731640 | 4.62777877582095 | 0.996907714588613 | 0.215418187186739 | 1.99571777309234 | 0 | |  |  |  |
| BMP4 | 1740900 | 4.81605446738551 | 0.996535658641987 | 0.206919515838237 | 1.9952031647319 | 0 | |  |  |  |
| GABBR2 | 1660718 | 4.28826892860394 | 0.996090520277271 | 0.232282661573082 | 1.99458764693602 | 0 | |  |  |  |
| CD248 | 1726589 | 3.36890892127462 | 0.99526252969962 | 0.29542577521599 | 1.99344324295043 | 0 | |  |  |  |
| NQO1 | 2354953 | 3.58146946665903 | 0.994893795681087 | 0.277789271957461 | 1.99293380998628 | 0 | |  |  |  |
| LOC404266 | 1683798 | 3.38475276273342 | 0.991608408795245 | 0.292963320604384 | 1.98840055185579 | 0 | |  |  |  |
| SERPINB2 | 2150851 | 5.34314275623734 | 0.984720390890496 | 0.184296103588282 | 1.97892973876243 | 0 | |  |  |  |
| TMEM118 | 1738093 | 3.96142098312003 | 0.984059327199613 | 0.248410691868594 | 1.97802317231001 | 0 | |  |  |  |
| PDGFRL | 1680339 | 4.98978886502438 | 0.983006275993533 | 0.197003581230431 | 1.9765799014754 | 0 | |  |  |  |
| CCPG1 | 1794190 | 3.32328184857026 | 0.982395871706098 | 0.295610157810943 | 1.97574378739007 | 0 | |  |  |  |
| HRSP12 | 1807633 | 3.92821159782353 | 0.98165757318701 | 0.249899362277457 | 1.97473296008907 | 0 | |  |  |  |
| QPRT | 1700268 | 4.35369136116452 | 0.981341159535972 | 0.22540439322127 | 1.97429990671776 | 0 | |  |  |  |
| C3orf39 | 1792415 | 6.85325766294297 | 0.98115347059861 | 0.143165997669096 | 1.97404307478985 | 0 | |  |  |  |
| SLC30A3 | 1805807 | 3.9874023034605 | 0.979265784958691 | 0.24558991303908 | 1.97146183908359 | 0 | |  |  |  |
| PRCP | 2367215 | 2.05222382115482 | 0.978593094519476 | 0.476845207833523 | 1.97054281298397 | 0.0848201265005491 | |  |  |  |
| ATP1B1 | 2407824 | 4.38048454217977 | 0.977378099318771 | 0.223121001776762 | 1.96888397857104 | 0 | |  |  |  |
| HIST1H2BD | 1651496 | 4.64054029974343 | 0.976549278259097 | 0.210438702216009 | 1.9677531894552 | 0 | |  |  |  |
| NCRNA00152 | 3220934 | 3.337137791667 | 0.972592861898085 | 0.291445221209235 | 1.96236425735881 | 0 | |  |  |  |
| NQO1 | 1720282 | 4.16531557773904 | 0.97113038305431 | 0.233146892457413 | 1.96037599096539 | 0 | |  |  |  |
| EDEM2 | 1711909 | 5.86648957903171 | 0.971047334851939 | 0.165524428496848 | 1.96026314590003 | 0 | |  |  |  |
| SNCA | 1701933 | 4.30299024118937 | 0.970067366063947 | 0.225440289587042 | 1.95893206458215 | 0 | |  |  |  |
| STAT4 | 1785202 | 7.49771090238044 | 0.96706019065605 | 0.128980725350323 | 1.95485308951385 | 0 | |  |  |  |
| PPAPDC1A | 1689177 | 3.89188990249144 | 0.966543630837295 | 0.248348143203782 | 1.95415327578303 | 0 | |  |  |  |
| RNF20 | 1710758 | 6.33021171368093 | 0.964876022933662 | 0.152423973569219 | 1.95189577944229 | 0 | |  |  |  |
| 38596 | 1789627 | 4.51476020696102 | 0.96445829047573 | 0.213623370071503 | 1.95133068968693 | 0 | |  |  |  |
| FZD8 | 1729368 | 3.43798672285317 | 0.962191905032993 | 0.279870744885971 | 1.94826767536339 | 0 | |  |  |  |
| LOC728431 | 3305849 | 6.19226153270967 | 0.960747696793741 | 0.155152958530375 | 1.94631834006284 | 0 | |  |  |  |
| IDH2 | 1751753 | 5.51700097426794 | 0.960019364058936 | 0.174011091993023 | 1.94533600522711 | 0 | |  |  |  |
| LASS6 | 1767662 | 4.15963940782911 | 0.95803451202224 | 0.230316721737722 | 1.94266146270186 | 0 | |  |  |  |
| SNCA | 1766165 | 6.1628259608869 | 0.957396701828023 | 0.155350274030819 | 1.94180280900132 | 0 | |  |  |  |
| TMEM205 | 1730734 | 3.46833883930994 | 0.95523827904837 | 0.275416654284684 | 1.93889984087543 | 0 | |  |  |  |
| LOC440132 | 1759374 | 4.16921243773648 | 0.954721719229615 | 0.22899330113002 | 1.93820573821442 | 0 | |  |  |  |
| GLRB | 1669631 | 3.95119984161919 | 0.952293389792253 | 0.241013724429085 | 1.93494611432586 | 0 | |  |  |  |
| SIPA1L2 | 1732923 | 4.04318837611886 | 0.951773508045402 | 0.235401722478988 | 1.93424897327308 | 0 | |  |  |  |
| KCNU1 | 3244673 | 3.96973084692317 | 0.95047878657042 | 0.23943154415799 | 1.93251389397878 | 0 | |  |  |  |
| SLC7A2 | 1781400 | 5.2391774893001 | 0.947719094805591 | 0.180890816686609 | 1.92882077452712 | 0 | |  |  |  |
| SMYD3 | 1741954 | 3.00887581296061 | 0.94749320369514 | 0.314899405157851 | 1.9285187915399 | 0 | |  |  |  |
| SLC44A3 | 1658498 | 7.7257750543491 | 0.947244889570048 | 0.122608396297639 | 1.92818688685223 | 0 | |  |  |  |
| MKNK1 | 1750429 | 4.52237189924925 | 0.946993253516859 | 0.209401896751142 | 1.92785060024133 | 0 | |  |  |  |
| IL33 | 1809099 | 2.069614506998 | 0.945912796403998 | 0.457047818908099 | 1.92640734297043 | 0.0848201265005491 | |  |  |  |
| MME | 1678170 | 3.47692610173217 | 0.941916516905847 | 0.270904957236966 | 1.92107855942158 | 0 | |  |  |  |
| CTXN1 | 1759766 | 3.42554530355864 | 0.940505527947544 | 0.27455644126805 | 1.91920061885484 | 0 | |  |  |  |
| G6PD | 2347949 | 6.06492372353304 | 0.940140115857106 | 0.155012685849483 | 1.91871457690995 | 0 | |  |  |  |
| MT1X | 1775170 | 3.03848220260961 | 0.935162206606918 | 0.307772810320808 | 1.91210560697191 | 0 | |  |  |  |
| CKLF | 2414027 | 4.01813955032671 | 0.934541006053174 | 0.232580524978827 | 1.91128246324355 | 0 | |  |  |  |
| FGFR4 | 2410713 | 5.78554155558137 | 0.934032751054656 | 0.161442579243699 | 1.91060924558851 | 0 | |  |  |  |
| 37316 | 1703142 | 5.0867960649666 | 0.933927279837643 | 0.183598333392942 | 1.91046957163749 | 0 | |  |  |  |
| GM2A | 2221046 | 4.72352353378136 | 0.932715606565033 | 0.197461831172112 | 1.90886570315925 | 0 | |  |  |  |
| BTG1 | 1775743 | 4.85395029713108 | 0.932304517963291 | 0.192071294696678 | 1.90832185908879 | 0 | |  |  |  |
| PLCD1 | 1769394 | 7.27709711142111 | 0.931819516461437 | 0.128048245364073 | 1.90768043218551 | 0 | |  |  |  |
| GBP2 | 1774077 | 5.16131283560627 | 0.928802375269258 | 0.179954675264352 | 1.90369502523036 | 0 | |  |  |  |
| GLRX5 | 1796165 | 3.97933765582353 | 0.928763342614142 | 0.233396465176799 | 1.90364352074461 | 0 | |  |  |  |
| CELSR3 | 1691290 | 4.91495503659773 | 0.926900571434932 | 0.188587802845204 | 1.90118717073011 | 0 | |  |  |  |
| GOLPH4 | 2186216 | 2.48450570763054 | 0.926503601027595 | 0.372912647446158 | 1.90066411408929 | 0.0359206925696922 | |  |  |  |
| POLR3GL | 1760667 | 6.57288538726122 | 0.925997006993124 | 0.140881356122196 | 1.89999682402314 | 0 | |  |  |  |
| G6PD | 1697559 | 7.92085766709476 | 0.924575221768512 | 0.116726655196625 | 1.89812528731266 | 0 | |  |  |  |
| BLVRA | 1691436 | 7.14130190555303 | 0.923312889092455 | 0.129291955627096 | 1.89646518760088 | 0 | |  |  |  |
| C13orf15 | 1658494 | 3.93680805093473 | 0.922364478621364 | 0.234292469098757 | 1.8952188837581 | 0 | |  |  |  |
| PARD6G | 1674344 | 3.4815780053162 | 0.921567215878591 | 0.264698138163615 | 1.89417183643469 | 0 | |  |  |  |
| ENO2 | 1765796 | 4.88535169242289 | 0.921530674669548 | 0.188631388830988 | 1.89412386063209 | 0 | |  |  |  |
| ERMP1 | 1751425 | 3.17922734321586 | 0.920506690334298 | 0.28953786280763 | 1.89277994182038 | 0 | |  |  |  |
| GTPBP8 | 2286514 | 4.56190420238837 | 0.920137125833742 | 0.201700229775103 | 1.89229514450128 | 0 | |  |  |  |
| BEX1 | 1702637 | 5.28904517868359 | 0.917517785530924 | 0.173475127274161 | 1.88886263244741 | 0 | |  |  |  |
| STEAP3 | 2383349 | 5.43302930425767 | 0.917320130809277 | 0.168841373649578 | 1.88860386879067 | 0 | |  |  |  |
| FANCE | 2043452 | 4.67352464825043 | 0.916090186932146 | 0.196016979877295 | 1.88699445944447 | 0 | |  |  |  |
| TMEM59L | 3245912 | 3.82855236623294 | 0.914564591454569 | 0.23888000057694 | 1.88500008879617 | 0 | |  |  |  |
| JUP | 1733811 | 4.36661203042801 | 0.912647838943819 | 0.209005937002002 | 1.88249735643746 | 0 | |  |  |  |
| ALDH7A1 | 1763461 | 4.55540282470971 | 0.912137922981254 | 0.200232110766051 | 1.88183211131996 | 0 | |  |  |  |
| PEG10 | 2297626 | 3.84628022461392 | 0.910812473671393 | 0.236803462171771 | 1.88010400692519 | 0 | |  |  |  |
| TNC | 2145670 | 3.13941512960247 | 0.909473736649154 | 0.289695277337953 | 1.87836018904884 | 0 | |  |  |  |
| SEMA6B | 1712914 | 5.39409876428538 | 0.909436364958086 | 0.168598389591884 | 1.87831153248212 | 0 | |  |  |  |
| CLDN23 | 1725338 | 2.41681498063622 | 0.90895966827647 | 0.376098160413252 | 1.87769100150466 | 0.0359206925696922 | |  |  |  |
| LOC346887 | 1714741 | 3.87704946037087 | 0.908820977778509 | 0.234410467823016 | 1.8775105022476 | 0 | |  |  |  |
| C1orf57 | 1657446 | 5.10885649573282 | 0.907951463099673 | 0.177721073954228 | 1.87637926450725 | 0 | |  |  |  |
| IL11RA | 1664912 | 4.22410538093023 | 0.907753808378027 | 0.214898475894113 | 1.87612221100238 | 0 | |  |  |  |
| HMGCS1 | 1797728 | 3.17750256309831 | 0.906481509917684 | 0.285281126267226 | 1.87446840675968 | 0 | |  |  |  |
| UCHL3 | 1660111 | 4.21397185180617 | 0.905164365428062 | 0.214800762145598 | 1.87275784495483 | 0 | |  |  |  |
| OSR1 | 2197128 | 4.43080324717391 | 0.904887814914161 | 0.204226584760071 | 1.87239889001731 | 0 | |  |  |  |
| C16orf48 | 1787324 | 3.21391973929369 | 0.904488353060752 | 0.281428419634253 | 1.87188052101303 | 0 | |  |  |  |
| THAP10 | 1767542 | 3.66315725745059 | 0.904114636150077 | 0.246812946485215 | 1.87139569034056 | 0 | |  |  |  |
| METTL5 | 1691570 | 3.91552998297538 | 0.902859777812234 | 0.230584309592276 | 1.86976865522806 | 0 | |  |  |  |
| HADH | 1719906 | 3.72618794875802 | 0.902481078009417 | 0.242199559018547 | 1.86927791527585 | 0 | |  |  |  |
| LOC387856 | 1672121 | 5.63180187566363 | 0.899611762617457 | 0.159737821478574 | 1.8655638817419 | 0 | |  |  |  |
| HABP4 | 1792384 | 3.68859582804456 | 0.89874640034874 | 0.243655429395525 | 1.86444520837701 | 0 | |  |  |  |
| MDK | 2349393 | 3.38534472669214 | 0.898372683438066 | 0.265371108695296 | 1.86396230350461 | 0 | |  |  |  |
| HPCAL1 | 1764850 | 3.35865826404308 | 0.89469863096512 | 0.266385729248947 | 1.85922146612894 | 0 | |  |  |  |
| CBR4 | 1813400 | 6.50959731943192 | 0.893308404057408 | 0.13722944142655 | 1.85743072403338 | 0 | |  |  |  |
| C7orf10 | 1763011 | 4.74780614685113 | 0.893261066582056 | 0.188141857302765 | 1.85736977931802 | 0 | |  |  |  |
| AKR1B1 | 1701731 | 3.43997874861603 | 0.891657405794249 | 0.259204335536369 | 1.85530632435924 | 0 | |  |  |  |
| ASMTL | 1804384 | 5.43970324497981 | 0.891346805517378 | 0.163859454344312 | 1.85490693528928 | 0 | |  |  |  |
| TMEM218 | 2395856 | 5.05460447741735 | 0.890291262865228 | 0.17613470388095 | 1.85355029563386 | 0 | |  |  |  |
| TMEM106C | 1692511 | 2.91699598715103 | 0.888030690796657 | 0.304433291889434 | 1.85064822493926 | 0 | |  |  |  |
| CEND1 | 1720482 | 4.79066417799422 | 0.887422777955293 | 0.185240030397381 | 1.84986857588459 | 0 | |  |  |  |
| C1orf24 | 1667966 | 4.0816750930322 | 0.88687133789154 | 0.217281218538318 | 1.84916163735151 | 0 | |  |  |  |
| TMEM132A | 2317923 | 4.89928487276746 | 0.88515971444065 | 0.180671207620685 | 1.84696907998769 | 0 | |  |  |  |
| RPAIN | 2323774 | 3.63579313719506 | 0.883452243399877 | 0.242987488579024 | 1.84478443205552 | 0 | |  |  |  |
| CHCHD10 | 1740170 | 5.84787963699895 | 0.882283755192502 | 0.150872420425752 | 1.84329088277602 | 0 | |  |  |  |
| BOLA2 | 2298511 | 4.34643014712527 | 0.882053711671931 | 0.202937510051857 | 1.84299698607112 | 0 | |  |  |  |
| NBL1 | 1789599 | 4.22306839907156 | 0.880798853334086 | 0.20856845546895 | 1.84139464145525 | 0 | |  |  |  |
| RBPMS2 | 1808238 | 2.99488157540743 | 0.879799783459549 | 0.293767803937242 | 1.84011991255124 | 0 | |  |  |  |
| GALNT12 | 1735157 | 2.83585515627543 | 0.879299833281267 | 0.310065142549849 | 1.83948234960952 | 0 | |  |  |  |
| SLC25A4 | 2211780 | 5.80717542335592 | 0.878578144402654 | 0.151291820954658 | 1.83856240330672 | 0 | |  |  |  |
| NDUFA12 | 1737738 | 3.13849682475371 | 0.875662322017367 | 0.279006916658609 | 1.83485024831541 | 0 | |  |  |  |
|  | 1903568 | 2.46920912833332 | 0.874360956686195 | 0.354105671590634 | 1.83319589045765 | 0.0359206925696922 | |  |  |  |
|  | 1890253 | 3.81631159331037 | 0.874318602102984 | 0.229100423465312 | 1.83314207235615 | 0 | |  |  |  |
| C8orf13 | 1687213 | 4.96371523712695 | 0.87282456494231 | 0.1758409826603 | 1.83124467570327 | 0 | |  |  |  |
| GSTM2 | 1713162 | 5.79320295723895 | 0.872625249256616 | 0.150629152076611 | 1.83099169737728 | 0 | |  |  |  |
| PDGFD | 2376859 | 3.2118604857659 | 0.871143669326296 | 0.271227119978271 | 1.82911232023066 | 0 | |  |  |  |
| LRRC1 | 1755383 | 3.68707698341738 | 0.870346406583523 | 0.236053223325117 | 1.82810179465204 | 0 | |  |  |  |
| ATP5J | 1652806 | 3.51924349151954 | 0.869905420628927 | 0.247185346147595 | 1.82754308751373 | 0 | |  |  |  |
| CDK5RAP2 | 2415529 | 4.57871904221549 | 0.869083243425442 | 0.189809253507925 | 1.82650188604312 | 0 | |  |  |  |
| C5orf13 | 1680738 | 5.53262230197115 | 0.868672154823701 | 0.157009119258731 | 1.82598150777933 | 0 | |  |  |  |
| ACYP1 | 1694233 | 3.26758966158298 | 0.864732348103164 | 0.264639210446101 | 1.82100179966099 | 0 | |  |  |  |
| CTNS | 1807719 | 4.00073320034408 | 0.863764836545527 | 0.215901634348234 | 1.81978099443179 | 0 | |  |  |  |
| IRF2BP2 | 2394561 | 2.64750317709188 | 0.863761514617433 | 0.326255138083053 | 1.81977680423602 | 0 | |  |  |  |
| MOXD1 | 1761788 | 4.85100499403042 | 0.862853797765505 | 0.177871141923647 | 1.81863219468239 | 0 | |  |  |  |
| SPOCK1 | 1746013 | 3.27195369530826 | 0.862027468151901 | 0.263459556101905 | 1.81759083853756 | 0 | |  |  |  |
| DDIT4L | 1696537 | 5.49751537764727 | 0.861166258293302 | 0.15664644828367 | 1.81650616021651 | 0 | |  |  |  |
| ADK | 2358626 | 4.80124025482221 | 0.861084871054977 | 0.179346340810613 | 1.81640368793679 | 0 | |  |  |  |
| POLR1D | 1767422 | 3.9715884688227 | 0.860549210149676 | 0.216676329107373 | 1.81572939724504 | 0 | |  |  |  |
| GPR37 | 2218935 | 3.71393976818639 | 0.860528448099084 | 0.231702316626234 | 1.81570326698654 | 0 | |  |  |  |
| C2CD2 | 1652512 | 4.12838416705383 | 0.85995873743081 | 0.208303952014356 | 1.81498639940516 | 0 | |  |  |  |
| C11orf75 | 1798270 | 5.33805284763864 | 0.859794301990114 | 0.161068900314551 | 1.81477954274289 | 0 | |  |  |  |
| GLCE | 1714349 | 2.74991228084763 | 0.859531869670618 | 0.312567013739681 | 1.81444945670437 | 0 | |  |  |  |
| PODXL2 | 1657347 | 3.28175215457294 | 0.857429919668577 | 0.261271991083725 | 1.81180779998422 | 0 | |  |  |  |
| HABP4 | 2107991 | 4.29896396071163 | 0.857053711311831 | 0.199362850943733 | 1.81133540051539 | 0 | |  |  |  |
| GSR | 1775182 | 3.3734314685547 | 0.855421814135219 | 0.253576164836606 | 1.80928767602105 | 0 | |  |  |  |
| FIG4 | 1800889 | 4.92518449823453 | 0.854630364766611 | 0.173522507648792 | 1.80829538947396 | 0 | |  |  |  |
| ANG | 1760727 | 6.58849880441793 | 0.854194361704157 | 0.129649315733559 | 1.80774897933111 | 0 | |  |  |  |
| LOC100133578 | 3246330 | 6.18391913035776 | 0.853698563935995 | 0.138051379059125 | 1.80712783356309 | 0 | |  |  |  |
| FAM167A | 3248511 | 4.25517067334297 | 0.85188977408833 | 0.200201082279754 | 1.80486355306409 | 0 | |  |  |  |
| CCDC128 | 1667356 | 3.19543161156346 | 0.850545223691924 | 0.266175380068851 | 1.80318225558485 | 0 | |  |  |  |
| AGBL5 | 2308689 | 5.34209979324148 | 0.850431447654674 | 0.15919422709598 | 1.80304005585718 | 0 | |  |  |  |
| RPA3 | 1716895 | 3.50149756116431 | 0.850363348128727 | 0.242857044243083 | 1.80295494897618 | 0 | |  |  |  |
| IGFBP3 | 2396875 | 4.58256984608074 | 0.848780449391514 | 0.185219315340592 | 1.80097786443461 | 0 | |  |  |  |
| MAPRE3 | 1734290 | 4.73005376417608 | 0.847566284672833 | 0.179187452601919 | 1.79946280835738 | 0 | |  |  |  |
| STS-1 | 1653856 | 3.20443538739755 | 0.847528082499741 | 0.264485932789568 | 1.79941515970151 | 0 | |  |  |  |
| TRIM6 | 1656910 | 5.19414682470554 | 0.847165161855376 | 0.163099964334066 | 1.79896256039363 | 0 | |  |  |  |
| LARGE | 1662038 | 3.5920575301183 | 0.846641127698407 | 0.235698097984116 | 1.79830923685002 | 0 | |  |  |  |
| GLIS3 | 2402600 | 3.08248590917373 | 0.846052315943588 | 0.274470781334529 | 1.79757543688694 | 0 | |  |  |  |
| TXNDC12 | 1783753 | 3.12084336357617 | 0.845568144923759 | 0.270942192995813 | 1.79697226859332 | 0 | |  |  |  |
| FBXO2 | 1803211 | 2.71886777757648 | 0.844995942809414 | 0.310789641842245 | 1.79625969426498 | 0 | |  |  |  |
| NES | 1738147 | 4.37108342238375 | 0.842910602447849 | 0.19283791247987 | 1.79366517023207 | 0 | |  |  |  |
| SNF8 | 1766171 | 3.84409504806419 | 0.841336839012897 | 0.218864733700218 | 1.79170961794181 | 0 | |  |  |  |
| HSD11B1L | 2317457 | 4.43721014588033 | 0.841172403572199 | 0.189572360992002 | 1.79150541416846 | 0 | |  |  |  |
| TRAPPC6A | 1775703 | 3.83896933108672 | 0.840663318091657 | 0.218981514461249 | 1.79087335508923 | 0 | |  |  |  |
| SLCO2B1 | 2087656 | 5.19327054405727 | 0.840275482986579 | 0.161800829719552 | 1.79039198502426 | 0 | |  |  |  |
| GRIN3B | 1699854 | 4.09250252351437 | 0.839798786304963 | 0.205204219540419 | 1.78980049970924 | 0 | |  |  |  |
| LOC401052 | 1791423 | 4.81795569469666 | 0.838328002640951 | 0.174000770402214 | 1.78797678236945 | 0 | |  |  |  |
| FAM50B | 2153466 | 4.73770249541699 | 0.837926049341471 | 0.176863374209765 | 1.78747869854796 | 0 | |  |  |  |
| TMEM97 | 1753890 | 5.6538902154846 | 0.837274951434873 | 0.148088293108661 | 1.78667217948153 | 0 | |  |  |  |
| CREG1 | 1680624 | 4.3507196199353 | 0.837067330928942 | 0.192397443193867 | 1.7864150751867 | 0 | |  |  |  |
| TNFRSF1B | 1764788 | 4.21041223661203 | 0.836631327866487 | 0.198705324051522 | 1.78587527658847 | 0 | |  |  |  |
| SDC2 | 1784553 | 2.93864097685805 | 0.835090783712484 | 0.284175845327438 | 1.78396929420626 | 0 | |  |  |  |
| TNFRSF21 | 1699695 | 2.84666897389421 | 0.834502802439689 | 0.293150629768553 | 1.78324237217234 | 0 | |  |  |  |
| ELOVL4 | 1737965 | 3.53027502565419 | 0.834085069981756 | 0.236266314641362 | 1.78272610895458 | 0 | |  |  |  |
| SEL1L3 | 1797822 | 5.18867643900096 | 0.831768855617596 | 0.160304629782956 | 1.77986627868706 | 0 | |  |  |  |
| NRGN | 1705686 | 3.83030368255623 | 0.830754006584609 | 0.216889854025932 | 1.77861469026436 | 0 | |  |  |  |
| LTA4H | 1690342 | 6.43653390778933 | 0.829700124896505 | 0.128904801370257 | 1.77731589535085 | 0 | |  |  |  |
| HLTF | 1673820 | 4.11699403230934 | 0.827160510867964 | 0.200913701690256 | 1.77418999154302 | 0 | |  |  |  |
| GTPBP8 | 2393243 | 3.28231853606995 | 0.827156358457845 | 0.252003682570136 | 1.77418488502108 | 0 | |  |  |  |
| HIST1H2BD | 1758623 | 3.49171464178012 | 0.824646641782158 | 0.236172404215066 | 1.77110119058532 | 0 | |  |  |  |
| MCOLN2 | 1660462 | 4.76168866461614 | 0.823289634155396 | 0.172898669388702 | 1.76943606539642 | 0 | |  |  |  |
| ST3GAL5 | 1713496 | 5.34261243423157 | 0.822028962443386 | 0.153862735237245 | 1.76789055259112 | 0 | |  |  |  |
| NUDT6 | 1780659 | 3.68467398589095 | 0.821696769633898 | 0.223003927289164 | 1.76748352761016 | 0 | |  |  |  |
| CARD16 | 1726591 | 3.68306983542079 | 0.820741715306618 | 0.222841746690054 | 1.76631385270975 | 0 | |  |  |  |
| PNMAL1 | 3299393 | 4.8893060657474 | 0.820367167913921 | 0.167788057626643 | 1.765855348064 | 0 | |  |  |  |
| KDELC1 | 1712505 | 4.16161669500336 | 0.819525889623889 | 0.196924885131264 | 1.76482592558171 | 0 | |  |  |  |
| XRCC6BP1 | 1767481 | 4.8105656587273 | 0.8177478276111 | 0.169989952455497 | 1.76265219036144 | 0 | |  |  |  |
| KCNK1 | 1718863 | 5.23716059417191 | 0.817596679882783 | 0.156114494711625 | 1.76246753115683 | 0 | |  |  |  |
| LOC730101 | 3305735 | 3.80050309188193 | 0.817526088910767 | 0.215109965482476 | 1.76238129584798 | 0 | |  |  |  |
| TMEM123 | 1724139 | 2.77672527022385 | 0.816875821486193 | 0.294186763899887 | 1.76158711490225 | 0 | |  |  |  |
| LRRC49 | 1763941 | 3.29199731596224 | 0.814023946216732 | 0.247273575306302 | 1.75810830310713 | 0 | |  |  |  |
| RNF13 | 2339748 | 3.90405810178323 | 0.813909339697459 | 0.208477773249762 | 1.75796864596234 | 0 | |  |  |  |
| TMEM99 | 1758173 | 3.29067439713895 | 0.81232561047822 | 0.24685687869468 | 1.75603988164791 | 0 | |  |  |  |
| SERPINI1 | 1814333 | 3.48571391361893 | 0.811649598110911 | 0.232850319396477 | 1.75521723613873 | 0 | |  |  |  |
| LOC401074 | 1813179 | 4.43201316005155 | 0.810946179836819 | 0.1829746777709 | 1.75436164926712 | 0 | |  |  |  |
| HSD11B1L | 1708987 | 4.56134702900266 | 0.810104901546788 | 0.177602119811506 | 1.75333892714789 | 0 | |  |  |  |
| KLRAQ1 | 3271092 | 4.17624802995166 | 0.809521072684113 | 0.193839318660746 | 1.75262953067415 | 0 | |  |  |  |
| FLJ20021 | 1754114 | 2.65117888643096 | 0.80874623295598 | 0.305051551630574 | 1.75168848468865 | 0 | |  |  |  |
| HIST3H2A | 1779648 | 3.26283058821957 | 0.806572031017876 | 0.247200094890001 | 1.74905060486754 | 0 | |  |  |  |
| APLP1 | 1763834 | 4.43424315344445 | 0.805600367050121 | 0.181677084267322 | 1.74787300515135 | 0 | |  |  |  |
| C20orf3 | 1674394 | 2.78597594018579 | 0.805184295556237 | 0.289013370123556 | 1.74736899338569 | 0 | |  |  |  |
| MANBA | 1800733 | 4.82125048024659 | 0.805038961202085 | 0.166977211513995 | 1.7471929756231 | 0 | |  |  |  |
| OGFRL1 | 1715809 | 4.05783857610519 | 0.804432709324769 | 0.198241673304038 | 1.74645892132541 | 0 | |  |  |  |
| B4GALT4 | 1661500 | 4.31737178017732 | 0.804096364105161 | 0.18624672718645 | 1.74605180504351 | 0 | |  |  |  |
| CSRP1 | 1811921 | 4.89451988862387 | 0.801623188638517 | 0.163779738744488 | 1.74306115690928 | 0 | |  |  |  |
| KIAA0367 | 1810628 | 5.9488548677914 | 0.80036749981865 | 0.134541439925193 | 1.74154469632291 | 0 | |  |  |  |
| FYCO1 | 1709032 | 4.07405961100434 | 0.80011088087332 | 0.196391549773145 | 1.74123494713433 | 0 | |  |  |  |
| RTN2 | 1749115 | 6.79917375073585 | 0.799262128245076 | 0.117552831792036 | 1.74021086161685 | 0 | |  |  |  |
| CKLF | 1712389 | 3.27065638269481 | 0.799097692804379 | 0.244323340425622 | 1.7400125272323 | 0 | |  |  |  |
| NOTCH3 | 1658926 | 4.21111692131916 | 0.797083773896854 | 0.18928084610084 | 1.7375852648482 | 0 | |  |  |  |
|  | 1881909 | 2.57973663180908 | 0.795049092938735 | 0.308190023406069 | 1.73513641761829 | 0 | |  |  |  |
| CRISPLD2 | 1790689 | 5.73311869991938 | 0.793861503644813 | 0.138469399500829 | 1.73370868585317 | 0 | |  |  |  |
| BCKDHB | 1764166 | 5.9755897225881 | 0.793677136635547 | 0.132819884476907 | 1.73348714335576 | 0 | |  |  |  |
| NICN1 | 1807211 | 3.39172031414131 | 0.792962922095145 | 0.233793723730402 | 1.73262918288657 | 0 | |  |  |  |
| OAF | 1668345 | 5.56155302526807 | 0.791355939379244 | 0.142290460197689 | 1.73070032407581 | 0 | |  |  |  |
| APOO | 1721623 | 5.87522275677658 | 0.791144166463195 | 0.134657731155925 | 1.73044629356693 | 0 | |  |  |  |
| SH3GLB2 | 2158336 | 5.64005547203985 | 0.788741581968567 | 0.139846422766353 | 1.72756690231271 | 0 | |  |  |  |
| NT5DC2 | 1708743 | 4.90654880520013 | 0.786368064344772 | 0.160269080277231 | 1.72472705104118 | 0 | |  |  |  |
| RAB8B | 2173004 | 3.47877843893357 | 0.786224390954666 | 0.226005882454442 | 1.72455529953644 | 0 | |  |  |  |
| CRIP2 | 1694432 | 5.1806613593715 | 0.785584089314378 | 0.15163779965917 | 1.72379007157814 | 0 | |  |  |  |
| ATOX1 | 1670609 | 3.93174509778516 | 0.784923025623495 | 0.199637312720415 | 1.72300038702326 | 0 | |  |  |  |
| RNF130 | 1729294 | 4.63060007509736 | 0.784424736409262 | 0.169400234027502 | 1.72240538653116 | 0 | |  |  |  |
| AVPI1 | 1671731 | 2.61620745794092 | 0.784090052153703 | 0.299704845567109 | 1.7220058598897 | 0 | |  |  |  |
| SYNJ2BP | 1697793 | 2.38075784466505 | 0.783515358593286 | 0.329103340076789 | 1.72132004024696 | 0.0359206925696922 | |  |  |  |
| CLEC16A | 1781752 | 3.98040226605127 | 0.781364410151847 | 0.196302875419422 | 1.71875559532049 | 0 | |  |  |  |
| LAYN | 1716397 | 3.72380637053562 | 0.780660991877754 | 0.209640597334675 | 1.7179177818051 | 0 | |  |  |  |
|  | 1853167 | 2.91019373800753 | 0.779901100826049 | 0.267989409309914 | 1.71701316469783 | 0 | |  |  |  |
| CRYZ | 1672389 | 3.58793387091158 | 0.779764071292134 | 0.217329554932411 | 1.71685008772476 | 0 | |  |  |  |
| RGS17 | 1725485 | 3.00422519215119 | 0.778834761907589 | 0.259246465259118 | 1.71574453797298 | 0 | |  |  |  |
| LOC387763 | 1677402 | 3.8197391541825 | 0.777697832017114 | 0.20359972255319 | 1.71439296139612 | 0 | |  |  |  |
| B3GNT1 | 3246214 | 3.3150392648096 | 0.776702914552696 | 0.234296746586893 | 1.71321108203419 | 0 | |  |  |  |
| RCAN2 | 2120210 | 3.29596012943866 | 0.775424802718188 | 0.235265225386768 | 1.71169398684421 | 0 | |  |  |  |
| SERPINB2 | 2150856 | 1.70669342513622 | 0.775368329940576 | 0.454310257789086 | 1.71162698569758 | 0.200308840224877 | |  |  |  |
|  | 1909886 | 4.17470617611836 | 0.775252062457254 | 0.185702185914814 | 1.71148905041841 | 0 | |  |  |  |
| PIGF | 1808938 | 4.05536810833565 | 0.775033645685015 | 0.191113019824751 | 1.71122995919825 | 0 | |  |  |  |
| FLJ22536 | 3247023 | 2.87939873878785 | 0.773703213483013 | 0.268703046598097 | 1.70965261544748 | 0 | |  |  |  |
| PGM2L1 | 1716265 | 3.85471515253011 | 0.772540538649803 | 0.200414429622052 | 1.70827535327382 | 0 | |  |  |  |
| SV2A | 1702009 | 5.23899913516692 | 0.772536386239684 | 0.147458773385552 | 1.70827043646919 | 0 | |  |  |  |
| RAET1G | 1659836 | 5.69691168078993 | 0.772176787523412 | 0.135543050478947 | 1.70784469486245 | 0 | |  |  |  |
| SLC45A1 | 2324162 | 3.00279489777789 | 0.772105366069371 | 0.257128905687411 | 1.70776014911389 | 0 | |  |  |  |
| RICH2 | 3245564 | 3.68039674106943 | 0.771770681813811 | 0.209697686448215 | 1.70736401956035 | 0 | |  |  |  |
| LOC644761 | 3290211 | 4.98784057596585 | 0.771707565180009 | 0.15471776882736 | 1.7072893255264 | 0 | |  |  |  |
| LRRC20 | 1690523 | 3.4958110486507 | 0.771210936929822 | 0.220610017588763 | 1.70670171541754 | 0 | |  |  |  |
| SLIT2 | 1676449 | 1.8786671554246 | 0.770734240248206 | 0.410255876365716 | 1.70613787855374 | 0.200308840224877 | |  |  |  |
| PLD1 | 1719696 | 2.61725760651858 | 0.770650361563811 | 0.294449564171451 | 1.70603868611425 | 0 | |  |  |  |
| MRPS33 | 1772722 | 3.10193215749135 | 0.768425500222262 | 0.247724792551142 | 1.70340973534137 | 0 | |  |  |  |
| PID1 | 1671891 | 4.48083192425393 | 0.766721351109583 | 0.171111383794482 | 1.70139881112517 | 0 | |  |  |  |
| C12orf60 | 2234709 | 6.61003774831719 | 0.766719690145536 | 0.115993239273214 | 1.70139685231853 | 0 | |  |  |  |
| SHC4 | 1807050 | 4.90148735296255 | 0.766032881511917 | 0.156285801910498 | 1.70058707900362 | 0 | |  |  |  |
| DGCR6 | 1663685 | 3.20273963502182 | 0.765448222167218 | 0.238997954687629 | 1.69989804725331 | 0 | |  |  |  |
| PLTP | 1773389 | 3.71149608985919 | 0.763052281528779 | 0.205591562823855 | 1.69707730233268 | 0 | |  |  |  |
| GLIPR2 | 1652631 | 4.67861699158617 | 0.762161174317327 | 0.162903092022272 | 1.69602939488679 | 0 | |  |  |  |
| EVI5L | 1747281 | 4.64711287443605 | 0.761579006418697 | 0.163882183841105 | 1.69534513754125 | 0 | |  |  |  |
| SF3B5 | 1689389 | 3.69045086026386 | 0.761156291068624 | 0.206250217084371 | 1.69484846748081 | 0 | |  |  |  |
| DGCR6 | 1791396 | 3.41208024482826 | 0.760798353316398 | 0.222971999110968 | 1.69442802172907 | 0 | |  |  |  |
| FLJ22795 | 2150802 | 3.32638660255497 | 0.760550869673329 | 0.228641754716411 | 1.69413738008804 | 0 | |  |  |  |
| GDE1 | 1728698 | 3.5000208805551 | 0.759375737609763 | 0.216963202085048 | 1.69275800016906 | 0 | |  |  |  |
| C14orf179 | 1756877 | 4.41279306638894 | 0.75890402382029 | 0.171978158142211 | 1.69220461449755 | 0 | |  |  |  |
| ALDH7A1 | 1687508 | 2.57602615181521 | 0.757409156177591 | 0.294022308602682 | 1.69045212228456 | 0 | |  |  |  |
| ZMIZ2 | 1760718 | 3.34927237689371 | 0.755998167219287 | 0.225720121311972 | 1.68879962941747 | 0 | |  |  |  |
| ITPR1 | 1789505 | 4.23075055739761 | 0.755803003943712 | 0.178645134873803 | 1.68857118933411 | 0 | |  |  |  |
| PALM | 1812031 | 3.58587618298694 | 0.755258207736151 | 0.210620269411266 | 1.68793366478272 | 0 | |  |  |  |
| GTPBP8 | 1660698 | 5.43210253078258 | 0.754964217099754 | 0.138981952719326 | 1.68758973476257 | 0 | |  |  |  |
| FAM129A | 1810725 | 3.48611347788758 | 0.753811508050827 | 0.216232636382107 | 1.68624189411328 | 0 | |  |  |  |
| IFT20 | 3235216 | 3.64747467336319 | 0.753802372748565 | 0.206664182825844 | 1.6862312166796 | 0 | |  |  |  |
| AKAP12 | 1684836 | 3.14446700225253 | 0.753715172136075 | 0.239695685022661 | 1.68612929912458 | 0 | |  |  |  |
| PPARG | 1800225 | 3.20954067306265 | 0.753265881361242 | 0.234695851553877 | 1.6856042786814 | 0 | |  |  |  |
| CASP1 | 2326512 | 3.03696778366833 | 0.752940332407943 | 0.247925031163311 | 1.68522395935927 | 0 | |  |  |  |
| CDK5 | 1781987 | 4.88944578618158 | 0.751651424307127 | 0.15372937080751 | 1.68371904753769 | 0 | |  |  |  |
| FEZ1 | 1779071 | 6.13189639374048 | 0.750716301548416 | 0.12242807988647 | 1.6826280520095 | 0 | |  |  |  |
| PUS3 | 1694147 | 3.23262687259991 | 0.750536917431292 | 0.232175548558642 | 1.6824188477254 | 0 | |  |  |  |
| SCPEP1 | 1767470 | 2.77261404881037 | 0.749632522507459 | 0.270370310945044 | 1.68136450553813 | 0 | |  |  |  |
| ATG4C | 1700923 | 3.11418680166551 | 0.749459782246525 | 0.240659867239083 | 1.68116320037794 | 0 | |  |  |  |
| ZNF365 | 1658094 | 4.55578080851833 | 0.749292024877733 | 0.164470604792206 | 1.68096772516628 | 0 | |  |  |  |
| SPAG16 | 1768732 | 4.64266539937668 | 0.74795494881954 | 0.161104642372022 | 1.67941054195067 | 0 | |  |  |  |
| GABARAPL2 | 1796458 | 5.31704901379074 | 0.747033944255235 | 0.140497848020145 | 1.67833876231521 | 0 | |  |  |  |
| RAD51C | 1695386 | 3.69634233132094 | 0.746544790343262 | 0.201968520073863 | 1.67776980852589 | 0 | |  |  |  |
| COMMD6 | 1777378 | 3.1358371242353 | 0.745965113890703 | 0.23788388374049 | 1.67709581418831 | 0 | |  |  |  |
| SORT1 | 1707077 | 4.74845460342101 | 0.74552495841813 | 0.157003703453544 | 1.67658422283731 | 0 | |  |  |  |
| DFNA5 | 1670145 | 5.91914056069768 | 0.744736830977618 | 0.125818406125135 | 1.67566857459683 | 0 | |  |  |  |
| PECR | 1814120 | 3.90311183549993 | 0.742432243361791 | 0.19021546772223 | 1.67299396757161 | 0 | |  |  |  |
| NQO2 | 1712918 | 5.95334992619377 | 0.742335907447039 | 0.124692134117781 | 1.67288225718324 | 0 | |  |  |  |
| CSDC2 | 1806787 | 5.72491896876476 | 0.742168980560271 | 0.129638338046277 | 1.67268870770494 | 0 | |  |  |  |
| SLC27A1 | 1787718 | 4.44809993841342 | 0.741625845316756 | 0.166728683164723 | 1.67205910465488 | 0 | |  |  |  |
| HSD17B4 | 1673795 | 4.02963475130992 | 0.740634249780432 | 0.18379686882035 | 1.67091025709087 | 0 | |  |  |  |
| SLC2A12 | 1766261 | 3.1650191557471 | 0.73939517060104 | 0.233614753723822 | 1.66947578811856 | 0 | |  |  |  |
| KCNG1 | 1673769 | 6.33690209312195 | 0.738914321509304 | 0.116604976793206 | 1.66891944590631 | 0 | |  |  |  |
| HTRA1 | 1676563 | 5.44238764718371 | 0.73821505564533 | 0.135641763046287 | 1.66811072640055 | 0 | |  |  |  |
| GNS | 1744517 | 3.08802954507388 | 0.737020822495219 | 0.238670262618095 | 1.66673047015547 | 0 | |  |  |  |
| EVL | 1730622 | 5.59684334438943 | 0.735572461845849 | 0.131426308828748 | 1.66505803390968 | 0 | |  |  |  |
| LOC730525 | 1651610 | 2.88752696281833 | 0.735261861568976 | 0.254633764822523 | 1.66469959931003 | 0 | |  |  |  |
| ACSF2 | 3236756 | 4.49420055035757 | 0.735115696732802 | 0.163569847071981 | 1.66453095090418 | 0 | |  |  |  |
| NR2F1 | 1786197 | 4.32152940328222 | 0.73484329862902 | 0.170042415555684 | 1.66421669719189 | 0 | |  |  |  |
| C9orf89 | 1659189 | 3.89336087993417 | 0.734735335965937 | 0.188714932579885 | 1.66409216183353 | 0 | |  |  |  |
| C18orf45 | 1690442 | 5.46295085032694 | 0.73408922095148 | 0.13437595194684 | 1.66334706035877 | 0 | |  |  |  |
| AHCYL1 | 1770412 | 3.90018118857156 | 0.732698163561747 | 0.187862596155461 | 1.66174402159048 | 0 | |  |  |  |
| FLRT2 | 1769615 | 4.39062298915586 | 0.732462306667009 | 0.166824231658258 | 1.66147237599799 | 0 | |  |  |  |
| METTL7A | 1656285 | 6.18233070082793 | 0.730797190209448 | 0.11820739225606 | 1.65955585933989 | 0 | |  |  |  |
| ARRB1 | 2325168 | 5.48842323457861 | 0.730613653682206 | 0.133119043932169 | 1.65934474767957 | 0 | |  |  |  |
| OSBPL9 | 2313851 | 5.46184697713993 | 0.729822204313598 | 0.133621869555885 | 1.65843469589956 | 0 | |  |  |  |
| ADA | 1803686 | 5.05701776878441 | 0.729683513815637 | 0.144291269514569 | 1.65827527337995 | 0 | |  |  |  |
| STK4 | 1711383 | 2.84804008676933 | 0.729302322566748 | 0.256071649396631 | 1.65783717904816 | 0 | |  |  |  |
| PDGFD | 1803094 | 6.29535364735366 | 0.724484696347137 | 0.115082446027744 | 1.65231035657787 | 0 | |  |  |  |
| FDFT1 | 2144088 | 3.94534049803259 | 0.723922460017079 | 0.183487955064481 | 1.65166655599017 | 0 | |  |  |  |
| NUAK1 | 1689318 | 2.92534400561723 | 0.7238111754259 | 0.247427712445456 | 1.65153915696031 | 0 | |  |  |  |
| PTRH1 | 1763842 | 4.0596867260254 | 0.723460712011889 | 0.17820604416937 | 1.65113800928996 | 0 | |  |  |  |
| TCEA2 | 1665526 | 4.52574450319959 | 0.721901897253364 | 0.159510086515709 | 1.64935493786111 | 0 | |  |  |  |
| NUAK1 | 2079786 | 3.32996619516433 | 0.721462572262815 | 0.216657626528011 | 1.64885275790883 | 0 | |  |  |  |
| MAP1LC3A | 1776188 | 5.08683092522159 | 0.720643716987425 | 0.141668501977198 | 1.64791715568989 | 0 | |  |  |  |
| CCDC74B | 3251383 | 3.57179453894835 | 0.720485925402917 | 0.201715389154229 | 1.64773692824589 | 0 | |  |  |  |
| RNF24 | 1717809 | 3.98445855244155 | 0.72042197828709 | 0.180807999080738 | 1.64766389431561 | 0 | |  |  |  |
| SIL1 | 1678729 | 3.14830865112596 | 0.719242693813405 | 0.228453678947957 | 1.64631761498279 | 0 | |  |  |  |
| PAQR8 | 1694589 | 2.7927283282624 | 0.718810012679047 | 0.257386300487839 | 1.64582393908767 | 0 | |  |  |  |
| SYNGR1 | 1721712 | 4.44379280508282 | 0.716528678559882 | 0.161242593880686 | 1.64322345379426 | 0 | |  |  |  |
| SLC25A4 | 1681670 | 4.32726873747187 | 0.716070252482788 | 0.165478572264763 | 1.64270139141065 | 0 | |  |  |  |
| KIFAP3 | 2119224 | 2.6009407996258 | 0.71570484039235 | 0.275171522741048 | 1.64228537352732 | 0 | |  |  |  |
| THRA | 1718182 | 3.62969017384958 | 0.71560186062141 | 0.197152326051691 | 1.64216815115464 | 0 | |  |  |  |
| B3GNT6 | 1715384 | 3.21692164771921 | 0.715314513841201 | 0.22235994288153 | 1.6418411071652 | 0 | |  |  |  |
| C7orf68 | 1659990 | 5.22042086116339 | 0.715276311668111 | 0.137015066541725 | 1.64179763223393 | 0 | |  |  |  |
| COX7A2L | 3237665 | 2.92577552693009 | 0.714566249537828 | 0.244231398807138 | 1.64098977509621 | 0 | |  |  |  |
| RARA | 1659206 | 4.27284680603941 | 0.714417593255583 | 0.167199440018724 | 1.64082069510035 | 0 | |  |  |  |
| SRPX2 | 1676213 | 4.69352242738004 | 0.714213294677747 | 0.152169997209628 | 1.64058835660107 | 0 | |  |  |  |
| TUFT1 | 1781374 | 3.54455603686553 | 0.713542265202578 | 0.20130652690529 | 1.63982545998757 | 0 | |  |  |  |
| CCND2 | 1667081 | 2.93114316189792 | 0.713227512515588 | 0.243327423166111 | 1.63946773839282 | 0 | |  |  |  |
| RPS6KA2 | 1702501 | 3.99493560161422 | 0.713087161053579 | 0.178497786238518 | 1.63930825181028 | 0 | |  |  |  |
| PXMP2 | 1799015 | 4.36926735263916 | 0.713044806470369 | 0.163195508290348 | 1.63926012577074 | 0 | |  |  |  |
| CCRK | 1686804 | 4.21637999732306 | 0.713034840686085 | 0.169110668663352 | 1.63924880220207 | 0 | |  |  |  |
| C2orf89 | 3235176 | 3.53807786633178 | 0.712515789421258 | 0.201384993869562 | 1.63865914111729 | 0 | |  |  |  |
| TCTN3 | 3244526 | 4.58847864515957 | 0.710940365022256 | 0.154940323362349 | 1.63687070039596 | 0 | |  |  |  |
| ABLIM3 | 1656940 | 3.56989620862264 | 0.710143102279487 | 0.198925419894344 | 1.63596638211362 | 0 | |  |  |  |
| CCNC | 1676423 | 3.27917455919044 | 0.709854925017254 | 0.216473661954886 | 1.63563963167978 | 0 | |  |  |  |
| DIRC2 | 1793743 | 3.16490386686606 | 0.709419752436824 | 0.224152069787606 | 1.63514633395657 | 0 | |  |  |  |
| CTSH | 2390853 | 3.49290112012225 | 0.709014477209247 | 0.202987274138591 | 1.63468706071209 | 0 | |  |  |  |
| CTGF | 2115125 | 4.2994716246783 | 0.708905684064138 | 0.164882047364874 | 1.6345637941615 | 0 | |  |  |  |
| BEXL1 | 1804798 | 3.47495405106448 | 0.708614184873813 | 0.203920447424835 | 1.63423356089942 | 0 | |  |  |  |
| C10orf61 | 1768077 | 3.2493865362149 | 0.708012085406616 | 0.217891001121509 | 1.63355166636872 | 0 | |  |  |  |
| CDKN2A | 1717714 | 3.92196765663448 | 0.707924054312101 | 0.180502267303138 | 1.63345199252926 | 0 | |  |  |  |
| LOC402509 | 1727049 | 5.14694301655247 | 0.70778536381414 | 0.137515679023047 | 1.63329497155463 | 0 | |  |  |  |
| PUS3 | 2097185 | 4.53992280395304 | 0.707726399590455 | 0.155889522829379 | 1.63322821870716 | 0 | |  |  |  |
| EEF1E1 | 1725105 | 3.54389159284202 | 0.707431578472033 | 0.199619982705145 | 1.63289449539044 | 0 | |  |  |  |
| LOC100131713 | 3219340 | 4.72270324618962 | 0.70654047126058 | 0.149605095732118 | 1.6318862193987 | 0 | |  |  |  |
| ST3GAL5 | 2388701 | 4.35276011167445 | 0.705930897455169 | 0.162180060316626 | 1.63119685335803 | 0 | |  |  |  |
| IGFBP3 | 1746085 | 2.57725569667998 | 0.705589569343418 | 0.273775539715504 | 1.63081097313554 | 0 | |  |  |  |
| LEF1 | 2213136 | 4.12377597871737 | 0.705279799548571 | 0.171027670559334 | 1.63046084941885 | 0 | |  |  |  |
| RTN4IP1 | 1758827 | 3.32284613543399 | 0.705050586510023 | 0.212182736658054 | 1.63020182503167 | 0 | |  |  |  |
| RFC1 | 2217935 | 3.55667146488128 | 0.704950928667176 | 0.198205242071946 | 1.63008921856837 | 0 | |  |  |  |
| RNF150 | 1767446 | 2.27782088838173 | 0.704058160491675 | 0.309092854527237 | 1.62908079922294 | 0.0521258231948829 | |  |  |  |
| EBPL | 1805922 | 3.78602706802179 | 0.703675308278739 | 0.185861140355347 | 1.62864854263198 | 0 | |  |  |  |
| EPAS1 | 1704753 | 3.32220393452 | 0.703655376710171 | 0.211803787659964 | 1.62862604217627 | 0 | |  |  |  |
| PFKM | 2043809 | 4.19856152940163 | 0.703323183900683 | 0.167515273737317 | 1.62825108034233 | 0 | |  |  |  |
| GSTA4 | 1771964 | 5.20598869531704 | 0.702698661418843 | 0.134978906514135 | 1.62754638578647 | 0 | |  |  |  |
| NEFH | 1705153 | 3.39145622879202 | 0.701912194942378 | 0.206964839759229 | 1.62665939178716 | 0 | |  |  |  |
| TIMP4 | 1663399 | 3.06651022791809 | 0.701663050335261 | 0.228814841035646 | 1.62637850191682 | 0 | |  |  |  |
| CBR1 | 1809003 | 2.99922862506715 | 0.700115862325068 | 0.23343197529978 | 1.62463526135285 | 0 | |  |  |  |
| IDH1 | 2091347 | 2.97012984389531 | 0.699896615070805 | 0.235645123902359 | 1.62438838329076 | 0 | |  |  |  |
| C7orf50 | 1718336 | 2.92141944185372 | 0.699517915267988 | 0.239444533450535 | 1.62396204590653 | 0 | |  |  |  |
| FAM89A | 2285817 | 6.19843190066079 | 0.6990071688234 | 0.112771613857512 | 1.62338722862638 | 0 | |  |  |  |
| LYPD1 | 2388397 | 4.10625988004537 | 0.698220702346935 | 0.170038118079175 | 1.62250250131973 | 0 | |  |  |  |
| ANO10 | 1767111 | 4.74806956819558 | 0.698108587273732 | 0.147029982869235 | 1.62237641790395 | 0 | |  |  |  |
| A4GALT | 1735045 | 4.12501819929327 | 0.697454997921063 | 0.169079253526822 | 1.62164159330982 | 0 | |  |  |  |
| PARP1 | 1686871 | 2.34069296382887 | 0.696772341697564 | 0.297677804165222 | 1.6208744444499 | 0.0359206925696922 | |  |  |  |
| RAP2A | 1677404 | 2.83252612121746 | 0.695544889266503 | 0.245556390126969 | 1.61949598254163 | 0 | |  |  |  |
| HS1BP3 | 1738237 | 3.724341739891 | 0.695123834880476 | 0.186643407997468 | 1.61902339729638 | 0 | |  |  |  |
| ATP5G1 | 1676393 | 2.7367921053059 | 0.694334046475916 | 0.253703613485946 | 1.61813732232429 | 0 | |  |  |  |
| CAMK2G | 1809695 | 4.42561881466586 | 0.69280845099834 | 0.156544989528351 | 1.61642710766816 | 0 | |  |  |  |
| LOC100129076 | 3234081 | 5.21966568974568 | 0.692234587919947 | 0.13262048358382 | 1.61578426677237 | 0 | |  |  |  |
| C17orf61 | 2201533 | 3.21496734840079 | 0.691815194497968 | 0.215185760702079 | 1.61531462434179 | 0 | |  |  |  |
| EEF1A2 | 2108735 | 2.20381549341583 | 0.691405766860274 | 0.31373123971854 | 1.6148562734105 | 0.0521258231948829 | |  |  |  |
| LRCH2 | 1720606 | 2.34348387430673 | 0.690426628554305 | 0.294615480876118 | 1.61376066335184 | 0.0359206925696922 | |  |  |  |
| SLC1A1 | 1658917 | 2.22296676114322 | 0.690337766977767 | 0.310547948374516 | 1.61366126819459 | 0.0521258231948829 | |  |  |  |
| ABHD8 | 1712707 | 3.72133605163771 | 0.689572062551895 | 0.18530228202541 | 1.61280505138757 | 0 | |  |  |  |
| STXBP6 | 2172969 | 2.07933684831512 | 0.689306308304304 | 0.331502954349531 | 1.61250798907932 | 0.0848201265005491 | |  |  |  |
| NMNAT2 | 1803818 | 3.66024017181863 | 0.689137720453488 | 0.188276639811612 | 1.61231956854304 | 0 | |  |  |  |
| MRPL14 | 2072603 | 3.21247183552246 | 0.688813832464237 | 0.21441863702821 | 1.61195764012854 | 0 | |  |  |  |
| MAGEH1 | 1659659 | 4.47792946957487 | 0.688808019090072 | 0.153822882600129 | 1.61195114471977 | 0 | |  |  |  |
| LGMN | 1698019 | 3.47857893013356 | 0.688354575905119 | 0.197883845596308 | 1.61144458346743 | 0 | |  |  |  |
| MGST3 | 1751956 | 2.74707926811363 | 0.687554821716275 | 0.250285759751085 | 1.61055153096848 | 0 | |  |  |  |
| AGTPBP1 | 1718071 | 2.69058757082224 | 0.686809879340997 | 0.255263901011447 | 1.60972012977187 | 0 | |  |  |  |
| SRPX | 1709486 | 4.84596303394665 | 0.685672118968497 | 0.141493468721339 | 1.60845114795029 | 0 | |  |  |  |
| MICB | 1708006 | 2.36144512141693 | 0.684520240401595 | 0.289873448336104 | 1.60716743870213 | 0.0359206925696922 | |  |  |  |
| PPP1R14A | 1761968 | 3.36131169691796 | 0.684183895181989 | 0.203546697501849 | 1.60679279259735 | 0 | |  |  |  |
| EXT2 | 1761259 | 3.49760564662495 | 0.683904022739994 | 0.195534914978175 | 1.60648111660642 | 0 | |  |  |  |
| LOC728285 | 1660067 | 1.9996554159511 | 0.683385801957191 | 0.34175178208499 | 1.60590416695621 | 0.0848201265005491 | |  |  |  |
| ABLIM1 | 1785424 | 3.46105977544454 | 0.683328498697555 | 0.197433313213953 | 1.60584038236329 | 0 | |  |  |  |
| HSPA5 | 1773865 | 3.45048223951939 | 0.682576081984061 | 0.197820488442548 | 1.60500309791449 | 0 | |  |  |  |
| CCNB1IP1 | 2347349 | 2.98418117056274 | 0.682056200237212 | 0.228557236057016 | 1.60442483191467 | 0 | |  |  |  |
| FAM107B | 1758672 | 2.52133702091333 | 0.680798019971275 | 0.270014684401319 | 1.60302621639206 | 0.0359206925696922 | |  |  |  |
| NR1H3 | 1814022 | 3.24763426710299 | 0.680423472578575 | 0.209513577150896 | 1.60261009840355 | 0 | |  |  |  |
| LOC100127983 | 3273885 | 3.81567448450797 | 0.678559870917344 | 0.177834842482598 | 1.60054126292588 | 0 | |  |  |  |
| TOX2 | 3230215 | 2.74952407338194 | 0.678265880280947 | 0.246684830602946 | 1.60021513980843 | 0 | |  |  |  |
| NUDT7 | 3226181 | 3.64143557064332 | 0.678078191343586 | 0.186211777797236 | 1.6000069716684 | 0 | |  |  |  |
| BTN3A2 | 1676528 | 3.0992926825923 | 0.67803915868847 | 0.218772225836169 | 1.59996368346562 | 0 | |  |  |  |
| SC5DL | 1677607 | 2.30863628445062 | 0.677987668803 | 0.293674526979176 | 1.59990658167045 | 0.0359206925696922 | |  |  |  |
| RPL23AP7 | 1750273 | 2.96138471390406 | 0.677953619040027 | 0.22893128875048 | 1.59986882192533 | 0 | |  |  |  |
| LOC646278 | 3250427 | 3.55743332960979 | 0.677946975183838 | 0.190571941163603 | 1.59986145427409 | 0 | |  |  |  |
| ST6GALNAC4 | 1687857 | 2.9602814967852 | 0.67635244969829 | 0.228475721120709 | 1.59809419885041 | 0 | |  |  |  |
| MAP2 | 1764201 | 3.44658828419225 | 0.676158947386764 | 0.196182105790808 | 1.59787986791038 | 0 | |  |  |  |
| DACT1 | 1801068 | 3.30312523908 | 0.675866617714413 | 0.204614287620126 | 1.59755612632691 | 0 | |  |  |  |
| CDH2 | 1779228 | 1.86185387497984 | 0.67542646224184 | 0.362770930263877 | 1.59706879823864 | 0.200308840224877 | |  |  |  |
| TBC1D16 | 1758457 | 3.16971236381669 | 0.675082642684021 | 0.212979149272442 | 1.59668823405345 | 0 | |  |  |  |
| PIGH | 3251246 | 3.62353476216457 | 0.67467653697442 | 0.186192925211898 | 1.59623884389427 | 0 | |  |  |  |
| TNFSF9 | 1751464 | 3.01099421716802 | 0.674564421901218 | 0.22403378194983 | 1.59611480140702 | 0 | |  |  |  |
| NPC2 | 1716678 | 3.62733421233313 | 0.674037896298179 | 0.185821834118956 | 1.5955323900533 | 0 | |  |  |  |
| CYCSL1 | 1663751 | 2.01668254693918 | 0.673627638178459 | 0.334027603502028 | 1.5950787341958 | 0.0848201265005491 | |  |  |  |
| BLVRB | 1797793 | 1.54929192542859 | 0.673581131185131 | 0.434767083032977 | 1.59502731576136 | 0.378417023480785 | |  |  |  |
| VCAN | 1687301 | 1.8553273476454 | 0.67297820123591 | 0.362727473450972 | 1.59436086250205 | 0.200308840224877 | |  |  |  |
| IFI27L1 | 3272500 | 2.89571561377111 | 0.671889439302809 | 0.232028807009747 | 1.59315809648411 | 0 | |  |  |  |
| C1orf19 | 1734486 | 2.46696133448424 | 0.67138533671441 | 0.272150733507452 | 1.59260151674785 | 0.0359206925696922 | |  |  |  |
| DYNLT3 | 1681890 | 2.72567402841319 | 0.671146988373603 | 0.246231567449878 | 1.59233842401959 | 0 | |  |  |  |
| SAAL1 | 1658678 | 3.84997887841983 | 0.670844692916968 | 0.17424633072073 | 1.59200480795362 | 0 | |  |  |  |
| A2LD1 | 3241953 | 3.32826752022299 | 0.670562329028902 | 0.201474888948823 | 1.59169325168767 | 0 | |  |  |  |
| AKR1A1 | 2380771 | 4.14623829828893 | 0.670211035132868 | 0.16164315384609 | 1.59130572415272 | 0 | |  |  |  |
| PTGES | 1713829 | 4.50450099148775 | 0.669440347814855 | 0.148615873118889 | 1.59045587606063 | 0 | |  |  |  |
| GPC4 | 1789502 | 4.16792449001713 | 0.669077427170488 | 0.160530122072278 | 1.5900558354003 | 0 | |  |  |  |
| RGL1 | 1654398 | 3.065799186908 | 0.668705371223862 | 0.218117799130308 | 1.58964582951796 | 0 | |  |  |  |
| CD24 | 1805519 | 2.91297380100807 | 0.668109085130828 | 0.229356365958259 | 1.5889889423628 | 0 | |  |  |  |
| BOLA3 | 1786658 | 2.74041018255175 | 0.667609965434571 | 0.243616802216419 | 1.58843930541212 | 0 | |  |  |  |
| MRPL13 | 1671158 | 2.66514920446821 | 0.667227943703658 | 0.250352941810924 | 1.58801874667942 | 0 | |  |  |  |
| TGM2 | 1797236 | 3.89728569787855 | 0.666679825568003 | 0.171062600294072 | 1.58741553080711 | 0 | |  |  |  |
| SERPINE2 | 1655595 | 3.53405742420529 | 0.666664046409551 | 0.188639845477175 | 1.5873981688952 | 0 | |  |  |  |
| AKR1A1 | 1728047 | 3.78455417709648 | 0.666238839613407 | 0.176041564854687 | 1.586930382556 | 0 | |  |  |  |
| DGCR6L | 3245688 | 4.46699139423626 | 0.666066099352473 | 0.149108435761012 | 1.58674038373432 | 0 | |  |  |  |
| ADRA1B | 1679754 | 2.95875182391684 | 0.665742211363222 | 0.225007790779121 | 1.58638419725459 | 0 | |  |  |  |
| LXN | 1723962 | 2.04916267101344 | 0.66561930002371 | 0.324825017281093 | 1.58624904998129 | 0.0848201265005491 | |  |  |  |
| PTGR1 | 2225537 | 2.36588611367345 | 0.665501371576341 | 0.281290535385507 | 1.5861193924743 | 0.0359206925696922 | |  |  |  |
| RHPN2 | 1753143 | 3.44920687405711 | 0.66519741515566 | 0.192855180754417 | 1.58578525367432 | 0 | |  |  |  |
| FEZ1 | 1794106 | 4.75921771642717 | 0.66383874656485 | 0.139484845224355 | 1.5842925318318 | 0 | |  |  |  |
| PAK1 | 1767365 | 3.70103838580025 | 0.663678463534272 | 0.179322231858119 | 1.58411652713532 | 0 | |  |  |  |
| HSD17B8 | 1715324 | 4.0082575266004 | 0.663543094964406 | 0.165544027688059 | 1.58396789591213 | 0 | |  |  |  |
| MMP1 | 1726448 | 2.93199315799781 | 0.663413539768705 | 0.226267083181645 | 1.58382566068491 | 0 | |  |  |  |
| LGMN | 2332964 | 3.89072473151608 | 0.663322186746095 | 0.170488079347526 | 1.58372537429277 | 0 | |  |  |  |
| BTG3 | 1707339 | 2.81242458899331 | 0.663007434059105 | 0.235742297465982 | 1.58337989071518 | 0 | |  |  |  |
| APBB3 | 2320513 | 3.01226009486989 | 0.662296541446799 | 0.21986698378893 | 1.58259986738682 | 0 | |  |  |  |
| C16orf61 | 1783333 | 3.58759950110822 | 0.661963518155287 | 0.184514329972118 | 1.58223459144353 | 0 | |  |  |  |
| BPHL | 1657746 | 4.91649117889329 | 0.66181735331911 | 0.134611723938471 | 1.58207429745317 | 0 | |  |  |  |
| ST6GALNAC4 | 2413064 | 3.22921792361083 | 0.661590631726635 | 0.204876427474694 | 1.58182569174573 | 0 | |  |  |  |
| C14orf37 | 1796377 | 3.44929437399414 | 0.661248473132861 | 0.191705433470198 | 1.58145058053883 | 0 | |  |  |  |
| LIMCH1 | 2139761 | 5.40664462373089 | 0.658734604047056 | 0.12183796973741 | 1.57869733196286 | 0 | |  |  |  |
| PIR | 1761247 | 2.99452661116474 | 0.658722146816701 | 0.219975385879268 | 1.57868370045317 | 0 | |  |  |  |
| H1F0 | 1757467 | 2.10698047014573 | 0.658051117341534 | 0.312319514426262 | 1.5779495903737 | 0.0521258231948829 | |  |  |  |
| NOMO1 | 2126957 | 4.00580818847324 | 0.655332119195867 | 0.163595481451557 | 1.57497848324257 | 0 | |  |  |  |
| TMEM30A | 1735680 | 2.8256092928205 | 0.655226647978856 | 0.231888622975476 | 1.57486334537854 | 0 | |  |  |  |
| ODZ3 | 2336094 | 2.63637630714533 | 0.654967537587454 | 0.248434768516279 | 1.57458052274172 | 0 | |  |  |  |
| C1orf41 | 1681340 | 3.11774186273955 | 0.65426411931336 | 0.209851921075487 | 1.57381298787719 | 0 | |  |  |  |
| DHCR7 | 1815626 | 2.96516675360171 | 0.654164461470514 | 0.220616415814024 | 1.57370427648224 | 0 | |  |  |  |
| FAM65B | 1726597 | 3.27936396329561 | 0.652923721327074 | 0.199100718503632 | 1.57235144817139 | 0 | |  |  |  |
| ICAM2 | 1786823 | 4.19265631747752 | 0.651875653013137 | 0.155480345549843 | 1.57120960374372 | 0 | |  |  |  |
| PIR | 2383383 | 2.09541932434786 | 0.651363245604501 | 0.310851025394271 | 1.57065165042684 | 0.0521258231948829 | |  |  |  |
| RTN3 | 2363065 | 2.07939411618802 | 0.650223824267954 | 0.312698693915685 | 1.56941166039184 | 0.0848201265005491 | |  |  |  |
| SLC20A2 | 1813763 | 3.68257202858797 | 0.64890169688619 | 0.176208826833185 | 1.56797406506957 | 0 | |  |  |  |
| ANG | 1696974 | 4.61235950199661 | 0.648196617648049 | 0.140534712735956 | 1.56720794623042 | 0 | |  |  |  |
| SLFN11 | 1752520 | 5.3256958797149 | 0.647758123139525 | 0.121628823306787 | 1.56673167949823 | 0 | |  |  |  |
| SRI | 1699525 | 3.07613920258265 | 0.647709124700125 | 0.210559107389004 | 1.56667846928993 | 0 | |  |  |  |
|  | 1904578 | 3.33796466005812 | 0.647662617706798 | 0.194029201524117 | 1.5666279663571 | 0 | |  |  |  |
| NBEA | 1705346 | 4.16622975132418 | 0.646505756247752 | 0.155177653378878 | 1.56537222969076 | 0 | |  |  |  |
| NPW | 3246678 | 2.53519892791107 | 0.645700188684741 | 0.254694091882083 | 1.56449840600538 | 0.0359206925696922 | |  |  |  |
| MCAM | 1685275 | 3.57181729431927 | 0.645642054943081 | 0.180760101019145 | 1.56443536543812 | 0 | |  |  |  |
| SETD3 | 1724504 | 2.7601252756159 | 0.644858079912688 | 0.233633627288454 | 1.56358546643074 | 0 | |  |  |  |
| MAPKAPK3 | 1732452 | 3.09258398489692 | 0.64464215458652 | 0.208447743936696 | 1.5633514651843 | 0 | |  |  |  |
| CXCL12 | 1689111 | 2.02229934688174 | 0.644585681808907 | 0.318739005084889 | 1.56329027063577 | 0.0848201265005491 | |  |  |  |
| SLC25A39 | 1743911 | 3.07343292209094 | 0.64391465233374 | 0.209509909165568 | 1.56256331871722 | 0 | |  |  |  |
| RAB33A | 1724708 | 4.50935985730024 | 0.643731115806497 | 0.142754434371512 | 1.56236454545224 | 0 | |  |  |  |
| TAC3 | 2406184 | 2.29309291843893 | 0.643636440855793 | 0.280684849567266 | 1.56226202071291 | 0.0521258231948829 | |  |  |  |
| LOC339352 | 3289745 | 5.53265727269605 | 0.642965411380624 | 0.116212767155069 | 1.56153554694501 | 0 | |  |  |  |
| C21orf51 | 2356311 | 3.2495762665062 | 0.642888176552419 | 0.19783754059837 | 1.56145195221567 | 0 | |  |  |  |
| SLC25A42 | 2222880 | 4.37653187162035 | 0.642163996227735 | 0.146728966008874 | 1.56066835693474 | 0 | |  |  |  |
| MCEE | 1735347 | 3.05311730370185 | 0.641914851620618 | 0.210248997260049 | 1.56039886232841 | 0 | |  |  |  |
| RUVBL1 | 1693108 | 2.98815188797804 | 0.641398291801863 | 0.214647151767064 | 1.55984025843369 | 0 | |  |  |  |
| MOSC1 | 1799106 | 3.26948648097255 | 0.640963119221432 | 0.196043972945491 | 1.5593698212829 | 0 | |  |  |  |
| HSD11B1 | 2389501 | 2.69942434150489 | 0.640729753772765 | 0.237357922547134 | 1.55911760331763 | 0 | |  |  |  |
| S100A4 | 1684306 | 5.04694076785765 | 0.640414170603751 | 0.126891556699525 | 1.55877659052611 | 0 | |  |  |  |
| SCARB2 | 1814726 | 2.50840396081077 | 0.640261361911387 | 0.255246512090676 | 1.55861149534555 | 0.0359206925696922 | |  |  |  |
| CCNB1IP1 | 1752394 | 3.03452725573801 | 0.640134298161758 | 0.210950254920704 | 1.55847422842894 | 0 | |  |  |  |
| FABP5L2 | 3266606 | 3.35396766545311 | 0.64001886116046 | 0.190824398145769 | 1.55834953236424 | 0 | |  |  |  |
| STIM1 | 2064694 | 4.41697178270185 | 0.639652618588 | 0.144817003607102 | 1.55795397995142 | 0 | |  |  |  |
| STIM2 | 1738449 | 2.76018063680507 | 0.639124432020911 | 0.231551668575106 | 1.55738370021468 | 0 | |  |  |  |
| FLJ46906 | 1773553 | 3.15116259176065 | 0.63899238537914 | 0.202779884176689 | 1.55724116290027 | 0 | |  |  |  |
| PKNOX2 | 1807689 | 5.33010893306783 | 0.638798883067613 | 0.119847247230638 | 1.55703231103001 | 0 | |  |  |  |
| IL8 | 1666733 | 2.00493765449968 | 0.638489113272765 | 0.318458338013556 | 1.55669802707636 | 0.0848201265005491 | |  |  |  |
| RAB31 | 1660691 | 3.67922764896601 | 0.638392777358014 | 0.173512714696364 | 1.55659408208639 | 0 | |  |  |  |
|  | 1910948 | 2.45518814993834 | 0.638041483461979 | 0.259874781278169 | 1.55621510010169 | 0.0359206925696922 | |  |  |  |
| FEZ1 | 1727992 | 3.16116971157668 | 0.637674410407494 | 0.20172103005803 | 1.55581919386531 | 0 | |  |  |  |
| NOMO1 | 1678730 | 3.89594232755001 | 0.637079785278509 | 0.163523926104712 | 1.55517807530808 | 0 | |  |  |  |
| ST3GAL1 | 1683313 | 2.93309327859292 | 0.636956043456975 | 0.217161877566519 | 1.55504469139139 | 0 | |  |  |  |
| CLDND1 | 2352563 | 3.26820231421835 | 0.635844858509234 | 0.194554925728736 | 1.55384743422149 | 0 | |  |  |  |
| GEMIN6 | 1707484 | 2.18384702314119 | 0.635713642349487 | 0.291098064842973 | 1.55370611494365 | 0.0521258231948829 | |  |  |  |
| OLFM1 | 1742025 | 5.18789013351858 | 0.635379788575951 | 0.122473640000741 | 1.55334661371712 | 0 | |  |  |  |
| EDNRB | 1751904 | 3.05776746586252 | 0.635252724826321 | 0.207750501605632 | 1.55320981047865 | 0 | |  |  |  |
| EFEMP1 | 1735877 | 2.42689801305746 | 0.634340025082251 | 0.261378937915523 | 1.55222750594822 | 0.0359206925696922 | |  |  |  |
| TTC13 | 1745573 | 2.9356630850362 | 0.634322584959753 | 0.216074721991448 | 1.55220874184807 | 0 | |  |  |  |
| SOD3 | 1690034 | 3.21560424928358 | 0.634215452778692 | 0.197230568071302 | 1.55209348163774 | 0 | |  |  |  |
| BVES | 1689237 | 3.15937711428408 | 0.634169776267388 | 0.20072620435218 | 1.55204434229007 | 0 | |  |  |  |
| BBS7 | 1775012 | 3.39281278979718 | 0.633938071782769 | 0.186847347925927 | 1.55179509574267 | 0 | |  |  |  |
| WDR19 | 1655117 | 2.5401277586913 | 0.633771975378025 | 0.2495039760144 | 1.55161644901386 | 0.0359206925696922 | |  |  |  |
| ATP5J | 2348093 | 3.23930808719015 | 0.633415698589849 | 0.195540430715649 | 1.55123332114783 | 0 | |  |  |  |
| SEZ6L2 | 2413779 | 3.42080040241179 | 0.633367530632472 | 0.185151852234911 | 1.55118153023496 | 0 | |  |  |  |
| RNASET2 | 1671565 | 3.6560379458142 | 0.63315077482428 | 0.173179486703407 | 1.55094849252433 | 0 | |  |  |  |
| BPGM | 2352921 | 3.03016649919854 | 0.632683213444925 | 0.208794867744814 | 1.55044592885017 | 0 | |  |  |  |
| FAM190B | 2129388 | 2.08782996437159 | 0.632250532310566 | 0.302826639668841 | 1.54998100168496 | 0.0848201265005491 | |  |  |  |
| HIBADH | 1804150 | 4.18575390223481 | 0.632104367474391 | 0.151013266006132 | 1.54982397526101 | 0 | |  |  |  |
| TTC5 | 1706455 | 5.36946435098803 | 0.631502268007193 | 0.11760991911437 | 1.54917730117625 | 0 | |  |  |  |
| CD46 | 2307740 | 2.17739712532994 | 0.63072078442287 | 0.289667317498317 | 1.54833836518575 | 0.0521258231948829 | |  |  |  |
| C8orf48 | 1784408 | 3.54003960939643 | 0.630614482723835 | 0.178137691185708 | 1.54822428360188 | 0 | |  |  |  |
| DHCR7 | 2165867 | 2.89585918899018 | 0.630193428337807 | 0.217618809206522 | 1.5477724961565 | 0 | |  |  |  |
| RP11-529I10.4 | 2047112 | 2.65223083473127 | 0.630098753387102 | 0.237573119630421 | 1.54767092896964 | 0 | |  |  |  |
| GOLPH4 | 1795344 | 1.87900841817621 | 0.629624548151558 | 0.335083409984231 | 1.54716230238307 | 0.200308840224877 | |  |  |  |
| C5orf32 | 1761566 | 3.86763182377421 | 0.629221764370053 | 0.162689157872331 | 1.54673041284162 | 0 | |  |  |  |
| IFT57 | 1811636 | 2.69077284154217 | 0.628344775353003 | 0.233518328136863 | 1.54579046826313 | 0 | |  |  |  |
| INSIG2 | 1676629 | 4.45906043028511 | 0.628026700737917 | 0.140842832376183 | 1.54544970150533 | 0 | |  |  |  |
| GAMT | 1756469 | 3.59227932745209 | 0.625907310613379 | 0.174236815558917 | 1.54318102652532 | 0 | |  |  |  |
| NTHL1 | 2227968 | 3.06099013681806 | 0.62586744747624 | 0.2044656857754 | 1.54313838744688 | 0 | |  |  |  |
| B4GALT4 | 2380688 | 2.21809734673001 | 0.625448054054261 | 0.281975024665313 | 1.54268986020355 | 0.0521258231948829 | |  |  |  |
| SNORA12 | 3238435 | 2.91468358760853 | 0.624405799114489 | 0.214227644389629 | 1.54157576787789 | 0 | |  |  |  |
| KIAA0672 | 2065022 | 2.37601387554183 | 0.624315276573904 | 0.262757420316635 | 1.54147904405673 | 0.0359206925696922 | |  |  |  |
| PPM1G | 1806867 | 2.04864984549172 | 0.623457388643399 | 0.304325988169908 | 1.54056268742907 | 0.0848201265005491 | |  |  |  |
| CISD1 | 2122952 | 2.41886308651844 | 0.623451575269235 | 0.257745706544554 | 1.54055647970739 | 0.0359206925696922 | |  |  |  |
| TMOD2 | 3250273 | 2.21045546421655 | 0.623048791487728 | 0.281864439964438 | 1.54012643417786 | 0.0521258231948829 | |  |  |  |
| CCDC28A | 1798659 | 3.29382994011455 | 0.621876981352258 | 0.188800573392878 | 1.5388759945657 | 0 | |  |  |  |
| CD14 | 2396444 | 2.80720041705504 | 0.621730816516082 | 0.221477174461353 | 1.53872009317461 | 0 | |  |  |  |
| RUVBL1 | 1656540 | 4.28483335685465 | 0.62156305914729 | 0.145061197806665 | 1.53854118036243 | 0 | |  |  |  |
| PET112L | 1753010 | 2.82350231401881 | 0.620931892809261 | 0.219915489258255 | 1.53786822940071 | 0 | |  |  |  |
| CXCL6 | 1779234 | 4.73944857100962 | 0.620876250513672 | 0.131001790864757 | 1.53780891758079 | 0 | |  |  |  |
| CITED4 | 1787691 | 3.91226938809875 | 0.619923687632463 | 0.158456288699927 | 1.53679388934581 | 0 | |  |  |  |
| LRP11 | 1676197 | 3.099471498513 | 0.618802536900438 | 0.199647758399235 | 1.53560007621394 | 0 | |  |  |  |
| MDH1 | 1656913 | 3.96695692673175 | 0.618594916394507 | 0.155936887599167 | 1.53537910149946 | 0 | |  |  |  |
| CETN3 | 2224031 | 2.03200240020522 | 0.617668928938058 | 0.303970570544443 | 1.53439394138118 | 0.0848201265005491 | |  |  |  |
| LOC653381 | 1692517 | 3.17025106085064 | 0.616616708214003 | 0.194500907460797 | 1.53327524863541 | 0 | |  |  |  |
| ASCC2 | 1679919 | 2.56231209854817 | 0.616458916629496 | 0.240586974935172 | 1.53310755921118 | 0 | |  |  |  |
| PTK7 | 2353202 | 3.56685476039381 | 0.6160984874312 | 0.172728784550559 | 1.53272459005048 | 0 | |  |  |  |
| RSL24D1 | 2175465 | 2.49185002125982 | 0.61587757921289 | 0.247156760622984 | 1.53248991430322 | 0.0359206925696922 | |  |  |  |
| ASF1A | 1714737 | 3.03698622819044 | 0.615530437726974 | 0.202678047076207 | 1.53212121072263 | 0 | |  |  |  |
| IPO5 | 2166384 | 2.73000172418946 | 0.615094434664522 | 0.225309174428138 | 1.53165825175338 | 0 | |  |  |  |
| EFEMP1 | 2350634 | 3.10195137987043 | 0.614927507777753 | 0.198238925267565 | 1.53148104163935 | 0 | |  |  |  |
| C9orf119 | 1811555 | 2.31589561357015 | 0.614421744225306 | 0.265306320641163 | 1.53094424660189 | 0.0359206925696922 | |  |  |  |
| LOC729217 | 3229467 | 2.99095616580402 | 0.613894388140243 | 0.205250212343121 | 1.53038473457652 | 0 | |  |  |  |
| RIOK1 | 1797534 | 3.37995649671148 | 0.613766493908591 | 0.181590057299777 | 1.53024907270835 | 0 | |  |  |  |
| SERPING1 | 1670305 | 4.92240613471453 | 0.613337965184351 | 0.124601251582814 | 1.52979460500502 | 0 | |  |  |  |
| SMAD6 | 1767068 | 2.53675696912208 | 0.612593853291095 | 0.241487009101665 | 1.52900577242954 | 0.0359206925696922 | |  |  |  |
| BST2 | 1723480 | 1.57255403892393 | 0.612587209434905 | 0.389549226463523 | 1.52899873111395 | 0.378417023480785 | |  |  |  |
| PAPSS1 | 1781819 | 2.83988224093893 | 0.611471872077046 | 0.21531592516839 | 1.52781712978473 | 0 | |  |  |  |
| TPM1 | 2278152 | 3.12946620276453 | 0.611342316881345 | 0.195350349635121 | 1.52767993671588 | 0 | |  |  |  |
| SYNGR1 | 1727805 | 4.9723291996523 | 0.611255946750878 | 0.122931512015259 | 1.52758848151421 | 0 | |  |  |  |
| LAPTM4B | 2101832 | 2.52583570221806 | 0.610868942127824 | 0.241848249112716 | 1.52717875908367 | 0.0359206925696922 | |  |  |  |
| ARMC10 | 2052598 | 2.77377327711608 | 0.610464497382272 | 0.220084497323076 | 1.5267506902001 | 0 | |  |  |  |
| LOC387856 | 1779428 | 4.19185699510064 | 0.610399719784421 | 0.145615587673397 | 1.52668214000817 | 0 | |  |  |  |
|  | 1864685 | 4.20917098410833 | 0.61029009615729 | 0.144990569036381 | 1.52656613900268 | 0 | |  |  |  |
| PDIA5 | 1695763 | 2.56892278721371 | 0.610097424327785 | 0.237491538229339 | 1.52636227980644 | 0 | |  |  |  |
| KIAA0363 | 1652246 | 3.28159963134655 | 0.610078323241241 | 0.185908822457694 | 1.52634207111078 | 0 | |  |  |  |
| SGCD | 1702861 | 3.59555821053101 | 0.61002018949958 | 0.169659383545202 | 1.52628056803119 | 0 | |  |  |  |
| VPS41 | 1703379 | 3.36110767292113 | 0.608837583097799 | 0.181141945556496 | 1.52502995752203 | 0 | |  |  |  |
| AIG1 | 1797974 | 3.46507897422913 | 0.608447256546651 | 0.175594051700369 | 1.52461741076112 | 0 | |  |  |  |
|  | 1860638 | 2.68897573800293 | 0.608134995305732 | 0.226158602590214 | 1.52428745370653 | 0 | |  |  |  |
| GCHFR | 1694780 | 3.49712334362495 | 0.607831869367074 | 0.173809102408445 | 1.52396721795711 | 0 | |  |  |  |
| DBNDD2 | 1730612 | 3.02668879568176 | 0.607787853819817 | 0.200809496730209 | 1.52392072356574 | 0 | |  |  |  |
| BEND6 | 1706969 | 2.97673880472702 | 0.607514625234012 | 0.204087313360947 | 1.52363213916891 | 0 | |  |  |  |
| NOTCH2NL | 1656254 | 3.08201797168134 | 0.607281259785346 | 0.197040142324042 | 1.52338570154218 | 0 | |  |  |  |
| BZW2 | 1676548 | 3.46704515298629 | 0.606999726379305 | 0.175076960234127 | 1.52308845083517 | 0 | |  |  |  |
| TRAK1 | 2289924 | 3.29020536070625 | 0.606707396706954 | 0.184398033008104 | 1.52277986251339 | 0 | |  |  |  |
| PCOLCE | 1707070 | 3.02790130784499 | 0.6064009488402 | 0.200271041618522 | 1.52245643790248 | 0 | |  |  |  |
| ANKRD57 | 1724040 | 2.00682283150776 | 0.606084535189162 | 0.302011979170976 | 1.52212256749751 | 0.0848201265005491 | |  |  |  |
| AKR1C4 | 1687757 | 1.90668740938191 | 0.605153564840569 | 0.317384780464219 | 1.52114065945435 | 0.200308840224877 | |  |  |  |
| CD82 | 1662973 | 3.15858683072065 | 0.604660258518479 | 0.191433793314627 | 1.52062061883187 | 0 | |  |  |  |
| PPP1CC | 1701855 | 3.67694151299613 | 0.604603785740866 | 0.164431167480879 | 1.52056109690456 | 0 | |  |  |  |
| CACNA1H | 1671263 | 2.07447566916741 | 0.604153664484009 | 0.291231983803641 | 1.5200867554176 | 0.0848201265005491 | |  |  |  |
| MAD2L2 | 1669550 | 3.41940067824078 | 0.602839011440457 | 0.176299611588837 | 1.51870221025291 | 0 | |  |  |  |
| LEF1 | 1679185 | 2.34960705636003 | 0.602350688010509 | 0.2563623080634 | 1.51818824690368 | 0.0359206925696922 | |  |  |  |
| NEK1 | 2162234 | 4.421266002335 | 0.601557577677854 | 0.136060028362952 | 1.51735386413927 | 0 | |  |  |  |
| GPSM2 | 2139816 | 3.42321430772365 | 0.601454597906914 | 0.175698785948013 | 1.51724555907669 | 0 | |  |  |  |
| TRMT12 | 1809944 | 3.7774004815199 | 0.60133085608538 | 0.159191713726744 | 1.5171154285415 | 0 | |  |  |  |
| SNCAIP | 1807969 | 2.0754216830185 | 0.601243655472888 | 0.289697106083251 | 1.51702373251925 | 0.0848201265005491 | |  |  |  |
| CCNE1 | 2374425 | 3.76076805538433 | 0.601242824990865 | 0.159872349513834 | 1.51702285925044 | 0 | |  |  |  |
| MAPKAP1 | 2268068 | 2.74916725671918 | 0.60029358403775 | 0.218354697252626 | 1.51602504159156 | 0 | |  |  |  |
| C20orf108 | 1708016 | 1.83681216140133 | 0.599555285518661 | 0.326410777387956 | 1.51524941495878 | 0.200308840224877 | |  |  |  |
| PREP | 1751887 | 2.48667711837991 | 0.599346004048683 | 0.241022849174388 | 1.51502962448583 | 0.0359206925696922 | |  |  |  |
| PITPNM1 | 1653220 | 3.53426163739685 | 0.598650060112803 | 0.169384760250443 | 1.51429896320269 | 0 | |  |  |  |
| PAQR5 | 1806434 | 2.7563334613719 | 0.598304579590937 | 0.217065383407254 | 1.51393637918744 | 0 | |  |  |  |
| FLYWCH1 | 1690035 | 4.13540290503321 | 0.597596178424702 | 0.144507365339751 | 1.51319317913282 | 0 | |  |  |  |
| PPAP2A | 2343278 | 3.84517420099756 | 0.596838778819067 | 0.155217617621649 | 1.51239897724383 | 0 | |  |  |  |
| LOC100131727 | 3292990 | 2.78980063993969 | 0.596736629530149 | 0.213899380832836 | 1.51229189639409 | 0 | |  |  |  |
| APITD1 | 1793360 | 2.93492065853382 | 0.595849674728815 | 0.203020709604627 | 1.51136243986632 | 0 | |  |  |  |
| LOC730743 | 3247132 | 3.63404079214091 | 0.595848013764767 | 0.163962940386736 | 1.51136069984701 | 0 | |  |  |  |
| GALNTL4 | 1673773 | 3.40021873592369 | 0.595480940710281 | 0.175130186308004 | 1.51097620471748 | 0 | |  |  |  |
| NDUFB3 | 2119937 | 2.69794473145745 | 0.59455246180776 | 0.220372365258416 | 1.51000409477854 | 0 | |  |  |  |
| FAM164A | 1789558 | 3.96952832325138 | 0.594035901989006 | 0.149648989404978 | 1.50946353160027 | 0 | |  |  |  |
| MKKS | 2274180 | 2.53703320656525 | 0.593547578559057 | 0.23395341338974 | 1.5089526948299 | 0.0359206925696922 | |  |  |  |
| FBL | 1719205 | 1.85158869014379 | 0.592979528854831 | 0.320254456084835 | 1.50835867365436 | 0.200308840224877 | |  |  |  |
| ERLIN2 | 1700549 | 3.05594351509848 | 0.592816754378183 | 0.193988125581922 | 1.50818850016895 | 0 | |  |  |  |
| TFPI2 | 2068104 | 1.55147592720352 | 0.590816953665061 | 0.380809617027051 | 1.50609936355885 | 0.378417023480785 | |  |  |  |
| LOC728969 | 3233179 | 2.84378411688747 | 0.590363510480107 | 0.207597864751514 | 1.50562606659681 | 0 | |  |  |  |
| GLIPR1 | 1769245 | 2.35060115746729 | 0.590006403209908 | 0.251002345223731 | 1.50525342826323 | 0.0359206925696922 | |  |  |  |
| ALDH4A1 | 1696099 | 4.840473653691 | 0.589974014410983 | 0.121883529716376 | 1.50521963539503 | 0 | |  |  |  |
| UBE2CBP | 1719103 | 3.51428973842463 | 0.589918372115393 | 0.167862759198631 | 1.50516158275161 | 0 | |  |  |  |
| PBX3 | 1810100 | 2.50885318224724 | 0.589307967827956 | 0.23489137267893 | 1.50452488357284 | 0.0359206925696922 | |  |  |  |
| NCKIPSD | 1736623 | 3.64286108230299 | 0.589304645899863 | 0.161769727855587 | 1.50452141928026 | 0 | |  |  |  |
| POLR2F | 1745885 | 2.48173859917099 | 0.587897809351678 | 0.236889497366105 | 1.50305500815928 | 0.0359206925696922 | |  |  |  |
| RPL39L | 1712413 | 2.13808396688821 | 0.587337233985666 | 0.274702604332459 | 1.50247109270068 | 0.0521258231948829 | |  |  |  |
| GSTM4 | 1651800 | 4.03104634188474 | 0.587322285309239 | 0.145699710570589 | 1.5024555247274 | 0 | |  |  |  |
| CNFN | 1803838 | 2.83824418353102 | 0.587280761208053 | 0.20691692582892 | 1.50241228120355 | 0 | |  |  |  |
| KBTBD3 | 1743662 | 3.34392055490726 | 0.587041582385221 | 0.175554883181578 | 1.50216322273654 | 0 | |  |  |  |
| ZDHHC14 | 2309926 | 3.71378852936679 | 0.585440413043485 | 0.157639673991697 | 1.50049697767892 | 0 | |  |  |  |
| ANXA10 | 1699421 | 3.43578667846752 | 0.585297570135404 | 0.170353291664914 | 1.50034841908869 | 0 | |  |  |  |
| ADAMTSL4 | 1762294 | 3.81389854546086 | 0.585122338428399 | 0.15341843299025 | 1.50016619579052 | 0 | |  |  |  |
| COL11A1 | 2392803 | 3.75563179736701 | 0.585027663477695 | 0.155773434415974 | 1.50006775260155 | 0 | |  |  |  |
|  |  |  |  |  | | |  |  | |  |
| **Genes down-regulated by VPA** | | | |  | | |  |  | |  |
|  | Gene Name | Score(d) | Numerator(r) | Denominator(s+s0) | Fold Change | q-value(%) | |  |  |  |
| CENTA1 | 2047511 | -12.0172217311455 | -1.39931196580887 | 0.11644221910146 | 0.37910989942942 | 0 | |  |  |  |
| CDC25B | 2338323 | -11.9719935901296 | -1.17349559873866 | 0.0980200657396075 | 0.443345826983736 | 0 | |  |  |  |
| LOC730534 | 1689327 | -11.4051584240587 | -1.80838581816163 | 0.158558588221529 | 0.285510197860341 | 0 | |  |  |  |
| TCF25 | 1814971 | -11.3646337550657 | -1.47970428666919 | 0.130202549291095 | 0.358562300012837 | 0 | |  |  |  |
| ATF5 | 1669113 | -11.3158165844134 | -1.83548278563163 | 0.162205066858353 | 0.28019773802524 | 0 | |  |  |  |
| TMEM173 | 2145116 | -10.6327542989637 | -1.28846420817462 | 0.12117878133422 | 0.409386601624271 | 0 | |  |  |  |
| PRKCDBP | 1793476 | -10.2917862101662 | -1.52573790524409 | 0.148248114961519 | 0.347301872625369 | 0 | |  |  |  |
| USP5 | 1671494 | -9.75140240379918 | -1.16220768707224 | 0.119183645484616 | 0.446828252176874 | 0 | |  |  |  |
| HIGD2A | 1774334 | -9.75067458231573 | -1.13601179259798 | 0.116505969203228 | 0.455015692724229 | 0 | |  |  |  |
| BAIAP2L2 | 1771652 | -9.50413454408372 | -1.22602773914919 | 0.128999408990099 | 0.427492869327741 | 0 | |  |  |  |
| FBXO6 | 1701455 | -9.36125108522882 | -1.19315061679409 | 0.127456320307097 | 0.43734672124399 | 0 | |  |  |  |
| TRIM22 | 1779252 | -9.20119143230174 | -1.46246347985672 | 0.158942838067969 | 0.362872974764994 | 0 | |  |  |  |
| TRMT5 | 2167922 | -9.03995202051848 | -1.44260748515156 | 0.159581320993429 | 0.367901768088947 | 0 | |  |  |  |
| PTGER1 | 1744929 | -9.00933694248906 | -1.7031122558706 | 0.189038579280849 | 0.307122847002021 | 0 | |  |  |  |
| SERTAD1 | 1794017 | -8.85035521528144 | -1.43610148897772 | 0.162264841810878 | 0.369564609216816 | 0 | |  |  |  |
| RBM23 | 2363106 | -8.77867767831978 | -0.883230504699546 | 0.100610882078609 | 0.542152075867546 | 0 | |  |  |  |
| PARP4 | 1776464 | -8.75245472006543 | -0.920719293732375 | 0.105195550640391 | 0.528245583821113 | 0 | |  |  |  |
| MYPN | 1758272 | -8.74213106736377 | -1.07831820641002 | 0.123347293480375 | 0.473580569013441 | 0 | |  |  |  |
| HMGN2 | 2058141 | -8.61392626541225 | -1.7456727986223 | 0.202657039871789 | 0.298194840619921 | 0 | |  |  |  |
| TAX1BP3 | 1803392 | -8.16961079840019 | -1.55330907794963 | 0.190132567668194 | 0.340727647698946 | 0 | |  |  |  |
| IRF9 | 1745471 | -8.16538165880738 | -1.93927476799233 | 0.237499586550322 | 0.260747483080095 | 0 | |  |  |  |
| C10orf32 | 1772706 | -8.14035534498789 | -1.17740966051646 | 0.144638607360231 | 0.44214465064051 | 0 | |  |  |  |
| SGSH | 2133675 | -8.12898772870667 | -0.970751683251496 | 0.119418519949709 | 0.510240144627954 | 0 | |  |  |  |
| DIO2 | 1737650 | -8.07136556836387 | -2.32931480284341 | 0.288589927331911 | 0.19897860189054 | 0 | |  |  |  |
| MRGPRF | 2075603 | -8.06517051517874 | -1.18763538567455 | 0.147254838994341 | 0.43902183938553 | 0 | |  |  |  |
| UBA7 | 1794612 | -8.04070650999694 | -1.16515672873847 | 0.144907257501544 | 0.445915814531126 | 0 | |  |  |  |
| EMP3 | 1765446 | -7.96983796931308 | -1.72041784028092 | 0.215866100026774 | 0.303460818587274 | 0 | |  |  |  |
| GTF2IRD2B | 1680501 | -7.833770943182 | -1.07495143228585 | 0.137220176602358 | 0.474687040343338 | 0 | |  |  |  |
| PSMB10 | 1683026 | -7.70541950053998 | -1.28633402178377 | 0.166938869673952 | 0.409991520806351 | 0 | |  |  |  |
| PHF11 | 2390162 | -7.56746134597259 | -1.20648151423887 | 0.159430152210947 | 0.433324131254617 | 0 | |  |  |  |
| MZF1 | 1749838 | -7.55542819327048 | -0.981695775360103 | 0.129932513452313 | 0.506384174889607 | 0 | |  |  |  |
| RBM23 | 1780756 | -7.54240838883896 | -0.899090219906563 | 0.119204658983596 | 0.536224774182843 | 0 | |  |  |  |
| HIST2H2AA4 | 3242900 | -7.5289833905513 | -1.09065916928253 | 0.144861412584769 | 0.469546789036258 | 0 | |  |  |  |
| C7orf55 | 3238680 | -7.52896424789056 | -1.05708278106345 | 0.140402151777998 | 0.480602886668731 | 0 | |  |  |  |
| MPDU1 | 1655654 | -7.40925785756235 | -0.922675078898239 | 0.124530026709288 | 0.527529954474118 | 0 | |  |  |  |
| NRBP1 | 1670096 | -7.37736902662983 | -0.761365572538604 | 0.10320285860587 | 0.589937665133223 | 0 | |  |  |  |
| RPL6 | 1717490 | -7.3491251792616 | -0.787358829399074 | 0.107136401978961 | 0.579403847784966 | 0 | |  |  |  |
| NPTX1 | 1814221 | -7.32572748246507 | -2.20640097188651 | 0.30118523753003 | 0.216674161710719 | 0 | |  |  |  |
| SLC14A1 | 1805561 | -7.2308313586088 | -1.23738291985954 | 0.171125954747423 | 0.424141360387778 | 0 | |  |  |  |
| NUDT14 | 1669788 | -7.14544394912261 | -1.1311044743198 | 0.158297298582643 | 0.456566061084328 | 0 | |  |  |  |
| C1QTNF5 | 1744487 | -7.09749868693189 | -1.40139896713448 | 0.197449697273601 | 0.378561875876668 | 0 | |  |  |  |
| MX1 | 1662358 | -7.07704879672732 | -2.8121188492622 | 0.397357561044743 | 0.142386191890698 | 0 | |  |  |  |
| BTBD6 | 1744725 | -6.99738347065731 | -1.17284948372421 | 0.167612578136158 | 0.443544425122026 | 0 | |  |  |  |
| KIAA1644 | 1728885 | -6.99011389831683 | -1.08755399699583 | 0.155584588865956 | 0.47055850242264 | 0 | |  |  |  |
| RNH1 | 1660880 | -6.98608154518138 | -0.948318702826722 | 0.135744007093765 | 0.518236055391094 | 0 | |  |  |  |
| LOC728098 | 3282506 | -6.92622654036215 | -0.948886752530948 | 0.136999092796253 | 0.518032044230315 | 0 | |  |  |  |
| LOC440498 | 1690909 | -6.92124395535789 | -0.900918110840774 | 0.130167079307088 | 0.535545808977129 | 0 | |  |  |  |
| ESPNL | 2115862 | -6.91497749516821 | -0.829719225983053 | 0.119988709516815 | 0.562638731155868 | 0 | |  |  |  |
| SARS2 | 1655684 | -6.88927252181023 | -0.939998103431054 | 0.136443739227209 | 0.521233565634495 | 0 | |  |  |  |
| ARHGAP23 | 1764571 | -6.88870958031287 | -1.13229787698789 | 0.164370099187207 | 0.456188544114987 | 0 | |  |  |  |
| UBE2C | 1714730 | -6.87989748811485 | -0.806399290756945 | 0.117210945679062 | 0.57180720718428 | 0 | |  |  |  |
| ZNF469 | 1794442 | -6.8689185377925 | -1.11251413421879 | 0.161963506787537 | 0.462487367707817 | 0 | |  |  |  |
| CDC20 | 1663390 | -6.86877710392166 | -1.93038362744637 | 0.281037453718543 | 0.262359397567842 | 0 | |  |  |  |
| C10orf32 | 2151056 | -6.86389624679547 | -1.17115363943177 | 0.17062519556273 | 0.44406610469911 | 0 | |  |  |  |
| LOC440093 | 1769705 | -6.86351561526039 | -0.986439488679602 | 0.143722189031864 | 0.50472187178198 | 0 | |  |  |  |
| MED16 | 1790782 | -6.86237250277174 | -1.04473102192464 | 0.152240500133542 | 0.484735274763886 | 0 | |  |  |  |
| AURKA | 1680955 | -6.79599949944572 | -0.972060522920882 | 0.143034225208546 | 0.509777455227644 | 0 | |  |  |  |
| PARP3 | 2397954 | -6.77503226854274 | -1.10225435929773 | 0.162693595485238 | 0.465788085046508 | 0 | |  |  |  |
| SPPL2A | 1734229 | -6.71951560719021 | -0.707288735563957 | 0.105258887233943 | 0.612470076225979 | 0 | |  |  |  |
| MAP3K6 | 1694539 | -6.71655247990479 | -1.24262076998315 | 0.185008718937422 | 0.422604264301101 | 0 | |  |  |  |
| PSEN2 | 1714417 | -6.66640603544581 | -0.750133303167765 | 0.112524394580715 | 0.594548619434917 | 0 | |  |  |  |
| IFI35 | 1745374 | -6.66311364006112 | -1.39495608759445 | 0.209354989716437 | 0.380256262282997 | 0 | |  |  |  |
| LOC148915 | 1776052 | -6.62012606004269 | -1.34460479297822 | 0.203108638836032 | 0.393761839304173 | 0 | |  |  |  |
| PHF15 | 1795285 | -6.61227508917167 | -0.781014777219862 | 0.118115893045475 | 0.581957306305061 | 0 | |  |  |  |
| LOC100131735 | 3208233 | -6.58581305836878 | -0.698229007167174 | 0.106020168045905 | 0.616328321676205 | 0 | |  |  |  |
| HIST1H2BC | 1680937 | -6.56096252612086 | -1.11185057908184 | 0.169464552595031 | 0.462700133708597 | 0 | |  |  |  |
| RDH10 | 1714335 | -6.55623894937865 | -1.10762259509907 | 0.168941767322871 | 0.464058118636592 | 0 | |  |  |  |
| DHRS4 | 2185884 | -6.48269652199198 | -1.22093688434377 | 0.188337812853317 | 0.429004033060652 | 0 | |  |  |  |
| FER1L3 | 1810289 | -6.43535937296345 | -1.14490210266192 | 0.177908029110533 | 0.452220374176679 | 0 | |  |  |  |
| AIP | 2103841 | -6.422824048813 | -0.736017600210566 | 0.114594078028121 | 0.600394389164763 | 0 | |  |  |  |
| PAF1 | 1669508 | -6.38777473457325 | -0.640778752212169 | 0.100313298267081 | 0.641366652084998 | 0 | |  |  |  |
| MRPL54 | 1658486 | -6.38082661189563 | -1.16822203788803 | 0.183083181685291 | 0.444969378372314 | 0 | |  |  |  |
| KEAP1 | 2410772 | -6.36733964495713 | -0.80518678700231 | 0.126455762044987 | 0.572287980871791 | 0 | |  |  |  |
| TOR1A | 1805812 | -6.34663899979863 | -0.946055639312079 | 0.149064038358271 | 0.519049617097412 | 0 | |  |  |  |
| ALS2CR4 | 1800958 | -6.34161027487197 | -0.731568708009488 | 0.115360086208429 | 0.602248705250916 | 0 | |  |  |  |
| CKS1B | 2041046 | -6.33306637331107 | -1.10735102747731 | 0.174852269375216 | 0.464145479456361 | 0 | |  |  |  |
| PARP10 | 2262044 | -6.31681925705283 | -1.21222263646886 | 0.191903961018893 | 0.4316031692154 | 0 | |  |  |  |
| H2AFY2 | 1705570 | -6.31437613134889 | -1.09890917770618 | 0.174032898080056 | 0.466869362460785 | 0 | |  |  |  |
| U2AF2 | 1768930 | -6.31249322168751 | -0.763783105709658 | 0.120995473402738 | 0.588949930677866 | 0 | |  |  |  |
| TXNRD2 | 1657893 | -6.27371807162592 | -0.697291392962392 | 0.111144840268807 | 0.616729006503126 | 0 | |  |  |  |
| PPP1R15A | 1659936 | -6.26042614744365 | -0.97399471555413 | 0.155579619120952 | 0.509094464640376 | 0 | |  |  |  |
| MYD88 | 1738523 | -6.20611025372904 | -0.696406099125105 | 0.112212975705138 | 0.617107571568352 | 0 | |  |  |  |
| SPATA20 | 1687247 | -6.18383246317104 | -0.679818051183285 | 0.109934746006149 | 0.624243997459849 | 0 | |  |  |  |
| HNRPM | 1745385 | -6.17549676127735 | -1.01513845597336 | 0.164381667615576 | 0.494780841629753 | 0 | |  |  |  |
| NUDT16L1 | 1735415 | -6.14885925080105 | -0.738467522180546 | 0.120098296620522 | 0.599375690726082 | 0 | |  |  |  |
| SERPINH1 | 1751028 | -6.13781537238664 | -1.2468097213108 | 0.203135748742144 | 0.421378987240445 | 0 | |  |  |  |
| UBE2Z | 1692168 | -6.12244191148717 | -1.08244902399601 | 0.176800211360941 | 0.472226521974916 | 0 | |  |  |  |
| TNFRSF14 | 1697409 | -6.07643817560674 | -1.27352217560382 | 0.209583663784525 | 0.413648663062855 | 0 | |  |  |  |
| HIST2H2BE | 1732071 | -6.06412317791105 | -0.985217849622707 | 0.16246666182696 | 0.50514943898329 | 0 | |  |  |  |
| POLR2A | 1782385 | -6.0480824092124 | -1.40493765103756 | 0.23229472682078 | 0.377634466269627 | 0 | |  |  |  |
| ZNFX1 | 1745148 | -6.02426276069728 | -0.790116029717831 | 0.131155638640566 | 0.578297580110929 | 0 | |  |  |  |
| RPL13 | 1709039 | -6.01981842608019 | -0.657152535791869 | 0.109164843402058 | 0.63412864985222 | 0 | |  |  |  |
| TXNDC15 | 1662848 | -5.99293742975355 | -0.831340326893359 | 0.138720007782151 | 0.562006870726089 | 0 | |  |  |  |
| SMARCD1 | 1728845 | -5.98934802134149 | -0.899603457797222 | 0.150200565168649 | 0.536034046470488 | 0 | |  |  |  |
| HOXB7 | 1702125 | -5.98291217251728 | -0.83351868124158 | 0.139316549734823 | 0.561158925539515 | 0 | |  |  |  |
| KDELR3 | 1798952 | -5.96943778725073 | -0.760612325343089 | 0.127417748949084 | 0.590245758600218 | 0 | |  |  |  |
| KIF22 | 3234884 | -5.93390594684506 | -1.01034491373243 | 0.170266418575376 | 0.496427549626643 | 0 | |  |  |  |
| EIF4A1 | 3251629 | -5.93274287466111 | -0.776899738792321 | 0.130951189897421 | 0.583619608596007 | 0 | |  |  |  |
| CYB561D2 | 1738718 | -5.92978691124783 | -0.86565252218545 | 0.145983748681331 | 0.548798132696365 | 0 | |  |  |  |
| HIST2H2AC | 1768973 | -5.91623788993776 | -0.994821543745026 | 0.168151038253043 | 0.50179794103987 | 0 | |  |  |  |
| ARSD | 1684873 | -5.90113520328806 | -1.11752609323195 | 0.189374765148454 | 0.46088346252707 | 0 | |  |  |  |
| GTF3C6 | 1691578 | -5.89151337588101 | -1.2222997053447 | 0.207467865616433 | 0.4285989719543 | 0 | |  |  |  |
| HIST2H2AA3 | 1659047 | -5.85729551492682 | -1.11207397874622 | 0.189861340598608 | 0.462628490673216 | 0 | |  |  |  |
| RNH1 | 2375599 | -5.83584219680699 | -0.821610399503434 | 0.140786945876118 | 0.565810008060832 | 0 | |  |  |  |
| BIRC5 | 2349459 | -5.81652325288153 | -0.936946081993875 | 0.161083527265142 | 0.522337402442583 | 0 | |  |  |  |
| SOD2 | 2336781 | -5.80137123905454 | -0.967242896701272 | 0.166726599082272 | 0.511482612786684 | 0 | |  |  |  |
| SERPINB8 | 2397028 | -5.79191979714176 | -0.930141112291499 | 0.160592885410898 | 0.52480700709478 | 0 | |  |  |  |
| FLII | 1737170 | -5.78978125986294 | -0.838410220361302 | 0.144808617585175 | 0.559259506332992 | 0 | |  |  |  |
| ZNF428 | 1652754 | -5.78859680453012 | -1.51790230735028 | 0.262222842358337 | 0.349193278073442 | 0 | |  |  |  |
| FBXW4 | 1671427 | -5.77689671619813 | -0.698550403710354 | 0.120921393964281 | 0.616191034360602 | 0 | |  |  |  |
| WDR6 | 1669484 | -5.77390291736914 | -0.771279036455771 | 0.133580187871812 | 0.585897810429635 | 0 | |  |  |  |
| LIX1L | 1708098 | -5.7578465504784 | -0.716200638160515 | 0.124386892196877 | 0.60869835068458 | 0 | |  |  |  |
| DAB2 | 1764228 | -5.74271316409614 | -0.769614750480232 | 0.134015878642855 | 0.586574089292359 | 0 | |  |  |  |
| DENND2A | 1666503 | -5.71735229664659 | -0.740667469061385 | 0.129547285287249 | 0.598462407044629 | 0 | |  |  |  |
| FNTB | 1728517 | -5.68261958449028 | -0.680238275087289 | 0.119705052392365 | 0.624062196010673 | 0 | |  |  |  |
| PNKP | 1694111 | -5.62775728193348 | -0.752576581281555 | 0.133725842032583 | 0.593542572962346 | 0 | |  |  |  |
| PHF19 | 1713249 | -5.61751164369748 | -0.680079653020758 | 0.121064217781154 | 0.624130814446634 | 0 | |  |  |  |
| NUBP1 | 1689342 | -5.60843345699644 | -0.719165458985202 | 0.128229293348939 | 0.607448725224805 | 0 | |  |  |  |
| TNFRSF12A | 1689004 | -5.59990959156997 | -1.80151357941533 | 0.321704047173781 | 0.286873462312563 | 0 | |  |  |  |
| BRPF3 | 1658800 | -5.5550644714917 | -0.857061185650048 | 0.154284651429059 | 0.552076009176667 | 0 | |  |  |  |
| TOM1 | 2082314 | -5.55297969681115 | -0.607694839833161 | 0.109435811584569 | 0.656244422033316 | 0 | |  |  |  |
| MSRA | 2228180 | -5.551425138255 | -1.10266378693543 | 0.198627155995844 | 0.46565591611833 | 0 | |  |  |  |
| TIMELESS | 1735093 | -5.53881887437604 | -0.772660128061221 | 0.139499078338838 | 0.585337198980185 | 0 | |  |  |  |
| CSNK1D | 1720708 | -5.48389202994892 | -0.650230468124147 | 0.11857098290285 | 0.637178517324602 | 0 | |  |  |  |
| MYO1C | 1812616 | -5.47235654610774 | -0.635458684368206 | 0.116121579252759 | 0.643736115474233 | 0 | |  |  |  |
| SYDE1 | 1755822 | -5.45291247790098 | -0.906292160016278 | 0.166203320462085 | 0.533554607992832 | 0 | |  |  |  |
| WDR4 | 1656628 | -5.44872527814625 | -0.6374294182105 | 0.116986888798946 | 0.642857366726052 | 0 | |  |  |  |
| FAM38A | 1752249 | -5.44111477973802 | -1.24427924258452 | 0.228680940019507 | 0.422118732102231 | 0 | |  |  |  |
| CLCF1 | 1661197 | -5.4348233729417 | -0.808526155219695 | 0.148767696710273 | 0.570964852849364 | 0 | |  |  |  |
| TOB1 | 1672004 | -5.42770436813966 | -0.87596129554591 | 0.161387068294979 | 0.544890674173956 | 0 | |  |  |  |
| TXLNA | 1737084 | -5.41250921326908 | -0.902794169732362 | 0.166797715100255 | 0.534849845885163 | 0 | |  |  |  |
| MCM7 | 1663195 | -5.40215824018968 | -1.07763721115057 | 0.199482718431575 | 0.473804165991025 | 0 | |  |  |  |
| ALDH3B1 | 1728662 | -5.40075470667309 | -0.875246250523485 | 0.162059989401489 | 0.545160806061528 | 0 | |  |  |  |
| CCDC102A | 1796762 | -5.38483398261714 | -0.702761778052649 | 0.13050760345096 | 0.614394932502651 | 0 | |  |  |  |
| NFKBIA | 1773154 | -5.38163037585074 | -0.87472969070473 | 0.162539905124281 | 0.545356036917938 | 0 | |  |  |  |
| C4orf18 | 1672124 | -5.3775236434614 | -0.868706204586675 | 0.161543911693061 | 0.547637746889031 | 0 | |  |  |  |
| TP53I3 | 2358919 | -5.37425176284497 | -0.789853597398334 | 0.146969965727882 | 0.578402784450791 | 0 | |  |  |  |
| CCL2 | 1720048 | -5.37351411841354 | -1.57003332494334 | 0.292179994384545 | 0.336800614338691 | 0 | |  |  |  |
| PDCL3 | 1702806 | -5.3635283916038 | -1.09617772233016 | 0.204376231893569 | 0.467754123796741 | 0 | |  |  |  |
| TEAD2 | 1682781 | -5.36263078150135 | -0.815541236874074 | 0.152078573018176 | 0.568195284296179 | 0 | |  |  |  |
| SUMO3 | 1725642 | -5.33405845073758 | -0.909832504883405 | 0.170570403996528 | 0.532246880846806 | 0 | |  |  |  |
| SFRS1 | 1795341 | -5.31923452388578 | -0.687292389396782 | 0.129208890172172 | 0.621018267056923 | 0 | |  |  |  |
| LOC402560 | 1769288 | -5.31856506291457 | -0.949478886213862 | 0.178521626600831 | 0.517819468964765 | 0 | |  |  |  |
| LOC728635 | 1676014 | -5.31449152218015 | -0.711863861032639 | 0.133947689644749 | 0.610530865959442 | 0 | |  |  |  |
| STAG1 | 1792435 | -5.31429703536424 | -0.687364641332845 | 0.129342533313201 | 0.620987166479665 | 0 | |  |  |  |
| PITRM1 | 1698072 | -5.31363863587236 | -0.881802906100769 | 0.165950860893648 | 0.542688820353759 | 0 | |  |  |  |
| SIX5 | 1793672 | -5.30278472005611 | -0.711320725789126 | 0.134140977494104 | 0.610760757411421 | 0 | |  |  |  |
| MGAT1 | 1761912 | -5.30216097360645 | -0.858110084446009 | 0.161841575296862 | 0.551674773023952 | 0 | |  |  |  |
| CREBBP | 1809583 | -5.27789013396651 | -0.817367466844239 | 0.154866328418618 | 0.567476491516854 | 0 | |  |  |  |
| UFC1 | 2110281 | -5.26706758636743 | -1.13595781126644 | 0.215671774215807 | 0.455032718368457 | 0 | |  |  |  |
| PFKL | 1713037 | -5.26661901393226 | -0.715782905702583 | 0.135909376358734 | 0.608874624856811 | 0 | |  |  |  |
| PDE5A | 2408024 | -5.24507003738628 | -0.630555518500154 | 0.120218703278626 | 0.645927649057453 | 0 | |  |  |  |
| UNC84B | 2099301 | -5.22401128829335 | -0.649921528811322 | 0.12441043729514 | 0.637314977606179 | 0 | |  |  |  |
| RNF145 | 1710906 | -5.19897909753012 | -0.765831074380156 | 0.147304126447436 | 0.588114483625463 | 0 | |  |  |  |
| POLDIP3 | 1688000 | -5.16955561082138 | -0.676849908430503 | 0.130929998511605 | 0.625529613987894 | 0 | |  |  |  |
| ACYP2 | 2158705 | -5.13886112515194 | -0.825631593462294 | 0.160664313231053 | 0.564235133390393 | 0 | |  |  |  |
| MCRS1 | 1784227 | -5.13048067678016 | -0.705739886589715 | 0.137558238896366 | 0.613127965119004 | 0 | |  |  |  |
| KEAP1 | 2410771 | -5.12888188652295 | -0.747645179024695 | 0.145771572745566 | 0.595574884239617 | 0 | |  |  |  |
| CAPNS1 | 1655418 | -5.1259786167091 | -0.605297238230677 | 0.118084230054647 | 0.657335935331582 | 0 | |  |  |  |
| CCND1 | 1688480 | -5.11864270410991 | -0.980900173581376 | 0.191632866422535 | 0.506663507144342 | 0 | |  |  |  |
| ATP8B4 | 1783956 | -5.11362948118223 | -1.02601444855602 | 0.200643095541371 | 0.49106487960415 | 0 | |  |  |  |
| AGPAT2 | 2377430 | -5.10270959444392 | -0.886030059601512 | 0.173639130975877 | 0.541101047911128 | 0 | |  |  |  |
| EWSR1 | 1727041 | -5.08780173472159 | -1.00262724428599 | 0.197064920482962 | 0.499090295083454 | 0 | |  |  |  |
| LOC648024 | 1665736 | -5.08212950335039 | -0.855943356846118 | 0.168422185283126 | 0.552503934407022 | 0 | |  |  |  |
| ZFP36 | 1720829 | -5.05703586875132 | -0.805896018650569 | 0.159361341221722 | 0.572006712144903 | 0 | |  |  |  |
| EWSR1 | 1697735 | -5.05564041799414 | -1.08235517952733 | 0.214088639626148 | 0.472257240378429 | 0 | |  |  |  |
| ITFG2 | 1701244 | -5.04450785757804 | -0.904760751164535 | 0.179355603501612 | 0.534121272497217 | 0 | |  |  |  |
| LOC284023 | 1739325 | -5.04068197258602 | -0.778456062104775 | 0.154434671010479 | 0.582990361920125 | 0 | |  |  |  |
| TMEM87A | 1716816 | -5.03871366317399 | -0.883593425343912 | 0.175360912409402 | 0.54201571064189 | 0 | |  |  |  |
| TAF6L | 1727281 | -5.03808185098428 | -0.69104533766198 | 0.137164372890641 | 0.619404883371041 | 0 | |  |  |  |
| DPP3 | 2349129 | -5.03097685596707 | -0.862911931507168 | 0.171519757735259 | 0.549841638400859 | 0 | |  |  |  |
| MED16 | 1800451 | -5.0291104779177 | -1.14281344037226 | 0.227239676954848 | 0.452875550545461 | 0 | |  |  |  |
| H2AFZ | 1707858 | -5.01592890399625 | -0.677821572398258 | 0.135133807789467 | 0.62510845779387 | 0 | |  |  |  |
| FER1L3 | 2370976 | -5.01535886110531 | -1.16237959685115 | 0.231763993174154 | 0.446775011840207 | 0 | |  |  |  |
| KHDRBS1 | 2076640 | -5.01233724764415 | -0.839378562400962 | 0.167462507195716 | 0.55888425531339 | 0 | |  |  |  |
| RAB23 | 2346997 | -5.00904917397267 | -0.850428125726581 | 0.169778354372215 | 0.55462012579212 | 0 | |  |  |  |
| PFN1 | 1712950 | -5.00546963585288 | -0.775198911607739 | 0.154870365420897 | 0.584308057241531 | 0 | |  |  |  |
| PCBP2 | 1724825 | -4.99671251522826 | -0.871710888548501 | 0.174456882578661 | 0.546498375096559 | 0 | |  |  |  |
| SPATA18 | 1667948 | -4.9675875781306 | -0.707386732442755 | 0.142400455214311 | 0.61242847483712 | 0 | |  |  |  |
| CLIC3 | 1796423 | -4.95172785735169 | -1.24723741955302 | 0.251879233973103 | 0.421254084655065 | 0 | |  |  |  |
| SMARCB1 | 1758823 | -4.94306819830494 | -0.806876817920585 | 0.163234004782147 | 0.571617972258564 | 0 | |  |  |  |
| SERPINB8 | 1718960 | -4.94133770912777 | -0.852792508048117 | 0.172583328290397 | 0.553711922727332 | 0 | |  |  |  |
| EIF4G2 | 1761519 | -4.92815763456726 | -0.655884389741644 | 0.133089166048812 | 0.634686301511035 | 0 | |  |  |  |
| GPX1 | 1749662 | -4.92623057033921 | -0.733754536695923 | 0.148948476166311 | 0.60133692853197 | 0 | |  |  |  |
| DDX56 | 1679405 | -4.92618049062007 | -0.661294980126193 | 0.134240915732861 | 0.632310472823988 | 0 | |  |  |  |
| UBAP2L | 1814789 | -4.92107564763024 | -0.80827036675639 | 0.164246686015813 | 0.571066093351248 | 0 | |  |  |  |
| DDX59 | 1748077 | -4.89387515491956 | -0.682081114697926 | 0.139374441134295 | 0.623265553405556 | 0 | |  |  |  |
| S100A11 | 1750101 | -4.89179723693208 | -0.787012518395181 | 0.160884125051913 | 0.579542947183911 | 0 | |  |  |  |
| TSPAN10 | 1656194 | -4.86966881593947 | -0.915574457595418 | 0.188015754705648 | 0.530132738456725 | 0 | |  |  |  |
| C3orf14 | 2224486 | -4.86534957473983 | -0.756863529488007 | 0.155562003893313 | 0.591781487295865 | 0 | |  |  |  |
| CPT1C | 1773855 | -4.84453161177439 | -0.743660526274877 | 0.153505144742465 | 0.597222106505254 | 0 | |  |  |  |
| SRRM2 | 1734602 | -4.83058764308468 | -0.83403191913224 | 0.172656409686762 | 0.560959329095469 | 0 | |  |  |  |
| LOC728698 | 3226012 | -4.82209256681026 | -0.817850807382046 | 0.169604958024072 | 0.567286403907386 | 0 | |  |  |  |
| ARPC3 | 1655561 | -4.82087327116492 | -0.621791441703816 | 0.128979005820986 | 0.649863469737312 | 0 | |  |  |  |
| XBP1 | 1809433 | -4.81425807616643 | -1.14615945244583 | 0.238076030472906 | 0.451826422946223 | 0 | |  |  |  |
| COPZ2 | 1667361 | -4.81399521863455 | -0.615482269769601 | 0.127852696526811 | 0.652711666049937 | 0 | |  |  |  |
| STAT2 | 1690921 | -4.81394065300673 | -0.764832834987641 | 0.158878741994863 | 0.588521556626299 | 0 | |  |  |  |
| CYP20A1 | 1718177 | -4.78501769981533 | -0.777191237982646 | 0.162421810479957 | 0.583501699091433 | 0 | |  |  |  |
| HCFC1 | 1732705 | -4.78398716370366 | -0.90599567793381 | 0.189380875602603 | 0.533664267783938 | 0 | |  |  |  |
| MEPCE | 2180827 | -4.78140998201634 | -0.610972752380792 | 0.127780875239471 | 0.654755077529084 | 0 | |  |  |  |
| STAG1 | 2151048 | -4.76679995303266 | -0.824982156519745 | 0.173068340322293 | 0.564489184051325 | 0 | |  |  |  |
| DPP3 | 1700822 | -4.76623186026957 | -0.814093706706728 | 0.170804469982433 | 0.56876566996178 | 0 | |  |  |  |
| SLC16A3 | 2364022 | -4.75889014288978 | -0.76408706213034 | 0.160559928720346 | 0.588825859927565 | 0 | |  |  |  |
| LOC653171 | 1715702 | -4.73684854830374 | -0.771101313302695 | 0.162787833606972 | 0.585969990632112 | 0 | |  |  |  |
| PDXDC1 | 3243705 | -4.73567992202461 | -0.6001166913667 | 0.126722392823824 | 0.659700593813073 | 0 | |  |  |  |
| SLC15A3 | 2085862 | -4.72607036859548 | -1.12354542693989 | 0.237733537444934 | 0.458964531689741 | 0 | |  |  |  |
| RAB23 | 1667417 | -4.71755349495656 | -0.90913739142955 | 0.192713742918123 | 0.532503387662641 | 0 | |  |  |  |
| NT5C | 1806432 | -4.71649819526242 | -0.686487652315795 | 0.145550284108102 | 0.621364768446058 | 0 | |  |  |  |
| DDRGK1 | 1797828 | -4.70021141381278 | -1.0084181954374 | 0.214547412159782 | 0.497090971199383 | 0 | |  |  |  |
| ESYT1 | 1761159 | -4.6890183311831 | -0.691361751313019 | 0.147442748669865 | 0.619269049626242 | 0 | |  |  |  |
| MIR1978 | 3310491 | -4.67082405850459 | -0.674402477906594 | 0.144386187417753 | 0.626591681526942 | 0 | |  |  |  |
| PPM1M | 1657810 | -4.66602383807411 | -0.758879939841604 | 0.162639533396561 | 0.590954950313847 | 0 | |  |  |  |
| ERMAP | 1715013 | -4.65466136841359 | -0.982326111216107 | 0.211041369815245 | 0.506162976109917 | 0 | |  |  |  |
| NEXN | 1783276 | -4.65096209385747 | -0.908734607648044 | 0.195386371531217 | 0.532652077209319 | 0 | |  |  |  |
| LOC399942 | 1765701 | -4.6457165908625 | -0.905123671808902 | 0.19482972198286 | 0.533986927225671 | 0 | |  |  |  |
| AURKA | 2357438 | -4.62062579609639 | -0.917668933259243 | 0.19860273775784 | 0.529363660802922 | 0 | |  |  |  |
| HARS2 | 1715113 | -4.61579328600676 | -0.664225751187907 | 0.143902837503918 | 0.631027265941143 | 0 | |  |  |  |
| BCYRN1 | 1678757 | -4.61576263233349 | -0.887039095260335 | 0.192176064047707 | 0.540722728622697 | 0 | |  |  |  |
| TGFB1I1 | 2389876 | -4.60868096043775 | -0.924338534391754 | 0.200564660979257 | 0.526922052686172 | 0 | |  |  |  |
| SGMS2 | 1685176 | -4.60719133257007 | -0.652863926621369 | 0.141705407805839 | 0.636016488904956 | 0 | |  |  |  |
| MPDU1 | 2070815 | -4.60704182736361 | -0.749230569207981 | 0.162627255684529 | 0.594920761273151 | 0 | |  |  |  |
| SSH3 | 1755234 | -4.60249012927942 | -0.912385406624327 | 0.198237341307927 | 0.531305883297876 | 0 | |  |  |  |
| VCAM1 | 2325763 | -4.58090169074326 | -0.718642255310258 | 0.156877903920627 | 0.607669060800291 | 0 | |  |  |  |
| ACOX2 | 1685703 | -4.5764903353496 | -0.730718294417196 | 0.159667832962085 | 0.602603812497801 | 0 | |  |  |  |
| MLKL | 1667825 | -4.57581980524339 | -0.854852933948971 | 0.186819623659438 | 0.552921687749879 | 0 | |  |  |  |
| LOC100131261 | 3289262 | -4.57398637575043 | -0.731905053229096 | 0.160014699018209 | 0.602108315316365 | 0 | |  |  |  |
| HERC4 | 2370160 | -4.57006375377279 | -0.703759186963138 | 0.153993297441891 | 0.613970316640911 | 0 | |  |  |  |
| TK1 | 1806037 | -4.55899070430208 | -0.802912096739337 | 0.176116195188042 | 0.573191016269988 | 0 | |  |  |  |
| AVEN | 1710216 | -4.55648539687859 | -1.06402644126379 | 0.233519115850278 | 0.478295312918603 | 0 | |  |  |  |
| STAT1 | 1690105 | -4.54621935581863 | -1.22671870019292 | 0.269832712454331 | 0.427288175898033 | 0 | |  |  |  |
| MGLL | 1657708 | -4.52819195577153 | -0.617214655271084 | 0.13630487870206 | 0.651928361493972 | 0 | |  |  |  |
| STAT1 | 1691364 | -4.51893740949853 | -1.27366086610178 | 0.28184963647087 | 0.413608899717178 | 0 | |  |  |  |
| PDLIM7 | 1690125 | -4.50436197340202 | -0.692008696809498 | 0.153630791862592 | 0.618991414022633 | 0 | |  |  |  |
| BRD9 | 1651405 | -4.47441504290969 | -0.850483768022171 | 0.190077084907417 | 0.554598735449049 | 0 | |  |  |  |
| NDUFB2 | 2117330 | -4.46992306221846 | -0.616175722259409 | 0.137849290397763 | 0.652398006021832 | 0 | |  |  |  |
| PRIC285 | 1787509 | -4.46214648774465 | -1.21933820644811 | 0.273262702109184 | 0.429479684103694 | 0 | |  |  |  |
| PPRC1 | 1796210 | -4.45477562047756 | -0.651631491298166 | 0.146277062373864 | 0.63656004389429 | 0 | |  |  |  |
| LOC286512 | 3274596 | -4.4448331186167 | -0.752720254671658 | 0.169347247598334 | 0.593483466896853 | 0 | |  |  |  |
| PHF11 | 1776723 | -4.43600586471104 | -1.07653848343319 | 0.242681934214104 | 0.474165143216076 | 0 | |  |  |  |
| SPHK1 | 2357134 | -4.43232767613173 | -0.67078655917531 | 0.151339568775008 | 0.628164118006938 | 0 | |  |  |  |
| STAT6 | 1763198 | -4.4310624555589 | -0.82749851705162 | 0.186749459153639 | 0.563505455546272 | 0 | |  |  |  |
| NACC2 | 1809522 | -4.43022147918616 | -0.628799879502005 | 0.141934186012189 | 0.646714167357979 | 0 | |  |  |  |
| ANKRD35 | 1798006 | -4.42692201270646 | -0.825678100455623 | 0.186512908536835 | 0.56421694489186 | 0 | |  |  |  |
| SPAG7 | 1684446 | -4.424340742578 | -0.725063542317675 | 0.16388058345958 | 0.604970398598722 | 0 | |  |  |  |
| LMNA | 1737394 | -4.42142357568487 | -0.679191867737398 | 0.153613843168643 | 0.624515001441486 | 0 | |  |  |  |
| EIF4G2 | 2380946 | -4.40836191404932 | -0.887124634908779 | 0.201236797750552 | 0.5406906692757 | 0 | |  |  |  |
| LOC644563 | 3207122 | -4.39354727183223 | -1.1438091883187 | 0.260338427596273 | 0.452563083738764 | 0 | |  |  |  |
| LOC652595 | 1702783 | -4.39063421889561 | -0.999768725160338 | 0.227704854314148 | 0.50008016017649 | 0 | |  |  |  |
| PTPLA | 1725791 | -4.38523753937862 | -0.877729391774414 | 0.200155495316403 | 0.544223291948222 | 0 | |  |  |  |
| RHBDF1 | 1808404 | -4.38296407311799 | -0.618704540021643 | 0.141161216405204 | 0.651255456455155 | 0 | |  |  |  |
| SNRPA1 | 1715179 | -4.37034216643151 | -0.988355410708329 | 0.226150578849378 | 0.504052037958183 | 0 | |  |  |  |
| GBP3 | 1725314 | -4.36824032052521 | -0.755476624508391 | 0.172947587374853 | 0.592350657701043 | 0 | |  |  |  |
| HNRNPAB | 1696485 | -4.36736269262806 | -0.687278271202378 | 0.15736688696876 | 0.621024344363127 | 0 | |  |  |  |
| ALG14 | 1743635 | -4.36721321156741 | -0.717158183933867 | 0.164214145083261 | 0.608294479365691 | 0 | |  |  |  |
| FST | 1700081 | -4.36223215500571 | -0.656959033480341 | 0.150601575096473 | 0.634213708430371 | 0 | |  |  |  |
| LYRM2 | 1703132 | -4.35696746485242 | -0.797150212458755 | 0.182959872638332 | 0.575484823443101 | 0 | |  |  |  |
| ZNF394 | 2115949 | -4.35374283530373 | -0.690042945859348 | 0.158494190392669 | 0.619835398557868 | 0 | |  |  |  |
| SON | 2247664 | -4.33277373591277 | -0.862375440119843 | 0.199035419960182 | 0.550046144654471 | 0 | |  |  |  |
| COL1A1 | 1701308 | -4.31988592253843 | -0.946268242710153 | 0.219049359098379 | 0.51897313275483 | 0 | |  |  |  |
| FILIP1L | 2391150 | -4.31409818801025 | -0.670249237305962 | 0.155362536524718 | 0.628398116995679 | 0 | |  |  |  |
| AGPAT2 | 1681081 | -4.3074115209386 | -0.683536119203487 | 0.158688371399105 | 0.622637286848513 | 0 | |  |  |  |
| CYP20A1 | 2391333 | -4.30579587685677 | -0.657262989901024 | 0.15264610973171 | 0.634080102216062 | 0 | |  |  |  |
| KLF2 | 1735930 | -4.28612244453674 | -0.732044574209081 | 0.170794134717773 | 0.602050089095387 | 0 | |  |  |  |
| HNRNPM | 3192791 | -4.28043230985206 | -0.914996442106906 | 0.213762623929574 | 0.530345178589846 | 0 | |  |  |  |
| ASS1 | 1708778 | -4.2754878460897 | -1.06216533104863 | 0.248431376555092 | 0.47891272316694 | 0 | |  |  |  |
| NNMT | 1715508 | -4.27326394469932 | -1.0154374295019 | 0.237625721846991 | 0.494678317504172 | 0 | |  |  |  |
| CXCL10 | 1791759 | -4.26247515785779 | -0.714623552797467 | 0.167654596527108 | 0.609364114494453 | 0 | |  |  |  |
| SULF1 | 1702363 | -4.25707991163912 | -0.908114237576324 | 0.213318579031952 | 0.532881170991679 | 0 | |  |  |  |
| ASS1 | 2395451 | -4.25501646620577 | -0.990448225408107 | 0.232771890138253 | 0.503321375739593 | 0 | |  |  |  |
| DECR2 | 1783337 | -4.25487643849019 | -0.682743839352857 | 0.160461496173347 | 0.622979312321797 | 0 | |  |  |  |
| MUC1 | 1756992 | -4.23792651527204 | -0.87998248950477 | 0.207644584287531 | 0.543374026343961 | 0 | |  |  |  |
| PPAPDC1B | 1675406 | -4.22850368206914 | -0.602499344292759 | 0.14248523581702 | 0.658611978234496 | 0 | |  |  |  |
| S100A16 | 1728049 | -4.22773062999921 | -0.973638438765954 | 0.2302981253955 | 0.509220202189451 | 0 | |  |  |  |
| LOC100132715 | 3211935 | -4.22060472583363 | -0.750946345068989 | 0.177923874385244 | 0.594213651377292 | 0 | |  |  |  |
| TNFRSF11B | 1676663 | -4.21351889292975 | -1.82104485564922 | 0.432190979066196 | 0.283015925982308 | 0 | |  |  |  |
| QRICH1 | 1676002 | -4.19758141803463 | -0.615783734744213 | 0.146699652351837 | 0.652575289932164 | 0 | |  |  |  |
| AHCY | 1657862 | -4.17852923452065 | -0.727640528037284 | 0.174137953140528 | 0.603890746630819 | 0 | |  |  |  |
| ADSS | 1778796 | -4.1674425414433 | -0.667929701013706 | 0.160273283763712 | 0.629409255572501 | 0 | |  |  |  |
| PDGFRB | 1815057 | -4.15161098731954 | -1.01014891997484 | 0.243314925955294 | 0.496494995141717 | 0 | |  |  |  |
| ZMAT3 | 2361104 | -4.14689319603573 | -0.764072113453913 | 0.184251698159078 | 0.588831961156589 | 0 | |  |  |  |
| SMPDL3A | 1796349 | -4.14450184783976 | -0.725232130168491 | 0.174986562147753 | 0.604899708191166 | 0 | |  |  |  |
| LGALS8 | 2356654 | -4.14207057428854 | -0.785379790736544 | 0.189610431944763 | 0.58019919913717 | 0 | |  |  |  |
| GTF2IRD2B | 1760011 | -4.10112345828185 | -0.706724838269849 | 0.172324692357817 | 0.612709515410333 | 0 | |  |  |  |
| ICT1 | 2182198 | -4.1007035060698 | -0.688202597694781 | 0.167825495473182 | 0.620626585038884 | 0 | |  |  |  |
| PIAS3 | 1814966 | -4.09438407171189 | -0.646511569621921 | 0.157902033199248 | 0.638823119202615 | 0 | |  |  |  |
| WDR13 | 1805512 | -4.09408038217963 | -0.640189109975326 | 0.15636945301853 | 0.64162883788955 | 0 | |  |  |  |
| CUL4A | 1695792 | -4.07545542401756 | -0.690988034402343 | 0.169548666961292 | 0.619429486369428 | 0 | |  |  |  |
| HNRNPL | 2389582 | -4.07243309037737 | -0.701629000572291 | 0.172287422531299 | 0.614877533487507 | 0 | |  |  |  |
|  | 1881081 | -4.06893476993998 | -0.804463437159649 | 0.197708609905171 | 0.572574991088717 | 0 | |  |  |  |
| RCC2 | 1720124 | -4.06800356985477 | -0.603048292910439 | 0.148241829820215 | 0.658361422608151 | 0 | |  |  |  |
| MXD4 | 1756541 | -4.03936540036667 | -0.753631293451682 | 0.186571705888076 | 0.59310880991333 | 0 | |  |  |  |
| DIAPH3 | 1874530 | -4.0377956656429 | -0.628792405163791 | 0.155726653162295 | 0.646717517874062 | 0 | |  |  |  |
| HNRNPK | 3260017 | -4.02778644766845 | -0.814008167058284 | 0.202098144386351 | 0.568799393968886 | 0 | |  |  |  |
| HNRNPH1 | 3246409 | -4.02056199068544 | -0.961805730891965 | 0.239221714058933 | 0.513413903024787 | 0 | |  |  |  |
| RPL29 | 1737517 | -4.01965798793684 | -0.751715371422957 | 0.187009783837055 | 0.593896991117162 | 0 | |  |  |  |
| PARP3 | 1796682 | -4.01932042071904 | -0.663514028093577 | 0.165081147716727 | 0.631338646700706 | 0 | |  |  |  |
| RBMX | 2124951 | -4.01781837644818 | -0.644837317862097 | 0.160494392091497 | 0.639564905616035 | 0 | |  |  |  |
| EXD2 | 1771689 | -4.01590322058656 | -0.633434799676397 | 0.157731589852377 | 0.644639814391959 | 0 | |  |  |  |
| KIAA0368 | 1847822 | -4.01536594656937 | -0.771082212216149 | 0.192032861382147 | 0.585977748846622 | 0 | |  |  |  |
| CIRBP | 1674661 | -4.01524735803463 | -0.753798220338449 | 0.18773394342196 | 0.593040188287248 | 0 | |  |  |  |
| NRSN2 | 1770911 | -4.00644715630578 | -0.601585814066664 | 0.15015443623656 | 0.659029150583463 | 0 | |  |  |  |
| XBP1 | 2365465 | -4.00029510594091 | -1.22463003790326 | 0.306134923917124 | 0.427907230526029 | 0 | |  |  |  |
| FLJ10081 | 1745217 | -3.99854064613774 | -0.588391946155795 | 0.147151673129579 | 0.665083808437152 | 0 | |  |  |  |
| RPL29 | 1771051 | -3.99366501773241 | -0.648795395187157 | 0.162456137985138 | 0.637812644811339 | 0 | |  |  |  |
| SLC7A5 | 1720373 | -3.99180419344047 | -0.737368794463161 | 0.184720682360834 | 0.599832337140639 | 0 | |  |  |  |
| MIDN | 1746408 | -3.97334926116706 | -0.734330891220386 | 0.184814080754808 | 0.601096743302844 | 0 | |  |  |  |
| CASP3 | 2388155 | -3.96293759009532 | -0.66108237672812 | 0.166816247215294 | 0.632403660404942 | 0 | |  |  |  |
| LOC340274 | 3275388 | -3.95908525869061 | -0.620289099722904 | 0.156674852697679 | 0.650540553710244 | 0 | |  |  |  |
| PEF1 | 1729748 | -3.94365644411854 | -0.674017134247588 | 0.170911727174612 | 0.626759066437363 | 0 | |  |  |  |
| LRRC41 | 1712755 | -3.94168301146838 | -0.915434106133409 | 0.232244476146342 | 0.530184314515505 | 0 | |  |  |  |
| PIK3IP1 | 1719986 | -3.93577011301459 | -0.864045539469548 | 0.219536587417128 | 0.549409766090579 | 0 | |  |  |  |
| HNRNPM | 3269405 | -3.92486702890787 | -0.84210918729496 | 0.214557380184491 | 0.557827441585533 | 0 | |  |  |  |
| DUSP3 | 1797522 | -3.91840771102615 | -1.13726332900772 | 0.290236088962243 | 0.454621138258468 | 0 | |  |  |  |
| KIAA1600 | 1752927 | -3.91504949242814 | -0.641900733426217 | 0.163957246177265 | 0.640868056352156 | 0 | |  |  |  |
| GTPBP6 | 1776080 | -3.91241744130492 | -0.796333018147412 | 0.203539890641579 | 0.575810891065415 | 0 | |  |  |  |
| LOC286157 | 3292551 | -3.9115394035807 | -0.777480245726902 | 0.198765796661841 | 0.583384820877309 | 0 | |  |  |  |
| RBM3 | 1698213 | -3.88637393392553 | -0.869544160948611 | 0.223741764362418 | 0.547319756407973 | 0 | |  |  |  |
| PPP2R1A | 1810467 | -3.87905673642485 | -0.650470477429003 | 0.167687796706091 | 0.637072523993625 | 0 | |  |  |  |
| CENPN | 1720526 | -3.87270279145786 | -0.756609401988749 | 0.195369859948361 | 0.591885737460479 | 0 | |  |  |  |
| HIST1H2BK | 1813314 | -3.86760241188521 | -1.02621376424171 | 0.265335899338603 | 0.490997041170083 | 0 | |  |  |  |
| KBTBD4 | 1687092 | -3.86338008352575 | -0.668109915612855 | 0.17293403733736 | 0.629330637673857 | 0 | |  |  |  |
|  | 1869109 | -3.8558075372869 | -0.808559374500645 | 0.209699101078468 | 0.570951706049134 | 0 | |  |  |  |
| ASPSCR1 | 1660749 | -3.85210512999174 | -0.732534558603077 | 0.190164736912216 | 0.601845648759363 | 0 | |  |  |  |
| GADD45B | 1718977 | -3.85019141431093 | -0.694510108664947 | 0.180383267721053 | 0.617919107755368 | 0 | |  |  |  |
| ILVBL | 1759419 | -3.84876287907276 | -0.601090016298502 | 0.156177461481679 | 0.659255672006178 | 0 | |  |  |  |
| FAM57A | 1673899 | -3.84846501107966 | -0.676144829192363 | 0.175692081711996 | 0.625835399842846 | 0 | |  |  |  |
| GAPDHL6 | 3267451 | -3.84098361249394 | -0.744677036271913 | 0.193876650202211 | 0.59680145737125 | 0 | |  |  |  |
| AES | 1712944 | -3.83502608775046 | -0.594420415165991 | 0.154997750097357 | 0.662310476775835 | 0 | |  |  |  |
| CTHRC1 | 1725090 | -3.83359515089884 | -1.3039377492406 | 0.340134442452766 | 0.405019212588029 | 0 | |  |  |  |
| GMEB2 | 1683204 | -3.82913127779767 | -0.861617210032185 | 0.225016367296696 | 0.550335305662403 | 0 | |  |  |  |
| LOC653888 | 1804530 | -3.82179098539964 | -0.976449620416252 | 0.255495296353614 | 0.508228920831062 | 0 | |  |  |  |
| SHISA5 | 2139100 | -3.8094280682464 | -0.738905186207046 | 0.193967486186762 | 0.599193888346117 | 0 | |  |  |  |
| SCO2 | 1701621 | -3.80451628347933 | -0.591069420200274 | 0.15535993965039 | 0.663850634981594 | 0 | |  |  |  |
| STAT1 | 1777325 | -3.80082819867854 | -0.713869475119928 | 0.187819453499141 | 0.609682704337412 | 0 | |  |  |  |
| C6orf115 | 1720858 | -3.79904992714836 | -0.885411350493839 | 0.233061256754382 | 0.541333152364572 | 0 | |  |  |  |
| THOC4 | 1750518 | -3.79879778369585 | -0.689207480943484 | 0.181427788523387 | 0.620194449283829 | 0 | |  |  |  |
| CYBA | 1744604 | -3.78693564416918 | -0.706690788506877 | 0.186612832883755 | 0.612723976442897 | 0 | |  |  |  |
| DNAJB2 | 2390974 | -3.78504476494688 | -0.667634879895285 | 0.176387578313002 | 0.629537891283465 | 0 | |  |  |  |
| DHRS3 | 1752478 | -3.77645771139485 | -0.777750152384611 | 0.205947004262189 | 0.583275688514291 | 0 | |  |  |  |
| COL3A1 | 1773079 | -3.76666152023445 | -1.26545321226133 | 0.335961488831247 | 0.415968673264328 | 0 | |  |  |  |
| SMTN | 1785618 | -3.7666504502581 | -0.797303021151119 | 0.211674279756032 | 0.575423871943434 | 0 | |  |  |  |
| LOC648210 | 1715947 | -3.76586212274088 | -0.668595747596731 | 0.177541217868622 | 0.62911874432954 | 0 | |  |  |  |
| GPR68 | 1671142 | -3.76379663938456 | -0.924065305805949 | 0.245514142856838 | 0.527021854652041 | 0 | |  |  |  |
| TNFRSF1A | 1685005 | -3.75955652915049 | -0.601670523233083 | 0.16003763171744 | 0.658990456216664 | 0 | |  |  |  |
| RASSF2 | 2352303 | -3.75200442459223 | -0.786272558912045 | 0.209560669427381 | 0.579840271484653 | 0 | |  |  |  |
| ALDOA | 1736700 | -3.75118062442016 | -0.833858348389283 | 0.222292241264223 | 0.561026822212211 | 0 | |  |  |  |
| CTSK | 1758895 | -3.74530252372375 | -0.61168862788524 | 0.163321553869318 | 0.65443026401306 | 0 | |  |  |  |
| LOC100128288 | 3235637 | -3.74508268907506 | -0.810250235900943 | 0.216350426190738 | 0.570282933604059 | 0 | |  |  |  |
| DAB2 | 2128428 | -3.74124805211922 | -0.752368130293601 | 0.201100841166472 | 0.593628338471312 | 0 | |  |  |  |
| AHNAK | 1714567 | -3.74102126665202 | -0.730885221303965 | 0.195370506930601 | 0.602534092316941 | 0 | |  |  |  |
| FAM173A | 1773780 | -3.73841829716935 | -0.591495457478443 | 0.158220779607866 | 0.663654624491726 | 0 | |  |  |  |
| TDP1 | 2285713 | -3.72570952799374 | -1.07948420317133 | 0.289739228208866 | 0.473197972337944 | 0 | |  |  |  |
| GLE1 | 1754912 | -3.72199606511487 | -0.633845057796115 | 0.17029707896173 | 0.64445652470784 | 0 | |  |  |  |
| FKBP9L | 2089977 | -3.7082781954011 | -0.58857382171899 | 0.158718896130533 | 0.664999968911499 | 0 | |  |  |  |
| SFRS3 | 1723212 | -3.70747060326868 | -0.61137138375218 | 0.164902557342779 | 0.654574187007453 | 0 | |  |  |  |
| LIMS2 | 1760493 | -3.70654070001567 | -0.807816093089413 | 0.217943402883988 | 0.57124593810581 | 0 | |  |  |  |
| ARHGDIA | 1734742 | -3.70354359882495 | -0.85509377373585 | 0.230885299691666 | 0.552829392134293 | 0 | |  |  |  |
| PDCL3 | 1667925 | -3.70052589771979 | -1.01241530541757 | 0.273586872082536 | 0.495715644266068 | 0 | |  |  |  |
| TRIP12 | 1720241 | -3.69477277724112 | -0.87179310626885 | 0.235953104244699 | 0.546467231598442 | 0 | |  |  |  |
| C17orf91 | 2390310 | -3.6670313734714 | -0.724095200278015 | 0.197460868624244 | 0.605376593185069 | 0 | |  |  |  |
| IGFBP6 | 1669362 | -3.66604780579766 | -0.78671105342057 | 0.214593779212707 | 0.579664060898204 | 0 | |  |  |  |
| AGPAT2 | 1732176 | -3.65894143115437 | -0.701493632002425 | 0.191720377382785 | 0.61493523036391 | 0 | |  |  |  |
| ALDOA | 1741148 | -3.656251044602 | -0.64391382185172 | 0.176113131728845 | 0.639974434103176 | 0 | |  |  |  |
| PRC1 | 1728934 | -3.6554289940726 | -0.922624419494792 | 0.252398397285479 | 0.527548478709141 | 0 | |  |  |  |
| AKIRIN2 | 2148847 | -3.64755868992649 | -0.596257441402464 | 0.163467538726424 | 0.661467673966964 | 0 | |  |  |  |
| TMED1 | 1693045 | -3.64051304489891 | -0.799858414338111 | 0.219710355236571 | 0.574405546721824 | 0 | |  |  |  |
| ALG9 | 1732809 | -3.6396731204455 | -0.667776061839318 | 0.183471438159694 | 0.629476287803801 | 0 | |  |  |  |
| LOC644237 | 3283772 | -3.63207514945495 | -0.763264884926856 | 0.210145675273651 | 0.589161521421254 | 0 | |  |  |  |
| BAT1 | 1686626 | -3.62907135926016 | -0.844127258612604 | 0.232601449530244 | 0.557047686651739 | 0 | |  |  |  |
| FLJ21986 | 1677038 | -3.6262855829956 | -0.631637636577063 | 0.174183092346323 | 0.645443341636999 | 0 | |  |  |  |
| ZNF668 | 1739236 | -3.60352130186295 | -0.696963352563023 | 0.193411747615508 | 0.61686925446024 | 0 | |  |  |  |
| ITPA | 2407482 | -3.60267138913906 | -0.654272424133601 | 0.181607577673065 | 0.63539585151261 | 0 | |  |  |  |
| SEH1L | 2381138 | -3.59006654265078 | -0.60272938781333 | 0.167888082477796 | 0.65850696828082 | 0 | |  |  |  |
| C10orf10 | 1767556 | -3.58608020055002 | -0.624105995103929 | 0.174035704781005 | 0.6488217126526 | 0 | |  |  |  |
| MFSD10 | 1674874 | -3.58302560990079 | -0.633039490233105 | 0.176677355719663 | 0.644816474819215 | 0 | |  |  |  |
| HIST1H2BK | 1796179 | -3.57667783036056 | -0.726644780090841 | 0.203161932540507 | 0.604307695825249 | 0 | |  |  |  |
| TYMS | 1806040 | -3.57619570276669 | -0.850912296746411 | 0.237937844421689 | 0.554434025521713 | 0 | |  |  |  |
| APOBEC3F | 2296950 | -3.57503545562151 | -0.747296376574732 | 0.209031878383096 | 0.595718894641988 | 0 | |  |  |  |
| SLC35C1 | 1680104 | -3.56693544317242 | -0.703017566515955 | 0.197092876424669 | 0.614286010519634 | 0 | |  |  |  |
| RAN | 3307930 | -3.55228710084927 | -0.674517084425869 | 0.189882480012555 | 0.626541907571017 | 0 | |  |  |  |
| LRAP | 2123312 | -3.55077449344283 | -0.637250034093375 | 0.179467897854447 | 0.642937304320289 | 0 | |  |  |  |
| TMEM120A | 1654516 | -3.53908627772895 | -0.697102043060985 | 0.196972322332961 | 0.616809955863161 | 0 | |  |  |  |
| TAF7 | 1759460 | -3.52460812286142 | -0.656374374135641 | 0.186226199127853 | 0.634470778777793 | 0 | |  |  |  |
| VPS52 | 1666632 | -3.51745131594674 | -0.712887014885866 | 0.202671466028233 | 0.610098033283832 | 0 | |  |  |  |
| NUCKS1 | 3251404 | -3.51371022191503 | -0.648771311208468 | 0.184639958970458 | 0.637823292379902 | 0 | |  |  |  |
| CMPK1 | 2104877 | -3.50734691869145 | -0.975051088688304 | 0.278002464909312 | 0.508721830884937 | 0 | |  |  |  |
| NFKB1 | 1714965 | -3.49886337803773 | -0.679137886405856 | 0.194102430711867 | 0.624538369361318 | 0 | |  |  |  |
| GINS2 | 1809590 | -3.49818292840038 | -0.614052179724752 | 0.175534611051784 | 0.653358995681402 | 0 | |  |  |  |
| OPA3 | 2284591 | -3.49236785193195 | -0.622414303221608 | 0.178221289855618 | 0.649582961680844 | 0 | |  |  |  |
| RCAN1 | 1712112 | -3.48147552802252 | -0.832650827526791 | 0.239166072208394 | 0.56149659244233 | 0 | |  |  |  |
| PRRX1 | 1739496 | -3.4809332208499 | -0.664665076178456 | 0.190944506547061 | 0.630835136742616 | 0 | |  |  |  |
| SOD2 | 2406501 | -3.45499740566356 | -0.683944716359159 | 0.197958098387574 | 0.622460969751566 | 0 | |  |  |  |
| HNRNPAB | 1651262 | -3.45186478540483 | -0.642652319657685 | 0.186175403618052 | 0.640534276758597 | 0 | |  |  |  |
| APH1B | 1767816 | -3.44685217618589 | -0.612692680651919 | 0.17775426659866 | 0.653974967570481 | 0 | |  |  |  |
| LOC645385 | 1720745 | -3.44590668599145 | -0.688638600757235 | 0.199842498218753 | 0.620439051153125 | 0 | |  |  |  |
| LOC728908 | 3236935 | -3.44336609036488 | -0.606479014150433 | 0.176129693513409 | 0.656797702594313 | 0 | |  |  |  |
| MRPL38 | 1719656 | -3.43435794976657 | -0.594879671725111 | 0.173214231139053 | 0.662099675453656 | 0 | |  |  |  |
| MGLL | 1738589 | -3.43023561940906 | -0.600465493816663 | 0.175050801297465 | 0.659541116333319 | 0 | |  |  |  |
| LOC100130835 | 3265742 | -3.42127856995366 | -1.1769171846764 | 0.343999227368479 | 0.442295606123778 | 0 | |  |  |  |
| LOC392871 | 3241522 | -3.41349695823243 | -0.599199839212512 | 0.175538413112513 | 0.660119975684246 | 0 | |  |  |  |
| PARVA | 1756408 | -3.40674788572471 | -0.669244354057258 | 0.196446692419357 | 0.628835968858232 | 0 | |  |  |  |
| MAGED1 | 2306189 | -3.39274211785777 | -0.776028563149435 | 0.228731962581179 | 0.583972135439346 | 0 | |  |  |  |
| GBE1 | 1789702 | -3.38709526562803 | -1.05024127015203 | 0.310071370241575 | 0.48288740168438 | 0 | |  |  |  |
| ALG14 | 2191822 | -3.38200499569371 | -0.611138018303513 | 0.180702872728358 | 0.654680077268207 | 0 | |  |  |  |
| FDX1L | 1744628 | -3.38109976951968 | -0.750826755657574 | 0.222065838584893 | 0.594262909608692 | 0 | |  |  |  |
| XAF1 | 1742618 | -3.37925126294694 | -1.08810460657756 | 0.321995768266328 | 0.47037894639931 | 0 | |  |  |  |
| CXorf26 | 1768176 | -3.37887642757454 | -1.08646440458071 | 0.321546060611812 | 0.470914024985301 | 0 | |  |  |  |
| HERC6 | 1654639 | -3.37220114649262 | -1.01203494465071 | 0.300111084922474 | 0.495846354939335 | 0 | |  |  |  |
| LOC441481 | 3213925 | -3.36818500216209 | -0.704449317524852 | 0.209148047708975 | 0.613676686776011 | 0 | |  |  |  |
| CLIC1 | 1756982 | -3.36792218413482 | -0.624154163061307 | 0.185323213820525 | 0.648800050489772 | 0 | |  |  |  |
| ARF4 | 1723884 | -3.35161530851375 | -0.762318135419813 | 0.22744798112223 | 0.589548277752162 | 0 | |  |  |  |
| LMNA | 1696749 | -3.35126102341795 | -0.713641923045429 | 0.212947281055889 | 0.609778875393595 | 0 | |  |  |  |
| PRDX2 | 1767766 | -3.35027040200239 | -0.591142502618363 | 0.176446206331599 | 0.663817007236665 | 0 | |  |  |  |
| ILVBL | 1769451 | -3.3448251857211 | -0.642407327460688 | 0.192060060478824 | 0.640643058739721 | 0 | |  |  |  |
| NLRX1 | 1723768 | -3.34432607016121 | -0.692389888058387 | 0.207034204659658 | 0.618827884701376 | 0 | |  |  |  |
| FILIP1L | 1738578 | -3.34052559320298 | -0.703100614718328 | 0.210476044892139 | 0.614250650392123 | 0 | |  |  |  |
| UBXN8 | 1731891 | -3.32279589635507 | -0.696587144206276 | 0.209638860144975 | 0.617030135050361 | 0 | |  |  |  |
| NSMAF | 1716596 | -3.31995668791923 | -0.704409454387713 | 0.212174290390878 | 0.613693643523766 | 0 | |  |  |  |
| FLJ35258 | 1763891 | -3.312579130678 | -0.827989331927641 | 0.249953072595181 | 0.563313779681925 | 0 | |  |  |  |
| PARVA | 3307892 | -3.29672987325917 | -0.596304778877818 | 0.180877658104365 | 0.661445970353153 | 0 | |  |  |  |
| PCBP2 | 2312296 | -3.29173639052389 | -0.718732777850843 | 0.218344573374678 | 0.607630933531567 | 0 | |  |  |  |
| PGAP3 | 1805636 | -3.28152861235138 | -0.729813899493363 | 0.222400590001382 | 0.602981690477045 | 0 | |  |  |  |
| CAP2 | 1691237 | -3.27799551724972 | -0.636263421449193 | 0.19410137021268 | 0.6433771387997 | 0 | |  |  |  |
| PSG3 | 1685312 | -3.27611493332026 | -0.669587343133056 | 0.204384570371115 | 0.628686485963261 | 0 | |  |  |  |
| MLST8 | 1789240 | -3.26983691105996 | -0.623723142890993 | 0.19075053583905 | 0.64899391522075 | 0 | |  |  |  |
| LOC100132805 | 3295874 | -3.26708423786897 | -0.621584651679909 | 0.190256695702879 | 0.649956625193048 | 0 | |  |  |  |
| SFRS5 | 2378868 | -3.250991455479 | -0.655543892111918 | 0.201644298697589 | 0.634836114659113 | 0 | |  |  |  |
| LOC731878 | 1791827 | -3.23366666463569 | -0.913217549612095 | 0.282409303222038 | 0.530999515722227 | 0 | |  |  |  |
| PARP9 | 1731224 | -3.22822291264033 | -0.852526753800527 | 0.264085466484486 | 0.553813929627753 | 0 | |  |  |  |
| LOC646723 | 1694778 | -3.22340555147563 | -0.87393990230017 | 0.271123160999736 | 0.545654668145664 | 0 | |  |  |  |
| DDX3X | 1794392 | -3.22274631743737 | -0.622014010886175 | 0.193007438258678 | 0.649763220952521 | 0 | |  |  |  |
| DNAJB12 | 2333865 | -3.22235407469244 | -0.721587144566375 | 0.223931674744728 | 0.606429926499287 | 0 | |  |  |  |
| UBE2C | 2301083 | -3.21978347059794 | -0.777057530376827 | 0.241338443243987 | 0.583555779980618 | 0 | |  |  |  |
| USP16 | 2397231 | -3.2126018555911 | -0.606411745106511 | 0.188760317140181 | 0.656828328043431 | 0 | |  |  |  |
| C9orf167 | 1813641 | -3.21125854861837 | -0.669476889023901 | 0.208478040272385 | 0.628734620643204 | 0 | |  |  |  |
| SHMT2 | 1661264 | -3.21116789069833 | -0.667533561088391 | 0.207878748109686 | 0.629582104555171 | 0 | |  |  |  |
| LOC100129362 | 3264112 | -3.20237398209591 | -0.712275780116405 | 0.2224211738225 | 0.610356571733342 | 0 | |  |  |  |
| SAMD9L | 1799467 | -3.19413951702532 | -0.819570735653172 | 0.256585766302542 | 0.56661050877002 | 0 | |  |  |  |
| TRPM4 | 1679401 | -3.17493508218135 | -0.592670589542011 | 0.186671719011909 | 0.663114271700433 | 0 | |  |  |  |
| LOC728643 | 2125675 | -3.17266660441501 | -0.603545751642648 | 0.190232957601901 | 0.658134450745398 | 0 | |  |  |  |
| KDELR2 | 1724293 | -3.16756072160248 | -0.68578838645182 | 0.216503627467914 | 0.621666013321763 | 0 | |  |  |  |
| RHBDD2 | 1809437 | -3.16386754952116 | -0.6872965418069 | 0.217233032372331 | 0.62101647962524 | 0 | |  |  |  |
| C3orf34 | 2288483 | -3.15772341906338 | -0.7781313436335 | 0.246421627345787 | 0.583121594813979 | 0 | |  |  |  |
| EWSR1 | 2413251 | -3.14946929212782 | -0.623797886273128 | 0.198064444645429 | 0.648960292908134 | 0 | |  |  |  |
| ISG15 | 2054019 | -3.14800744539827 | -1.17343414306891 | 0.372754564092351 | 0.44336471295359 | 0 | |  |  |  |
| TNFRSF10D | 1666022 | -3.14266806396545 | -0.598092806674889 | 0.190313706220761 | 0.660626704215025 | 0 | |  |  |  |
| ADPRHL2 | 1811480 | -3.1400593506643 | -0.585850671163207 | 0.186573120358144 | 0.6662563710835 | 0 | |  |  |  |
| WDR55 | 1678957 | -3.13920045811235 | -0.607841004669337 | 0.193629241834031 | 0.656177938821769 | 0 | |  |  |  |
| ARFGAP3 | 1731287 | -3.12966502263621 | -0.691159944181254 | 0.220841508334674 | 0.619355680305834 | 0 | |  |  |  |
| CFLAR | 1789830 | -3.12486510521125 | -0.615235616608556 | 0.19688389607044 | 0.652823267708068 | 0 | |  |  |  |
| RIT1 | 1656335 | -3.10997711659993 | -0.66470826124369 | 0.213734132542558 | 0.630816253854668 | 0 | |  |  |  |
| ZFAND2B | 1653718 | -3.10449577048265 | -0.702952788918104 | 0.226430583543304 | 0.614313592832166 | 0 | |  |  |  |
| TMBIM4 | 1664750 | -3.09904750918325 | -0.64476423544401 | 0.208052388204251 | 0.639597304794237 | 0 | |  |  |  |
| HGF | 1801586 | -3.08822094293266 | -0.638768155232738 | 0.206840173367307 | 0.642261109197791 | 0 | |  |  |  |
| FAP | 2232854 | -3.08496037403332 | -0.697263156573587 | 0.226020133821678 | 0.616741077224892 | 0 | |  |  |  |
| UQCC | 1745152 | -3.07582318274716 | -0.687688529322097 | 0.223578693723184 | 0.620847769237967 | 0 | |  |  |  |
| GEMIN4 | 1770206 | -3.0672180547739 | -0.591131706352053 | 0.192725686858813 | 0.663821974864476 | 0 | |  |  |  |
| LOC100132510 | 3280402 | -3.06096563932091 | -0.657906613469407 | 0.214934334779193 | 0.633797285774586 | 0 | |  |  |  |
| TFPI | 1707124 | -3.05635466627717 | -0.712391217117702 | 0.23308525838902 | 0.61030773610862 | 0 | |  |  |  |
| DCN | 1768227 | -3.0561600546725 | -0.746029891488556 | 0.244106944054833 | 0.596242082377777 | 0 | |  |  |  |
| LOC100129585 | 3177271 | -3.05607401687837 | -0.620289099722904 | 0.202969265893795 | 0.650540553710243 | 0 | |  |  |  |
| C6orf125 | 1790461 | -3.05569027862709 | -0.740145095868464 | 0.242218624395731 | 0.598679138449029 | 0 | |  |  |  |
| PNRC2 | 2147306 | -3.04292217842646 | -0.711304946630675 | 0.233757192896238 | 0.610767437508875 | 0 | |  |  |  |
| HSPB6 | 1721283 | -3.04149331692665 | -0.75718326506714 | 0.248951152005902 | 0.591650348957111 | 0 | |  |  |  |
| KRCC1 | 1745620 | -3.01142759651262 | -0.614714073897658 | 0.204127130471119 | 0.653059309807359 | 0 | |  |  |  |
| GRINA | 2370872 | -3.01045754203304 | -0.673947373757595 | 0.223868752290212 | 0.626789373657848 | 0 | |  |  |  |
| WDR37 | 2096405 | -3.00302989610985 | -0.585996835999381 | 0.19513519887314 | 0.666188873575291 | 0 | |  |  |  |
| SLC25A22 | 1687958 | -2.98362911534715 | -0.605686734299803 | 0.203003359628138 | 0.657158492977136 | 0 | |  |  |  |
| HIST2H2AA3 | 2144426 | -2.97761893105181 | -0.81538842818171 | 0.273839079836077 | 0.568255470111141 | 0 | |  |  |  |
| LGALS8 | 2353358 | -2.96760690828058 | -0.85442191377866 | 0.28791613585834 | 0.553086903541965 | 0 | |  |  |  |
| LOC728620 | 3232828 | -2.96583185088445 | -0.598852697726595 | 0.201917279143121 | 0.66027883295804 | 0 | |  |  |  |
| CYP26B1 | 1812297 | -2.95920388194784 | -0.642438885777588 | 0.217098554681104 | 0.640629045109002 | 0 | |  |  |  |
| SPRYD3 | 1788095 | -2.95526794040715 | -0.740719789428879 | 0.250643868632375 | 0.598440703770953 | 0 | |  |  |  |
| SPOCD1 | 1784948 | -2.95421782227733 | -0.785569140637953 | 0.265914427404131 | 0.580123054526635 | 0 | |  |  |  |
| FAM119A | 2049364 | -2.95169076406285 | -0.643267706837263 | 0.217931944182337 | 0.640261112635894 | 0 | |  |  |  |
| LOC647000 | 1703692 | -2.94018364105602 | -0.706187516400501 | 0.240184832858556 | 0.612937757366357 | 0 | |  |  |  |
| EEF1B2 | 1685678 | -2.93371952280838 | -0.658138317954027 | 0.224335800623506 | 0.633695502738544 | 0 | |  |  |  |
| EHD2 | 1808777 | -2.92189256194157 | -0.590623451353536 | 0.202137292468095 | 0.664055877570804 | 0 | |  |  |  |
| MRVI1 | 1766157 | -2.91313113719134 | -0.617255348890248 | 0.211887251147014 | 0.651909973026857 | 0 | |  |  |  |
| ATL3 | 1751086 | -2.91090619535771 | -0.684036069381767 | 0.234990763519849 | 0.622421556092315 | 0 | |  |  |  |
| OAS3 | 1745397 | -2.90676189269694 | -0.769232728749321 | 0.264635617620408 | 0.586729433085462 | 0 | |  |  |  |
| CTHRC1 | 2117508 | -2.90174230938835 | -1.2987879302115 | 0.447588997137817 | 0.406467545531932 | 0 | |  |  |  |
| LOC100133372 | 3243291 | -2.89936472550502 | -0.653620495744979 | 0.225435761839562 | 0.635683040549907 | 0 | |  |  |  |
| PARP9 | 2053527 | -2.89508225187406 | -0.781260599898884 | 0.269857825073244 | 0.58185815429247 | 0 | |  |  |  |
| PDHX | 1652357 | -2.87831065446352 | -0.668550071085426 | 0.232271686882956 | 0.629138662887187 | 0 | |  |  |  |
| IQGAP1 | 1803819 | -2.87224985738063 | -0.615273818781647 | 0.214213194997858 | 0.652805981354006 | 0 | |  |  |  |
| C6orf62 | 1653797 | -2.85036149771016 | -1.15554805172401 | 0.405404034769737 | 0.448895627349339 | 0 | |  |  |  |
| MYOF | 3302919 | -2.84769757944314 | -1.00704956106231 | 0.353636414320103 | 0.497562767787648 | 0 | |  |  |  |
| LOC389322 | 3217285 | -2.80658316767502 | -0.652454498983674 | 0.232472889632616 | 0.636197011936517 | 0 | |  |  |  |
| TNPO2 | 1656066 | -2.80393290689667 | -0.604184392318889 | 0.215477478377893 | 0.657843177536265 | 0 | |  |  |  |
| LOC100128266 | 3260070 | -2.79792020341142 | -0.595875419671552 | 0.212970841321715 | 0.661642852003915 | 0 | |  |  |  |
| IL11 | 1788107 | -2.79675852563497 | -0.694341520814133 | 0.248266525139667 | 0.617991319649293 | 0 | |  |  |  |
| BMPER | 3307916 | -2.79126069652033 | -0.679231730874537 | 0.243342276026488 | 0.624497745692692 | 0 | |  |  |  |
| NCL | 2121437 | -2.78188452435674 | -0.72595049711901 | 0.260956373552879 | 0.604598583020379 | 0 | |  |  |  |
| COL5A2 | 1729117 | -2.78089150774804 | -0.64835025682244 | 0.233144750529111 | 0.638009469971627 | 0 | |  |  |  |
| LOC100128266 | 3191695 | -2.77908674906944 | -0.821029892568852 | 0.295431545216705 | 0.566037722660622 | 0 | |  |  |  |
| RHBDD2 | 2406410 | -2.77023039195011 | -0.631765530808716 | 0.228055230584624 | 0.645386125926761 | 0 | |  |  |  |
| BRD8 | 2349600 | -2.76782058908717 | -0.616351784448438 | 0.222684875919545 | 0.652318394177788 | 0 | |  |  |  |
| HNRPA1L-2 | 2220283 | -2.76604548959445 | -0.699460612008353 | 0.252873864381351 | 0.615802396923286 | 0 | |  |  |  |
| LOC100131609 | 3215712 | -2.76346161509625 | -0.710617307515033 | 0.257147522380289 | 0.611058620104645 | 0 | |  |  |  |
| SLC16A5 | 1755649 | -2.75756823304538 | -0.635072510227176 | 0.23030164861082 | 0.643908450935756 | 0 | |  |  |  |
| LOC100133012 | 3240003 | -2.75273912661706 | -0.591804396791269 | 0.214987461423037 | 0.663512524431143 | 0 | |  |  |  |
| PRKAR1A | 1738632 | -2.74556958265473 | -0.68375536645775 | 0.249039532917836 | 0.622542671465961 | 0 | |  |  |  |
| SNHG9 | 3238785 | -2.74113331777262 | -0.748075368712982 | 0.272907327732914 | 0.595397319354903 | 0 | |  |  |  |
| C6orf136 | 1813236 | -2.73947920774048 | -0.591136689244196 | 0.215784331406467 | 0.663819682108559 | 0 | |  |  |  |
| C15orf52 | 1775330 | -2.73798094357979 | -0.662508314362851 | 0.241969658669957 | 0.631778911130812 | 0 | |  |  |  |
| FIS1 | 1658351 | -2.73722047595674 | -0.701162269674961 | 0.256158492103156 | 0.615076486669651 | 0 | |  |  |  |
| CTSO | 1769633 | -2.73442060429914 | -0.665766295341911 | 0.243476184422825 | 0.630353799620541 | 0 | |  |  |  |
| ARMCX6 | 1716026 | -2.71355551683581 | -0.813282325769551 | 0.299710958822723 | 0.569085637387197 | 0 | |  |  |  |
| BRD2 | 1758918 | -2.70152246767539 | -0.731155127961675 | 0.270645584743488 | 0.602421377746832 | 0 | |  |  |  |
| C14orf173 | 2393450 | -2.68729334402638 | -0.628642918399521 | 0.233931632286047 | 0.646784531841093 | 0 | |  |  |  |
| SFRS2 | 1696407 | -2.66960493476113 | -0.679490010783915 | 0.254528301898241 | 0.624385954371978 | 0 | |  |  |  |
| TUBB | 2101885 | -2.6654976767367 | -0.771375372370524 | 0.289392626038563 | 0.585858688428469 | 0 | |  |  |  |
| HNRPUL1 | 2395728 | -2.65849250979591 | -0.716932292823414 | 0.269676250800666 | 0.60838973100897 | 0 | |  |  |  |
| PPP1CA | 1695827 | -2.65672774352486 | -0.591654079544974 | 0.222700305286076 | 0.663581660713465 | 0 | |  |  |  |
| IFI27 | 2058782 | -2.63775187516454 | -0.78607075178028 | 0.298007844930921 | 0.579921386400382 | 0 | |  |  |  |
| HIST1H4H | 1751120 | -2.63161031333159 | -0.593590763624294 | 0.225561801691229 | 0.662691461613061 | 0 | |  |  |  |
| LOC100130516 | 3231944 | -2.6097001883394 | -0.603951026870223 | 0.231425444795836 | 0.65794959662059 | 0 | |  |  |  |
| KDM3B | 1706539 | -2.60743253590535 | -0.589095364429888 | 0.225929283430278 | 0.664759610974122 | 0 | |  |  |  |
| TNS3 | 1667893 | -2.59075675613717 | -0.64797570942974 | 0.250110593321727 | 0.638175129240567 | 0 | |  |  |  |
| NFIC | 1675130 | -2.57118319361602 | -0.633866650328732 | 0.24652722213748 | 0.644446879326105 | 0.0359206925696922 | |  |  |  |
| SEC24D | 1656386 | -2.56669564730001 | -0.958909840075247 | 0.373597018050798 | 0.514445502749703 | 0.0359206925696922 | |  |  |  |
| GLS | 2188722 | -2.56237319108775 | -0.889183399845584 | 0.347015572492826 | 0.539919639302954 | 0.0359206925696922 | |  |  |  |
|  | 1882590 | -2.55388373269061 | -0.717695505803214 | 0.281021213540954 | 0.608067966444697 | 0.0359206925696922 | |  |  |  |
| RASSF2 | 1709683 | -2.54711534698997 | -0.789773040642033 | 0.310065675500459 | 0.578435082027002 | 0.0359206925696922 | |  |  |  |
| AXL | 2364521 | -2.54498404542589 | -0.606147651822968 | 0.238173458459357 | 0.656948575096825 | 0.0359206925696922 | |  |  |  |
| RCAN1 | 2367239 | -2.54262672147089 | -0.651241995229041 | 0.256129611841844 | 0.636731924367785 | 0.0359206925696922 | |  |  |  |
| NFIX | 1694325 | -2.53800595662104 | -0.62717213473551 | 0.247112160276602 | 0.647444245198369 | 0.0359206925696922 | |  |  |  |
|  | 1912662 | -2.52790261199213 | -0.888332986253293 | 0.35141107969869 | 0.540237995116724 | 0.0359206925696922 | |  |  |  |
| LOC100130561 | 3265797 | -2.51597038464619 | -0.7048080857591 | 0.280133697145332 | 0.613524097128316 | 0.0359206925696922 | |  |  |  |
| P8 | 1810560 | -2.50822421107594 | -0.727403010178499 | 0.290007171992997 | 0.603990176268302 | 0.0359206925696922 | |  |  |  |
| CMPK1 | 1738642 | -2.50251622318466 | -0.954248344476097 | 0.381315547781639 | 0.516110417222286 | 0.0359206925696922 | |  |  |  |
| SPARC | 1796734 | -2.47794726209186 | -0.981555423898093 | 0.396116349574557 | 0.506433440475515 | 0.0359206925696922 | |  |  |  |
| HERC4 | 1678922 | -2.46979943017424 | -0.800238775104975 | 0.324009620104463 | 0.574254126962473 | 0.0359206925696922 | |  |  |  |
| ELF1 | 1664010 | -2.46829868656095 | -0.631768022254787 | 0.255952825196784 | 0.645385011385369 | 0.0359206925696922 | |  |  |  |
| PRDX2 | 2382829 | -2.46027893023405 | -0.690702348586183 | 0.280741480203009 | 0.619552159377751 | 0.0359206925696922 | |  |  |  |
| TOP2A | 1686097 | -2.44721925595619 | -0.731658400068049 | 0.298975418033058 | 0.602211264734706 | 0.0359206925696922 | |  |  |  |
| RBX1 | 1666670 | -2.44202020713228 | -0.775919770004328 | 0.317736834338283 | 0.584016174241032 | 0.0359206925696922 | |  |  |  |
| BUB3 | 2386100 | -2.43923581908363 | -0.806716534890006 | 0.330725110126118 | 0.57168148238928 | 0.0359206925696922 | |  |  |  |
| VCP | 1777220 | -2.43431561180508 | -0.626832467587807 | 0.257498437979043 | 0.647596696980793 | 0.0359206925696922 | |  |  |  |
| ADAMTS19 | 1656171 | -2.41835735142754 | -0.744076597768761 | 0.307678514645298 | 0.597049893210864 | 0.0359206925696922 | |  |  |  |
| TRIOBP | 1735788 | -2.39743334966367 | -0.631620196454564 | 0.263456832509305 | 0.645451144172298 | 0.0359206925696922 | |  |  |  |
| CLIP1 | 2404085 | -2.39397570662846 | -0.789473236631469 | 0.32977495738389 | 0.578555298131264 | 0.0359206925696922 | |  |  |  |
| HNRNPAB | 2335718 | -2.3936648811365 | -0.682643351027986 | 0.28518751994384 | 0.623022706335099 | 0.0359206925696922 | |  |  |  |
| IFITM1 | 1801246 | -2.35262952683868 | -0.811187019623701 | 0.344800152497331 | 0.569912752556869 | 0.0359206925696922 | |  |  |  |
| GABARAPL1 | 2151281 | -2.35155784135009 | -0.68277124525964 | 0.29034848016651 | 0.622967478115459 | 0.0359206925696922 | |  |  |  |
| NNT | 2336647 | -2.32458420469474 | -0.601122405097427 | 0.258593517018398 | 0.659240871747555 | 0.0521258231948829 | |  |  |  |
| SFRS5 | 1761996 | -2.31748342581905 | -0.608050286139314 | 0.26237524694461 | 0.65608275867645 | 0.0521258231948829 | |  |  |  |
| NUSAP1 | 1726720 | -2.31219506080219 | -0.626139845580024 | 0.270798885524299 | 0.647907675668947 | 0.0521258231948829 | |  |  |  |
| ARMCX2 | 1785170 | -2.30998861302464 | -0.600296075483823 | 0.259869711954039 | 0.659618572007788 | 0.0521258231948829 | |  |  |  |
| IFIT1 | 1707695 | -2.29045434999516 | -0.978957676127892 | 0.427407634703551 | 0.507346156920233 | 0.0521258231948829 | |  |  |  |
| GTF2IP1 | 1815668 | -2.26411843162962 | -0.590030487188598 | 0.26060054056621 | 0.664328868229986 | 0.0521258231948829 | |  |  |  |
| HNRPC | 1695588 | -2.25981104868773 | -0.74004377706157 | 0.327480378278225 | 0.598721184470054 | 0.0521258231948829 | |  |  |  |
| HK2 | 2156172 | -2.251971194328 | -0.667659794355997 | 0.296477946093459 | 0.629527019643109 | 0.0521258231948829 | |  |  |  |
| SBDSP | 1765725 | -2.24744937444068 | -0.651075068342272 | 0.28969509869574 | 0.636805601634337 | 0.0521258231948829 | |  |  |  |
| PRKAR1A | 2389590 | -2.24450924420331 | -0.731585317649962 | 0.325944443997885 | 0.602241771646374 | 0.0521258231948829 | |  |  |  |
| ADNP | 1657993 | -2.22526076696354 | -0.615211532629868 | 0.276467163652622 | 0.652834165862187 | 0.0521258231948829 | |  |  |  |
| ACOT1 | 1715543 | -2.21985483683076 | -0.602488548026449 | 0.271408984961652 | 0.658616906910839 | 0.0521258231948829 | |  |  |  |
| DDX5 | 1805344 | -2.21935795516681 | -0.753850540705945 | 0.339670551544393 | 0.593018681650647 | 0.0521258231948829 | |  |  |  |
| EID2B | 2051900 | -2.20522163355118 | -0.845539078052931 | 0.383425894789231 | 0.556502827172368 | 0.0521258231948829 | |  |  |  |
| PDE5A | 1810836 | -2.20468653730696 | -0.821333848989534 | 0.372539966608042 | 0.565918478695574 | 0.0521258231948829 | |  |  |  |
| KPNB1 | 1703949 | -2.17203005064307 | -0.647172633312803 | 0.29795749516504 | 0.638530468282497 | 0.0521258231948829 | |  |  |  |
| GPX7 | 1726030 | -2.13153370773921 | -0.616900733066119 | 0.289416362887561 | 0.652070232818396 | 0.0521258231948829 | |  |  |  |
| REPIN1 | 2404385 | -2.11945358759696 | -0.631272224486625 | 0.297846684721396 | 0.64560684304615 | 0.0521258231948829 | |  |  |  |
| PHF23 | 1746135 | -2.09690742460615 | -1.14243806249754 | 0.54482045754219 | 0.452993400524172 | 0.0848201265005491 | |  |  |  |
| DDAH1 | 1668507 | -2.08148444857173 | -0.818386468287345 | 0.393174433202661 | 0.567075814188672 | 0.0848201265005491 | |  |  |  |
| ABCF1 | 2392635 | -2.07146797562685 | -0.58836204880294 | 0.284031448096558 | 0.665097591288531 | 0.0848201265005491 | |  |  |  |
| P4HA1 | 1693334 | -2.06603808735968 | -1.38467887255089 | 0.67020975122509 | 0.382974735591779 | 0.0848201265005491 | |  |  |  |
| SBDS | 1679045 | -2.05243663519759 | -0.691809381123804 | 0.337067351683285 | 0.619076936754356 | 0.0848201265005491 | |  |  |  |
| NUPR1 | 2404688 | -2.02578206962604 | -0.80982336814075 | 0.39975838481493 | 0.570451695129742 | 0.0848201265005491 | |  |  |  |
| ADAMTS5 | 1681886 | -2.01129845740462 | -0.849069457135773 | 0.422149907195479 | 0.555142689371406 | 0.0848201265005491 | |  |  |  |
| BAG2 | 3244893 | -2.01076924437474 | -1.0209235937506 | 0.50772787409928 | 0.492800766889782 | 0.0848201265005491 | |  |  |  |
| PRDX3 | 2395974 | -2.00538263084789 | -0.65313217231503 | 0.325689552840538 | 0.635898242969788 | 0.0848201265005491 | |  |  |  |
| VCAM1 | 1766955 | -1.96692661843174 | -1.31882995288998 | 0.670502875161406 | 0.400859910340856 | 0.0848201265005491 | |  |  |  |
| ACTC1 | 1658660 | -1.95893370521367 | -0.624424900201038 | 0.318757545770509 | 0.648678307651348 | 0.0848201265005491 | |  |  |  |
| IFIT2 | 1739428 | -1.9255179552563 | -0.738561366649225 | 0.383565037465942 | 0.599336703786869 | 0.0848201265005491 | |  |  |  |
| MGC3032 | 1862180 | -1.91376126302856 | -0.598948203159323 | 0.312969132947898 | 0.660235124394102 | 0.0848201265005491 | |  |  |  |
| AIDA | 3240117 | -1.89901147373978 | -0.70099035989605 | 0.369134346784946 | 0.615149782799377 | 0.0848201265005491 | |  |  |  |
| CAV1 | 1687583 | -1.89523681771523 | -0.643826621239227 | 0.33970774270594 | 0.640013117156508 | 0.0848201265005491 | |  |  |  |
| UBQLN2 | 1680770 | -1.87173212814637 | -0.642137420802977 | 0.343071217909212 | 0.64076292468344 | 0.200308840224877 | |  |  |  |
| EPSTI1 | 2388547 | -1.86649531664195 | -0.711451941948873 | 0.381169958266416 | 0.61070520995723 | 0.200308840224877 | |  |  |  |
| FAM45A | 1691760 | -1.84580055121174 | -0.819424570816997 | 0.443939931797646 | 0.566667917110183 | 0.200308840224877 | |  |  |  |
| CUL4A | 2331163 | -1.83854443938718 | -0.79412559692836 | 0.431931684606468 | 0.576692595126919 | 0.200308840224877 | |  |  |  |
| LOC643167 | 3278995 | -1.8189569685774 | -0.680681752487956 | 0.374215423589879 | 0.623870391824682 | 0.200308840224877 | |  |  |  |
| TOR1AIP1 | 2141941 | -1.80443606451324 | -0.686206118909753 | 0.380288408331532 | 0.621486035938194 | 0.200308840224877 | |  |  |  |
| TMEM16A | 2091310 | -1.79126820804239 | -0.96261462038307 | 0.53739279023719 | 0.513126123073369 | 0.200308840224877 | |  |  |  |
| KIAA0240 | 1696127 | -1.76879983594526 | -0.586432008579811 | 0.331542324158129 | 0.665987955568539 | 0.200308840224877 | |  |  |  |
| RGS2 | 2197365 | -1.75949473349746 | -1.36588423387204 | 0.776293448265676 | 0.387996559882772 | 0.200308840224877 | |  |  |  |
| HNRPA2B1 | 2369682 | -1.73818155792891 | -1.36870039841448 | 0.787432355481512 | 0.387239922915237 | 0.200308840224877 | |  |  |  |
| LOC730278 | 3241234 | -1.70859507056617 | -0.879184396279974 | 0.514565686993725 | 0.543674701877703 | 0.200308840224877 | |  |  |  |
| KPNA1 | 1676005 | -1.69713158333798 | -0.730896848052297 | 0.430665986790925 | 0.602529236485442 | 0.200308840224877 | |  |  |  |
| MYADM | 2350574 | -1.68112801751121 | -1.0390687954869 | 0.61807832875522 | 0.486641480833233 | 0.200308840224877 | |  |  |  |
| LOC100132863 | 3205271 | -1.67192325289062 | -0.912005045857461 | 0.545482601716722 | 0.531445978432093 | 0.200308840224877 | |  |  |  |
| SFRS10 | 1742798 | -1.61951187474496 | -0.608450578474749 | 0.375699979705656 | 0.655900746219565 | 0.378417023480785 | |  |  |  |
| SLC38A2 | 1651799 | -1.61880532341814 | -1.49926629073796 | 0.926156017063387 | 0.353733242442655 | 0.378417023480785 | |  |  |  |
| MORF4L1 | 1760676 | -1.60598787376834 | -1.35783686306218 | 0.845483882687173 | 0.390166856844627 | 0.378417023480785 | |  |  |  |
| SMNDC1 | 1691789 | -1.60470377405276 | -0.747431745144598 | 0.465775526443064 | 0.595663000756359 | 0.378417023480785 | |  |  |  |
| ZC3H11B | 3303965 | -1.5994228880354 | -0.692807620516317 | 0.433161001820666 | 0.618648728979203 | 0.378417023480785 | |  |  |  |
| SYNCRIP | 1727740 | -1.58770527068366 | -1.17497302625886 | 0.740044797957322 | 0.442892040125539 | 0.378417023480785 | |  |  |  |
| LOC220433 | 1795243 | -1.5761112880914 | -0.817967074865365 | 0.51897799415921 | 0.567240687866952 | 0.378417023480785 | |  |  |  |
| LOC100131940 | 3275696 | -1.57443389986693 | -0.97123668475335 | 0.61687993686838 | 0.510068642239958 | 0.378417023480785 | |  |  |  |
| CCT6A | 1719611 | -1.56540060624144 | -0.614790478243842 | 0.392736834132169 | 0.653024725056634 | 0.378417023480785 | |  |  |  |
| LOC440595 | 3279414 | -1.55243669035079 | -0.601839941565923 | 0.387674386534841 | 0.658913074298657 | 0.378417023480785 | |  |  |  |
| COL1A2 | 2104356 | -1.53094078569716 | -0.828789086116486 | 0.54135933529204 | 0.563001594712117 | 0.378417023480785 | |  |  |  |
|  |  |  |  |  | | |  |  | |  |
