# Supplementary material for: Epigenetic and Metabolic Reprogramming of Fibroblasts in Crohn’s Disease Strictures Reveals Histone Deacetylases as Therapeutic Targets
Source: J Crohns Colitis. 2023 Dec 9;18(6):895–907. doi: 10.1093/ecco-jcc/jjad209 (PMC11147807; doi:10.1093/ecco-jcc/jjad209)
Supplement: jjad209_suppl_Supplementary_Tables_10 [file jjad209_suppl_supplementary_tables_10.docx]

**Supplementary Table 10. Enriched pathways associated with genes up- and down-regulated by valproic acid (illumina HT-12 array).**

| **Enriched pathways associated with genes up-regulated by VPA** | | | | |
| --- | --- | --- | --- | --- |
| Term | P-value | Adjusted P-value | Odds Ratio | Combined Score |
| Epithelial Mesenchymal Transition | 7.51E-11 | 3.53E-09 | 4.8647343 | 113.4066408 |
| Estrogen Response Early | 1.98E-06 | 4.64E-05 | 3.507251692 | 46.0654445 |
| Estrogen Response Late | 7.01E-06 | 1.10E-04 | 3.324022346 | 39.4485039 |
| Coagulation | 9.46E-05 | 0.001111539 | 3.434869811 | 31.82703367 |
| Xenobiotic Metabolism | 2.28E-04 | 0.0017887 | 2.789361081 | 23.38782885 |
| Glycolysis | 2.28E-04 | 0.0017887 | 2.789361081 | 23.38782885 |
| Fatty Acid Metabolism | 4.22E-04 | 0.002831035 | 2.951387819 | 22.93626828 |
| p53 Pathway | 6.52E-04 | 0.00382835 | 2.616005537 | 19.19108857 |
| Angiogenesis | 0.00138787 | 0.006859379 | 5.586666667 | 36.76017792 |
| Apoptosis | 0.00152553 | 0.006859379 | 2.67518503 | 17.34968848 |
| Complement | 0.00175133 | 0.006859379 | 2.445012788 | 15.51942343 |
| mTORC1 Signaling | 0.00175133 | 0.006859379 | 2.445012788 | 15.51942343 |
| UV Response Up | 0.00358453 | 0.01295946 | 2.514939163 | 14.16194301 |
| Cholesterol Homeostasis | 0.00412137 | 0.013836018 | 3.389358703 | 18.6129019 |
| Hypoxia | 0.00442166 | 0.013854527 | 2.276342422 | 12.34060005 |
| Reactive Oxygen Species Pathway | 0.0068078 | 0.019997926 | 3.895045501 | 19.4350524 |
| KRAS Signaling Up | 0.01045872 | 0.028915273 | 2.109954908 | 9.622068731 |
| Bile Acid Metabolism | 0.01628098 | 0.042511437 | 2.442150337 | 10.05618403 |
|  |  |  |  |  |
| **Enriched pathways associated with genes down-regulated by VPA** | | | | |
| Term | P-value | Adjusted P-value | Odds Ratio | Combined Score |
| Interferon Gamma Response | 2.12E-10 | 9.95E-09 | 5.328267477 | 118.694068 |
| G2-M Checkpoint | 5.76E-09 | 1.35E-07 | 4.827438686 | 91.58773945 |
| Interferon Alpha Response | 5.16E-08 | 8.08E-07 | 6.725598186 | 112.8602346 |
| Epithelial Mesenchymal Transition | 1.29E-07 | 1.51E-06 | 4.341232482 | 68.8728167 |
| Apoptosis | 1.87E-06 | 1.76E-05 | 4.343095238 | 57.28500879 |
| UV Response Dn | 3.81E-05 | 2.99E-04 | 3.942191142 | 40.11020931 |
| TNF-alpha Signaling via NF-kB | 1.17E-04 | 7.84E-04 | 3.186311787 | 28.85115012 |
| Estrogen Response Late | 3.80E-04 | 0.001983603 | 2.965228986 | 23.35344143 |
| E2F Targets | 3.80E-04 | 0.001983603 | 2.965228986 | 23.35344143 |
| IL-2/STAT5 Signaling | 0.00109985 | 0.004930895 | 2.762305487 | 18.81844337 |
| mTORC1 Signaling | 0.00115404 | 0.004930895 | 2.747311828 | 18.58415527 |
| PI3K/AKT/mTOR Signaling | 0.00221421 | 0.008672318 | 3.405605066 | 20.81798728 |
| Myogenesis | 0.00326565 | 0.010963251 | 2.53250508 | 14.49681047 |
| Myc Targets V1 | 0.00326565 | 0.010963251 | 2.53250508 | 14.49681047 |
| IL-6/JAK/STAT3 Signaling | 0.00934162 | 0.029270425 | 3.169299065 | 14.8110063 |
| Unfolded Protein Response | 0.01182483 | 0.034735432 | 2.761262707 | 12.25325207 |
